# One Size Fits All? Development of the CPOSS209 Data Set of Experimental and Hypothetical Polymorphs for Testing Computational Modeling Methods

**DOI:** 10.1021/acs.cgd.5c00255

**Published:** 2025-04-28

**Authors:** Louise S. Price, Matteo Paloni, Matteo Salvalaglio, Sarah L. Price

**Affiliations:** † Department of Chemistry, University College London, 20 Gordon Street, London WC1H 0AJ, U.K.; ‡ Department of Chemical Engineering, University College London, Torrington Place, London WC1E 7JE, U.K.

## Abstract

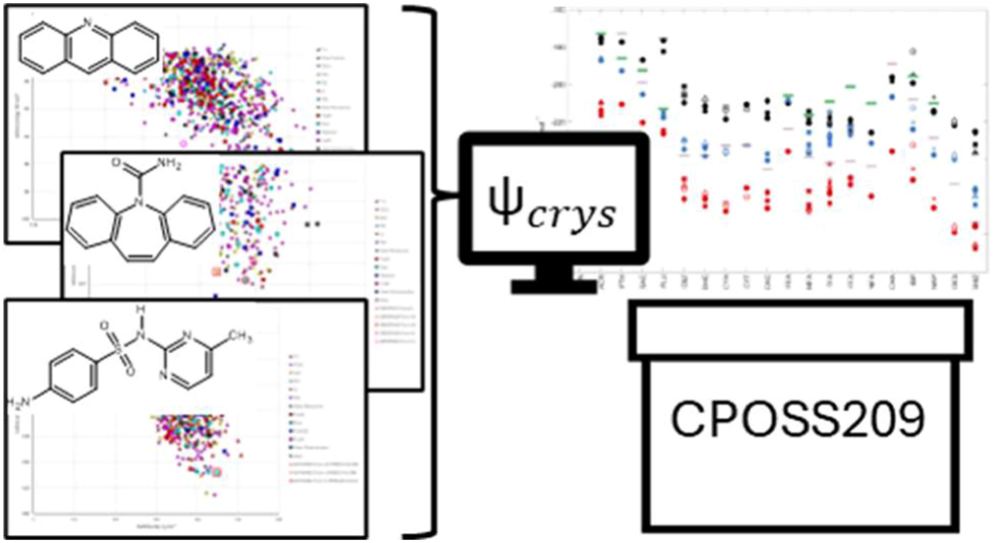

Organic crystal structure prediction (CSP) studies have led to the rapid development of methods for predicting the relative energies of known and computer-generated crystal structures. There is a compromise between the level of theoretical treatment, its reliability across different types of organic systems, how its accuracy depends on the size and shape of the unit cell, and the size and the number of structures that can be modeled at an affordable computational cost. We have used our database of crystal structure prediction studies, often performed as a complement to experimental screening, to produce sets comprising 6 to 15 crystal structures, covering known polymorphs, observed packings of closely related molecules, and CSP-generated energetically competitive but distinct structures, for 20 organic molecules. These have been chosen to illustrate some of the issues that need consideration in any lattice energy method, seeking to be generally applicable to moderate-sized organic molecules, including small drug molecules. We included the methods of crystallization reported for the experimental polymorphs. In all of the examples, the original CSP used electronic structure calculations on the molecule to give the conformational energy and an anisotropic atom–atom model for the electrostatic intermolecular energy, combined with an empirical “exp-6” repulsion dispersion model to give the intermolecular lattice energy. The lattice energies and structures are compared with those obtained by reoptimizing with periodic, plane-wave, dispersion-corrected density functional theory, specifically PBE with the TS dispersion correction, and with single point energies where the many body dispersion (MBD) dispersion correction is applied, as an example of a widely used “workhorse” method. The use of this data set for a preliminary test of modeling methods is illustrated for two Machine Learned Foundation Models, MACE-MP-0 and MACE-OFF23. The challenges in modeling the putative and observed polymorphs for a range of molecules, their energies, and the possible level of agreement with experimental data are illustrated. Very similar molecules can differ significantly in the polymorphs observed, only partially reflecting the range of polymorph screening experiments used and the energetically competitive structures produced by CSP approaches based on a purely thermodynamic paradigm.

## Abstract 2

Derivation and illustrative use of the CPOSS209 dataset of 209 experimental and hypothetical crystal structures of 20 small pharmaceutical molecules and precursors, as optimized by two well-established lattice energy models used in CSP, discussed in the context of current experimental knowledge.

## 1 Introduction

Organic polymorphs, different crystalline forms of the same molecule, can differ in their physical properties, such as solubility, morphology, and stability. Hence, the quality control of pharmaceuticals and other specialty products, such as organic semiconductors, depends on controlling their polymorphism. One can envisage being able to design the industrial crystallization process for an organic molecule from the chemical diagram (i.e., potentially before synthesis),^1^ by using crystal structure prediction (CSP)^2−4^ to predict the crystal structures, then calculating the growth rates, solubilities, and morphologies in different solvents and using a 3-D population balance model to optimize the yield and particle size distribution of the crystalline product. However, a recent assessment^1^ of this digital design vision, considering olanzapine and succinic acid, identified some major challenges. One was the accuracy of current methods of modeling the structure and thermodynamics of organic crystals. The development of first-principles methods for calculating the lattice energies of polymorphs is an extremely active field.^5−8^ However, the digital design vision also requires force fields that can realistically model the dynamic motion within the crystals at ambient conditions, both to reduce the lattice energy landscape to those that are distinct and stable at practically important temperatures and pressures,^9−12^ and to simulate crystal growth rates, etc. A second major challenge is predicting which of the thermodynamically plausible crystal structures will actually be experimentally realized, which is related to the relative nucleation, growth, and transformation rates of different structures in the crystallization experiments that can be performed. This paper aims to provide a data set of crystal structures of organic molecules, both observed and computer-generated, alongside the crystallization conditions for the observed structures, to aid researchers working on these major challenges.^13^

Uncertainty as to whether all polymorphs are known is a major frustration for developing first-principles modeling of organic polymorphism and the main reason why CSP is being developed. CSP has become a basic technology in pharmaceutical development^14,15^ for evaluating the risk of the late appearance of a more stable form that has not crystallized yet in the experimental polymorph screening work and which may threaten the reliable manufacture of the drug substance. The lack of any standard polymorph screening protocol that can cover all crystallization methods that can produce novel polymorphs means that computational work on the thermodynamics of organic crystals tends to rely on the hierarchy of theoretical methods.^6,16^ The X23 data set of 23 small-molecule crystal structures and lattice energies,^17,18^ based on the C21 compilation,^19^ has recently been used to show that Diffusion Monte Carlo and other extremely high-level methods are now converging energies to within the spread of experimental values.^7^ It is now timely to develop a benchmark set for larger molecules, which include those with conformational polymorphs.^20^ POLY59^21^ provided a data set of both the experimental crystal structures of the five systems used in the Cambridge Crystallographic Data Centre’s sixth blind test of crystal structure prediction,^22^ and enough hypothetical energetically competitive structures to give a set of 10 structures per compound. This combination of experimental and hypothetical polymorphs provides the ability to test the reproducibility of polymorphic energy differences and ranking. Comparisons with experiments rely on the assumption that the observed low-temperature structure should be the most stable and hence on consideration of the available thermodynamic data on the polymorphs with known crystal structures.

We have used our database of CSP studies built over two decades to produce the CPOSS209 data set of computational models of known and putative polymorphs linked to the current experimental data. The experimental collaborators in the Control and Prediction of the Organic Solid State (CPOSS) project, working alongside the development of the CSP methods, have provided much of the experimental data. The CSP studies evaluate the lattice energy directly by explicitly identifying the molecules within the crystal lattice and assuming that the charge distribution is the same in the crystal and isolated molecules. The lattice energy is the energetic cost of separating the infinite static lattice into infinitely separated static molecules in their most stable conformation, i.e.,:
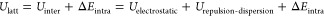
where the intermolecular contribution, *U*_inter_, is summed over all of the molecules in the crystal, and Δ*E*_intra_ is the energy penalty for changing the conformation of the molecules from those observed in the crystal to the lowest-energy conformation of the isolated molecule. The static lattice and energy, *U*_latt_, are fictional, and the relationship to measurable thermodynamic quantities is complex^23,24^ but it provides a useful zeroth-order model for CSP.^25,26^ If the molecule is assumed rigid in its most stable conformation, Δ*E*_intra_ = 0. In that case, an isolated molecule electronic structure calculation can estimate its structure and provide the molecular charge density, which is analyzed to provide an atomic multipolar description of the molecular charge density,^27^ which is used to calculate the electrostatic contribution to the lattice energy, *U*_electrostatic_. As the atomic multipoles describe the anisotropy of the lone pair and π electron density, the electrostatic term models hydrogen bonding and π–π stacking directionality, etc., giving a marked improvement over atomic charge models for CSP.^28^ All other intermolecular interactions are modeled by an empirical isotropic atom–atom potential, *U*_repulsion-dispersion_, parametrized to organic crystal data, which therefore has crudely absorbed some of the effects of the temperature-dependent motions within the crystals and the errors in modeling the intermolecular and intramolecular forces. For the CSP-generated structures used in this study, an exp-6 potential with the FIT parametrization^29^ has been used. However, this model has since been reparametrized to a much wider range of experimental data,^30^ and recently to DFT+D data with more sophisticated optimization techniques.^31^ This approach, which we will denote ψ_mol_ as it involves electronic structure calculations on the molecule, is readily extended to flexible organic molecules by recalculating the charge density and atomic multipoles at a range of conformations to give Δ*E*_intra_ and the conformation-dependent atomic multipoles. The conformational variations are defined by choosing the torsion angles (and sometimes bond angles) that are likely to change in response to the packing forces within the crystal from the gas phase optimized structures. It is common for a change in the torsions defining the position of a polar proton to improve the hydrogen bonding geometry and lower the overall lattice energy *U*_latt_. The methodology for using this hybrid *ab initio*/empirical force-field approach has developed considerably^2^ over the period in which we have been using it to develop CSP as a complement to industrial polymorph screening.^32,33^ Thus, the baseline ψ_mol_ structures and lattice energies used in this study vary in the specific method used (Supporting information (SI) subsections of Section S2). However, they are all based on approximating the flexibility of the molecule within the crystalline environment and absorbing the energetic effects of the change of molecular charge density and other errors into an average empirical atom–atom force field for *U*_repulsion-dispersion_.

These approximations are not necessary if periodic electronic structure calculations are performed on the organic crystals, the ψ_crys_ approach. This was pioneered in the fourth blind test^34,35^ where Avant-garde Materials Simulation optimized the crystal structures with a periodic plane wave PBE functional^36^ plus a dispersion correction whose damping was fitted to organic crystal structures.^37^ Dispersion-corrected periodic density functional theory (denoted ψ_crys_ in this work, also known as pDFT+D) has become very popular, using programs such as CASTEP,^38^ VASP,^39^ and FHI-aims.^40^ It can be used to validate crystal structures, particularly to adjust for systematic and other errors in the location of protons by X-ray crystallography.^41^ The academic Tkatchenko-Scheffler (TS) dispersion correction^42^ has become popular because of the quality of the reproduction of crystal structures.^43^ Thus, ψ_crys_ (PBE+TS) has become the workhorse method for calculating relative lattice energies of organic polymorphs, as it is relatively affordable with modern supercomputers, even though its application to large sets (10^2^–10^3^) of crystal structures in a CSP study can be prohibitively expensive. In addition, ψ_crys_ codes scale poorly with the size of the unit cell, and many well-established codes were initially developed for inorganic solids, where the forces are stronger and the unit cells are often of higher symmetry. The success of current ψ_crys_ and ψ_mol_ in CSP studies is often due to the cancellation of errors in relative lattice and free energies. The most recent seventh blind test^44,45^ illustrated the computational costs of CSP, with the successful commercial CSP companies using more sophisticated energy corrections to their ψ_crys_ optimized structures to correct for known deficiencies with the PBE or optPBE functional.^13,46^ In this study, we contrast the original ψ_mol_ CSP structures and energies with those reoptimized with ψ_crys_(PBE+TS), see the effect of a better dispersion model, many body dispersion (MBD),^47^ as a baseline for using our test data set for more accurate calculations, and demonstrate its use for computationally cheaper methods of modeling small organic molecules.

The availability of experimental data often limits our ability to assess the accuracy of computational predictions. Even when high-quality structural and thermodynamic data are available, it includes the effects of temperature-dependent thermal motions. Indeed, polymorphism often occurs because the crystals are kinetically hindered from transforming to the most stable form. Therefore, it is valuable to calculate whether a thermodynamic transition exists and what its associated temperature and enthalpy are. Comparison with experiments is also limited by there being relatively few polymorphs that have been structurally and thermodynamically characterized and difficulties in making measurements on phase-pure materials. Hence, this study includes a range of highly polymorphic systems where there has been some degree of screening or, at least, experimental studies in multiple laboratories. We also included more structures from our database of CSPs to increase the number and diversity of structures for any given molecule. For families of molecules, we include CSP-generated structures that are isostructural with the known crystal structures of related molecules.

In addition to examining the ability to reproduce experimentally observed structures and the sensitivity of relative lattice energies to the method, we also examine the variations in absolute lattice energy with a change in the method. A poor absolute lattice energy suggests that success in relative lattice energies results from a cancellation of errors, which thus indicates that methods cannot be relied upon for differences between very different crystal structures, such as a multicomponent and neat form of the same molecule. An imbalance in the accuracy of modeling of the inter- and intramolecular interactions may be more evident in the absolute lattice energies than the relative lattice energies. The absolute lattice energy is also a key component in the calculation of the absolute solubility from a thermodynamic cycle.^48^ The closest experimental measurement to the lattice energy is the heat of sublimation, where the temperature correction may be of the same order of magnitude as experimental error.^31^

This study aims to illustrate many of the issues confronting the adaptation of methods for calculating intermolecular forces to model the organic solid state, in which minor adjustments of the conformation of the molecule can make a significant difference to the lattice energy, by providing a data set of starting structures for a range of molecules. A couple of well-established methods are contrasted to see how they perform over an unusually wide range of crystal structures of diverse small organic molecules, most of which have been subjected to at least some experimental polymorph screening. The aim is to illustrate the requirements for modeling that arise from the diversity of polymorphs generated by experimental and *in silico* polymorph screening to assist the development of methods. It is not seeking to propose a recommended protocol for carrying out calculations for a specific code and level of theory and establishing its accuracy, nor provide a data set appropriate for meaningful statistical analysis or machine learning, but, unusually, to link the data to experimental studies. The utility of this data set is illustrated by using it to test the applicability of two recent machine-learned foundation model force fields. It is hoped that this data set will provide an initial test suite for proposed new methods of both more accurate and/or more cost-effective methods of evaluating the relative thermodynamic stability of polymorphs and for tackling the “over-prediction” problem by seeking models to distinguish which low-energy structures can be observed.

## 2 Method

### 2.1 Choice of Molecules and Crystal Structures

**Figure 1:**
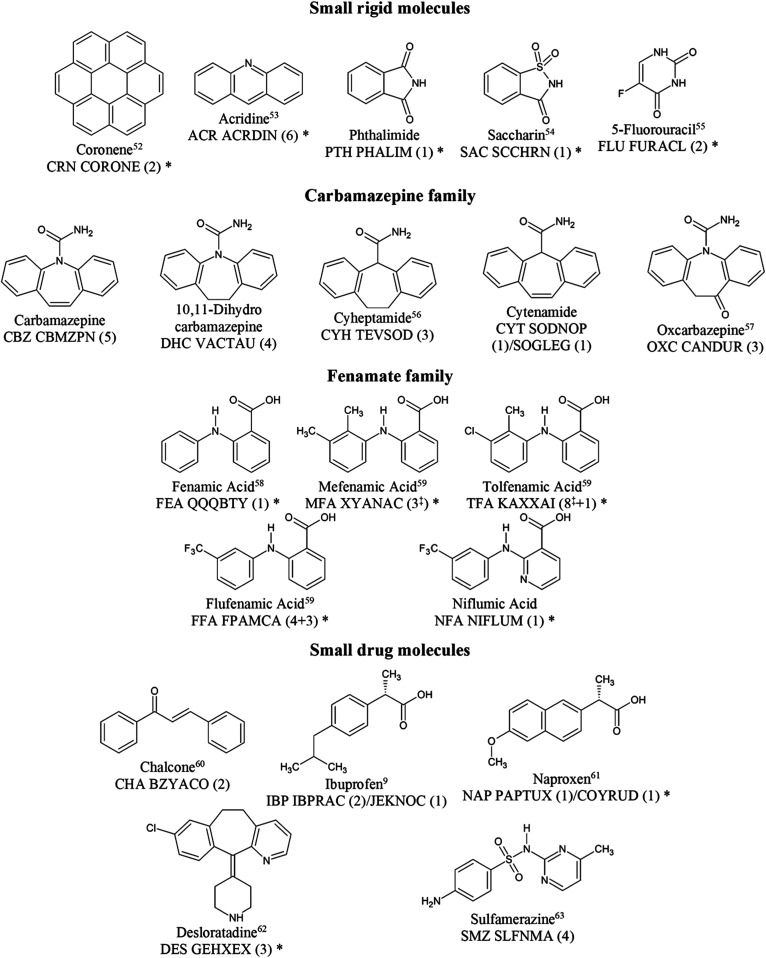
Molecular diagrams of the 20 molecules studied in this work, with their common names, reference to the previous CSP study^52−63^ used in this work, the three-letter abbreviation which is used with a number to identify the structures in the data set, CSD Refcode stems, and number of experimental polymorphs included in this work in parentheses (for TFA and FFA, one or three additional polymorphs, respectively, are included with the ψ_mol_ method that could not be included with the ψ_crys_ method; SI Section S2.3.3). ^‡^ denotes that a disordered structure is included, and the calculations are applied to the two ordered components. * denotes heats of sublimation are available in SITable S20. Where two refcodes are given, in the case of cytenamide, this is due to residual solvent in SOGLEG being reflected in a different refcode, and in the cases of ibuprofen and naproxen, the racemic form is first, and the enantiopure form is second.

The set of crystal structures chosen for each molecule in Figure 1 included most experimentally determined polymorphs and further hypothetical structures generated by the referenced CSP (SI Section S2). These were selected to include low-energy structures, particularly those with different hydrogen bonding or conformations from the experimental structures, structures that are isostructural to those of closely related molecules, and the lowest-energy structure in a Sohncke space group (i.e., where the symmetry elements are only translations, rotations, and roto translations). These are chiral crystal structures when the molecules are chiral, although only a subset of the Sohncke space groups are chiral space groups.^49^ The word isostructural perhaps needs a more careful definition;^50^ rather than consider space group and cell parameters, we base it on the ability to overlay a 20-molecule cluster ignoring molecular differences, and quantify using the minimum root-mean-square difference in the atomic positions in the cluster of 20 molecules (RMSD_20_ structural similarity),^51^ ignoring hydrogen atoms. In this work, we have referred to the molecules by their 3-letter abbreviations (Figure 1), the computationally optimized crystal structures by the abbreviation with Arabic numerals (SI Section S2), and the crystal structures of the experimentally observed polymorphs by their abbreviation and Roman numerals or Greek letters as conventional in the literature on the specific system. More details on the structures (both experimental and hypothetical) and why each was chosen are included in the tables of SI Section S2.

### 2.2 Experimental Data

We follow the basic assumption of CSP methods that the lowest-energy crystal structure should correspond to the polymorph that is most stable at low temperatures. For each molecule, we have outlined in subsections of SI Section S2 the evidence for which polymorph is the most stable at low temperature from the current literature. We have also tabulated in the SI the method of crystallization reported in the reference on the CSD (SI Tables S15–S18 in Section S3.1) and crudely assigned these: “type 1” = solution crystallization; “type 2” = solution crystallization with additives or other modifications; and “type 3” = crystallization not from solution, including heating and sublimation onto a template.

In order to assess the absolute lattice energies, we have tabulated the limited measurements of standard heats (or enthalpies) of sublimation (SI Table S20), mainly taken from the database of Chickos and Gavezzotti,^64^ and the compilations by Perlovich,^65−67^ with some additions from the NIST webbook.^68^ These are not the best type of reference sublimation enthalpies for evaluating the accuracy of theoretical calculations, as the enthalpies of sublimation are not linked to a specific polymorph, let alone a crystal structure at the same temperature, and the heat capacity corrections used are empirical (SI Section S3.2); however, there is no overlap of our molecules with those with better sublimation enthalpy data.^69,70^ As eight of the 20 molecules do not have any reported heat of sublimation, we also test an estimated heat of sublimation based solely on the number of occurrences of specific functional groups in the molecule^71^ (SI Section S3.2.2 and Table S20). We have largely followed the approach of Chickos and Gavezzotti,^64^ which describes in detail the uncertainties in comparing *U*_latt_ with −Δ*H*_sub_; they used the 679 heats of sublimation which could be matched to a crystal structure (without consideration of polymorph) from their critical compilation of 1655 sublimation enthalpies to test the AA-CLP atom–atom potential^72^ and the 500 smaller compounds to test the PIXEL-CLP^73^ method which is based on the molecular charge density.

### 2.3 Computational Methods

Using the structures generated in a CSP search as starting point structures has the advantage of providing a computationally consistent set where the hydrogen atom positions have been corrected for the systematic error in X-ray determinations, and all bond lengths, bond angles, and torsion angles have been determined by a molecular *ab initio* method. In addition, these crystal structures have been checked to be true minima, at least on the atomistic ψ_mol_ potential energy surface. It also allows a comparison of the energies and structures from a relatively cheap energy model that can be used as a starting point for further energy refinement in the CSP.

CASTEP^38^ was used as the periodic electronic structure crystal structure optimization code. The PBE functional^36^ was used as the canonical and favored GGA. A variety of dispersion corrections are available, and we chose to optimize the unit cells with the Tkatchenko Scheffler (TS) dispersion correction (denoted ψ_crys_(PBE+TS)),^42^ and then evaluate the energy of this optimized structure with the many body dispersion (MBD) correction^47^ (denoted ψ_crys_(PBE+MBD)) to show sensitivity to dispersion correction.

A key requirement of modeling the organic solid state is to minimize the errors introduced from the different size and shape of unit cells of polymorphs to a level where the calculations are worth doing. The ability to do this in a resource-efficient manner depends on many factors that can vary with code implementation, even for nominally the same type of theory, and so this methodology used within the plane wave code CASTEP^38^ needs adapting for other codes. The unit cell size is often a major consideration when estimating required computing resources. As the majority of organic crystals are in either a monoclinic or triclinic space group (34% of structures in the Cambridge Structural Database are *P*2_1_/*c* and 25% *P*1̅^74^), the standard choice of unit cells is preferably used, with angles as close to 90° as possible, as the Monkhorst–Pack algorithm^75^ for selecting the *k*-point grid is most efficient for rectilinear cells. The CASTEP optimizations were done within the space group constraints, using the default optimization method, LBFGS, a low memory modification of Broyden–Fletcher–Goldfarb–Shanno algorithm. Phonon calculations are not performed on the ψ_crys_ optimized structures, and so rely on the testing of the ψ_mol_ starting structures for the expectation that the converged structure is a true minimum rather than a saddle point between two lower-energy structures.

We have used on-the-fly generated ultrasoft pseudopotentials and frozen core approximation to allow a smaller plane wave basis set in the ψ_*crys*_ calculations. The size of the basis set is determined by using plane waves up to a cutoff in their kinetic energy, *E*_cut_. The number of plane waves required to reach this limit is proportional to the volume of the unit cell, which is one reason the cost of the calculation scales poorly with size. The key factor in determining the size of the basis set is the elements involved: although many organic systems are composed of just C, H, N, and O atoms, other elements such as F, Cl, I, S, and P are not uncommon for neutral molecules, and organic salts will require K, Na, etc. The first row p-block elements require the highest cutoff energies, increasing in order C, N, O, and F, making organic crystals more computationally demanding than inorganic ones. The elements involved in drug-like molecules are changing,^76^ and molecular functional material, such as explosives, often involve other functional groups. A second parameter is the sampling of the Brillouin zone in evaluating the energy, as represented by the *k*-point spacing, *k*_sp_. In the limit that the crystal was just a superposition of molecular charge densities (the assumption behind the ψ_mol_ method) and so a perfect insulator with no variation in the filled electron bands across the Brillouin zone, then the nonbonded energy would be insensitive to the *k*-point sampling. However, the interactions of the overlapping charge densities and the polarization of the charge distribution within the crystals, for example, in hydrogen bonding, mean that the nonbonded energy can be very dependent on the *k*-point grid, particularly in the directions of reciprocal space associated with charge delocalization. Hence, in principle, the number of plane waves (which increases with *E*_cut_) should be increased until the cancellation of errors has converged on the relative energy. As the shape and size of the unit cells usually differ for organic polymorphs, the k-point grid does not benefit from cancellation of errors, so it should be sufficiently fine that further changes do not result in a significant change in the calculated energy. Unfortunately, organic polymorphic energy differences are small, of the order of kJ mol^–1^, with an estimate based on ψ_crys_ (PBE+D2) calculations on known polymorphs^77^ showing that 40 to 50% of polymorphs differ in lattice energy by less than 2 kJ mol^–1^ and ∼90% by less than 6 kJ mol^–1^. (In contrast, inorganic electronic structure calculations are usually presented in eV, as the energies involved are 2 orders of magnitude larger than 1 kJ mol^–1^ ∼ 0.01 eV.) Hence, achieving convergence is very resource-intensive. Alternatively, the choice of these parameters should be determined by ensuring that the energy difference between polymorphs is insensitive to further improvement in these parameters. This will be very dependent on the difference in the size and shape of the unit cells chosen for this testing as well as the types of atoms and intermolecular interactions in the system. It is not practical to do comprehensive convergence testing on all pairs of polymorphs, particularly when the largest unit cell considered may well be stretching the available computational resources. The parameter testing is described in subsections of SI Section S2 for each molecule, and the results are summarized in SI Table S1. Typically, the *k-*point grid was determined to provide a maximum spacing of 0.1 Å^–1^ and a basis set cutoff between 700 and 1100 eV was applied.

We have converged the electronic energy to 10^–10^ eV and the forces in the cell optimization to 0.001 eV Å^–1^ (apart from five *R*3̅ structures of the carbamazepine family where forces were only converged to 0.01 eV Å^–1^), but we note that some molecular crystals have extremely shallow energy wells; tighter convergence would be needed for properties such as phonons.

### 2.4 Isolated Molecule Calculation

In order to calculate the absolute lattice energy from ψ_crys_ crystal structure optimizations, it is necessary to calculate the energy of the optimized isolated molecule *U*_mol_ by the same method. Then, , where *U*_total_ is the energy of the unit cell containing *Z* molecules. The starting points were the gas-phase-optimized molecules (SI Section S4). A cuboid cell was constructed around each conformer, with the longest edge, *l*_long_, parallel and equal to the longest atom–atom distance in the molecule, the medium edge, *l*_mid_, along the longest atom–atom distance of the projection of the atoms onto a plane normal to *l*_long_, and the shortest distance, *l*_short_, in the direction defined by orthogonality such that the box approximates the smallest cuboid that contains the whole molecule. All box lengths were increased by 1 Å, and then, single-point energies were evaluated in 1 Å steps in all three dimensions, until the point where three consecutive steps led to the energy changing by less than 0.1 kJ mol^–1^. The molecule was then optimized in this fixed cell with ψ_crys_(PBE+TS), and a single point energy of the final conformation was also evaluated with ψ_*crys*_(PBE+MBD). SI Table S1 contains the minimum intermolecular atom–atom distances of this cell and its volume. This box size depends on the molecule, with there being a very crude correlation with the molecular dipole moment, reflecting that the range of the intermolecular forces determines the intermolecular gap needed for negligible interaction between the “isolated” molecules (SI Section S1.1, Table S2, and Figure S1). For all systems considered, the plausible conformational minima for an isolated molecule were calculated by these methods (SI Section S4).

### 2.5 Illustrative Use of CPOSS209 Data Set

The 209 ψ_crys_(PBE+TS) optimized crystal structures in the data set (provided in SI as All_Psi_Crys.cif) were reoptimized using the ASE 3.22.1 Python library optimizer,^78^ and two general-purpose machine-learned foundation models developed using the MACE architecture^79^ using version 0.3.5 of the MACE Python library. The first foundation model is MACE-OFF23,^80^ which was trained on organic molecule and dimer calculations with the ωB97M-D3(BJ)/def2-TZVPPD method. To the best of our knowledge, this is the first application of this model to crystal structures. The second foundation model is MACE-MP-0,^81^ which was based on approximately 1.5 M configurations derived from approximately 150 k unique members of the Materials Project database of mainly small periodic unit cells (90% under 70 atoms), describing inorganic crystals with some molecular components. The training data set DFT calculations used the PBE exchange–correlation functional (with Hubbard U terms applied to some transition metal oxide and fluoride systems) but with no additional dispersion correction. We found that this resulted in unrealistically high (although still negative) lattice energies, with two structures of DES becoming unbound for certain parameter sets (i.e., positive lattice energies, see SI Section S6). Hence, the D3 dispersion energy correction, as implemented in ASE 3.22.1,^78^ which uses the parameters of the D3(BJ), i.e., with a Becke-Johnson damping function,^82^ was applied to the optimizations following the same procedure as the original MACE-MP-0 testing.^81^ Optimizations were performed with “small,” “medium,” and “large” parameter sets, which are distinct for the two models and provide a compromise between speed of evaluation and model quality.^80,81^

Initial coordinates of the atoms in the crystal structures were obtained by replicating the atoms in the .cif file according to the symmetries in the unit cell and then converting the internal coordinates (**s**_ij_) to Cartesian coordinates (**r**_ij_) as **r**_ij_ = **hs**_ij_, where **h** is the box matrix. Energy minimizations were then performed using the LBFGS algorithm as implemented in ASE until the maximum force between atoms was lower than 10^–5^ eV Å^–1^, using periodic boundary conditions in all directions.

The calculation of the absolute lattice energy, *U*_latt_, required the number of molecules in the unit cell, which, for this specific data set, could be done by defining a molecule to comprise all atoms with any interatomic distance of less than 1.8 Å, or less than 1.3 Å if either atom was hydrogen. The isolated molecule energies, *U*_mol_, were calculated using the .mol files (provided as All_Psi_Mol.mol and All_Psi_Crys.mol in the SI) in a large box, turning off periodic boundary conditions, optimizing with the force field and then taking the lowest-energy conformation for the evaluation of *U*_latt_, which could be different for the different foundation models and/or parameter sets (SI Section S6).

## 3 Results

### 3.1 Choice of Molecules, Crystal Structures, and Relative Lattice Energies

The lattice energies relative to the most stable low-temperature experimentally observed polymorph are reported and separated into the four groups of five molecules defined in Figure 1.

#### 3.1.1 Small Rigid Molecules

The early development of CSP started with rigid molecules, and the ψ_mol_ method is most effective when the molecules are rigid, so that only one quantum mechanical calculation is required to determine the molecular structure and its charge distribution, and hence the model for the intermolecular electrostatic interactions. This approach works best when using distributed multipoles.^28^ A recent study performed a thousand CSP studies of rigid organic (containing only C, H, N, and O) molecules^83^ using a very similar ψ_mol_ approach, and found 74% of observed crystal structures are found within 2 kJ mol^–1^ of the global lattice energy minimum and over 41% at the global minimum. In our small sample of very rigid molecules, the ability of the ψ_crys_(PBE+TS) optimization to allow the molecular structure to change in response to the packing forces resulted in very little change (RMSD_1_ ≤ 0.01 Å even when hydrogen atom positions are included).

The first crystal structure of the β form of coronene (CRN), named after the type of packing, was found by crystallization at ambient temperature in a magnetic field.^84^ This structure was later used to identify the β form as the low-temperature form and confirm that the γ form is the most stable at ambient conditions.^85^ All calculations give β as the most stable form. However, the energy difference is unrealistically large when the TS dispersion correction is included. Although dispersion is dominant for the packing of these systems, the electrostatic model is also crucial in modeling the stacking.

Acridine (ACR) is a prime example that conformational flexibility is not necessary for extensive polymorphism; the crystal forms numbering suggests that ACR has nine polymorphs. However, form I is a hydrate and forms V and VIII do not have full structure determinations (SI Section S2.1.2.2), leaving six fully characterized experimental structures of this dipolar molecule. Both ψ_mol_ and ψ_crys_(PBE+MBD) give forms II and IX as the most stable and very close in energy and the highly metastable form IV as the least stable, in agreement with experiment. Form IV crystallizes in a polar space group, with a net dipole in the unit cell. Hence, in principle, there is a morphology-dependent correction to the lattice energy,^86^ but we have followed the usual assumption that the surface dipole will be canceled out by the environment during crystallization^87^ and so ignored this term which would have further destabilized it.

Phthalimide (PTH) was included because of the unusual hydrogen bonding properties of the imide group, found to be so weak in the near-spherical 3-aza-bicyclo(3.3.1)nonane-2,4-dione (CSD REFCODE: BOQQUT), that it has a plastic crystal at high temperatures.^88^ There is only one known polymorph of PTH, and no more were found in some screening work by collaborators after we had performed this early unpublished CSP. The ψ_crys_ results have switched the energy ordering and so give the experimental structure as the most stable, although some hypothetical structures remain within the energy range of plausible polymorphism. However, PTH could well be monomorphic if there were low barriers to transformation between the hypothetical and known experimental structures. This is the case for 3-Aza-bicyclo(3.3.1)nonane-2,4-dione, which has remarkably low barriers to changing hydrogen bonding between catemeric and dimer hydrogen bonding,^88^ a counterexample to the assumption that the barriers to changing hydrogen bonding are sufficient to give rise to long-lived polymorphs when the hydrogen bonding motif differs.

**Figure 2:**
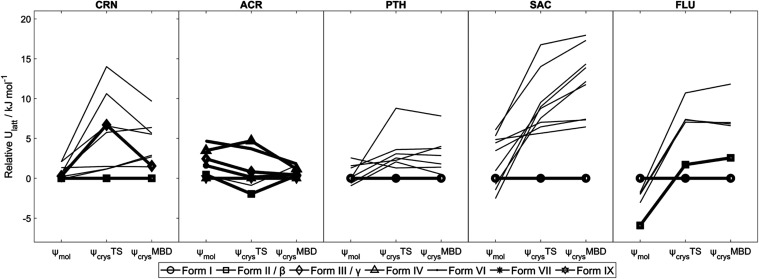
Relative energies of various crystal structures of coronene, acridine, phthalimide, saccharin, and 5-fluorouracil, with different computational models. Energies are calculated relative to that of the form believed to be most stable experimentally at low temperature (SI subsections of Section S2.1). Structures with symbols joined by bold lines are experimentally observed forms, with the form names in the legend. ψ_mol_ denotes lattice energy minimization corresponding to the original CSP; ψ_crys_TS denotes lattice energy minimization with ψ_crys_(PBE+TS); ψ_crys_MBD denotes single-point energy calculation of the ψ_crys_(PBE+TS) structure with ψ_crys_(PBE+MBD).

Saccharine (SAC) is a heavily studied compound that appears to be monomorphic. Figure 2 clearly shows that it is not worth doing the polymorph screening suggested by the published ψ_mol_ CSP,^54^ as all of the structures that appeared to be more stable than the known form are implausibly metastable when calculated with ψ_crys_. This illustrates the value of confirming the predicted relative stability of CSP-generated structures with the best feasible methods before embarking on an expensive polymorph screen.

Fluorouracil (FLU) was predicted by an early ψ_mol_ CSP to have a polymorph more stable than the *Z*′ = 4 structure of FLU I, and this was subsequently found as FLU II.^55^ The formation of FLU II requires the exclusion of water, as it could only be reproduced with fresh, dry nitromethane solvent,^89^ although it has since been shown that FLU II can be reliably obtained from methanol containing nicotinamide, presumably as an alternative inhibitor of FLU I crystallization.^90^ It is worth noting that the PTH and FLU searches were performed prior to ψ_crys_ being possible in the period when rigid-molecule ψ_mol_ CSPs were being evaluated against commercial programs using traditional force fields. The ψ_crys_ calculations give the correct prediction that FLU I is more stable than FLU II and show that the alternative uracil hydrogen bonding motifs^91^ are unlikely to be found in additional polymorphs.

#### 3.1.2 Carbamazepine Family

**Figure 3:**
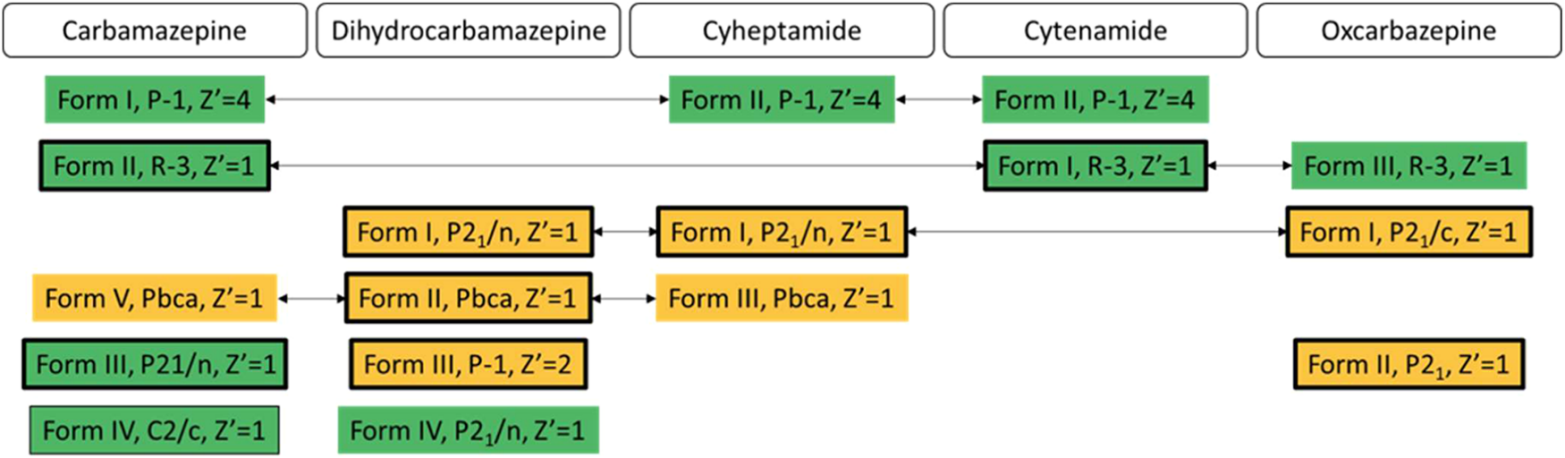
Isostructural relationships between the observed polymorphs of carbamazepine and its analogues. Structures in green have *R*_2_^2^(8) hydrogen-bonded amide dimers, whereas those in orange have *C*_1_^1^(4) hydrogen-bonded amide chains. Boxes with a thick black outline denote forms crystallized from a routine crystallization from solvent, boxes with a thin black outline denote forms crystallized from solvent but with an additive or external modification, and boxes with no outline denote forms produced by solvent-free methods, such as heating, sublimation, or polymer templating. See SI Section S3.1 for the crystallization conditions of all polymorphs.

The antiepileptic generic drug carbamazepine (CBZ) is an iconic system for polymorphism studies,^92^ used in the development of CSP and experimental screening methods. Early CSP studies^93,94^ persistently predicted that structures with a catemeric *C*_1_^1^(4) hydrogen bonding motif were thermodynamically competitive with the observed polymorphs containing the amide *R*_2_^2^(8) dimer. (The hydrogen bonding graph set notation *G*_donors_^acceptors^ (number of atoms in pattern) denotes the pattern of hydrogen bonding as a combination of chains (*G* = *C*), rings (*G* = *R*), intramolecular (*G* = *S*), or other finite (*G* = *D*) patterns,^95^ and is often a useful way of distinguishing between polymorphs with different hydrogen bonding.) This led to investigations of the polymorphism of closely related compounds, namely, dihydrocarbamazepine (DHC), cyheptamide (CYH), cytenamide (CYT), and oxcarbazepine (OXC), and subsequently the use of certain crystal structures as templates for the crystallization of new polymorphs of another molecule in the family.^56^ This means that there are a significant number of isostructural polymorphs within this family (Figure 3) and a number of polymorphs that have not been obtained by crystallization from solution. The polymorphs vary considerably in unit cell size, shape, and symmetry, and the *Z* = 18, *R*3̅ structures were challenging to optimize, and the ψ_crys_(PBE+MBD) single point energies could not be calculated.

**Figure 4:**
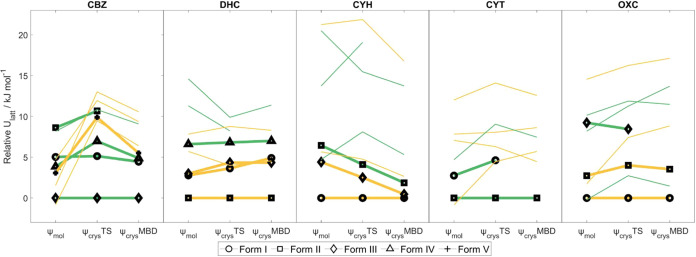
Relative lattice energies of carbamazepine and its analogues dihydrocarbamazepine, cyheptamide, cytenamide, and oxcarbazepine. Structures in green have *R*_2_^2^(8) hydrogen-bonded amide dimers, whereas those in orange have *C*_1_^1^(4) hydrogen-bonded amide chains. Energies are calculated relative to that of the form believed to be most stable experimentally at low temperature (SI Section S2.2.4). Structures with symbols joined by bold lines are experimentally observed forms, with the form names in the legend. ψ_mol_ denotes lattice energy minimization corresponding to the original CSP; ψ_crys_TS denotes lattice energy minimization with ψ_crys_(PBE+TS); ψ_crys_MBD denotes single-point energy calculation of the ψ_crys_(PBE+TS) structure with ψ_crys_(PBE+MBD).

The CSD structure CBMZPN03, called CBZ II, is an *R*3̅ structure that is now considered to usually be a solvate with small amounts of various disordered solvents in its hydrophobic channel.^92^ Despite solvent occlusion being ruled out by thermogravimetric studies in the original structure determination,^96^ the relatively high lattice energy of CBZ II in early CSP studies led to the identification of toluene and *n*-tridecane in the channels of CBZ II by TGA, DSC, solution ^1^H NMR and hot stage microscopy^97^ and the anomalies in the Hirschfeld surface led to another group refining single crystal diffraction data as a nonstoichiometric 1:0.1 THF solvate.^98^ Thus, CBZ II illustrates how solvents can stabilize the crystallization of structures that would otherwise be highly metastable, an observation that has been exploited in the CSP-based design of highly porous organic crystals.^99,100^ The isomorphous OXC III forms crystal aggregates with a twisted unicorn-horn morphology^101^ particularly when grown by sublimation. The *R*3̅ structure of CYT I is known to have uncharacterized solvent in the channel,^102^ but is not highly metastable (Figure 4) in contrast with the *R*3̅ CBZ II and OXC III channel structures, which are about 10 kJ mol^–1^ above the most stable structure. This illustrates how desolvated solvate polymorphs may be higher in energy than others, for example, in galunisertib,^32^ and how structures with void space channels need careful consideration. Indeed, finding porous polymorphs, like the δ polymorph of trimesic acid, which has a guest-free hexagonal pore structure, is particularly challenging for CSP.^103^ Of the other high lattice-energy observed polymorphs, DHC IV was obtained from the vapor,^104^ CBZ V by sublimation onto a related template crystal from this family,^105^ CBZ IV in the presence of polymer,^106^ and CBZ I formed by heating. Although the ψ_mol_ method has hypothetical structures competitive in energy with the observed polymorphs, the ψ_crys_(PBE+MBD) model mainly has all experimental structures more stable than the hypothetical structures, with the exception of a hypothetical *R*_2_^2^(8) hydrogen-bonded structure of OXC, which is the lowest-energy structure from the ψ_mol_ CSP study.^101^

#### 3.1.3 Fenamate Family

The fenamates are included as a family with tolfenamic acid (TFA) and flufenamic acid (FFA) being nonsteroidal anti-inflammatory drugs that have been used in many studies of polymorphism. The molecules are conformationally flexible and resemble ROY^107^ (5-methyl-2-[(2-nitrophenyl)amino]-3-thiophenecarbonitrile, which has many polymorphs which vary in color), in being two aromatic rings linked by an amine group. However, it is not this flexibility that gives rise to the polymorphism, as the scaffold, fenamic acid (FEA), is monomorphic.^58^ The family includes mefenamic acid (MFA), which is related to TFA by Cl/CH_3_ exchange, which is one of the most likely substitutions to give isostructural crystal structures.^108,109^ Nonetheless, in marked contrast to the carbamazepine family, the only isostructural polymorphs for the entire set of five molecules are TFA VI, which was templated by sublimation onto isomorphous MFA I,^59^ TFA IX^110^ isomorphous with FFA I, and the disordered structures of MFA II and TFA V (with different disorder ratios in the four determinations of these two structures; SI Table S9). FEA I and TFA VIII differ only by a twist of the phenyl ring in one of the independent molecules for FEA I. Overall, there are 9 distinct packings in the 17 observed crystal structures of the carbamazepine family and 14 in the 19 crystal structures of the fenamate family. Another contrast to the carbamazepine family is that the experimental crystal structures of the fenamates have the *R*_2_^2^(8) carboxylic acid hydrogen bonding motif across an inversion center as the only intermolecular hydrogen bonding. Thus, the majority of the fenamate crystal structures in our study are based on substituting the hydrogen-bonded dimer of one molecule into the observed crystal structures of another (SI Figure S3), which usually differ in the angles between the substituted phenyl rings and the central, approximately planar, benzoic acid dimer and in the packing of these dimers. The exceptions are the lowest-energy chiral structures, which cannot contain this dimer, and all had *C*_1_^1^(4) hydrogen bonds along a screw axis.

The erstwhile record-breaking number of structurally characterized polymorphs of FFA comes from screening in the presence of polymers as heteronuclei, which accessed eight polymorphs, with a further one (FFA VI) being found from a solid–solid low-temperature transformation.^111^ Of these, four structures had large unit cells of 6 to 16 molecules and more than one independent molecular conformation (*Z*′), and so could not be optimized by plane wave PBE+TS with reasonable computational resources. The ψ_mol_ energies for an ordered model for FFA IV (*Z*′ = 3), FFA V (*Z*′ = 4) and FFA VI (*Z*′ = 6) were 2.1, 3.3, and 3.1 kJ mol^–1^ above the global minimum, i.e., of comparable stability with the other polymorphs (SI Table S25). This was also the case for TFA IV (*Z*′ = 3), with an ψ_mol_ energy only 0.5 kJ mol^–1^ above the global minimum (SI Table S25). FFA VIII has *Z*′ = 9.5, and one independent molecule was so disordered that it does not have atomic coordinates and so could not be modeled at all. The reported disorder in MFA II and TFA V was included as two ordered structures, each modeling the separate disorder components (namely, MFA02/MFA03 and TFA04/TFA05 in SI Table S9).

**Figure 5:**
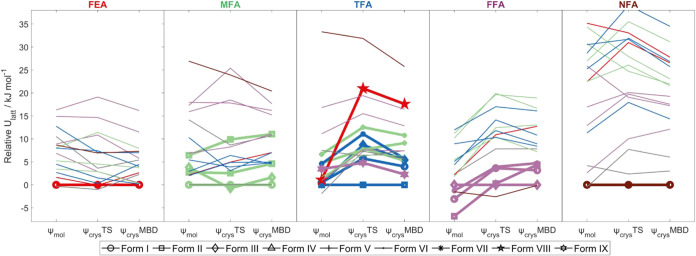
Relative energies of various crystal structures of fenamic acid, mefenamic acid, tolfenamic acid, flufenamic acid, and niflumic acid, with different computational models. The lines are colored according to the molecule which exhibits the packing in an experimental crystal structure (see title colors; TFA has packings in common with many other molecules). Note that the structures that could not be optimized by the ψ_crys_ approach, TFA IV, FFA IV, FFA V, and FFA VI are not included, but their ψ_mol_ structures are included in a separate .cif file and their energies are in SI Table S25. Energies are calculated relative to that of the form believed to be most stable experimentally at low temperature (SI Section S2.3.4). Structures with symbols joined by bold lines are experimentally observed forms, with the form names in the legend. ψ_mol_ denotes lattice energy minimization corresponding to the original CSP; ψ_crys_TS denotes lattice energy minimization with ψ_crys_(PBE+TS); ψ_crys_MBD denotes single-point energy calculation of the ψ_crys_(PBE+TS) structure with ψ_crys_(PBE+MBD).

The results in Figure 5 show that the experimentally observed structures are among the most stable, with TFA VIII (found by sublimation onto copper)^59^ and the highly metastable MFA II being more metastable by ψ_crys_ methods. There is some reranking by method, with the effect of changing the model for the dispersion correction being as significant as changing from ψ_mol_ to ψ_crys_. All methods agree that there are energetically competitive structures for FEA. Some of the most unstable hypothetical structures are those derived from niflumic acid (NFA), which is unsurprising, as substituting a nitrogen atom for a C–H means that the molecule prefers to be planar. In all other molecules, the steric clash of H atoms means that the two aromatic rings are not coplanar, and there is considerable variation in the conformation. Indeed, the CSP landscape generated for NFA contained only one structure that could be considered isostructural with an experimentally observed polymorph of one of the other molecules, and the four CSP-generated NFA structures included were always in the five lowest-energy crystal structures of NFA regardless of the energy model. A probable form II of NFA has been observed by a fast evaporation technique and characterized by DSC, TGA, IR, and PXRD, but the structure has not been solved.^112^

#### 3.1.4 Small Drug Molecules

These small molecules span an increasing range of functional groups and all have conformational flexibility that requires the balancing of the intramolecular energy penalty for changing at least some torsion angles and intermolecular forces.

The chalcone (CHA) molecule is a scaffold for many drugs, fluorescent probes, etc. Its CSP shows a large range of possible structures that are similar in energy. This is consistent with the fact that there are about 170 different types of packing in only 232 crystal structures of this molecule with small substituents.^60^ The sample of 14 structures in the CPOSS209 data set includes some where the molecule is approximately planar, as in the observed structures (and lowest-energy chiral structure), some where there is a marked twist to the shape, and two with a *cis* configuration about the central single bond. These last two structures are somewhat stabilized with ψ_crys_, although this conformation is only observed experimentally for molecules with bulky substituents *ortho* to the carbonyl group. There is considerable reordering of the relative stability with a change of method and dispersion model, as may be expected for a molecule that is mainly stabilized by the stacking of aromatic rings and CH···O= interactions as well as close packing. All methods have CHA I and the major (88%) component of CHA II among the most stable structures.

The pain relief drug ibuprofen (IBP) is a conformationally flexible, chiral molecule possessing two enantiomers. S-IBP is biologically active, while R-IBP needs to be transformed in the body to its S-counterpart. It is usually marketed in racemic form I because of the expense of manufacturing the enantiopure form.^113,114^ A second less-stable racemic form II, containing two different conformations,^115^ can be formed by quenching the melt. While racemic form I was the most stable structure in the CSP study,^9^ the ψ_crys_ calculations give smaller energy differences, although agreeing that racemic form II is highly metastable relative to hypothetical structures containing different conformations or alternative hydrogen bonding motifs. The difference is very sensitive to dispersion correction, consistent with the importance of packing of the isopropyl group.

The first crystal structure of the anti-inflammatory naproxen (NAP) was obtained by crystallizing the enantiopure molecule, which showed an unexpected bending of the naphthalene ring, which is reproduced by the calculations. The CSP was used to solve the structure of racemic crystals from powder data.^61^ The global minimum of the *Z*′ = 1 search was actually a transition point between lower-symmetry structures (lowering the symmetry from *Z*′ = 1 *Pbca* (NAP03) to *Z*′ = 2 *Pbc*2_1_ (NAP01)) although the energy difference was less than the estimated zero-point energy. This was consistent with the solid-state NMR showing that it was a *Z*′ = 1 structure and is an example of how dynamical averaging can mean that an observed structure has a higher symmetry than a lattice energy minimum. The full optimization with the ψ_crys_(PBE+TS) method leads to both close approximations of the racemic structure being the same *Z*′ = 1 structure and gives the observed chiral structure as the lowest-energy chiral structure.

The antihistamine desloratadine (DES) undergoes an unusual, reversible, two-step single crystal to single crystal transformation between three conformational polymorphs: low-temperature DES I, a polytypic intermediate DES II, and high-temperature DES III, involving a sequential flipping of the piperidine rings.^62^ All three polymorphs crystallize simultaneously during production.^116^ The original CSP had some less dense hydrogen-bonded structures that were more stable than the experimental forms, but ψ_crys_(PBE+MBD) has the experimental forms as marginally more stable. The isolated molecule calculations gave eight conformational minima within a small energy range, and a crystal structure with each conformation is in the data set. The low-energy molecular conformations of DSE tend to have the aromatic nitrogen fairly well shielded, and only DES06 uses it as a hydrogen bond acceptor. All other crystal structures selected (with the exception of the three experimentally observed ones which exhibit no hydrogen bonds) have a hydrogen bonding motif using the piperidine amine group as both donor and acceptor. The highly unusual *R*_2_^2^(4) “hydrogen bonding” motif observed in the least stable ψ_mol_ hypothetical structure, DES07, is even more unstable with ψ_crys_ and so an artifact of the ψ_mol_ model. The lack of hydrogen bonding in the experimental forms emphasizes how some low-energy molecular conformations may be incapable of close packing with themselves with favorable interactions. The sensitivity of the relative energy to the dispersion model underlines that, for flexible drug molecules, the inter- and intramolecular dispersion and ability to close pack can outweigh the traditional strong hydrogen bonding interactions.

**Figure 6:**
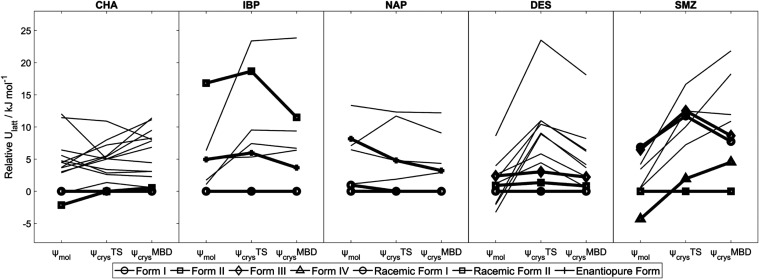
Relative lattice energies of various crystal structures of chalcone, ibuprofen, naproxen, desloratadine, and sulfamerazine. For racemic naproxen, the *Z*′ = 2 approximate model for the experimentally observed form, NAP03, is indistinguishable from that for NAP01 after ψ_crys_(PBE+TS) optimization. Energies are calculated relative to that of the form believed to be most stable experimentally at low temperature (SI subsections of Section S2.4). Structures with symbols joined by bold lines are experimentally observed forms, with the form names in the legend. ψ_mol_ denotes lattice energy minimization corresponding to the original CSP; ψ_crys_TS denotes lattice energy minimization with ψ_crys_(PBE+TS); ψ_crys_MBD denotes single-point energy calculation of the ψ_crys_(PBE+TS) structure with ψ_crys_(PBE+MBD).

The early antibiotic, sulfamerazine (SMZ), stands out in having a large lattice energy difference between high-temperature SMZ I and ambient stable SMZ II (Figure 6). The as-yet unreproduced^117^ SMZ III is also high in energy. The global minimum with the ψ_mol_ method has recently been published as SMZ IV, and this is the only metastable form according to ψ_crys_(PBE+MBD) within 6 kJ mol^–1^ of SMZ II, which is the lattice energy difference for 93% of observed polymorphic pairs which are not conformational polymorphs.^77^ This system is strongly affected by temperature, with SMZ II transforming to SMZ I above 420 K. Phonon calculations on SMZ I and SMZ II show that the thermal corrections are sufficiently large that ψ_crys_(PBE+TS) modeling correctly predicts that SMZ I and SMZ II are enantiotropically related.^63^ The hypothetical structures that are least stable with ψ_crys_(PBE+MBD) are the chiral structure (SMZ05), different layers (SMZ06 and SMZ07), and conformational polymorph with the methyl group on the other side (SMZ08). Thus, SMZ appears to challenge some of the current expectations of the lattice energy differences between polymorphs.

### 3.2 Relative Energies of Chiral and Racemic Structures

**Figure 7:**
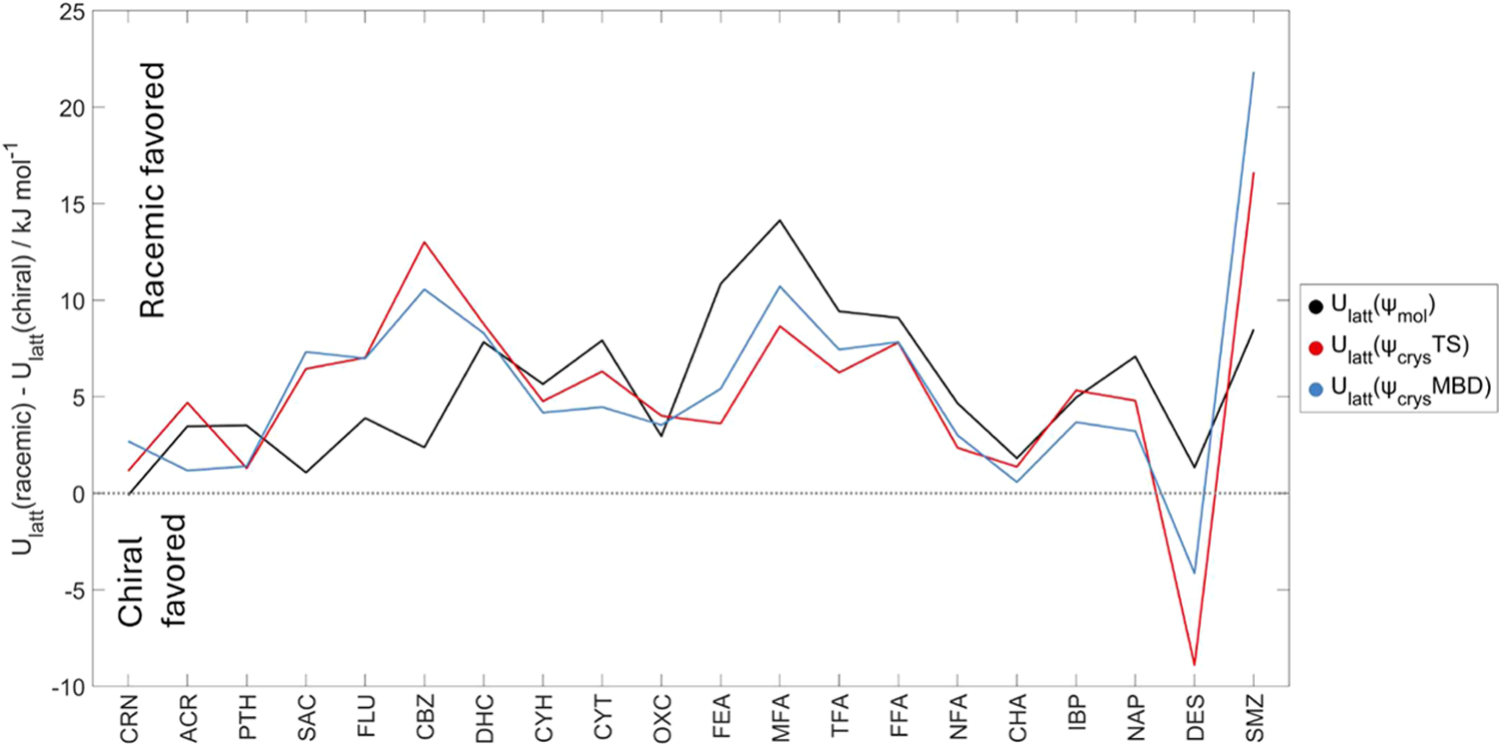
Lattice energy difference between lowest-energy crystal structure in a Sohncke space group and lowest-energy racemic crystal structure specific for each computational method for each of the 20 molecular systems, colored black for ψ_mol_, red for ψ_crys_(PBE+TS), and blue for ψ_crys_(PBE+MBD). Further details are available in SI Section S5.3.

Many drug molecules are intrinsically chiral, commonly having a chiral *sp*^3^ carbon atom that cannot racemize under the crystallization conditions. This is the case with IBP and NAP, which have the typical property (applying to approximately 90% of molecules^118^) that the racemic crystal structure is markedly more stable and therefore less soluble than the crystal structure of an enantiopure sample. This tendency is attributed to the possibility of inversion symmetry relating the two enantiomers of the molecule, allowing more effective packing of bumps into hollows and the formation of strong synthons like the *R*_2_^2^(8) carboxylic acid or amide hydrogen-bonded dimers. Unfortunately, for the ability to separate the enantiomers by crystallization, the enantiopure crystal structure is rarely significantly more stable than any racemic structure, as this requires a strong preference for a translation and/or rotation packing defining all three dimensions.^119^ This has recently been confirmed by an analysis of the CSP landscapes of 356 chiral rigid molecules, which showed significant thermodynamic stabilization in many of the 86% of cases where racemic crystallization was favored, and small lattice energy differences in the majority of cases where the enantiopure crystal is more stable.^83^ In addition to the enantiopure forms of IBP and NAP, OXC II and all polymorphs of DES have experimental structures in the Sohncke space groups as the molecules have a chiral conformation. Most of the molecules in the CPOSS209 data set (15 out of 20), apart from the rigid planar molecules, can adopt chiral conformations. To examine whether the difference between racemic and chiral crystal structures extends to chiral conformations, Figure 7 shows the energy difference between the lowest-energy structure in a Sohncke space group and the lowest-energy racemic crystal structure for all 20 molecules, and Figure S18 gives the relative energies of all of the structures, classified by whether the crystal structures of the chiral or achiral conformations adopt racemic or Sohncke space groups. The preference for a racemic structure is perhaps smaller for the near-planar molecules, CRN, ACR, PTH, SAC, FLU, NFA, and CHA (the latter two being practically planar in both the lowest-energy racemic structure and Sohncke structure), than for the molecules whose low-energy conformations are very three-dimensional. This is consistent with planar molecules having fewer constraints on the ability to close pack. However, the tendency is not marked and comparable within the uncertainty in the relative energy to computational models.

As most drugs have to be developed as enantiopure because of the difference in the biological effects of the enantiomers, the pure enantiomer can only crystallize in one of the 65 Sohncke space groups.^49^ Hence, CSP studies of chiral molecules are usually limited to these space groups. However, it should be noted that there are cases of solid solutions containing both enantiomers,^120^ some of which might be predicted by examining racemic structures that are lower in energy than chiral structures. An example of this is tazofelone, where an alternative conformation of one enantiomer can be substituted for the other enantiomer in a low-energy structure, giving rise to the solid solution.^121^ This emphasizes the role of the input material in the crystallization experiments. Some molecules are slow to reach an equilibrium conformational distribution, or could racemize, isomerize, or change tautomer in certain crystallization conditions,^122^ and so removing the memory of the input material in crystallization experiments requires careful consideration.^123^ The sample history can be important; it is hard to see why it was not possible to crystallize the active enantiomer of a melatonin agonist when the other enantiomer crystallized readily.^124^ Such possibilities should be considered in designing or interpreting CSP studies, which will preserve the input molecular structure and not consider any kinetic effects, including equilibration in solution.

### 3.3 Absolute Lattice Energies

**Figure 8:**
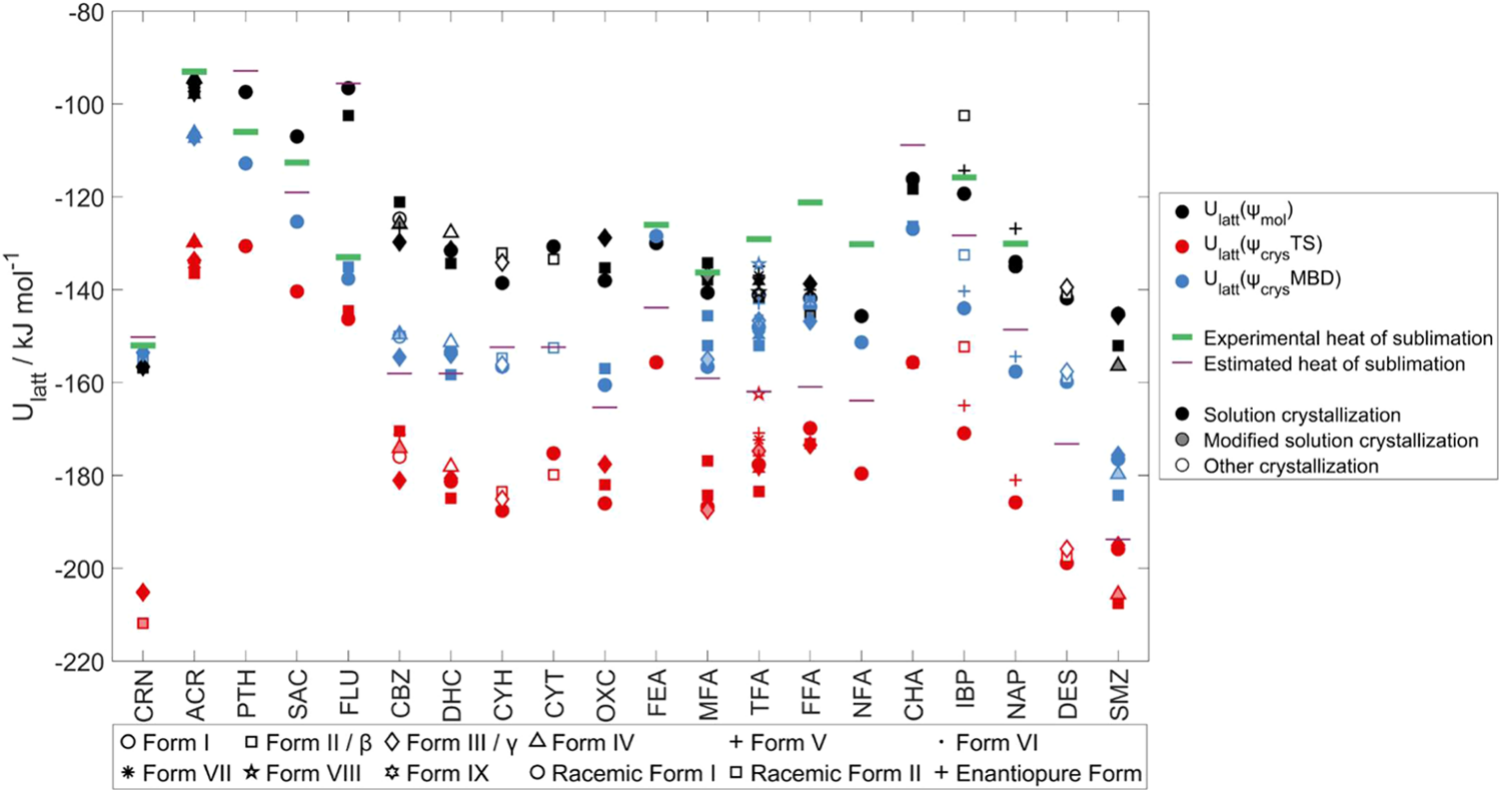
Calculated lattice energies for the experimentally observed crystal structures of the molecules studied in this work, colored black for ψ_mol_, red for ψ_crys_(PBE+TS), and blue for ψ_crys_(PBE+MBD). Filled symbols represent solution crystallization experiments; shaded symbols are modified solution crystallization experiments, and open symbols are crystallization experiments not from solution (SI Section S3.1). Thick green lines are experimental Δ*H*_sub_ (where available), and thin purple lines are Δ*H*_sub_ estimated from the atomic types.^71^

Figure 8 compares the three different lattice energies for the experimentally observed polymorphs against the negative of the heat of sublimation, −Δ*H*_sub_, where available (SI Table S20). There is also a molecule-based estimate of the heat of sublimation that depends only on the types of atoms in the molecule, calculated using the atomic contributions (SI Table S20) fitted by Ouvrard and Mitchell to experimental heats of sublimation.^71^ The type of crystallization experiment used to produce each polymorph is indicated by the shading. As the lattice energy scale is so large, there are molecule-specific plots and tabulated data in SI Section S4.

The differences between the methods of calculating the lattice energies are very significant, consistent with the poor correlation between the lattice energies calculated by the different models for the entire set of structures (SI Section S5 and Figure S17). This confirms that the similarity of the relative lattice energies (Section 1) arises from cancellation of errors. The ψ_crys_ lattice energies differ markedly for just changing the dispersion correction from TS to the more theoretically sound MBD model, with the latter being much closer to experiment. These results are consistent with studies of other ψ_crys_(PBE+D) lattice energies for molecular crystals.^125^ The difference made by changing the dispersion correction is heavily dependent on the types of intermolecular forces that occur between the different molecules, being small for FLU and particularly large for CRN. The ψ_mol_ method uses empirical exp-6 parameters that have been fitted to a very limited range of heats of sublimation (with an incorrect sublimation energy for C_6_F_6_ being used for the F parametrization^126^), but this may explain their relative success for absolute heats of sublimation.^48^ The experimental heats of sublimation and the empirical estimates of Δ*H*_sub_ based on the additivity of atomic contributions (defined by atomic number, hybridization state and bonding environment) fitted to experimental Δ*H*_sub_ values two decades ago^71^ (Figure 8, SI Table S20) agree well for CRN and ACR but are in error by around 30 kJ mol^–1^ for some of the more flexible molecules. The empirical estimate does not distinguish between CBZ and DHC or CYH and CYT because the number of H atoms is not considered or between isomers. A study of different isomers of dichloronitrobenzene, suggested that highly polymorphic molecules may be less stable in lattice energy than monomorphic isomers.^127^ Overall (Figure 8) demonstrates that ψ_crys_(PBE+TS) lattice energies are significantly overestimated, and even this crude comparison shows that none of the other methods reliably predict sufficient agreement with the experimental data over this range of molecules for the consideration of experimental and systematic modeling errors^23,64,128^ to be likely to produce reliable agreement.

There is a systematic error in this comparison of lattice energies with −Δ*H*_sub_ from the neglect of the correction for thermal and zero-point energies.^48^ The traditional estimate, using some severe approximations that are most plausible for very rigid molecules, would be 2*RT* or 2.5 kJ mol^–1^ at ambient temperature, which is insignificant on the scale in Figure 8. However, the vibrational correction is challenging to obtain from computation as it needs large periodic cells (or Brillouin zone averaging), and anharmonicity and nuclear quantum effects are often important^129^ as is the effect of the anisotropic thermal expansion in molecular crystals.^130^ Calculations of the vibrational energy correction for the X23 data set of small-molecule crystal structures,^17^ are estimated to have a magnitude of generally less than 10 kJ mol^–1^. The coupling of the intermolecular and intramolecular modes in the crystal will be far greater in the larger flexible molecules in this data set, and semiempirical methods suggest that this will give a wider range of vibrational corrections than for rigid molecules.^131^

The difference in the temperature correction is vital for judging whether the polymorphs switch relative stability with temperature (i.e., are enantiotropically related). Rigid-body harmonic calculations using a ψ_mol_ potential on Nyman’s set of 475 pairs of polymorphs estimated that 21% were enantiotropically related.^132^ In our data set, less than a third are known to have either a monotropic (FLU, OXC) or enantiotropic (CRN, CBZ, MFA, SMZ) relationship between their most commonly observed polymorphs, a quarter are currently considered monomorphic (PTH, SAC, FEA) or could not interconvert (IBP, NAP), and there seems to be currently insufficient evidence for the remaining 45%. Hence, it is important to compare free energies, even for the stability ranking. A benchmark set of relative vibrational free energy polymorph corrections, PV17, was constructed^133^ using the 17 examples from the extensive polymorph library of Nyman^132^ where both polymorphs had a cell volume of less than 600 Å^3^ enabling plane wave DFT to be used to calculate the vibrational free energies and free-energy differences (Δ*F*_vib_) between each pair. This confirmed that the vibrational free-energy corrections are small, having a mean value of 1.0 kJ mol^–1^ and a maximum value of 2.3 kJ mol^–1^ for the PV17 set. This study found that Δ*F*_vib_ values calculated using various approximate methods can have mean absolute errors equivalent to or larger than the vibrational free-energy corrections themselves.^133^

Experimental data on sublimation thermodynamics are often lacking, often because there is insufficient vapor pressure or the crystal decomposes before subliming, and the crystal may transform to a high-temperature polymorph during the experiment. The experimental errors^64^ are often such that its suitability for judging the energy scale of organic crystal modeling has been questioned.^31^ The challenge of evaluating heats of sublimation has recently been illustrated by the Z20 database of critically evaluated enthalpies of sublimation^70,134^ of low-temperature phases, which has 12 organic molecules, the largest of which are acetic acid and butane. Expensive Amoeba-embedded CCSD(T)/CBS fragment-based calculations of the sublimation enthalpies in the Z20 database gave a mean absolute deviation of 4.2 kJ mol^–1^ (14%), showing that a much more acceptable level of accuracy can be achieved for these small molecular crystals.^134^

The experimentally observed forms of the molecules in the CPOSS209 data set have been roughly classified by types of experiment that produced the sample used for structure determination (SI Section S3.1) in Figure 8 to see if there are any general trends behind the spread in lattice energies of the polymorphs shown in detail in Figure 2 and Figures 4 to 6. This shows that many new polymorphs have been found from rather specific modified solution crystallization, usually with the presence of additives, for example, new polymorphs found instead of the anticipated cocrystals. The classification shows that many polymorphs are not found from solution crystallization, which may be as simple as heating or as complex as sublimation onto specifically designed templates. As solution crystallization is the easiest method of producing crystals suitable for determining the structure by single crystal diffraction, the nonsolution methods are usually more demanding in terms of characterization. There are no clear trends in Figure 8 – some methods, such as desolvating solvates (c.f. the *R*3̅ structures of the carbamazepine family, Section 3.1.2) or making a polymorph by a solid-state reaction,^135^ may lead to higher than “normal” energy differences. The expectation that the low-temperature form is more stable in lattice energy than the high-temperature form holds with the ψ_crys_(PBE+MBD) model for the polymorph pairs that are known to be enantiotropically related (CRN β vs CRN γ, CBZ III vs CBZ I, MFA I vs MFA II, FFA III vs FFA I, SMZ II vs SMZ I for low-temperature vs high-temperature forms).

### 3.4 Structure Reproduction

**Figure 9:**
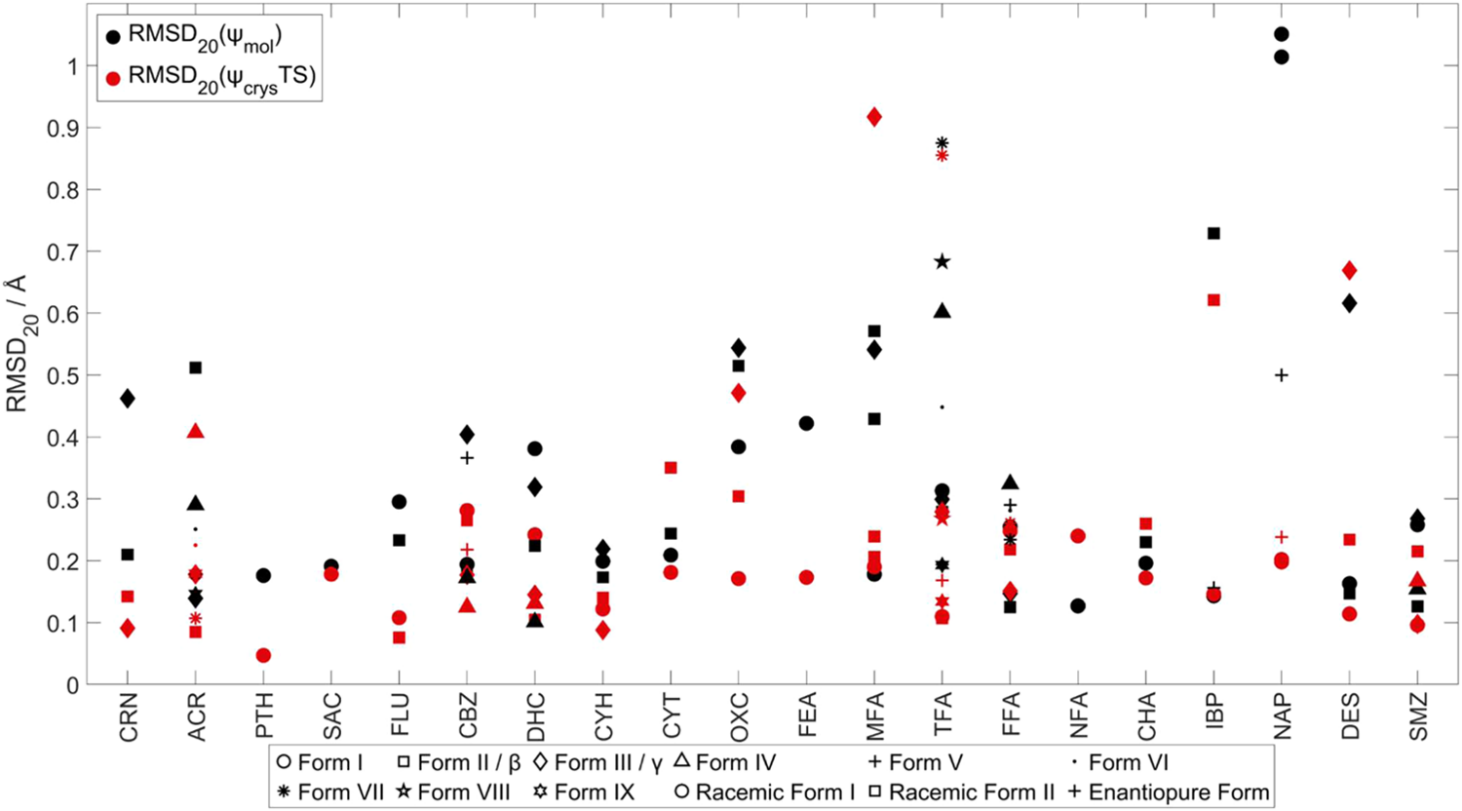
Crystal structure reproduction as given by the RMSD_20_ of the experimentally observed crystal structures of the 20 compounds, colored black for ψ_mol_ and red for ψ_crys_(PBE+TS).

The ability of the different methods to reproduce the experimental crystal structures, in terms of the RMS distances between the nonhydrogenic atoms in the optimum overlay of a 20-molecule cluster, is shown in Figure 9. One might expect the ψ_crys_(PBE+TS) method, where all of the atomic positions are optimized, to perform better than the ψ_mol_ approach, where only selected torsion and bond angles are allowed to change in response to the crystal packing forces. This is generally, but not always, the case. The ψ_mol_ method has the advantage that the exp-6 parameters were fitted to the experimental crystal structures of selected rigid molecules. It should be noted that part of the popularity of ψ_crys_(PBE+TS) comes from its success in reproducing experimental crystal structures.^43^ Yet there are a few spectacular failures of the ψ_crys_(PBE+TS) model, including for two fenamate crystal structures, which may be attributed to the delocalization error in this functional making the conformational profile very sensitive to the electronic structure method used.^136^ The fenamates are similar to ROY where the delocalization error gives rise to the failure to locate one of the polymorphs of ROY as a lattice energy minimum.^137^ It is not uncommon to find that one structure is an outlier in how well it is reproduced by a given method.^138,139^ The inability to reproduce the structure is clearly very polymorph-specific and should just reflect the distance from the nearest minimum in the potential energy surface provided care is taken with the optimization procedure.

The more relevant question is how well should we expect the experimental structures to be reproduced? Half of the structures in this study were determined between 80 and 123 K (SI Tables in Section S2), and there is a huge variation in the thermal expansion of organic crystals, with a recent review of over 4000 organic crystals suggesting that one-third may have at least one direction with negative thermal expansion.^140^ The zero-point motions alone were estimated to increase the molecular volume of crystalline imidazole crystal by 4%^141^ and ammonia by 3%.^5^

The criteria for evaluating the similarity of crystal structures is an active area of debate, as it is important in the removal of duplicates (clustering) in a CSP workflow and the discussion of how different structures have to be in order to be considered polymorphs. The unit cell has too much arbitrariness, and hence we have focused on the optimal overlay of the coordination sphere.^51^ Although 15 molecules define the first coordination sphere for small, spherical molecules, for larger elongated molecules, a cluster of 20 may be needed. The distinction between polytypes is particularly tricky, as shown by two *Z*′ = 3 CSP-generated structures of methyl 2-aminobenzoate only being distinguished by considering a cluster of 70 or more molecules.^44^ Thermal expansion plays a significant role, with the RMSD_20_ overlap of determinations at 100 K and room temperature ranging from 0.082 Å for OXC I to 0.185 Å for MFA I. A similar comparison based on naphthalene and form I of paracetamol^83^ suggested that an RMSD_30_ ≤ 0.204 Å can be explained by the temperature-free nature of lattice energy structural optimization. It is worth noting that determinations at the same temperature (within 5 degrees) have RMSD_20_ overlaps ranging from 0.015 Å for NFA I to 0.103 Å for CHA II. Thus, the standard of RMSD_30_ < 0.4 Å being an acceptable reproduction^83^ implies that the vast majority of the structure reproductions in Figure 9 are reasonable. Alternative approaches for comparing crystal structures are being developed, such as the use of pointwise distance distributions,^142,143^ which have considerable efficiency advantages for comparing large data sets of structures.^44^

### 3.5 Illustrative Use of the CPOSS209 Data Set

**Figure 10:**
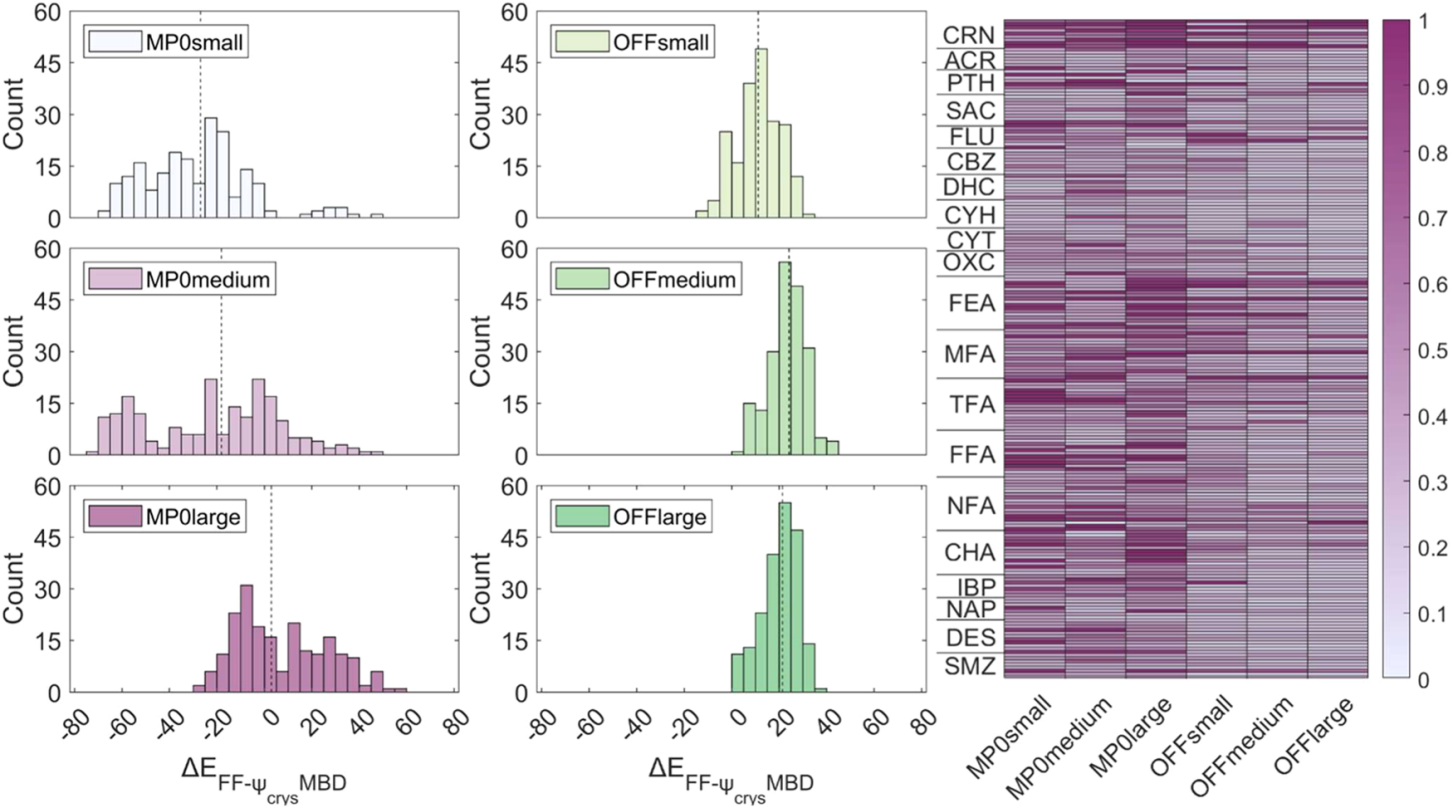
Histograms of the absolute differences in lattice energy between the ψ_crys_(PBE+MBD) single point energy of the ψ_crys_(PBE+TS) optimized structures for (left) the MACE-MP-0+D3(BJ) and (middle) the MACE-OFF23 optimization, with the small, medium, and large parameter sets. The vertical dashed lines indicate the median error, and the data are binned into 5 kJ mol^–1^ bins. (Right) the RMSD_20_ overlap of the ψ_crys_(PBE+TS) optimized structures with the MACE-MP-0+D3(BJ) and MACE-OFF23 optimized crystal structures in Å. Only one label is included for each molecule, for clarity.

The lattice energies of the 209 crystals optimized with the foundation MACE-MP-0+D3(BJ) and MACE-OFF23 models are shown in SI Figures S19 and S20. The absolute difference in the lattice energy between the single point energies calculated with the ψ_crys_(PBE+MBD) method and the MACE-MP-0+D3(BJ) and MACE-OFF23 optimizations is shown in Figure 10, along with the RMSD_20_ matches between the ψ_crys_(PBE+TS) optimized structures and the force-field optimized structures.

The MACE-MP-0 foundation model, even with D3(BJ) dispersion, produced very large deviations from the ψ_crys_(PBE+MBD) lattice energies (Figure 10 and SI Figures S19 and S20), whereas the MACE-OFF23 foundation model gave much closer lattice energies despite not having been trained on crystal structures. The variation of the results with the size of the parameter set is significant. The plots of lattice energies for the different molecules (SI Figure S19) show that MACE-OFF23 lattice energies are usually far more consistent with the ψ_mol_ and ψ_crys_(PBE+MBD) values than MACE-MP-0+D3(BJ) but do not show any obvious correlation with the type of molecule or elements involved. The plot relating the energies to individual structures (SI Figure S20) shows that even MACE-OFF23 leads to a different ranking of the relative stabilities of the structures for any given molecule.

The modeling of the molecular conformation is very important for flexible molecules, as a preference for an unrealistic conformation can frequently result in an incorrect modeling of the entire crystal structure, e.g., an inability to form hydrogen bonds from the poor positioning of the hydrogen-bonding protons or an inability to close pack. The energy differences between the different conformational minima for the isolated molecules, Δ*E*_conf.min_, are extremely poor for MACE-MP-0+D3(BJ), where sometimes the conformations were unrealistic (SI Section S6.2), with the MACE-OFF23 conformations and energy differences being more realistic. This is not surprising as MACE-OFF23 was trained on organic molecule structures. The training data set used for MACE-OFF23 was ωB97M-D3(BJ)/def2-TZVPP, which should be more accurate than the PBE0/6–31G(d,p) used in most of the ψ_mol_ energies, and both are a higher level of functional than the ψ_crys_(PBE+D) calculations. However, the MACE-OFF23 conformational energy differences depend on the size of the parameter set, implying that the fitting errors have lost some of the accuracy of the training data. The correlations between the ψ_mol_ and ψ_crys_(PBE+D) conformational energy differences (SI Figure S16) are better than those with either MACE model (SI Figure S23), but there is still a difference between the TS and MBD dispersion models, showing the sensitivity of molecular conformational energies to the level of theory used.

#### 3.5.1 Reproduction of Experimental Crystal Structures

**Figure 11:**
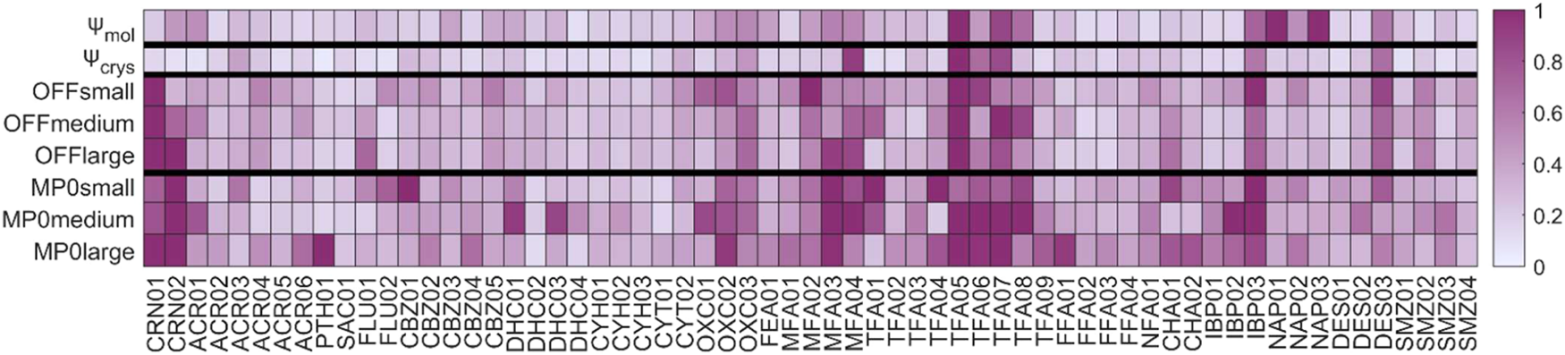
Crystal structure similarity of structures minimized with ψ_mol_, ψ_crys_(PBE+TS), MACE-OFF23, and MACE-MP-0+D3(BJ) with the experimentally determined structures colored by RMSD_20_. Entries in the darkest purple denote that either fewer than 20 molecules were matched in Mercury (only for some CRN structures) or the RMSD_20_ is above 1.0 Å.

Figure 11 shows how well the experimentally observed crystal structures are reproduced by different computational methods. It is clear that the ψ_crys_(PBE+TS) method is the best overall at reproducing the experimental crystal structures. It is also apparent that certain crystal structures are poorly reproduced with all of the methods. Some of these errors can be attributed to the determination of the experimental structure. For example, IBP03 is a crystal structure that was determined from powder diffraction data, and DES03 is a crystal structure that was recorded at high temperatures. MFA02/MFA03 and TFA04/TFA05 are ordered structures that mimic the components of disordered experimental crystal structures.

It is encouraging that most crystal structures that were well reproduced with the ψ_mol_ and ψ_crys_(PBE+TS) methods are reproduced adequately with the machine-learned force fields. Many crystal structures are so well-defined by hydrogen bonding and close packing that they are very easy to reproduce. Historically, it has been known that most organic crystal structures are close-packed,^109^ with a packing coefficient of 65–75% (74% is the packing coefficient of hard spheres), and so the modeling of the repulsive wall around the molecule is of prime importance in the reproduction of crystal structures where the “bumps fit into the hollows”.^144^ However, it is also noticeable that neither of the machine-learned force fields reproduced both of the experimental CRN crystal structures (SI Table S29), switching whether the single minimum was closer to one structure or the other. This also happens when atomic point charges are used as a model for the electrostatic interactions rather than atomic multipoles. The polymorphs of CRN are an example of specific crystal structures that are very sensitive to the underlying force field because the molecules can slip on the potential energy surface, as seems likely from visualizing the structures (SI Figure S22). Polymorphs with shear planes are often more difficult to reproduce than those where the tight fitting of bumps into hollows or directional hydrogen bonding defines the crystal packing in all three dimensions. Hence, the reproduction of certain crystal structures can be very sensitive to the balance of dispersion, repulsion, and other intermolecular forces defining multiple shallow minima in broad potential wells, but other structures can be reproduced by a range of force fields that give very different lattice energies.

The sensitivity of the results of both of the MACE force fields to the size of the parameter set is marked (Figures 10 and 11). A sensitivity to the size of the parameter set is to be expected, and it could be argued that the large parameter set is not a significant improvement over the medium for MACE-OFF23 (Figure 10). The difference in computing requirements is significant, notably in terms of the required memory of the graphics cards used: to complete some of the optimizations of the larger crystal structures (>500 atoms/unit cell) with the large parameter set, we needed to perform the calculations on NVIDIA A100 GPUs, which have 80 GB of memory, while for all other optimizations NVIDIA A16 GPUs were sufficient, with 16 GB of memory for each chip of the graphics card. Modeling organic crystal structures is very sensitive to the modeling of the long-range electrostatic and dispersion interactions because of the poor convergence of the lattice summations. The anisotropy around each atom is also marked, as it has to describe the covalent bonds, any intramolecular steric clashes, and the intermolecular forces. The anisotropy of the intermolecular forces is much improved by using distributed multipoles rather than atomic charges, showing the challenge to the MACE parametrization in modeling the directionality of intermolecular interactions such as hydrogen bonding and π–π stacking effectively in balance with the highly directional covalent bonding.

Thus, using the CPOSS209 data set has shown that despite being fitted to a wide range of crystal structures, MACE-MP-0 is unable to model organic crystal structures, which is hardly surprising given how little organic chemistry is sampled in the training set, which is heavily skewed to inorganic oxide structures. The MP-0 data set is composed of crystal structures that are much more strongly bound than organic crystals. The dispersion contribution is a major component of the MACE-MP-0+D3(BJ) lattice energies (SI Figure S21), so our attempt to improve the MACE-MP-0 potential by just adding a damped D3(BJ) dispersion model is insufficient. This reinforces the conclusion that the model needs improvement in describing intermolecular interactions and that taking into account the different sizes of the atoms may be helpful when modeling mixed organic/inorganic crystals, as the organic elements tend to be smaller.^81^

It is encouraging that MACE-OFF23 has much more realistic lattice energies and structures for the CPOSS209 data set, despite being only trained on a large database of organic molecules and dimers. It could be used as a starting point for training a condensed-phase, molecule-specific force field based on, for example, MD-generated conformations, which could make the initial stages of crystal structure prediction more efficient. It could also be refined against the results of a crystal structure prediction,^83,145^ to enable MD simulations at similar accuracy for use in molecular dynamics landscape reduction^9,10^ or other property calculations.

## 4 Discussion

We have gathered together sets of known polymorphs, where there is a crystal structure available, for a reasonably diverse set of 20 organic compounds, which are larger than those in the X23 data set used by many computational scientists but small relative to pharmaceuticals. This has been supplemented by hypothetical structures derived from closely related molecules that might naively be expected to have similar crystallization behavior. The data sets also contain structures generated by a hybrid ψ_mol_ model CSP study, which are energetically competitive with the known forms but have sufficiently different packing, such as different conformations or hydrogen bonding, that the structures, if found as metastable polymorphs, might have difficulty in undergoing a solid-state transformation to the most stable form. All of these structures have been refined by a periodic ψ_crys_(PBE+TS) calculation, with a single point lattice energy evaluation with ψ_crys_(PBE+MBD). This significantly improves the number of cases where the structure with the lowest lattice energy corresponds to the polymorph believed to be the most stable at low temperatures, from 6 for ψ_mol_ to 15 for ψ_crys_(PBE+TS) and 19 for ψ_crys_(PBE+MBD). The extent to which the known and computer-generated structures are reranked in relative lattice energy varies, but the remarkable differences in the absolute lattice energies show that the success of these models in CSP involves considerable cancellation of errors. Full optimization of the crystal structure by ψ_crys_(PBE+TS) generally slightly improves the reproduction of the experimental crystal structures, but this difference is not so significant, given the extent to which a lattice energy minimum can represent an experimental crystal structure at ambient temperature (Section 3.4).

The resulting set of 209 ψ_crys_(PBE+TS) optimized structures, the CPOSS209 data set, is provided to help develop the computational prediction of polymorphism in a variety of ways, mainly to avoid the problems of assuming that “one size fits all” in assessing new methods.

### 4.1 Caveats on Distinguishing Experimental and Hypothetical Structures

Although all compounds are solid at ambient conditions (in contrast to the X23 data set), there is no “one size fits all” approach to polymorph screening, partly from the range of solubilities in different solvent mixtures and varying susceptibility to thermal degradation. It is practically impossible to cover the huge diversity of crystallization methods that have resulted in finding a new polymorph for some systems,^146,147^ or the range of additives or impurities that can be associated with the discovery of a new form. The extent of screening that has been carried out on the compounds in the CPOSS209 data set is very variable, and many of the experimental polymorphs have been found by serendipity,^110^ although some have been discovered with the aid of CSP.^53,55,56,59,104,105^ Polymorphs are being discovered continuously, sometimes by developments in polymorph screening techniques. Indeed, recent development of efficient screening from bulk and confined melts^148^ found an additional coarse spherulite structure of CBZ VI and evidence for TFA X, which have yet to be structurally characterized, in common with NFA II.^112^ Similarly, we have only classified the crystallization conditions used to produce the samples used in the crystallographic studies in the CSD. The most stable polymorph can often be accessed by a range of conditions, although the recipes for producing metastable polymorphs can often be hard to reproduce^149^ or varied^150,151^ (c.f. FLU II, which has been obtained by type 1 and type 2 experiments^55,90^). Indeed, the range of conditions that lead to a specific polymorph is not uniquely defined, as discussed for ACR,^53^ and there are apparently similar or identical conditions that lead to different forms or a mixture of concomitant forms, complicating and confusing their identification and characterization. Nonetheless, this data set, by associating the crystal structure with a method of crystallization, provides a starting point to work on the biggest challenge facing CSP: given a hypothetical structure that is thermodynamically competitive with the known forms, can you design a recipe for finding it, or show that it is sufficiently kinetically unfavorable that it cannot appear? Of this data set, only saccharin does not have any hypothetical structures that are thermodynamically competitive so that polymorphs appear unlikely on thermodynamic grounds.

The increasing ability to detect and characterize new forms also impacts the proportion of observed polymorphs that have been structurally characterized, often aided by having CSP structures available to provide approximate models. This is illustrated by olanzapine, which, despite the extensive solid form screening work at Eli Lilly to defend the patent on this blockbuster antipsychotic drug,^152^ had a new form discovered in work with polymer dispersions,^153^ and only recently was the crystal structure of form III established definitively by electron diffraction.^154^ The rapid emergence of electron diffraction may well result in the characterization of many more new polymorphs that appear concomitantly with other forms, possibly below the limits of detection by powder X-ray diffraction.^155^ In some cases of high *Z*′ structures, it is unclear whether the diffraction data could have been modeled equally well by a smaller unit cell with a disorder model. Disorder is frequent in organic crystals, as highlighted by the experimental work involved in the seventh blind test,^44^ and poses a significant challenge to computational modeling^156^ unless there are clear archetype crystal structures.^157^ Only the cases where the disorder was approximately 50:50 between well-defined components have these archetype structures been included in this data set. Experimental determination as to whether the disorder is temperature-dependent is vital to the appropriate modeling of the relative stability. The archetype crystal structures may well be generated in a CSP study and used to estimate the thermodynamic contribution to the lattice energy from the static, configurational disorder.^158−160^

Even more generally, the experimental basis for verifying the calculated thermodynamic relationship between polymorphs is lacking, as evidenced by the limited available heats of sublimation. The experimental work is often hampered by the problems of ensuring polymorph purity throughout the experiment, although experiments that enable the simultaneous monitoring of the crystal structure with thermal characterization are possible with synchrotron facilities.^161^ Thus, reassessment of the existing thermodynamic data is often valuable. For example, the recent study^6^ of the relative stability of the FFA polymorphs contributes to disentangling the prevailing controversies on the polymorph ranking.^59,111^ Fortunately, this need has been recognized by an EU COST action, BEST-CSP, which aims to provide some benchmark studies of polymorphic pairs.

Although a CSP search may be an invaluable aid in the characterization of new forms, this data set shows that there is no “one size fits all” set of parameters for a CSP in terms of the range of *Z*′ and density limits considered in structure generation and the lattice energy range of plausible polymorphs. The prediction of polymorphs resulting from desolvation occurring late in the crystallization process is a particularly challenging type, as exemplified by the channel structures of the carbamazepine family (see Section 3.1.2). Thus, this data set may be useful to help define the limitations on the types of polymorphs that may be generated in a given type of CSP, as new methods appear.^162,163^ This data set does not include any examples where the molecule may racemize, change tautomer, or otherwise alter the covalent bonds upon crystallization, although examples such as barbituric acid^122^ and guanine^164^ are known to provide this type of challenge to the fixed covalent bonding assumed in CSP. This is industrially relevant as a GSK challenge to developing CSP methods suitable for their portfolio included systems where it was necessary to consider tautomers.^162^

### 4.2 Challenge of Theoretical Modeling with Methods Used in This Work

In this work, we have contrasted the ψ_mol_ lattice energies as the output from a CSP search with the far more expensive ψ_crys_(DFT+D) calculations that are often used to refine the structures and energies. Our study has been limited to 10 compounds in two families of closely related molecules, and 10 other molecules, and so is far from representing the diversity of organic compounds whose solid form landscapes are of interest. However, this range is sufficient to indicate the diversity of the experimental structures, even for closely related molecules, and hence the challenge of developing computational methods that can be applied to all of the polymorphs of a given molecule, let alone family of molecules. Our inability to afford the ψ_crys_(PBE+TS) calculations on a few fenamate structures and the ψ_crys_(PBE+MBD) single point energies of the *R*3̅ structures of the carbamazepine family, along with the variation in the *k*-point grids and the box size required for the “isolated molecule” calculation, all show that one size of code parameter settings does not necessarily fit all.

The ψ_crys_(PBE+TS) optimized crystal structures are presented as the main test data set, as this method has fully optimized all atomic positions on the potential energy surface, allowing a more complete relaxation of the structure in response to the balance of the intermolecular and intramolecular forces. The ψ_mol_ structures are provided as a subsidiary data set, allowing a test of whether the optimizations are sensitive to small differences in the starting structure, as exemplified by the CRN polymorphs (Section 3.5). The ψ_mol_ structures which we could not optimize with the ψ_crys_ method (FFA IV, FFA V, FFA VI, and TFA IV), are provided to complete the supplementary ψ_mol_ data set.

The quality of the ψ_mol_ and ψ_crys_ potential energy surfaces differs. The PBE functional is known to suffer from delocalization error,^13,165,166^ and ψ_mol_ has used a better-quality charge density for the intramolecular and electrostatic forces, but is limited by the empirical “repulsion-dispersion” model and the lack of polarization. Hence, the approach that is likely to be more accurate will depend on the conformational flexibility and functional groups in the molecule. It will also depend on the specific low-energy structures that are thermodynamically competitive. Thus, it is difficult to estimate the likely accuracy of a given potential energy surface based on statistical methods, even on a data set that was orders of magnitude bigger than this one with considerably more experimental thermodynamic measurements. The conclusions that can be drawn from our three sets of lattice energies are similar to those drawn from a comparison of several flavors of ψ_mol_ and ψ_crys_ calculations with the X23 benchmark data set.^167^ The correlations of the lattice energies calculated by the three methods is fairly poor (SI Figure S17), even for just changing the dispersion model from TS to MBD, but as evident from the comparison of total lattice energies (Figure 8, SI Figure S17) there is a considerable offset as well as slope difference in the best linear relationship. The different offsets will cancel in comparing relative lattice energies, so that cancellation of errors may be particularly favorable in the low relative energy regime of CSP.^168^

### 4.3 Use of the CPOSS209 Data Set for Developing More Accurate Calculations

This data set underlines the problems of developing more accurate methods of evaluating relative polymorph stability that can be applied quite widely. It contrasts with PV17^133^ which chose the 17 polymorphic systems as the only ones where both experimental polymorphs had computationally tractable unit cells.

The challenge facing theoretical methods of modeling organic polymorphism is evident in the huge sensitivity to the dispersion correction. Using the TS dispersion model gives a larger range of lattice energies for the set of structures than the MBD model by over 4 kJ mol^–1^ for CRN, ACR, CYH, MFA, TFA, NFA, and DES, despite the lattice energy being evaluated at identical structures. However, the range is smaller for TS than MBD for our set of crystal structures of SAC, FLU, DHC, OXC, CHA, IBP, and SMZ, showing that there is no correlation with size, flexibility, or functional groups. The TS correction is not only the more approximate model, but is also shown to be significantly overbinding in the comparison of lattice energies with heats of sublimation (Figure 8). Dispersion forces have their origin in electron correlation effects, which are particularly challenging for electronic structure modeling. The most accurate and computationally demanding methods, such as Diffusion Monte Carlo, have only recently been shown to be converging to within the experimental variation for the heavily studied X23 small-molecule crystal database.^7^

A second challenge, exemplified by the fenamates and small drug molecules. is the accurate balancing of the intermolecular and intramolecular forces (i.e., *U*_inter_ and Δ*E*_intra_) that can cause significant adjustment of the molecular conformation and conformational polymorphism^20^ (Section 3.5). Correcting ψ_crys_(PBE+D) lattice energies with a converged conformational energy penalty evaluated by a highly accurate ψ_mol_ method has been shown to make a significant improvement to the rankings of several polymorph pairs.^46,169^ Indeed, this type of molecular correction was used in the seventh blind test by the commercial companies, showing that the state-of-the-art lattice energy evaluations are mixing ψ_crys_ with ψ_mol_.^170^ Another correction that was applied is to (partially) correct GGA functionals, such as PBE, for the delocalization error by a single point calculation with a hybrid functional, such as PBE0.^170^

There are other thermodynamic factors that may need modeling for some systems. There are terms that depend on the morphology of the crystal, such as the surface energy contribution, which can change the relative polymorph stability with size,^171^ and the surface dipole term for polar crystals such as ACR IV (Section 3.1.1). Other systems will provide additional challenges to modeling accuracy, for example, distinguishing between a salt and a cocrystal when there are short hydrogen bonds requires consideration of quantum nuclear effects.^172^

Currently, the most accurate density-functional methods for molecular crystal lattice energies, as judged by the X23 data set, are exchange-hole dipole moment (XDM) dispersion-corrected hybrid functionals, with the use of numerical atomic basis sets.^8^ The numerical basis sets make this approach sufficiently computationally efficient that they have been applied to blind test targets,^173^ and circumvent some of the issues we have identified with a plane wave basis set. Alternatively, the development of dispersion-corrected second-order Møller–Plesset perturbation theory seems promising for improving the balance of intermolecular and intramolecular forces.^174^ These approaches have been evaluated^175,176^ using many of the target systems in the seventh blind test, and the CPOSS209 data set provides an alternative challenge for evaluating these and other developing methods.

### 4.4 Use of Data Set for More Cost-Efficient Methods

As commented by Mihails Arhangelskis^177^ in the overview of the seventh blind test, “It is evident that the future of CSP, particularly for wide adaptation in industry, lies in finding the right balance between the accuracy of the calculations and their computational cost.” This data set is aimed at being useful for evaluating approximate methods, particularly force fields that could be used in Molecular Dynamics simulations for landscape reduction, assessing dynamic disorder, calculating properties, or studying kinetics effects. This application has been demonstrated by the MACE foundation models, where just using this data set quickly showed what adaptions were needed, such as the need to add dispersion to MACE-MP-0, having a protocol for defining a molecule, and evaluating the computational resources needed. Thus, this small test set is useful for picking up problems and deciding which models are worth pursuing (e.g., MACE-OFF23 is more promising than MACE-MP-0+D3(BJ), Section 3.5) before embarking on a larger study. The hybrid approach of combining an ML force field trained on monomers and dimers with a classical long-range force field using atomic multipoles appears promising for molecular condensed phases.^178^

## 5 Conclusions

This study has reviewed the polymorphism of 20 organic molecules, demonstrating the range of structures and crystallization conditions that may produce new polymorphs, and how the improvements in experimental polymorph screening, characterization, and serendipity make it difficult to produce a complete database of experimental polymorphs, let alone a reliable experimental recipe for crystallizing them. This outline of the continually evolving experimental data is essential background for computational studies into polymorph prediction, which would start with a CSP_0 (lattice energy CSP study).

We have produced a data set of 209 periodic ψ_crys_(PBE+TS) optimized, idealized crystal structures, representing the vast majority of the currently experimentally structurally characterized polymorphs, and selected hypothetical structures generated by CSP or molecular substitution, for 20 moderately sized organic molecules. It is hoped that this small data set may provide a useful initial trial for the development of new methods of evaluating organic crystal energies that may be used in CSP. The associated lattice energies from either the ψ_mol_ hybrid anisotropic force field used in the original CSP, the ψ_crys_(PBE+TS) optimization, or a single point calculation with the ψ_crys_(PBE+MBD) model, are sufficiently accurate to rank the known structures among the most stable in lattice energy (Section 3.1). However, the absolute lattice energies differ significantly relative to the available heats of sublimation, far more than is plausible from neglect of the thermodynamic effects of the molecular motions (Section 3.3). The reproduction of the experimental structures is usually acceptable, given the neglect of the structural changes with temperature and zero-point motion and the limitations of the experimental structure determinations (Sections 3.4 and 3.5.1).

The lattice energy calculations are far from state-of-the-art, and it is hoped that this data set will help with the development of highly accurate methods of predicting the thermodynamics of polymorphs and their temperature dependence. It is also suitable for an initial test of more efficiently evaluated energy models, which may be used in molecular dynamics simulations. The utility of the CPOSS209 data set is illustrated by testing two machine-learned transferable force fields developed using the MACE architecture (Section 3.5), where one (MACE-OFF23) is clearly a better foundation model for further molecule-specific development than the other (MACE-MP-0, with or without D3(BJ) damped dispersion).

The prediction of organic polymorphs and their properties is an industrially important challenge to computational modeling techniques, and this paper gives insight into this challenge and a test set of crystal structures for facilitating progress on moderate-sized molecules.

## Supporting Information Available

Molecule and structure selection, methods for ψ_mol_ calculations, convergence testing for ψ_crys_ calculations, experimental crystallization conditions, experimental and estimated heats of sublimation, calculated lattice energies of crystal structures, and calculated isolated molecule energies (PDF)
All_Psi_Crys.mol file of the 59 or 60 molecular conformations optimized with the ψ_crys_(PBE + TS) method (MOL)
All_Psi_Mol.mol file of the 59 or 60 molecular conformations optimized with the ψ_mol_ method (MOL)
All_Psi_Crys.cif and All_Psi_Mol.cif files of the 209 crystal structures, optimized with the ψ_crys_(PBE + TS) and ψ_mol_ methods, respectively; additional_Psi_Mol.cif file of the additional four crystal structures that were only optimized with the ψ_mol_ method (ZIP)

## Supplementary Material

cg5c00255_si_001.pdf

cg5c00255_si_002.mol

cg5c00255_si_003.mol
