# Supplementary material for: One Size Fits All? Development of the CPOSS209 Data Set of Experimental and Hypothetical Polymorphs for Testing Computational Modeling Methods
Source: Cryst Growth Des. 2025 Apr 28;25(9):3186–209. doi: 10.1021/acs.cgd.5c00255 (PMC12063060; doi:10.1021/acs.cgd.5c00255)
Supplement: Supplementary file 1 — cg5c00255_si_001.pdf [file cg5c00255_si_001.pdf]

Supporting Information for:

One size fits all? Development of the CPOSS209 dataset of experimental and hypothetical polymorphs for testing computational modelling methods

Louise S Price[†], *Matteo Paloni*[‡], *Matteo Salvalaglio*[‡], *Sarah L Price*[†,*]

[†]Department of Chemistry, UCL, 20 Gordon Street, London WC1H 0AJ, UK, [‡]Department of Chemical Engineering, UCL, Torrington Place, London, WC1E 7JE

Table of Contents

S1 Molecule selection 4
S1.1 Discussion of required intermolecular gap 5
S2 Structure selection 6
S2.1 Small rigid molecules 6
S2.1.1 Coronene 6
S2.1.2 Acridine 7
S2.1.3 Phthalimide 8
S2.1.4 Saccharine 9
S2.1.5 5-fluorouracil 10
S2.2 Carbamazepine family 11
S2.2.1 Isostructurality 11
S2.2.2 Selected structures 12
S2.2.3 $R3$ packings 13
S2.2.4 Most thermodynamically stable form at low temperature 14
S2.2.5 Previous CSP method 14
S2.2.6 Plane wave basis set cutoff and k-point spacing for crystal structure optimization 14
S2.2.7 Isolated molecule calculations 14
S2.3 Fenamate family 15
S2.3.1 Isostructurality 15
S2.3.2 Selected structures 16

S2.3.3 Experimentally observed polymorphs not included in study ... 19
S2.3.4 Most thermodynamically stable form at low temperature ... 19
S2.3.5 Previous CSP method and reoptimization of structures ... 19
S2.3.6 Plane wave basis set cutoff and k-point spacing for crystal structure optimization ... 20
S2.3.7 Isolated molecule calculations ... 20
S2.4 Small drug molecules ... 20
S2.4.1 Chalcone ... 20
S2.4.2 Ibuprofen ... 22
S2.4.3 Naproxen ... 23
S2.4.4 Desloratadine ... 25
S2.4.5 Sulfamerazine ... 27
S3 Experimental Information ... 28
S3.1 Crystallization conditions ... 28
S3.1.1 Rigid molecules ... 28
S3.1.2 Carbamazepine family ... 28
S3.1.3 Fenamate family ... 29
S3.1.4 Small drug molecules ... 30
S3.2 Experimental and estimated heats of sublimation ... 30
S3.2.1 Experimental heats of sublimation ... 30
S3.2.2 Estimated heats of sublimation ... 31
S4 Results by molecular family ... 33
S4.1 Small rigid molecules ... 33
S4.1.1 Structure optimizations ... 33
S4.1.2 Molecule optimizations ... 38
S4.2 Carbamazepine family ... 38
S4.2.1 Structure optimizations ... 38
S4.2.2 Molecule optimizations ... 42
S4.3 Fenamate Family ... 43
S4.3.1 Structure optimizations ... 43
S4.3.2 Molecule optimizations ... 48
S4.4 Small drug molecules ... 49
S4.4.1 Structure optimizations ... 49
S4.4.2 Molecule optimizations ... 54
S5 Comparison of results across the ψmol and $\psi crys$ datasets ... 57
S5.1 Comparison of isolated molecule energy differences with different methods ... 57
S5.2 Comparison of absolute lattice energies with different methods ... 58
S5.3 Energy comparison between chiral and racemic crystal structures ... 59
S6 Use of the database structures for evaluating the MACE-OFF23 and MACE-MP-0+D3(BJ) force fields ... 60

S6.1 Comparison of crystal structure optimizations 60
S6.1.1 Role of dispersion correction 62
S6.1.2 Reproduction of coronene experimental crystal structures 62
S6.1.3 Comparison of experimental crystal structures of CRN β and CRN γ 63
S6.2 Comparison of isolated molecule conformations 64
S6.2.1 Conformational differences 65
S7 Notes on provided datasets of computed crystal and molecular structures 66
S7.1 ψmol optimized crystal structures 66
S7.2 $\psi crys$(PBE+TS) optimized crystal structures 66
S7.3 Structures where symmetry added by Platon required special consideration 66
S7.3.1 CRN 66
S7.3.2 PTH 66
S7.3.3 FLU 66
S7.3.4 FEA 66
S7.3.5 MFA 67
S7.3.6 TFA 67
S7.3.7 NFA 67
S7.3.8 IBP 67
S7.3.9 NAP 67
S8 Full author lists for truncated references 67
S8.1 From main paper 67
S8.2 From SI 70
S9 Supporting Information References 70

S1 Molecule selection

Table S1. Summary of systems chosen. For each molecule, the table gives the three letter abbreviation, CSD REFCODE stem, molecular formula, number of experimentally observed polymorphs included (and number of experimentally observed polymorphs that could not be included), number of computer-generated structures included, and the most stable form at low temperature (to the best of our knowledge) used to calculate relative lattice energies. The ab initio method for the ψ_{mol} optimization of the isolated molecule is given. The plane wave basis set cutoff and k-point spacing used in the periodic electronic structure calculations are given, along with the minimum intermolecular separation and molecular box volume for the equivalent molecular optimization in the ψ_{crys} calculations.

Molecule	REFCODE	Formula	# polymorphs included (not included)	# CSP strs included	Low T form	Molecular ab initio method (ψ_{mol} molecular optimization and ΔE_{intra})	plane wave basis set cutoff / eV	k-point spacing / $Å^{-1}$	Intermol. Gap / Å	mol box volume / $Å^3$
Rigid molecules										
Coronene (CRN)	CORONE	$C_{24}H_{12}$	2	7	β form	PBE0/6-31G(d,p)	1000	0.05	14	7764
Acridine (ACR)	ACRDIN	$C_{13}H_9N$	6	1	Form IX	MP2/6-31G(d,p)	800	0.1	11	3841
Phthalimide (PTH)	PHALIM	$C_8H_5N_1O_2$	1	7	Form I	MP2/6-31G(d,p)	900	0.1	9	2012
Saccharin (SAC)	SCCHRN	$C_7H_5N_1O_3S$	1	9	Form I	PBE0/6-31G(d,p)	900	0.1	11	3891
5-fluorouracil (FLU)	FURACL	$C_4H_3N_2O_2F$	2	4	Form I	MP2/6-31G(d,p)	900	0.1	11	2800
Carbamazepine family										
Carbamazepine (CBZ)	CBMZPN	$C_{15}H_{12}N_2O$	5	4	Form III	PBE/6-31G(d,p)	1100	0.05	10	4573
Dihydrocarbamazepine (DHC)	VACTAU	$C_{15}H_{14}N_2O$	4	4	Form II	PBE/6-31G(d,p)	1100	0.05	10	4772
Cyheptamide (CYH)	TEVSOD	$C_{16}H_{15}NO$	3	6	Form I	PBE/6-31G(d,p)	1100	0.05	10	4681
Cytenamide (CYT)	SODNOP/SOGLEG	$C_{16}H_{13}NO$	2	5	Form II	PBE/6-31G(d,p)	1100	0.05	10	4640
Oxcarbazepine (OXC)	CANDUR	$C_{15}H_{12}N_2O_2$	3	5	Form I	PBE/6-31G(d,p)	1100	0.05	10	4728
Fenamates family										
Fenamic Acid (FEA)	QQQBTY	$C_{13}H_{11}N_1O_2$	1	16	Form I	PBE/6-31G+(d)	900	0.1	5	1356
Mefenamic Acid (MFA)	XYANAC	$C_{15}H_{15}N_1O_2$	3	12	Form I	PBE/6-31G+(d)	700	0.1	6	2090
Tolfenamic Acid (TFA)	KAXXAI	$C_{14}H_{12}N_1O_2Cl$	8 (1)	7	Form I	PBE/6-31G+(d)	700	0.1	7	2544
Flufenamic Acid (FFA)	FPAMCA	$C_{14}H_{10}F_3N_1O_2$	4 (4)	11	Form III	PBE/6-31G+(d)	1000	0.1	9	3982
Niflumic Acid (NFA)	NIFLUM	$C_{13}H_9N_2O_2F_3$	1	16	Form I	PBE/6-31G+(d)	900	0.1	8	3154
Small drug molecules										
Chalcone (CHA)	BCYACO	$C_{15}H_{12}O$	2	12	Form I	MP2/6-31G(d,p)	900	0.08	7	2072
Ibuprofen (IBP)	IBPRAC / JEKNOC	$C_{13}H_{18}O_2$	3	4	Racemic Form I	PBE0/6-31G(d,p)	900	0.1	6	2048
Naproxen (NAP)	PAPTUX / COYRUD	$C_{14}H_{14}O_3$	2	4	Racemic Form I	PBE0/6-31G(d,p)	900	0.1	7	2810
Desloratadine (DES)	GEHXEX	$C_{19}H_{19}ClN_2$	3	8	Form I	PBE0/6-31G(d,p)	900	0.06	8	4121
Sulfamerazine (SMZ)	SLFNMA	$C_{11}H_{12}N_4O_2S$	4	4	Form II	PBE0/6-31G(d,p)	900	0.1	17	13508

S1.1 Discussion of required intermolecular gap

There is a wide range of box sizes necessary for the isolated molecule calculations in the ψ_{crys} method. The box required to adequately separate the molecules so that the intermolecular forces are negligible will vary with the range of these forces. Table S2 lists the molecular dipole moments and minimum box sizes to approximate infinite separation needed for the ψ_{crys} calculations and Figure S1 is a plot of this data. This shows that, while there is generally a correlation, with SMZ exhibiting the largest dipole moment and requiring the largest box size, there are some outliers. In particular, CRN is a symmetrical molecule with no dipole moment, but a large quadrupole moment, and requires the second largest intermolecular gap. The fenamate family require the smallest boxes probably because all the polar functional groups in the fenamate molecules are on the same side, and in the periodic arrangement are proximate to non-polar regions. Thus, whilst having a box that was sufficiently large and of a constant size for all twenty molecules would be computationally very inefficient, the box size defined by our methodology reflects the intermolecular interactions of the specific molecule and conformation.

Table S2. Molecular dipole moments and required intermolecular gap for isolated molecule calculations.

Molecule	Required intermolecular gap / Å	Molecular dipole moment /Debye	Molecule	Required intermolecular gap / Å	Molecular dipole moment /Debye
CRN	14	0.00	FEA	5	0.85
ACR	11	2.2	MFA	6	0.71
PTH	9	3.59	TFA	7	2.20
SAC	11	4.01	FFA	9	2.85
FLU	11	4.52	NFA	8	4.84
CBZ	10	3.29	CHA	7	3.43
DHC	10	3.89	IBP	6	1.44
CYH	10	3.31	NAP	7	1.34
CYT	10	3.08	DES	8	3.29
OXC	10	3.95	SMZ	17	6.67

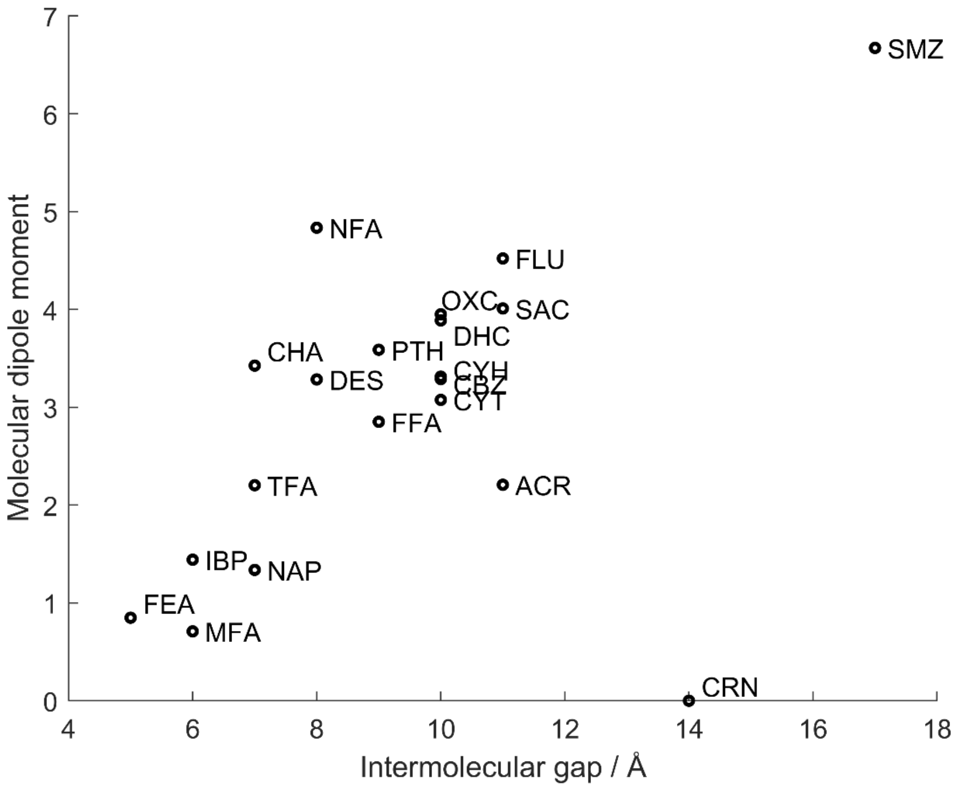

Figure S1. Correlation of minimum intermolecular gap to approximate infinite separation of the molecules with dipole moment (Table S2).

S2 Structure selection

Crystal structures were selected if they were:

- Generated in the search and equivalent to experimentally observed structures
- Experimentally observed structures that were not (or could not be) generated in the search, optimized by same method as search
- The lowest energy structure in the ψ_{mol} energy ranking
- The lowest energy structure in a chiral packing in the ψ_{mol} energy ranking (or lowest energy structure in a Sohncke space group for symmetrical molecules)
- Any search structures that had the same packing as experimentally observed structures for related molecules
- Low energy structures that had conformations, hydrogen-bonding motifs or other packing features that were very distinct from those found in the other selected structures.

The target was to consider between six and ten crystal structures for each molecule. Where there were more or fewer considered, this is mentioned in detail. In many cases, these structures were taken directly from the published search, but to ensure consistency across families, some were reoptimized with CrystalOptimizer to allow the same degrees of freedom to be optimized. In most cases, the ψ_{mol} optimized structure was taken as the starting point for the ψ_{crys} optimization, but when equivalent ψ_{crys} structures were available, these were used to conserve computational resources.

S2.1 Small rigid molecules

S2.1.1 Coronene

S2.1.1.1 Selected structures

All structures of CRN were based on stacks of the molecule, with each molecule offsetting the one below. The difference is in the arrangement of the stacks.

Table S3. Structures selected for CRN.

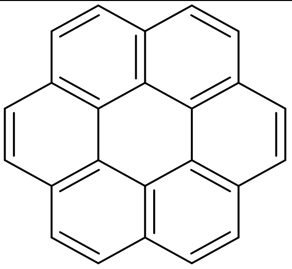

Name	Coronene
CSD REFCODE	CORONE
Formula	$C_{24}H_{12}$
ψ_{mol} published	Potticary et al.[1]
ψ_{crys} published	Potticary et al.[1]

Key	Original ψ_{mol} label	Z′	Space group	mols / cell	Reason
CRN01	ag11 (=CORONE04, 80 K, β form)	1	$P2_1/c$	4	Experimentally observed (beta stacks)
CRN02	ak33 (=CORONE03, 100 K, γ form)	1	Pn	2	Experimentally observed (gamma stacks)
CRN03	af134	2	$P1$	2	Lowest energy ψ_{mol} structure (Sohncke space group) (parallel stacks)
CRN04	ai133	1	$P2_1/c$	4	Packing (parallel stacks)
CRN05	ak1	1	$P2_1/c$	4	Packing (beta stacks)
CRN06	ak12	1	$P2_1/c$	4	Packing (offset stacks in both directions)
CRN07	aq112	1	$P2_12_12_1$	4	Packing (double gamma stacks)
CRN08	fc27	1	$P2_1/c$	4	Packing (double gamma stacks)
CRN09	fd132	4	$P1$	4	Packing (double beta stacks)

S2.1.1.2 Most thermodynamically stable form at low temperature

CRN β is the low temperature form[2] although the only determination on the CSD (CORONE04) was for a sample crystallized in an external magnetic field at ambient.[3] It is enantiotropically related to the usual ambient CRN γ. Energies are calculated relative to CRN01 (CRN β).

S2.1.1.3 Previous CSP method

A rigid molecule Z'=1 search was carried out in MOLPAK, using the PBE0/6-31G(d,p) optimized molecule. Structures were optimized with DMACRYS 2.0.8, using multipoles derived from the PBE0/6-31G(d,p) charge density and the FIT parameters to model the repulsion-dispersion interactions. Some structures were found to not be true minima, and the symmetry was reduced. However, the structures included in Figure 9 of the previously published search[1] only included Z'=1 structures after flexible molecule refinement with CrystalOptimizer.

S2.1.1.4 Plane wave basis set cutoff and k-point spacing for crystal structure optimization

Convergence of the plane wave basis set cutoff and k-point spacing was tested for CRN01. The point at which increasing the plane wave basis set cutoff by 100 eV led to a change in energy of less than 1 kJ mol^{-1} was 900 eV, and the point at which increasing the plane wave basis set cutoff by 100 eV led to a change in energy of less than 0.1 kJ mol^{-1} was 1000 eV. k-point spacings of 0.1, 0.05 and 0.04 Å^{-1} were tested, and the energy difference between the first two was above 3 kJ mol^{-1}, while the energy difference between the last two was below 0.1 kJ mol^{-1}. A plane wave basis set cut off of 1000 eV and a k-point spacing of 0.05 Å^{-1} was used for crystal structures of CRN.

S2.1.1.5 Isolated molecule calculations

CRN is a rigid molecule, and was optimized in GAUSSIAN with the PBE0/6-31G(d,p) level of theory. However, the molecule was perfectly planar, and hence orthogonal axes could not be defined by the atoms (required for our computational workflow). The molecule was reoptimized with minor changes to the z coordinates of a few atoms, and the resulting molecule was not completely planar.

For evaluation of the molecular energy within the periodic electronic structure method, the point at which increasing the space between molecules by 2 Å led to an energy difference of less than 0.1 kJ mol^{-1} was 14 Å, and so a box with this spacing between molecules was used for the molecule optimization for the ψ_{crys} method.

S2.1.2 Acridine

S2.1.2.1 Selected structures

Table S4. Structures selected for ACR.

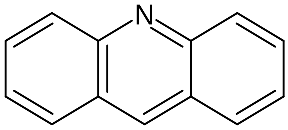

Name	Acridine
CSD REFCODE	ACRDIN
Formula	$C_{13}H_9N$
ψ_{mol} published	Schur et al.[4]
ψ_{crys} published	Schur et al.[4]

Key	Original ψ_{mol} label	Z'	Space group	mols / cell	Reason
ACR01	A48 (=ACRDIN04, 185 K, form II)	1	$P2_1/n$	4	Experimentally observed
ACR02	E3 (from ACRDIN09, 173 K, form III)	2	$P2_1/c$	8	Experimentally observed (not found in CSP – high Z')
ACR03	E4 (from ACRDIN08, RT, form IV)	3	$P2_12_12_1$	12	Experimentally observed (Sohncke space group; not found in CSP – high Z')
ACR04	E6 (from ACRDIN05, 187 K, form VI)	2	Cc	16	Experimentally observed (not found in CSP – high Z')
ACR05	E7 (from ACRDIN06, 185 K, form VII)	2	$P2_1/n$	8	Experimentally observed (not found in CSP – high Z')
ACR06	A90 (=ACRDIN12, RT, form IX)	1	$P2_1/n$	4	Experimentally observed
ACR07	A15	1	$P2_1/c$	4	Lowest energy ψ_{mol} structure

S2.1.2.2 Experimentally observed polymorphs not included in study

So called ACR I is a hydrate, containing 0.75 molecule equivalents of water (CSD refcode ZZZRLO01). ACR V is reported on the CSD as ACRDIN02, but has no 3D coordinates. ACR VIII was reported by Braga et al.[5] as a high temperature polymorph, but a full structure determination was not carried out.

S2.1.2.3 Most thermodynamically stable form at low temperature

This is uncertain as "The heat of fusion rule shows that II and III are enantiotropically related, and III and IX are enantiotropically related. However, we cannot conclude from experiment whether II or IX is the most stable low temperature form."[4] Energies are calculated relative to ACR06 (ACR IX).

S2.1.2.4 Previous CSP method and reoptimization of structures

A rigid molecule search was carried out using CrystalPredictor in 2009, using the MP2/6-31G(d,p) optimized molecule. Structures were optimized with DMACRYS 2.0.4, using multipoles derived from the MP2/6-31G(d,p) charge density and the FIT parameters to model the repulsion-dispersion interactions. The selected structures were reoptimized with DMACRYS 2.3.1.1, and the same multipoles and repulsion-dispersion potential but using splines to evaluate the lattice summations.

S2.1.2.5 Plane wave basis set cutoff and k-point spacing for crystal structure optimization

Convergence of the plane wave basis set cutoff and k-point spacing was tested for forms ACR01 and ACR06. The point at which increasing the plane wave basis set cutoff by 100 eV led to a change in energy of less than 1 kJ mol^{-1} was 800 eV for both structures. The energy difference between the two structures was consistent at all plane wave basis set cutoffs. k-point spacings of 0.1 and 0.06 Å^{-1} were tested, and the energy difference at all steps was below 1 kJ mol^{-1}. A plane wave basis set cut off of 800 eV and a k-point spacing of 0.1 Å^{-1} was used for crystal structures of ACR.

S2.1.2.6 Isolated molecule calculations

ACR is a rigid molecule, and was optimized in GAUSSIAN with the MP2/6-31G(d,p) level of theory.

For evaluation of the molecular energy within the periodic electronic structure method, the point at which increasing the space between molecules by 2 Å led to an energy difference of less than 0.1 kJ mol^{-1} was 11 Å, and so a box with this spacing between molecules was used for the molecule optimization for the ψ_{crys} method.

S2.1.3 Phthalimide

S2.1.3.1 Selected structures

Table S5. Structures selected for PTH.

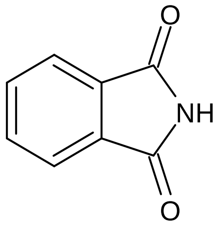

Name	Phthalimide
CSD REFCODE	PHALIM
Formula	$C_8H_5N_1O_2$
ψ_{mol} published	Unpublished
ψ_{crys} published	Unpublished

Key	Original ψ_{mol} label	Z′	Space group	mols / cell	Reason
PTH01	be46 (=PHALIM02, 120 K, form I)	2	*Pn*	4	Experimentally observed ($R_2^2(8)$ C-H···N and both $R_2^2(10)$ and $C_1^1(5)$ C-H···O graph sets)
PTH02	ai24	1	*P*2$_1$/*c*	4	Lowest energy ψ_{mol} structure ($C_1^1(4)$ C-H···N graph set)
PTH03	bb27	2	*P*2$_1$	4	Lowest energy ψ_{mol} structure in Sohncke space group ($C_1^1(4)$ C-H···N with different twist from PTH02 and $C_1^1(5)$ C-H···O graph sets)
PTH04	fc70	1	*P*2$_1$/*c*	4	Packing (different packing of same chain as experimental structure)
PTH05	am62	1	*P*2$_1$/*c*	4	Packing (different packing of same chain as experimental structure)

PTH06	ab111	1	*P*-1	2	Packing (sheet of $R_2^2(8)$ C-H···N and $R_2^2(10)$ C-H···O graph sets)
PTH07	am42	1	$P2_1/n$	4	Packing (different chain with $R_2^2(8)$ C-H···N and $R_2^2(10)$ C-H···O graph sets)
PTH08	ai84	1	$P2_1/c$	4	Packing ($C_1^1(4)$ C-H···N with same chain as PTH03, and $R_2^2(10)$ C-H···O graph sets)

S2.1.3.2 Previous CSP method
A rigid molecule search was carried out with MOLPAK in 2006, using the MP2/6-31G(d,p) optimized molecule. Structures were optimized with DMAREL 4.1.1, using multipoles derived from the MP2/6-31G(d,p) charge density and the FIT parameters to model the repulsion-dispersion interactions.

S2.1.3.3 Plane wave basis set cutoff and k-point spacing for crystal structure optimization
Convergence of the plane wave basis set cutoff and k-point spacing was tested for PTH01 and PTH02. The point at which increasing the plane wave basis set cutoff by 100 eV led to a change in absolute energy of less than 1 kJ mol^{-1} was 900 eV, and the point at which increasing the plane wave basis set cutoff by 100 eV led to a change in relative energy of less than 0.02 kJ mol^{-1} was 700 eV. k-point spacings of 0.1, 0.06 and 0.04 Å^{-1} were tested, and the energy difference at all steps was below 0.5 kJ mol^{-1}.

A plane wave basis set cut off of 900 eV and a k-point spacing of 0.1 Å^{-1} was used for crystal structures of PTH.

S2.1.3.4 Isolated molecule calculations
PTH is a rigid molecule, and was optimized in GAUSSIAN with the MP2/6-31G(d,p) level of theory. For evaluation of the molecular energy within the periodic electronic structure method, the point at which increasing the space between molecules by 2 Å led to an energy difference of less than 0.1 kJ mol^{-1} was 9 Å, and so a box with this spacing between molecules was used for the molecule optimization for the ψ_{crys} method.

S2.1.4 Saccharine

S2.1.4.1 Selected structures
Table S6. Structures selected for SAC.

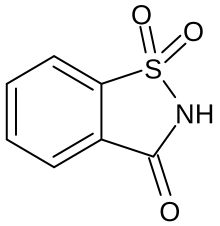

Name	Saccharine
CSD REFCODE	SCCHRN
Formula	$C_7H_5N_1O_3S$
ψ_{mol} published	Corpinot et al.[6]
ψ_{crys} published	Unpublished

Key	Original ψ_{mol} label	Z′	Space group	mols / cell	Reason
SAC01	ai26 (=SCCHRN07, 150 K, form I)	1	$P2_1/c$	4	Experimentally observed (amide dimer)
SAC02	ak47	1	$P2_1/c$	4	Lowest energy ψ_{mol} structure (antiparallel polar ribbon)
SAC03	aq4	1	$P2_12_12_1$	4	Lowest energy ψ_{mol} structure in Sohncke space group (antiparallel polar ribbon)
SAC04	ca54	1	*P*-1	2	Packing (non-polar ribbon)
SAC05	ak111	1	$P2_1/c$	4	Packing (antiparallel twisted molecule polar ribbon)
SAC06	af34	1	$P2_1$	2	Packing (parallel twisted molecule polar ribbon)
SAC07	aq139	1	$P2_12_12_1$	4	Packing (antiparallel polar sheet)
SAC08	bh33	1	$Pca2_1$	4	Packing (parallel polar sheet)
SAC09	da29	1	*Cc*	2	Packing (parallel polar ribbon)
SAC10	ab29	1	*P*-1	2	Packing (SO_2 dimer)

S2.1.4.2 Previous CSP method

A rigid molecule search was carried out in MOLPAK, using the PBE0/6-31G(d,p) optimized molecule. Structures were optimized with DMACRYS 2.0.8b, using multipoles derived from the PBE0/6-31G(d,p) charge density and the FIT parameters with Scheraga's sulfoxide parameters[7] to model the repulsion-dispersion interactions.

S2.1.4.3 Plane wave basis set cutoff and k-point spacing for crystal structure optimization

Convergence of the plane wave basis set cutoff and k-point spacing was tested for SAC01 and SAC04. The point at which increasing the plane wave basis set cutoff by 100 eV led to a change in absolute energy of less than 1 kJ mol^{-1} was 900 eV, and the point at which increasing the plane wave basis set cutoff by 100 eV led to a change in relative energy of less than 0.05 kJ mol^{-1} was 700 eV. k-point spacings of 0.1 and 0.06 Å^{-1} were tested, and the energy difference at all steps was below 0.1 kJ mol^{-1}. A plane wave basis set cut off of 900 eV and a k-point spacing of 0.1 Å^{-1} was used for crystal structures of SAC.

S2.1.4.4 Isolated molecule calculations

SAC is a rigid molecule, and was optimized in GAUSSIAN with the PBE0/6-31G(d,p) level of theory.

For evaluation of the molecular energy within the periodic electronic structure method, the point at which increasing the space between molecules by 2 Å led to an energy difference of less than 0.1 kJ mol^{-1} was 11 Å, and so a box with this spacing between molecules was used for the molecule optimization for the ψ_{crys} method.

S2.1.5 5-fluorouracil

S2.1.5.1 Selected structures

Table S7. Structures selected for FLU.

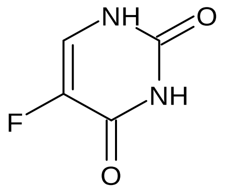

Name	5-fluorouracil
CSD REFCODE	FURACL
Formula	$C_4H_3N_2O_2F$
ψ_{mol} published	Hulme et al.[8]
ψ_{crys} published	Unpublished

Key	Original ψ_{mol} label	Z′	Space group	mols / cell	Reason
FLU01	eo1 (=FURACL01, 150 K, form I)	4	*P*-1	8	Experimentally observed (not found in CSP – high Z′)
FLU02	am75 (=FURACL03, 150 K, form II)	1	$P2_1/n$	4	Experimentally observed, lowest energy ψ_{mol} structure, asymmetric ribbon
FLU03	ak24	1	$P2_1/c$	4	Packing, non-polar symmetric ribbon
FLU04	am64	1	$P2_1/c$	4	Packing, dimers
FLU05	fc19	1	$P2_1/c$	4	Packing, antiparallel polar symmetric ribbon
FLU06	aq90	1	$P2_12_12_1$	4	Lowest energy ψ_{mol} structure in Sohncke space group, antiparallel polar symmetric ribbon

S2.1.5.2 Most thermodynamically stable form at low temperature

"Thermal analysis suggests a monotropic relationship with Form I being more stable."[8]

Energies are calculated relative to FLU01 (FLU I).

S2.1.5.3 Previous CSP method

A rigid molecule search was carried out in MOLPAK, using the MP2/6-31G(d,p) optimized molecule. Structures were optimized with DMAREL 3.0, using multipoles derived from the MP2/6-31G(d,p) charge density and the FIT parameters to model the repulsion-dispersion interactions.

S2.1.5.4 Plane wave basis set cutoff and k-point spacing for crystal structure optimization

Convergence of the plane wave basis set cutoff and k-point spacing was tested for structures FLU02 and FLU03. The point at which increasing the plane wave basis set cutoff by 100 eV led to a change in energy of less than 1 kJ mol^{-1}

was 800 eV for FLU02 and 900 eV for FLU03. The point at which increasing the plane wave basis set cutoff by 100 eV led to a change in relative energy of less than 1 kJ mol^{-1} was also 900 eV. k-point spacings of 0.1, 0.05 and 0.04 Å^{-1} were tested, and the energy difference for all k-point spacings was below 1 kJ mol^{-1} for FLU03, while it was only below 1 kJ mol^{-1} from 0.05 Å^{-1} for FLU02.

A plane wave basis set cut off of 900 eV and a k-point spacing of 0.1 Å^{-1} was used for crystal structures FLU

S2.1.5.5 Isolated molecule calculations

FLU is a rigid molecule, and was optimized in GAUSSIAN with the MP2/6-31G(d,p) level of theory.

For evaluation of the molecular energy within the periodic electronic structure method, the point at which increasing the space between molecules by 2 Å led to an energy difference of less than 0.1 kJ mol^{-1} was 11 Å, and so a box with this spacing between molecules was used for the molecule optimization for the ψ_{crys} method.

S2.2 Carbamazepine family

All the experimentally observed crystal structures and virtually all the CSP-generated crystal structures of this family of molecules exhibit either $C_1^1(4)$ (chain) or $R_2^2(8)$ (dimer) hydrogen bonding of the amide group. Hence all the crystal structures of this family have been classified by one of these two motifs.

Throughout, green is used to denote structures containing dimer interactions and orange is used to denote structures containing chain interactions.

S2.2.1 Isostructurality

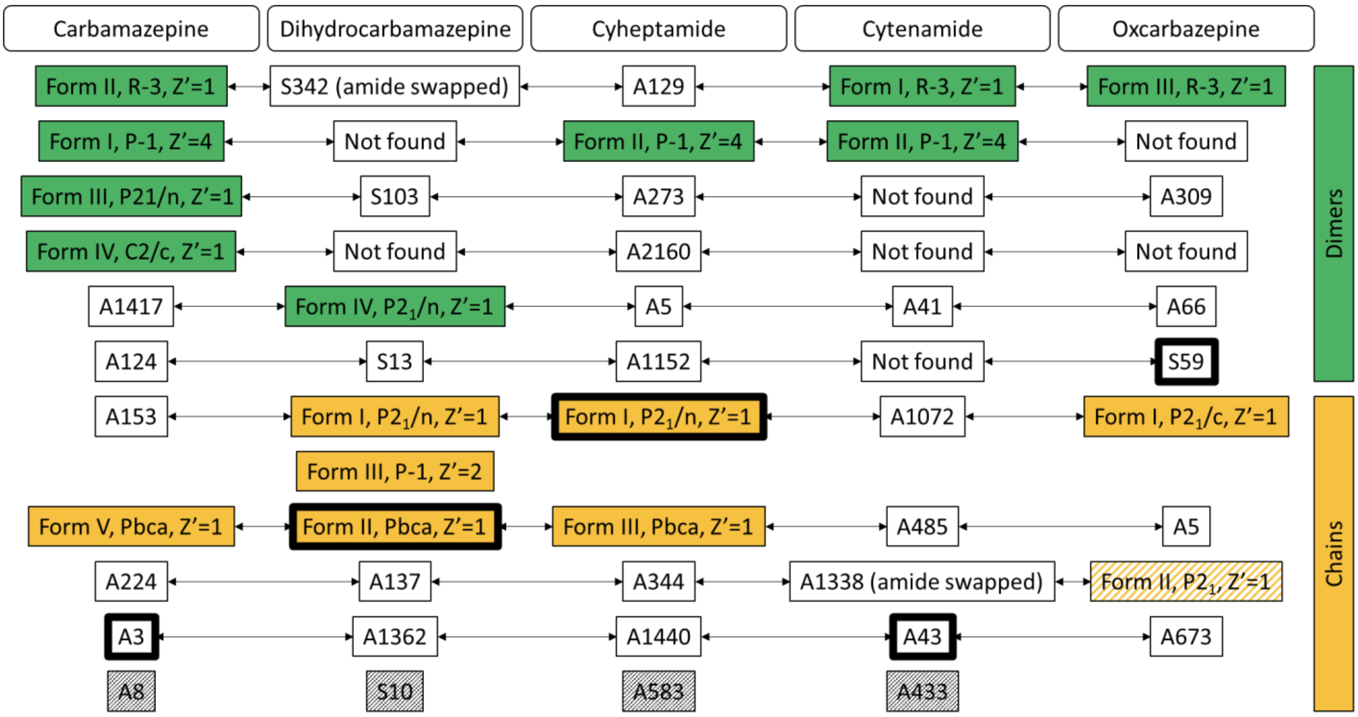

Figure S2. Isostructural relationships between carbamazepine and its analogues. Experimentally observed forms are colored green ($R_2^2(8)$ hydrogen bonded dimers) or orange ($C_1^1(4)$ hydrogen bonded chains); the lowest energy structure on the ψ_{mol} lattice energy landscape has a thick black box; the lowest energy chiral structure on that same landscape has a shaded box.

In their 2011 publication, Arlin et al calculated the relative energies for each of the as yet known crystal packings with each of the molecules CBZ, CYT, CYH, and DHC with the ψ_{mol} method.[9]

S2.2.2 Selected structures

Table S8. Structures selected for the carbamazepine family of molecules. Structures based on the dimer are shaded green, while those based on the chain are shaded orange.

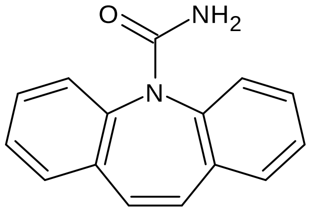

Name	Carbamazepine
CSD REFCODE	CBMZPN
Formula	$C_{15}H_{12}N_2O$
ψ_{mol} published	Unpublished
ψ_{crys} published	Unpublished

Key	Original ψ_{mol} label	Z′	Space group	mols / cell	Reason
CBZ01	E1 (from CBMZPN11, 158 K, form I)	4	*P*-1	8	Experimentally observed (not found in CSP – high Z′)
CBZ02	A193 (=CBMZPN03, RT, form II)	1	*R*-3	18	Experimentally observed
CBZ03	A1 (=CBMZPN10, RT, form III)	1	$P2_1/n$	4	Experimentally observed
CBZ04	A54 (=CBMZPN12, 158 K, form IV)	1	*C*2/*c*	8	Experimentally observed
CBZ05	A145 (=CBMZPN16, 123 K, form V)	1	*Pbca*	8	Experimentally observed
CBZ06	A3	1	$P2_1/c$	4	Lowest energy ψ_{mol} structure
CBZ07	A8	1	*P*21212	4	Lowest energy chiral ψ_{mol} structure
CBZ08	A153	1	$P2_1/n$	4	Isostructural with CYH I
CBZ09	A1417	1	$P2_1/c$	4	Isostructural with DHC IV

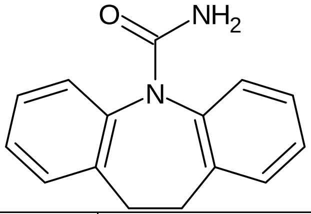

Name	Dihydrocarbamazepine
CSD REFCODE	VACTAU
Formula	$C_{15}H_{14}N_2O$
ψ_{mol} published	Unpublished
ψ_{crys} published	Unpublished

Key	Original ψ_{mol} label	Z′	Space group	mols / cell	Reason
DHC01	A3 (=VACTAU01, RT, form I)	1	$P2_1/n$	4	Experimentally observed
DHC02	A2 (=VACTAU02, 120 K, form II)	1	*Pbca*	8	Experimentally observed; lowest energy ψ_{mol} structure
DHC03	E3 (from VACTAU03, 150 K, form III)	2	*P*-1	4	Experimentally observed (not found in CSP – high Z′)
DHC04	S16 (=VACTAU04, 123 K, form IV)	1	$P2_1/n$	4	Experimentally observed
DHC05	S342	1	*R*-3	18	Same packing as CBZ II and CYT I, but opposite amide configuration
DHC06	S103	1	$P2_1/n$	4	Isostructural with CBZ III
DHC07	A1362	1	$P2_1/c$	4	Isostructural with CBZ06
DHC08	S10	1	$P2_1$	2	Lowest energy chiral ψ_{mol} structure

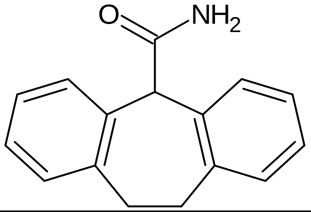

Name	Cyheptamide
CSD REFCODE	TEVSOD
Formula	$C_{16}H_{15}NO$
ψ_{mol} published	Srirambhatla et al.[10]
ψ_{crys} published	Srirambhatla et al.[10]

Key	Original ψ_{mol} label	Z′	Space group	mols / cell	Reason
CYH01	A621 (=TEVSOD, 150 K, form I)	1	$P2_1/n$	4	Experimentally observed; lowest energy ψ_{mol} structure

CYH02	E2 (from TEVSOD01, 100 K, form II)	4	*P*-1	8	Experimentally observed (not found in CSP – high Z′)
CYH03	A16 (=TEVSOD02, 123 K, form III)	1	*Pbca*	8	Experimentally observed
CYH04	A583	1	$P2_12_12_i$	4	Lowest energy chiral ψ_{mol} structure
CYH05	A5	1	$P2_1/n$	4	Isostructural with DHC IV
CYH06	A1440	1	$P2_1/c$	4	Isostructural with CBZ06
CYH07	A273	1	$P2_1/n$	4	Isostructural with CBZ III
CYH08	A129	1	*R*-3	18	Isostructural with CBZ II
CYH09	A2160	1	$C2/c$	8	Isostructural with CBZ IV

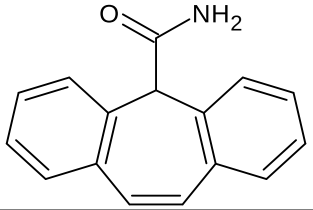

Name	Cytenamide
CSD REFCODE	SODNOP, SOGLEG
Formula	$C_{16}H_{13}NO$
ψ_{mol} published	Unpublished
ψ_{crys} published	Unpublished

Key	Original ψ_{mol} label	Z′	Space group	mols / cell	Reason
CYT01	A12 (=SOGLEG, 120 K, form I)	1	*R*-3	18	Experimentally observed
CYT02	E2 (from SODNOP, RT, form II)	4	*P*-1	4	Experimentally observed (not found in CSP – high Z′)
CYT03	A43	1	$P2_1/a$	4	Lowest energy ψ_{mol} structure
CYT04	A433	1	$P2_1$	2	Lowest energy chiral ψ_{mol} structure
CYT05	A1072	1	$P2_1/c$	4	Chain contact
CYT06	A485	1	*Pbca*	8	Chain contact
CYT07	A41	1	$P2_1/c$	4	Isostructural with DHC IV

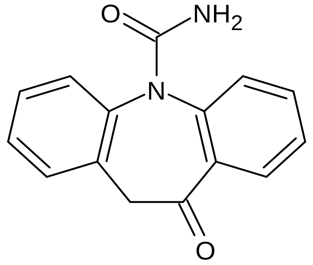

Name	Oxcarbazepine
CSD REFCODE	CANDUR
Formula	$C_{15}H_{12}N_2O_2$
ψ_{mol} published	Polyzois et al.[11]
ψ_{crys} published	Polyzois et al.[11]

Key	Original ψ_{mol} label	Z′	Space group	mols / cell	Reason
OXC01	A2 (=CANDUR01, 95 K, form I)	1	$P2_1/c$	4	Experimentally observed
OXC02	A20 (=CANDUR02, 95 K, form II)	1	$P2_1$	2	Experimentally observed; lowest energy chiral ψ_{mol} structure
OXC03	A165 (=CANDUR03, RT, form III)	1	*R*-3	18	Experimentally observed
OXC04	S59	1	*P*-1	2	Lowest energy ψ_{mol} structure
OXC05	A309	1	$P2_1/c$	4	Isostructural with CBZ III
OXC06	A5	1	*Pbca*	8	Isostructural with CBZ V
OXC07	A673	1	$P2_1/c$	4	Isostructural with CBZ06
OXC08	A66	1	$P2_1/c$	4	Isostructural with DHC IV

S2.2.3 $R\overline{3}$ packings

Some of the experimentally observed crystal structures in $R\overline{3}$ have include a small non-stoichiometric number of solvent molecules,[12] as discussed in the manuscript. The phase pure ordered models for these crystals were partially optimized with CASTEP, but took a very long time, and the decision was made to reduce the convergence criteria to

$|F|_{max} < 1*10^{-2}$ (rather than $1*10^{-3}$). For the partially optimized structures that were below this new threshold when the decision to change was made, a single point calculation was carried out (in order to write out the partially optimized unit cell), but this included the energy correction for the finite basis set. Optimization calculations in CASTEP do not have the energies corrected for the finite basis set, while single point energy calculations do. The difference is typically of the order of $1*10^{-4}$ kJ mol^{-1}and so was ignored.

S2.2.4 Most thermodynamically stable form at low temperature

Stabilities were calculated relative to:

- CBZ03 (CBZ III). Low temperature form. Crystallized at room temperature, and converts to CBZ I on heating to 150 °C.[13]
- DHC02 (DHC II). Form II is the most stable computationally.[14]
- CYH01 (CYH I). This was the form purchased from the manufacturer, which then transformed to CYH II on heating to 441.6 K.[15]
- CYT02 (CYT II). CYT I has included solvent, so cannot be considered a true polymorph.[16] However, transformation of CYT I to CYT II occurs at 498 K, suggesting that CYT II is the more stable at high temperatures, but not indicating which is the more stable at low temperatures.[16]
- OXC01 (OXC I). OXC II converts to OXC I when heated to 118-150 °C,[17] with the enthalpy changes implying that OXC I is more stable.

S2.2.5 Previous CSP method

Flexible molecule searches were carried out in CrystalPredictor 2.1.01. The resulting crystal structures were optimized with CrystalOptimizer 2.4 using DMACRYS 2.2.01 as the lattice energy minimizing program, with intramolecular energy being evaluated at the PBE0/6-31G(d,p) level of theory, multipoles extracted from the charge density calculated at the same level of theory, and repulsion-dispersion parameters taken from the FIT potential.

S2.2.6 Plane wave basis set cutoff and k-point spacing for crystal structure optimization

Convergence of the plane wave basis set cutoff and k-point spacing was tested for CBZ06 and CYH01. The point at which increasing the plane wave basis set cutoff by 100 eV led to a change in energy of less than 1 kJ mol^{-1} was 700 eV for CBZ06 and 900 eV for CYH01, and the point at which increasing the plane wave basis set cutoff by 100 eV led to a change in energy of less than 0.2 kJ mol^{-1} was 1100 eV for both structures. k-point spacings of 0.1, 0.05 and 0.04 Å^{-1} were tested, and the energy difference at all steps between the first two was below 1 kJ mol^{-1}, but 0.05 Å^{-1} was chosen as the difference between using 0.05 and 0.04 Å^{-1} was below 0.003 kJ mol^{-1} for both crystal structures. A plane wave basis set cut off of 1100 eV and a k-point spacing of 0.05 Å^{-1} was used for crystal structures of all five molecules, including the large unit cells of the $R\overline{3}$ structures.

S2.2.7 Isolated molecule calculations

This family has an NH_2 group, where there is a low barrier to inversion in the gas phase, but the hydrogen bonding in the crystal structure may define the proton positions. Hence, optimizations started from both configurations. “In” denotes that the pyramidality of the NH_2 group points the hydrogen atoms towards the benzodiazepine group, and “Out” denotes that the pyramidality of the NH_2 group points the hydrogen atoms away from the benzodiazepine group.

For CYH and DHC, “Syn” denotes that the CH_2 that is closer to the amide is on the side of the carbonyl group, and “Anti” denotes that the CH_2 that is closer to the amide is on the side of the NH_2 group. For OXC, “Syn” denotes that the ring carbonyl is on the side of the carbonyl group, and “Anti” denotes that the ring carbonyl is on the side of the NH_2 group.

All possible conformations were optimized with GAUSSIAN at the PBE/6-31G(d,p) level of theory.

For evaluation of the molecular energy within the periodic electronic structure method, the point at which increasing the space between molecules by 2 Å led to an energy difference of less than 0.1 kJ mol^{-1} was 7 Å for CBZ_In, 6 Å for CYH_Syn_In, 8 Å for CYT_In, 9 Å for DHC_Anti_In and 10 Å for OXC_Anti_In. Boxes with 10 Å between molecules were used for the molecule optimizations for the ψ_{crys} method.

S2.3 Fenamate family

Virtually all crystal structures in all the energy landscapes of these five molecules contain the carboxylic acid group in the $R_2^2(8)$ centrosymmetric configuration. The only crystal structures we consider without this hydrogen bonding are the lowest energy structures which are chiral (or in a Sohncke space group when the molecule is symmetrical), which all contain a $C_1^1(4)$ hydrogen bonding motif.

For this family of molecules, all possible isostructurally related crystals were considered. If a particular experimentally observed packing was not found in the CSP of a certain molecule, then a crystal structure was constructed by editing the molecule in the CSD structure, and using that as the starting point for the ψ_{mol} optimization. Throughout, structures with the prefix "A" were found in the CSP, those with the prefix "E" had ψ_{mol} optimizations started directly from the CSD structure, and those with prefixes "Q," "M," "T," "F," or "N" were derived from FEA, MFA, TFA, FFA or NFA CSD structures by editing the molecule. ("Q" was chosen as the FEA REFCODE is QQQBTY.) The criteria used for isostructurality, namely 60 % distance and 60° angle tolerances as well as allowing molecule differences, do allow two structures of the same molecule to be included in the set that are isostructural to the same structure of another molecule, c.f. FEA I and TFA VIII.

S2.3.1 Isostructurality

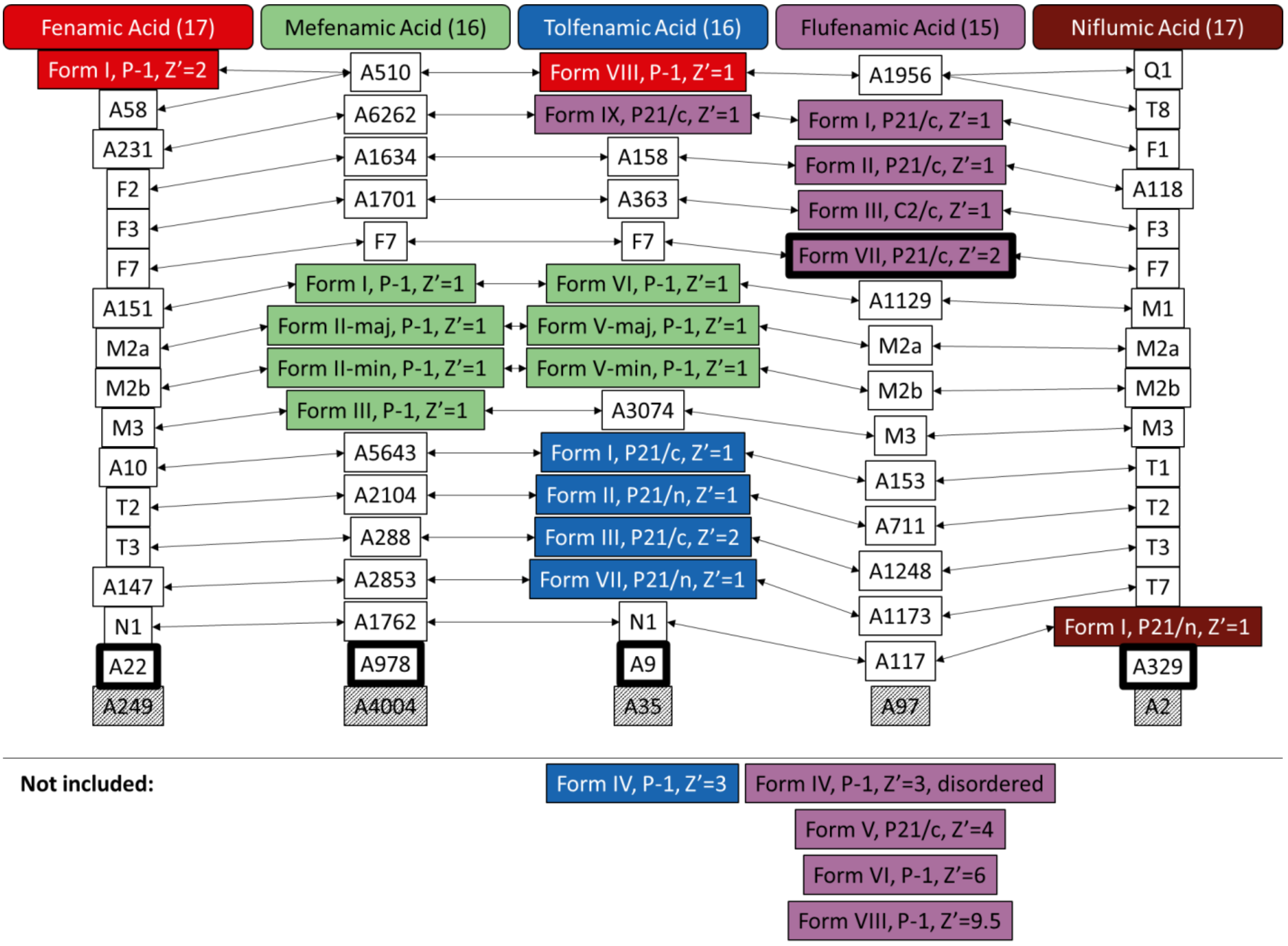

Figure S3. The isostructural relationships within the fenamate family of molecules. Experimental structures are classified by the molecule color of the experimentally observed packing (FEA=red, MFA=green, TFA=blue, FFA=lilac, NFA=brown), except when there is an isostructural relationship, in which case the color is that of the molecule which was the earliest deposited on the CSD to exhibit that packing. Some experimentally observed crystal structures with high numbers of molecules are excluded from the study. The lowest energy structure on the original CrystalOptimizer energy landscapes is outlined with a thick black line, and the lowest energy chiral structure is marked with diagonal stripes. Isostructural relationships of these two groups of structures are not included. Note that FEA I is Z'=2 with distinct conformations but the packings in FEA I and A58 are similar apart from the twist of a phenyl ring. Both the Z'=1 and Z'=2 models were constructed for NFA.

S2.3.2 Selected structures

Table S9. Structures selected for the fenamate family of molecules. Rows are colored by the molecular color of the experimentally observed packing (FEA=red, MFA=green, TFA=blue, FFA=lilac, NFA=brown), except when there is an isostructural relationship, in which case the color is that of the molecule which was the earliest deposited on the CSD to exhibit that packing. Uncolored rows are as yet unobserved packings.

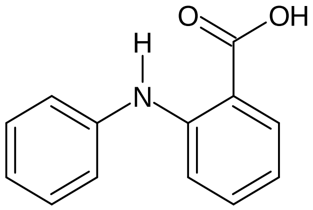

Name	Fenamic Acid
CSD REFCODE	QQQBTY
Formula	$C_{13}H_{11}N_1O_2$
ψ_{mol} published	Uzoh et al.[18]
ψ_{crys} published	Unpublished

Key	Original ψ_{mol} label	Z′	Space group	mols / cell	Reason
FEA01	E1 (from QQQBTY02, RT, form I)	2	*P*-1	4	Experimentally observed (not found in CSP – high Z′)
FEA02	A58	1	*P*-1	2	Close match to FEA I; Isostructural with TFA VIII
FEA03	A22	1	$P2_1/c$	4	Lowest energy ψ_{mol} structure
FEA04	A249	1	$P2_1$	2	Lowest energy chiral ψ_{mol} structure
FEA05	A231	1	$P2_1/c$	4	Isostructural with FFA I and TFA IX
FEA06	F2 (from FPAMCA17)	1	$P2_1/c$	4	Constructed from FFA II, (not found in CSP)
FEA07	F3 (from FPAMCA19)	1	$C2/c$	8	Constructed from FFA III (not found in CSP)
FEA08	F7 (from FPAMCA20)	2	$P2_1/c$	8	Constructed from FFA VII (not found in CSP – high Z′)
FEA09	A151	1	*P*-1	2	Isostructural with MFA I and TFA VI
FEA10	M2a (from XYANAC05-maj)	1	*P*-1	2	Constructed from major component of MFA II (isostructural with TFA V; not found in CSP)
FEA11	M2b (from XYANAC05-min)	1	*P*-1	2	Constructed from minor component of MFA II (isostructural with TFA V; not found in CSP)
FEA12	M3 (from XYANAC03)	1	*P*-1	2	Constructed from MFA III (not found in CSP)
FEA13	A10	1	$P2_1/c$	4	Isostructural with TFA I
FEA14	T2 (from KAXXAI)	1	$P2_1/n$	4	Constructed from TFA II (not found in CSP)
FEA15	T3 (from KAXXAI02)	1	$C2/c$	8	Constructed from TFA III (not found in CSP)
FEA16	A147	1	$P2_1/c$	4	Isostructural with TFA VII
FEA17	N1 (from NIFLUM11)	1	$P2_1/n$	4	Constructed from NFA I (not found in CSP)

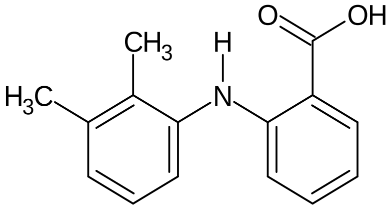

Name	Mefenamic Acid
CSD REFCODE	XYANAC
Formula	$C_{15}H_{15}N_1O_2$
ψ_{mol} published	Case et al.[19]
ψ_{crys} published	Case et al.[19]

Key	Original ψ_{mol} label	Z′	Space group	mols / cell	Reason
MFA01	A128 (=XYANAC06, 100 K, form I)	1	*P*-1	2	Experimentally observed
MFA02	A889 (=XYANAC05-maj, 100 K, form II)	1	*P*-1	2	Experimentally observed
MFA03	A497 (=XYANAC05-min, 100 K, form II)	1	*P*-1	2	Experimentally observed
Although XYANAC05 does not specify the ratio of the disorder components, the major and minor components were determined in XYANAC02, which specifies a 70:30 ratio, and XYANAC04, which specifies a 55:45 ratio of the major and minor components.					
MFA04	A1666 (=XYANAC03, RT, form III)	1	*P*-1	2	Experimentally observed
MFA05	A978	1	*P*-1	2	Lowest energy ψ_{mol} structure
MFA06	A4004	1	$P2_1$	2	Lowest energy chiral ψ_{mol} structure
MFA07	A510	1	*P*-1	2	Isostructural with FEA I and TFA VIII

MFA08	A6262	1	$P2_1/c$	4	Isostructural with FFA I and TFA IX
MFA09	A1634	1	$P2_1/c$	4	Isostructural with FFA II
MFA10	A1701	1	$P2_1/c$	4	Close match to FFA III
MFA11	F7 (from FPAMCA20)	2	$P2_1/c$	8	Constructed from FFA VII (not found in CSP – high Z′)
MFA12	A5643	2	$P2_1$ (not chiral)	4	Isostructural with TFA I
MFA13	A2104	1	$P2_1/c$	4	Isostructural with TFA II
MFA14	A288	1	$C2/c$	8	Isostructural with TFA III
MFA15	A2853	2	$P2_1$ (not chiral)	4	Isostructural with TFA VII
MFA16	A1762	1	$P2_1/c$	4	Isostructural with NFA I

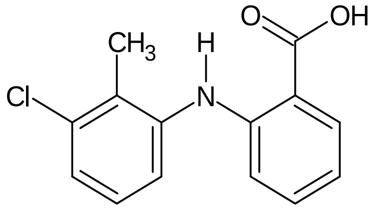

Name	Tolfenamic Acid
CSD REFCODE	KAXXAI
Formula	$C_{14}H_{12}N_1O_2Cl$
ψ_{mol} published	Case et al.[19]
ψ_{crys} published	Case et al.[19]

Key	Original ψ_{mol} label	Z′	Space group	mols / cell	Reason
TFA01	A4121 (=KAXXAI01, 110 K, form I)	2	$P2_1$ (not chiral)	4	Experimentally observed
TFA02	A283 (=KAXXAI, 110 K, form II)	1	$P2_1/n$	4	Experimentally observed
TFA03	A93 (=KAXXAI02, 85 K, form III)	1	$C2/c$	8	Experimentally observed
TFA04	A7232 (=KAXXAI04-maj, 85 K, form V)	1	P-1	2	Experimentally observed
TFA05	A646 (=KAXXAI04-min, 85 K, form V)	1	P-1	2	Poor match to experimentally observed
The two components have equal occupancies, so the maj and min terms are misnomers in this case					
TFA06	A917 (=KAXXAI07-maj, 151 K, form VI)	1	P-1	2	Experimentally observed
TFA07	A4809 (=KAXXAI05, RT, form VII)	1	$P2_1/n$	4	Experimentally observed
TFA08	A2 (=KAXXAI06, 120 K, form VIII)	1	P-1	2	Experimentally observed
TFA09	A5102 (=KAXXAI11, RT, form IX)	1	$P2_1/c$	4	Experimentally observed
TFA10	A9	1	$P2_1/c$	4	Lowest energy ψ_{mol} structure
TFA11	A35	1	$P2_12_12_1$	4	Lowest energy chiral ψ_{mol} structure
TFA12	A158	1	$P2_1/c$	4	Isostructural with FFA I
TFA13	A363	1	$P2_1/c$	4	Close match to FFA III
TFA14	F7 (from FPAMCA20)	2	$P2_1/c$	8	Constructed from FFA VII (not found in CSP – high Z′)
TFA15	A3074	1	P-1	2	Close match to MFA III
TFA16	N1 (from NIFLUM11)	1	$P2_1/n$	4	Constructed from NFA I (not found in CSP)
TFA17	E4 (from KAXXAI03, 85 K, form IV)	3	P-1	6	Experimentally observed (not found in CSP – high Z′)

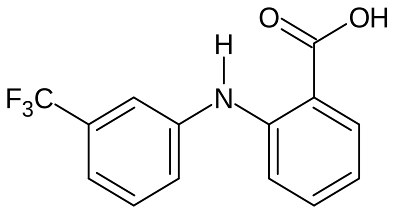

Name	Flufenamic Acid
CSD REFCODE	FPAMCA
Formula	$C_{14}H_{10}F_3N_1O_2$
ψ_{mol} published	Case et al.[19]
ψ_{crys} published	Case et al.[19]

Key	Original ψ_{mol} label	Z′	Space group	mols / cell	Reason
FFA01	A560 (=FPAMCA18, 90 K, form I)	1	$P2_1/c$	4	Experimentally observed
FFA02	A748 (=FPAMCA17, 95 K, form II)	1	$P2_1/c$	4	Experimentally observed; lowest energy ψ_{mol} structure
FFA03	E3 (from FPAMCA19, 90 K, form III)	1	$C2/c$	8	Experimentally observed (not found in CSP)
FFA04	E7 (from FPAMCA20, 100 K, form VII)	2	$P2_1/c$	8	Experimentally observed (not found in CSP – high Z′)
FFA05	A97	1	$P2_12_12_1$	4	Lowest energy chiral ψ_{mol} structure
FFA06	A1956	1	*P*-1	2	Isostructural with FEA I and TFA VIII
FFA07	A1129	1	*P*-1	2	Isostructural with MFA I and TFA VI
FFA08	M2a (from XYANAC05-maj)	1	*P*-1	2	Constructed from major component of MFA II (isostructural with TFA V; not found in CSP)
FFA09	M2b (from XYANAC05-min)	1	*P*-1	2	Constructed from minor component of MFA II (isostructural with TFA V; not found in CSP)
FFA10	M3 (from XYANAC03)	1	*P*-1	2	Constructed from MFA III (not found in CSP)
FFA11	A153	1	$P2_1/c$	4	Isostructural with TFA I
FFA12	A711	1	$P2_1/n$	4	Isostructural with TFA II
FFA13	A1248	1	$C2/c$	8	Isostructural with TFA III
FFA14	A1173	1	$P2_1/c$	1	Isostructural with TFA VII
FFA15	A117	1	$P2_1/n$	4	Isostructural with NFA
FFA16	E4 (from FPAMCA15, 273 K, form IV)	3	*P*-1	6	Experimentally observed (not found in CSP – high Z′)
FFA17	E5 (from FPAMCA16, 95 K, form V)	4	$P2_1/c$	16	Experimentally observed (not found in CSP – high Z′)
FFA18	E6 (from FPAMCA14, 85 K, form VI)	6	*P*-1	12	Experimentally observed (not found in CSP – high Z′

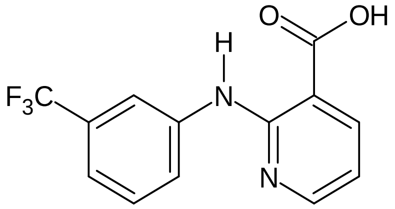

Name	Niflumic Acid
CSD REFCODE	NIFLUM
Formula	$C_{13}H_9N_2O_2F_3$
ψ_{mol} published	Unpublished
ψ_{crys} published	Unpublished

Key	Original ψ_{mol} label	Z′	Space group	mols / cell	Reason
NFA01	A1470 (=NIFLUM11, RT, form I)	1	$P2_1/n$	4	Experimentally observed
NFA02	A329	1	$P2_1/c$	4	Lowest energy ψ_{mol} structure
NFA03	A2	1	$P2_12_12_1$	4	Lowest energy chiral ψ_{mol} structure
NFA04	Q1 (from QQQBTY02)	2	P-1	4	Constructed from FEA I* (not found in CSP, high Z′)
NFA05	F1 (from FPAMCA18)	1	$P2_1/c$	4	Constructed from FFA I (not found in CSP)
NFA06	A118	1	$P2_1/c$	4	Isostructural with FFA II
NFA07	F3 (from FPAMCA19)	1	$C2/c$	8	Constructed from FFA III (not found in CSP)
NFA08	F7 (from FPAMCA20)	2	$P2_1/c$	8	Constructed from FFA VII (not found in CSP)
NFA09	M1 (from XYANAC06)	1	*P*-1	2	Constructed from MFA I (not found in CSP)

NFA10	M2a (from XYANAC05-maj)	1	*P*-1	2	Constructed from major component of MFA II (isostructural with TFA V; not found in CSP)
NFA11	M2b (from XYANAC05-min)	1	*P*-1	2	Constructed from minor component of MFA II (isostructural with TFA V; not found in CSP)
NFA12	M3 (from XYANAC03)	1	*P*-1	2	Constructed from MFA III (not found in CSP)
NFA13	T1 (from KAXXAI01)	1	$P2_1/c$	4	Constructed from TFA I (not found in CSP)
NFA14	T2 (from KAXXAI)	1	$P2_1/n$	4	Constructed from TFA II (not found in CSP)
NFA15	T3 (from KAXXAI02)	2	$P2_1/c$	8	Constructed from TFA III (not found in CSP)
NFA16	T7 (from KAXXAI05)	1	$P2_1/n$	4	Constructed from TFA VII (not found in CSP)
NFA17	T8 (from KAXXAI06)	1	*P*-1	2	Constructed from TFA VIII (not found in CSP)

* The phenyl ring of NFA is not symmetrical, so there exist two different configurations of substituting it into the FEA I crystal structure, and hence four different crystal structures possible. All were optimized with CrystalOptimizer, and the lowest energy (NFA04) was retained and optimized with CASTEP.

S2.3.3 Experimentally observed polymorphs not included in study

TFA IV is a Z′=3 structure (KAXXAI03, 85 K). FFA IV is also Z′=3 (FPAMCA15, 273 K), with disorder. It was assumed that the lower symmetry of these structures would make them intractable computationally, particularly in light of our experience with the high symmetry structures of the carbamazepine family, and hence they were not included. FFA V (FPAMCA16, 95 K) and FFA VI (FPAMCA14, 85 K) were also high Z′ or high symmetry structures, with 16 and 12 molecules in the unit cells. These could not have been optimized with CASTEP, and so were not included. FFA VIII (FPAMCA13, 85 K, Z′=9.5) had a whole molecule disorder, which is reported on the CSD as a molecule-sized void space across a symmetry element. This could not even be used as a starting point for a ψ_{mol} optimization.

There is another polymorph of NFA that has not been structurally characterized, but has a melting point of 203.6 °C which is slightly lower than that of NFA I at 204.7 °C.[20]

S2.3.4 Most thermodynamically stable form at low temperature

Stabilities were calculated relative to:

- MFA01 (MFA I). "Form I is the most stable form under ambient conditions, Form II is the stable form above 160 °C and Form III is the least stable form at ambient conditions and converts back to Form I immediately."[21]
- TFA02 (TFA II). "At low temperature, the order of stability is II > IX > I. Due to the more favourable entropic contributions of form I, the stability order at room temperature reverses to I > II > IX."[22]
- FFA03 (FFA III). Of the two "well-established" polymorphs (FFA I and FFA III), it has recently been confirmed[23] that FFA III is the stable structure at low temperatures, enantiotropically related to FFA I. This work suggests that it is very likely that FFA I and FFA II are in a monotropic relationship.

S2.3.5 Previous CSP method and reoptimization of structures

Flexible molecule searches were carried out in CrystalPredictor 1.6. The resulting crystal structures were optimized with CrystalOptimizer 2.2 or above using DMACRYS (versions 2.0.4, 2.0.8 or 2.2.1.0) as the lattice energy minimizing program, with intramolecular energy being evaluated at the PBE0/6-31G+(d) level of theory, multipoles extracted from the charge density calculated at the same level of theory, and repulsion-dispersion parameters taken from the FIT potential.

Because of the variation in versions of DMACRYS and different scientists selecting different degrees of freedom to optimize with CrystalOptimizer, all previous crystal structures were reoptimized, along with the new structures derived from substituting one molecule in the family into a crystal packing observed for another molecule in the family. The consistent optimization methodology used CrystalOptimizer 2.4.7 with DMACRYS 2.3.1.1 as the lattice energy minimizing program, and other parameters as before. The independent degrees of freedom optimized within CrystalOptimizer were torsion angles O1_C7_C1_C2, H12_O1_C7_C1, C1_C2_N1_C8, C2_N1_C8_C13 and H11_N1_C8_C13, and bond angles H11_N1_C8 and H12_O1_C7 using the numbering in Figure S4. Additionally, a single angle was chosen to account for the methyl and CF_3 groups. Derivatives of the multipole moments were used

in the optimization for all degrees of freedom apart from methyl group rotations to increase the rate at which the optimizations converged.

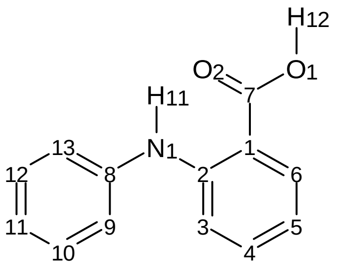

Figure S4. Atomic numbering used to define the independent degrees of freedom optimized with CrystalOptimizer for the fenamate family of molecules.

S2.3.6 Plane wave basis set cutoff and k-point spacing for crystal structure optimization

Convergence of the plane wave basis set cutoff and k-point spacing was tested for FEA01, FEA13, MFA01, TFA01, FFA01, NFA01 and NFA11. The point at which increasing the plane wave basis set cutoff by 100 eV led to a change in energy of less than 1 kJ mol^{-1} was 900 eV for both FEA and both NFA structures, 1000 eV for FFA01, and 700 eV for MFA01 and TFA01, and the point at which increasing the plane wave basis set cutoff by 100 eV led to a change in energy of less than 0.2 kJ mol^{-1} was 1000 eV for FEA and 1100 eV for all other structures. k-point spacings of 0.1, 0.06 and 0.04 Å^{-1} were tested, and the energy difference at all steps between the first two was below 1 kJ mol^{-1}.

A plane wave basis set cut off of 1000 eV was used for crystal structures of FFA, a plane wave basis set cut off of 700 eV was used for MFA and TFA, and a plane wave basis set cutoff of 900 eV was used for FEA and NFA. A k-point spacing of 0.1 Å^{-1} was used for all crystal structures of all five molecules.

S2.3.7 Isolated molecule calculations

Apart from FEA, all molecules have two different conformations due to the substituents on the ring without the carboxylic acid group. "A" denotes that the substituents are nearer to the amine and acid, and "B" denotes that they are further away.

All possible conformations were optimized with GAUSSIAN at the PBE/6-31G+(d) level of theory.

For evaluation of the molecular energy within the periodic electronic structure method, the point at which increasing the space between molecules by 2 Å led to an energy difference of less than 0.1 kJ mol^{-1} was 5 Å for FEA, 6 Å for MFA_A, 7 Å for TFA_A, 9 Å for FFA_B and 8 Å for NFA_B. Boxes with these spacings between molecules were used for the molecule optimizations for the ψ_{crys} method.

S2.4 Small drug molecules

S2.4.1 Chalcone

S2.4.1.1 Structure selection

The chalcone molecule was studied as part of a series of substituted chalcones in a structural diversity investigation.[24] The structural contacts identified in that work were used to select crystal structures to encompass all commonly observed dimer interactions in the CSP work. The reader is referred to that paper for a full explanation of the labels used for the different dimer interactions.

Table S10. Structures selected for CHA. Structures with the planar conformation are shaded lilac, those with the twisted conformation are shaded orange, those with the Region B conformation are shaded blue, and a structure with a conformation intermediate between planar and twisted is unshaded. The contacts (as identified in the packing preferences paper[24]) are listed, with "Tx" denoting a translation contact and "Ix" denoting an inversion contact. The planar1 value approximates the angle between the two aromatic rings in degrees and is defined as C8_C5_C11_C14.

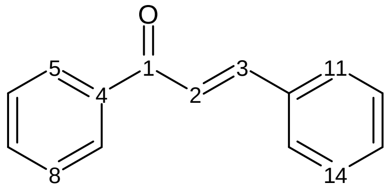

Name	Chalcone
CSD REFCODE	BZYACO01
Formula	$C_{15}H_{12}O$
ψ_{mol} published	Price et al.[24]
ψ_{crys} published	Unpublished

Key	Original ψ_{mol} label	Z′	Space group	mols / cell	Reason
CHA01	A17130 (=BZYACO03$_{maj}$, RT, form II)	1	*Pbca*	8	Experimentally observed<br>planar1=-0.48<br>Lowest energy ψ_{mol} structure
CHA02	A315 (=BZYACO, RT, form I)	1	*Pca*2$_1$	4	Experimentally observed<br>planar1=4.91
CHA03	A364	1	*P*-1	2	planar1=0.69<br>I4, I10, T3, T5
CHA04	A117	1	*P*-1	2	planar1=-15.12<br>I1, I2, I4, I6
CHA05	A235	1	*P*-1	2	planar1=0.64<br>I8, T2, T5
CHA06	A11708	1	*P*-1	2	planar1=-44.97<br>I1, T1
CHA07	A1813	1	*P*-1	2	planar1=-43.94<br>I1, T5
CHA08	A1361	1	*P*-1	2	planar1=-42.73<br>I6, I7
CHA09	A4928	1	*P*2$_1$/*a*	4	planar1=36.52<br>I3
CHA10	A1220	1	*P*-1	2	planar1=34.68<br>I4
CHA11	A113	1	*P*2$_1$2$_1$2	4	planar1=-6.58<br>T4
CHA12	B2401	2	*P*2$_1$/*c*	8	Lowest energy structure with Region B conformation
CHA13	B1	1	*Pbca*	8	Second lowest energy structure with Region B conformation
CHA14	A3384	1	*P*2$_1$	2	planar1=-0.05<br>Lowest energy ψ_{mol} structure in Sohncke space group

S2.4.1.2 Most thermodynamically stable form at low temperature

It is not clear which is the more stable low temperature structure from the available information in the literature. CHA I has a melting point of 59 °C and CHA II has a melting point of 56 °C. No DSC data is reported in any of the crystal structure determinations. We are reporting energies relative to CHA02 (ordered CHA I), since that has the higher melting point and is therefore more stable at least near the melting point.

S2.4.1.3 Previous CSP method

Flexible molecule searches were carried out in CrystalPredictor 2.2. The resulting crystal structures were optimized with CrystalOptimizer 2.4.7 using DMACRYS 2.3.0 as the lattice energy minimizing program, with intramolecular

energy being evaluated at the PBE0/6-31G(d,p) level of theory, multipoles extracted from the charge density calculated at the same level of theory, and repulsion-dispersion parameters taken from the FIT potential.

S2.4.1.4 Plane wave basis set cutoff and k-point spacing for crystal structure optimization

Convergence of the plane wave basis set cutoff and k-point spacing was tested for CHA01 and CHA02. The point at which increasing the plane wave basis set cutoff by 100 eV led to a change in energy of less than 1 kJ mol^{-1} was 900 eV for both structures, and the point at which increasing the plane wave basis set cutoff by 100 eV led to a change in relative energy of less than 0.05 kJ mol^{-1} was 700 eV. k-point spacings of 0.1, 0.08, 0.06 and 0.04 $Å^{-1}$ were tested, and the energy difference at all steps was below 1 kJ mol^{-1}.

A plane wave basis set cut off of 900 eV and a k-point spacing of 0.08 $Å^{-1}$ was used for all crystal structures of CHA.

S2.4.1.5 Isolated molecule calculations

The experimental analysis of all the crystal structures of substituted chalcones showed that there were three common conformations.[24] The planar and twisted conformations were very similar in energy, but the two were only in distinct conformational energy wells with the MP2 level of theory. The third conformation, denoted "Region B" in the previous paper, was generally much higher in energy. Therefore, the MP2/6-31G(d,p) optimized molecular conformations were used as starting points for the molecular calculations of this molecule.

For evaluation of the molecular energy within the periodic electronic structure method, the point at which increasing the space between molecules by 2 Å led to an energy difference of less than 0.1 kJ mol^{-1} was 7 Å for CHA_planar, and so boxes with this spacing between molecules were used for the molecule optimizations for the ψ_{crys} method.

S2.4.2 Ibuprofen

S2.4.2.1 Structure selection

Table S11. Structures selected for IBP. ϑ1 is defined by C1-C2···C10-C11, ϑ2 by O1-C1-C2-C3, and ϑ3 by C12-C11-C10-C7. "Opp" has ϑ_1 close to 180° and "Same" has ϑ_1 close to 0°. "Ac" has ϑ_2 close to 40° and "At" has ϑ_2 close to 150°. "1," "2," and "3" have ϑ_3 close to 60°, 180° and -60° respectively.

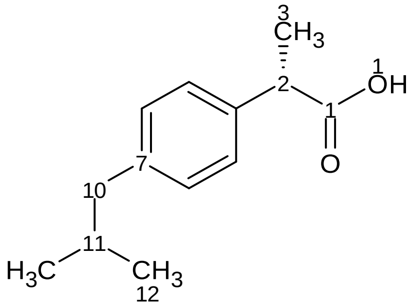

Name	Ibuprofen
CSD REFCODE	IBPRAC, JEKNOC
Formula	$C_{13}H_{18}O_2$
ψ_{mol} published	Francia et al.[25]
ψ_{crys} published	Unpublished

Key	Original ψ_{mol} label	Z'	Space group	mols / cell	Reason
IBP01	A227 (=IBPRAC01, 100 K, Racemic form I)	1	$P2_1/c$	4	Experimentally observed, lowest energy ψ_{mol} structure (OppAt2; $R_2^2(8)$)
IBP02	Ee1 (=JEKNOC12, 99 K, Enantiopure form I)	2	$P2_1$	4	Experimentally observed, lowest energy chiral ψ_{mol} structure (OppAc2; $R_2^2(8)$)
IBP03	E2 (=IBPRAC04, 258 K, Racemic form II)	2	*P*-1	4	Experimentally observed (OppAc1, OppAt2; $R_2^2(8)$)
IBP04	A6037	1	$C2/c$	4	Lowest energy Same conformation (SameAt2; $R_2^2(8)$)
IBP05	A4124	1	$P2_1/c$	4	Lowest energy different i-Pr conformation (OppAt3; $R_2^2(8)$)
IBP06	A6314	1	$P2_1$	2	Packing (SameAt3; $C_1^1(4)$)
IBP07	A2863	1	$P2_1/c$	4	Packing (OppAc1; $C_1^1(2)$)

S2.4.2.2 Most thermodynamically stable form at low temperature

For chiral molecules, the racemic form is usually more stable and will not crystallize if the enantiopure form is more stable. Then, enantiopure forms can only be crystalized when just one hand of the molecule is present. IBP racemic form II is reported as being metastable,[26] and so energies are calculated relative to IBP01 (racemic form I).

S2.4.2.3 Previous CSP method

A flexible-molecule search was carried out in CrystalPredictor 1.9. The resulting crystal structures were optimized with DFTB3-D3,[27] to relax atomic positions and remove unfeasible structures that resulted from the rigid-fragment method of the search algorithm. A single step DMACRYS calculation was run to obtain accurate energies, using distributed multipoles obtained from the PBE0/aug-cc-pVDZ charge density with GDMA2.2 and repulsion-dispersion parameters taken from the FIT potential. The lowest energy structures were then reoptimized with CrystalOptimizer 2.4.7.1, with intramolecular energy evaluated at the PBE0/6-31G(d,p) level, distributed multipoles derived from the PBE0/aug-cc-pVDZ charge density, and repulsion-dispersion parameters taken from the FIT potential.

S2.4.2.4 Plane wave basis set cutoff and k-point spacing for crystal structure optimization

Convergence of the plane wave basis set cutoff and k-point spacing was tested for IBP01 and IBP02. The point at which increasing the plane wave basis set cutoff by 100 eV led to a change in absolute energy of less than 1 kJ mol^{-1} was 900 eV for both structures, and the point at which increasing the plane wave basis set cutoff by 100 eV led to a change in relative energy of less than 0.1 kJ mol^{-1} was 700 eV. k-point spacings of 0.1, 0.06 and 0.04 Å^{-1} were tested, and the energy difference at all steps was below 1 kJ mol^{-1}.

A plane wave basis set cut off of 900 eV and a k-point spacing of 0.1 Å^{-1} was used for all crystal structures of IBP.

S2.4.2.5 Isolated molecule calculations

Extensive conformational analysis of IBP has been carried out previously.[25] The main descriptor is whether the acid and isopropyl groups are on the same or opposite sides of the aromatic ring (denoted Same and Opp, respectively). Then the OH group of the propanoic acid can be either cis or trans to the methyl group (denoted Ac and At respectively). Finally, there are three different configurations of the isopropyl group, two of which are roughly degenerate, but which have different overall shapes of the molecule (denoted 1, 2 and 3). All conformations were optimized with GAUSSIAN at the PBE0/6-31G(d,p) level of theory, and IBP_SameAt1 was the lowest in energy.

For evaluation of the molecular energy within the periodic electronic structure method, the point at which increasing the space between molecules by 2 Å led to an energy difference of less than 0.1 kJ mol^{-1} was 6 Å for IBP_SameAt1, and so boxes with this spacing between molecules were used for the molecule optimizations for the ψ_{crys} method.

S2.4.3 Naproxen

S2.4.3.1 Structure selection

Table S12. Structures selected for NAP. ϑ_1 is defined by C14-O1-C2-C1, ϑ_2 by C12-C11-C7-C8, and ϑ_3 by O2-C12-C11-C7. "A" has ϑ_1 close to 0° and "B" has ϑ_1 close to 180°. "C" and "E" have ϑ_2 close to -60 and "D" and "F" have ϑ_2 close to 120°. "C" and "D" have ϑ_3 close to 90° and "E" and "F" have ϑ_3 close to -90°.

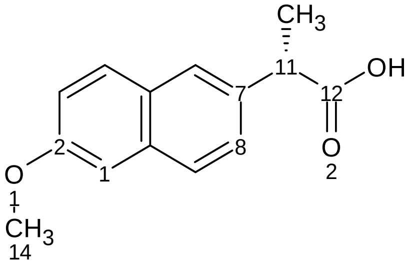

Name	Naproxen
CSD REFCODE	PAPTUX, COYRUD
Formula	$C_{14}H_{14}O_3$
ψ_{mol} published	Braun et al.[28]
ψ_{crys} published	Unpublished

Key	Original ψ_{mol} label	Z′	Space group	mols / cell	Reason
NAP01	AC4 (=PAPTUX, RT, Racemic form I)	2	*Pbc*2$_1$	8	Experimentally observed, lowest energy ψ_{mol} structure
NAP02	AC20 (=COYRUD14, 100 K, Enantiopure form I)	1	*P*2$_1$	2	Experimentally observed

NAP03	XX4 (=PAPTUX, RT, Racemic form I)	1	*Pbca*	8	Saddle point of experimentally observed structure
NAP04	AF13	1	*Pbca*	8	Alternative conformation, same packing as NAP01 (PAPTUX)
NAP05	BC11	1	$P2_1$	2	Alternative conformation, same packing as NAP02 (COYRUD14)
NAP06	AD15	1	$P2_1$	2	Lowest energy chiral ψ_{mol} structure
NAP07	AD2	1	*C2/c*	4	Dense, low energy structure

S2.4.3.2 Most thermodynamically stable form at low temperature

For chiral molecules, the racemic form is usually more stable and will not crystallize if the enantiopure form is more stable. Then, enantiopure forms can only be crystalized when just one hand of the molecule is present. It is rare for the two hands of the molecule to spontaneously resolve to a physical mixture of enantiopure crystals. Energies are calculated relative to NAP01, the racemic form.

S2.4.3.3 Previous CSP method

The crystal structures from the previous work were no longer available, and so a new search was run, following the methodology reported in the previous paper as far as possible. This will give some small differences in lattice energy from those reported in the previous paper.[28]

For the new search, flexible molecule searches were carried out in CrystalPredictor 2.4.3. The resulting crystal structures were optimized with CrystalOptimizer 2.4.7.1 using DMACRYS 2.3.1.1 as the lattice energy minimizing program, with intramolecular energy being evaluated at the PBE0/6-31G(d,p) level of theory, multipoles extracted from the charge density calculated at the same level of theory, and repulsion-dispersion parameters taken from the FIT potential.

S2.4.3.4 Plane wave basis set cutoff and k-point spacing for crystal structure optimization

Convergence of the plane wave basis set cutoff and k-point spacing was tested for NAP01 and NAP02. The point at which increasing the plane wave basis set cutoff by 100 eV led to a change in absolute energy of less than 1 kJ mol^{-1} was 900 eV for both structures, and the point at which increasing the plane wave basis set cutoff by 100 eV led to a change in relative energy of less than 0.01 kJ mol^{-1} was 700 eV. k-point spacings of 0.1, 0.06 and 0.04 Å^{-1} were tested, and the energy difference at all steps was below 1 kJ mol^{-1}.

A plane wave basis set cut off of 900 eV and a k-point spacing of 0.1 Å^{-1} was used for all crystal structures of NAP.

S2.4.3.5 Isolated molecule calculations

NAP has two configurations of the methoxy group, and these were named A and B. The propanoic acid group always had the hydrogen atom in the same plane as the aromatic rings, and the two possible configurations were denoted C/E and D/F. Conformations with the carbonyl group of the acid cis to the methyl group were denoted C and D, and conformations with the hydroxyl group of the acid cis to the methyl group were denoted E and F. All conformations were optimized in GAUSSIAN with the PBE0/6-31G(d,p) method, and NAP_AC found to be the most stable.

For evaluation of the molecular energy within the periodic electronic structure method, the point at which increasing the space between molecules by 2 Å led to an energy difference of less than 0.1 kJ mol^{-1} was 7 Å for NAP_AC, and so boxes with this spacing between molecules were used for the molecule optimizations for the ψ_{crys} method.

S2.4.3.6 Multiple structure matches

There were different computer-generated structures that were reasonable matches for both of the experimental crystal structures. These different structures came from different conformational regions.

For the racemic crystal structure, NAP01 was the closest match with the same conformation as that determined experimentally ($RMSD_{20}$=1.051 Å from the ψ_{mol} model), yet NAP04 also matched 20 molecules with a better overlap ($RMSD_{20}$=0.604 Å from the ψ_{mol} model). The difference is in the rotation of the propanoic acid group, and this is a case where the two enantiomers of the molecule take up roughly the same space (Figure S5). Optimization of the two computer-generated structures with the ψ_{crys}(PBE+TS) computational model led to structure NAP01 matching

the experimental determination with an $RMSD_{20}$ of 0.198 Å, yet the overlap of structure NAP04 with the experimental structure remained high at 0.601 Å.

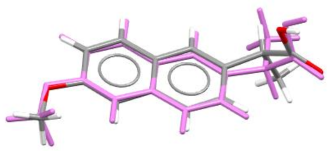

Figure S5. A single molecule of NAP from CSD structure PAPTUX (colored by element) overlaid with a single molecule of NAP from computer-generated structure NAP04 (colored lilac). $RMSD_1$=0.417 Å ignoring hydrogen atoms.

For the enantiopure crystal structure, NAP02 was the closest matching structure with the same conformation as the experimental determination ($RMSD_{20}$=0.5 Å from the ψ_{mol} model), yet NAP05 also matched 20 molecules, albeit with a slightly poorer overlap ($RMSD_{20}$=0.869 Å). In this case, the difference is not in the propanoic acid group but in the methoxy group. Optimization of the two computer-generated structures with ψ_{crys}(PBE+TS) led to NAP02 matching the experimental determination with an $RMSD_{20}$ of 0.238 Å, yet the overlap of structure NAP05 with the experimental crystal structure increased to 0.91 Å.

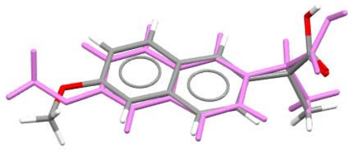

Figure S6. A single molecule of NAP from CSD structure COYRUD14 (colored by element) overlaid with a single molecule of NAP from computer-generated structure NAP05 (colored lilac). $RMSD_1$=0.528 A ignoring hydrogen atoms.

These two crystal structures have relatively high $RMSD_{20}$ structural matches (indicating poor matches) with the experimentally determined structures, even after optimization with the ψ_{crys}(PBE+TS) model. It is possible that this is an indication of disorder in the crystal structures that has not been modelled in the crystal structure determinations.

S2.4.4 Desloratadine

S2.4.4.1 Structure selection

Table S13. Structures selected for DES. ϑ_1 is defined by C5-C6-C7-C8, ϑ_2 by N2···C15=C14···C7, and ϑ_3 by H1-N2···C15=C14. The first letter is "A" for ϑ_1 close to 60° and "S" for ϑ_1 close to -60°. The second letter is "A" for ϑ_2 close to 20° and "S" for ϑ_2 close to -160°. The third letter is "A" for ϑ_3 close to 180° and "E" for ϑ_3 close to 0°.

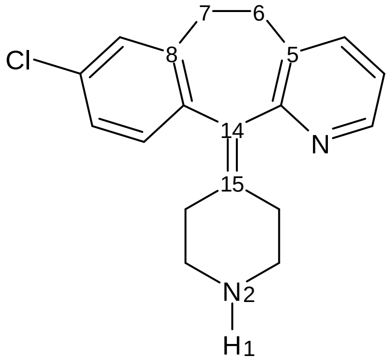

Name	Desloratadine
CSD REFCODE	GEHXEX
Formula	$C_{19}H_{19}ClN_2$
ψ_{mol} published	Srirambhatla et al.[29]
ψ_{crys} published	Srirambhatla et al.[29]

Key	Original ψ_{mol} label	Z′	Space group	mols / cell	Reason
DES01	AAA1 (=GEHXEX01, 80 K, form I)	1	$P2_1$	2	Experimentally observed, chiral structure
DES02	EO2 (=GEHXEX04, 335 K, form II)	2	$P2_1$	4	Experimentally observed, chiral structure
DES03	ASE1 (=GEHXEX02, 360 K, form III)	1	$P2_1$	2	Experimentally observed, chiral structure

DES04	SSACS1	1	$P2_1/n$	4	Lowest energy ψ_{mol} structure $C_1^1(2)$ hydrogen bonding
DES05	SSA1	1	$P2_12_12_1$	4	Lowest energy chiral ψ_{mol} structure $C_1^1(2)$ hydrogen bonding
DES06	SAACS7	1	$Pbca$	8	Lowest energy $C_1^1(8)$ hydrogen bonding
DES07	SAECS8	1	$P2_1/c$	4	Lowest energy $R_2^2(4)$ hydrogen bonding
DES08	AAECS1	1	$P2_1/c$	4	Lowest energy $R_2^2(16)$ hydrogen bonding
DES09	ASACS1	1	$Pbcn$	8	Lowest energy ASA conformation
DES10	SAE1	1	$P2_12_12_1$	4	Lowest energy SAE conformation
DES11	SSECS1	1	$P2_1/n$	4	Lowest energy SSE conformation

S2.4.4.2 Most thermodynamically stable form at low temperature

The DSC data suggest that DES I is the thermodynamically stable form at room temperature (RT) with DES II and DES III being enantiotropically related.[29] The powder diffraction data were recorded at 300 K (DES I), 320 K (DES II) and 340 K (DES III). Energies are reported relative to DES01 (DES I).

S2.4.4.3 Previous CSP method

Rigid molecule Z searches were carried out in CrystalPredictor 2.1.01 for each of the 8 conformations described in Section S2.4.4.5. The resulting crystal structures were optimized with CrystalOptimizer 2.4 using DMACRYS 2.2.0.1 as the lattice energy minimizing program, with intramolecular energy being evaluated at the PBE0/6-31G(d,p) level of theory, multipoles extracted from the charge density calculated at the same level of theory, and repulsion-dispersion parameters taken from the FIT potential.

S2.4.4.4 Plane wave basis set cutoff and k-point spacing for crystal structure optimization

Convergence of the plane wave basis set cutoff and k-point spacing was tested for DES01 and DES04. The point at which increasing the plane wave basis set cutoff by 100 eV led to a change in energy of less than 1 kJ mol^{-1} was 900 eV for both structures, and the point at which increasing the plane wave basis set cutoff by 100 eV led to a change in relative energy of less than 0.05 kJ mol^{-1} was 700 eV. k-point spacings of 0.1, 0.06 and 0.04 Å^{-1} were tested, and the energy difference for 0.06 and 0.04 was below 1 kJ mol^{-1}. A plane wave basis set cut off of 900 eV and a k-point spacing of 0.06 Å^{-1} was used for all crystal structures of DES.

S2.4.4.5 Isolated molecule calculations

The previous work identified three parts of the molecule which could each take two states. This generated eight distinct conformations. The first letter, A or S, denoted whether the bend in the 7-membered ring was next to the ring with the nitrogen or next to the ring with the chlorine. The second letter, A or S, denoted whether the piperidine ring sticks out or sits under the 7-membered ring. The third letter, A or E, denoted whether the hydrogen on the piperidine ring was axial or equatorial. All conformations were optimized in GAUSSIAN with the PBE0/6-31G(d,p) method, and DES_SAA found to be the most stable.

For evaluation of the molecular energy within the periodic electronic structure method, the point at which increasing the space between molecules by 2 Å led to an energy difference of less than 0.1 kJ mol^{-1} was 8 Å for DES_SAA, and so boxes with this spacing between molecules were used for the molecule optimizations for the ψ_{crys} method.

S2.4.5 Sulfamerazine

S2.4.5.1 Structure selection

Table S14. Structures selected for SMZ. ϑ_1 is defined by C11-C4···N3-H10 and ϑ_2 by C1-N3-S1-C5. The first letter is "O" for ϑ_1 close to 180° and "S" for ϑ_1 close to 0°. The second letter corresponds to the pyramidality of the NH_2 group prior to the search, but this was found not to be retained on optimization.

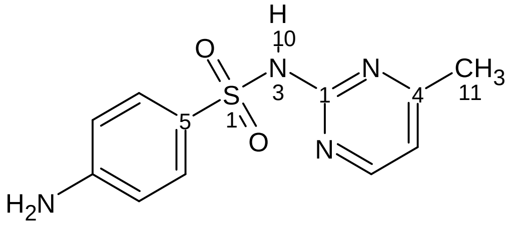

Name	Sulfamerazine
CSD REFCODE	SLFNMA
Formula	$C_{11}H_{12}N_4O_2S$
ψ_{mol} published	in preparation
ψ_{crys} published	in preparation

Key	Original ψ_{mol} label	Z′	Space group	mols / cell	Reason
SMZ01	OB1856 (=SLFNMA02, RT, form I)	2	$Pn2_1a$	8	Experimentally observed
SMZ02	OA25 (=SLFNMA01, RT, form II)	1	*Pbca*	8	Experimentally observed
SMZ03	OA17 (=SLFNMA03, 150 K, form III)	1	$P2_1/c$	4	Experimentally observed
SMZ04	OA2 (=SLFNMA06, 150 K, form IV)	1	$P2_1/c$	4	Experimentally observed. Lowest energy ψ_{mol} structure
SMZ05	SC45	1	$P2_12_12_1$	4	Lowest energy chiral ψ_{mol} structure
SMZ06	OA1153	1	$P2_1/a$	4	Distinct packing (different layer)
SMZ07	OC1250	1	$P2_1/a$	4	Distinct packing (different layer)
SMZ08	SC583	1	*P*-1	2	Lowest energy structure with alternative conformation

S2.4.5.2 Most thermodynamically stable form at low temperature

"Examination of the sulfamerazine polymorphs shows that polymorphs I and II are enantiotropically related, with II stable at lower temperatures and I stable at higher."[30] Energies are reported relative to SMZ02 (SMZ II).

S2.4.5.3 Previous CSP method

Flexible molecule searches were carried out in CrystalPredictor 2.4.3. The resulting crystal structures were optimized with CrystalOptimizer 2.4.7.1 using DMACRYS 2.3.1.1 as the lattice energy minimizing program, with intramolecular energy being evaluated at the PBE0/6-31G(d,p) level of theory, multipoles extracted from the charge density calculated at the same level of theory, and repulsion-dispersion parameters taken from the FIT potential with Scheraga's sulfoxide parameters.[7]

S2.4.5.4 Plane wave basis set cutoff and k-point spacing for crystal structure optimization

Convergence of the plane wave basis set cutoff and k-point spacing was tested for SMZ01 and SMZ02. The point at which increasing the plane wave basis set cutoff by 100 eV led to a change in energy of less than 1 kJ mol^{-1} was 900 eV for both structures, and the point at which increasing the plane wave basis set cutoff by 100 eV led to a change in relative energy of less than 0.05 kJ mol^{-1} was 800 eV. k-point spacings of 0.1 and 0.06 $Å^{-1}$ were tested, and the energy difference at all steps was below 1 kJ mol^{-1}. A plane wave basis set cut off of 900 eV and a k-point spacing of 0.1 $Å^{-1}$was used for all crystal structures of SMZ.

S2.4.5.5 Isolated molecule calculations

For evaluation of the molecular energy within the periodic electronic structure method, the point at which increasing the space between molecules by 2 Å led to an energy difference of less than 0.1 kJ mol^{-1} was 17 Å for SMZ_opp, and so boxes with this spacing between molecules were used for the molecule optimizations for the ψ_{crys} method.

S3 Experimental Information

S3.1 Crystallization conditions

The crystallization methods reported for the CSD determinations of each of the polymorphs investigated in this work are given here. In some cases, the best reported crystal structure was from an elaborate crystallization method, where a different determination had a more routine crystallization method. These are noted as appropriate. The information in Table S15-Table S18 is all taken from the papers linked to the CSD depositions, i.e. the method used for growing a sample suitable for structure determination. No other literature has been used in defining the crystallization methods, which are categorized as:

1. Routine crystallization from solvent.
2. Crystallization from solvent, but with additive or external modification.
3. Solvent free crystallization (e.g. heating or sublimation).

S3.1.1 Rigid molecules

Table S15. Crystallization conditions reported for the experimentally observed polymorphs of small rigid molecules investigated in this study. Where two conditions are reported, the first is from the CSD structure determination used as the reference in this work and the second is a less elaborate experiment which also led to a structure determination on the CSD.

Polymorph	Crystallization conditions	Category
CRN		
γ form	Obtained during cocrystallization of coronene with F_4-TCNQ.[31]<br>Other reports include slow crystallization from xylene or benzene.[32, 33]	1
β form	Slow cooling of toluene solution in 1 Tesla magnetic field.[3]	2
ACR		
Form II	Crystallization from DMF or 1:1 EtOH:MeOH mixture.[34]	1
Form III	Recrystallization from ethanol (maybe concomitantly with II or IV).[35]	1
Form IV	Recrystallization from DMSO.[5]	1
Form VI	Template-assisted crystallization.[34]	3
Form VII	Template-assisted crystallization.[34]	3
Form IX	Slow evaporation from toluene.[36]	1
PTH		
Form I	Attempted crystallization of N,N'-dithiodiphthalimide from hot pyridine.[37]	1
SAC		
Form I	Slow evaporation from acetone.[38]	1
FLU		
Form I	The vast majority of crystallizations from the polymorph screen. [8]	1
Form II	Solvent evaporation of a saturated solution of the compound in nitromethane at room temperature over a period of three months.[8]	1

S3.1.2 Carbamazepine family

Table S16. Crystallization conditions reported for the experimentally observed polymorphs of carbamazepine family molecules investigated in this study. Where two conditions are reported, the first is from the CSD structure determination used as the reference in this work and the second is a less elaborate experiment which also led to a structure determination on the CSD.

Polymorph	Crystallization conditions	Category
CBZ		
Form I	Crystallized from the melt in a sealed capillary.[13]<br>Heating form III to above 165 °C.[39]	3
Form II	Slow evaporation from THF, CHF_3 and CCl_4.[40]<br>This structure may contain residual solvent (Section S2.2.3).	1
Form III	Recrystallization from ethanol.[41]	1
Form IV	Evaporation of a methanol solution in the presence of hydroxypropylcellulose.[42]	2
Form V	Sublimation onto template.[9]	3
DHC		

Form I	Recrystallized from ethanol.[43]	1
Form II	Recrystallized from acetonitrile.[44]	1
Form III	Recrystallized from methanol.[45]	1
Form IV	Grown from the vapour by sublimation.[14]	3
CYH		
Form I	As supplied by Sigma-Aldrich.[46]	1
Form II	Heating Form I to 458 K and then cooling to 100 K.[15]	3
Form III	Sublimation onto dihydrocarbmazepine form II.[10]	3
CYT		
Form I	Crystallized from industrial methylated spirits.[16]<br>Reported to contain residual solvent.	1
Form II	Heating form I to 498 K and cooling to RT.[16]	3
OXC		
Form I	Directly from the bottle, or evaporation of methanol solution.[17]	1
Form II	Dissolved in toluene, heated to 115°C, cooled to RT or placed in an ice bath.[17]	1
Form III	From the vapour phase (all substrates – Au, Ag and Cu foils; Ag-coated glass) or solution (binary solvent mixtures of ethanol and toluene).[47]	3

S3.1.3 Fenamate family

Table S17. Crystallization conditions reported for the experimentally observed polymorphs of fenamate family molecules investigated in this study. Where two conditions are reported, the first is from the CSD structure determination used as the reference in this work and the second is a less elaborate experiment which also led to a structure determination on the CSD.

Polymorph	Crystallization conditions	Category
FEA		
Form I	Sublimation.[48]<br>From 50:50 ethanol acetone solution.[49]	1
MFA		
Form I	Not reported (2 private communications, and one very old article which is not available online). CSD has “from N,N-dimethylformamide / acetone” for one private communication.[50]<br>A determination with no coordinates has this prepared by slow evaporation from ethanol.[51]	1
Form II	Slow evaporation from methanol in the presence of bifunctional SAM substrates.[52]<br>Other determinations have slow evaporation from chloroform,[53] or slow evaporation from ethanol with FFA as an additive.[54]	1
Form III	Cocrystallization attempts with adenine in a 1:1 DMF/methanol mixture, followed by slow evaporation at room temperature. [53]	2
TFA		
Form I	Recrystallization from absolute ethanol.[55]	1
Form II	Rapid cooling of a boiling 96% ethanol solution using an ice bath.[55]	1
Form III	Grown from an ethanol solution using nonpolar aromatic polymers as heteronuclei.[56]	2
Form IV	Grown from an ethanol solution using nonpolar aromatic polymers as heteronuclei.[56]	2
Form V	Grown from an ethanol solution using nonpolar aromatic polymers as heteronuclei.[56]	2
Form VI	Sublimation onto isomorphous mefenamic acid form I.[19]	3
Form VII	Sublimation onto tolfenamic/flufenamic acid solid solution.[19]	3
Form VIII	Sublimation onto a metal surface.[19]	3
Form IX	A suspension of tolfenamic acid form I in 2-propanol was heated to 50°C. After dissolution and filtering into a crystallizing dish, the dish was covered with parafilm and put in a fridge at 5°C. After two hours a few blocky crystals of form IX were discovered concomitantly with forms I and II.[22]	1
FFA		
Form I	Recrystallization from mixed xylenes above 130 °C.[57]	1

Form II	Grown from ethanol solution using polymers as heteronuclei.[58]	2
Form III	Recrystallization from methanol at room temperature.[57]	1
Form IV	Grown from ethanol solution using polymers as heteronuclei.[58]	2
Form V	Grown from ethanol solution using polymers as heteronuclei.[58]	2
Form VI	Low temperature (-130 °C) transformation from form IV, by submerging a vial in liquid nitrogen for 10-15 minutes.[58]	3
Form VII	Encapsulated nanodroplet crystallization. 200 nL PDMSO, 50 nL of 50 mg/mL flufenamic acid in DMF, 50 nL H_2O.[59]	2
Form VIII	Grown from ethanol solution using polymers as heteronuclei.[58]	2
NFA		
Form I	Private communication – from methanol.<br>Grown from a THF solution.[60]	1

S3.1.4 Small drug molecules

Table S18. Crystallization conditions reported for the experimentally observed polymorphs of small drug molecules investigated in this study. Where two conditions are reported, the first is from the CSD structure determination used as the reference in this work and the second is a less elaborate experiment which also led to a structure determination on the CSD.

Polymorph	Crystallization conditions	Category
CHA		
Form I	Grown from slowly cooled ethanol solutions.[61]	1
Form II	Recrystallized from 2-propanol.[62]	1
IBP		
Racemic Form I	Recrystallized from acetonitrile.[63]	1
Racemic Form II	High temperature annealing.[64]	3
Enantiopure form	Slow evaporation from methanol solution.[65]	1
NAP		
Racemic form	Recrystallized from ethanol.[28]	1
Enantiopure form	Melt crystallization between two squeezed CaF_2 plates.[66]<br>From benzene or ethanol/water solutions.[67-69]	1
DES		
Form I	Slow evaporation from ethyl acetate.[29]	1
Form II	Heating in DSC or VT-PXRD.[29]	3
Form III	Heating in DSC or VT-PXRD.[29]	3
SMZ		
Form I	Crystallization from dry methanol.[70]	1
Form II	Slow evaporation from acetone.[71]	1
Form III	Slow evaporation from DMF at room temperature.[72]	1
Form IV	Cooling crystallization from ethanol with added ammonia.[73]	2

S3.2 Experimental and estimated heats of sublimation

S3.2.1 Experimental heats of sublimation

Two recent review articles have reported experimental heats of sublimation for many molecules.[74, 75] Chickos and Gavezzotti have used heats of sublimation recorded at a variety of temperatures, although many have been corrected to 298 K (all the molecules in our paper that are included in their work were reported at 298 K).[74] Their work has the advantage that the polymorph is sometimes specified by the CSD REFCODE. Perlovich and Raevsky have not specified which polymorph was measured, but they have corrected all measurements to 298 K.[75] Some molecules in our selection do not have data in either work, so we have interrogated the NIST Chemistry Webbook. Where we have done so, the underlying reference is given in Table S20.

We have ordered the literature experimental heats of sublimation in Table S20 according to the following hierarchy:

1. From Chickos and Gavezzotti,[74] (REFCODE given if specified)

2. From Perlovich and Raevsky[75]
3. Other data from NIST Chemistry Webbook since 2000 (or single most recent point if all pre 2000), if at 298 K
4. Other data from NIST Chemistry Webbook since 2000 (or single most recent point if all pre 2000)

Error estimates are reported if available. The first value reported in Table S20 is used for other Figures and Tables in this work.

S3.2.2 Estimated heats of sublimation

A 2003 paper gave a method of estimating the experimental heat of sublimation from the functional groups within a molecule.[76] An 18-parameter equation was fitted to experimental heats of sublimation of 226 organic compounds, including 70 which contain hydrogen bonds and acceptors.[76] The full list of molecules was not included in the paper, although the validation set of 35 molecules was.

The equation that was fitted to the heats of sublimation is:

ΔH_{sub}/kJ mol^{-1} = 6.942 + 3.127 F + 10.456 Cl + 12.926 Br + 19.763 I + 3.297 C_3 – 3.305 C_4 + 5.970 C_{arom} + 7.631 $C_{noC3C4,noarom}$ + 20.141 NH + 30.172 OH + 7.341 CO + 18.249 O_{ether} + 8.466 NO + 20.585 SO + 19.676 CS + 12.840 S_{ether} + 11.415 $N_{nitrile}$ + 8.953 $N_{nonitrile}$

The definitions of the terms in the equation are given in Table S19. Recalculating the estimated heats of sublimation of the molecules in the reported validation set has given further insight into how the equation should be applied. For example, for NO_2 and SO_2 the number of oxygen atoms should be counted (i.e. NO=2 and SO=2 for these groups), rather than the number of nitrogen or sulfur atoms as the definition says. The set of molecules in the validation set does not fully clarify how carbons with non-hydrogen terminal atoms (e.g. F or Cl) should be treated, as no such molecules were included. Applying the definition strictly to a CF_3 group gives this as a quaternary C atom (i.e. C_4), but in the extreme example of ZUKLEW (a perfluorocarbon molecule with molecular formula $C_{14}F_{20}$), the estimated heat of sublimation would be given as 23.212 kJ mol^{-1} by this definition. If terminal single atoms are treated in the same manner as hydrogen when assigning types to carbon atoms (i.e. as $C_{noC3C4,noarom}$) gives a more reasonable ΔH_{sub} of 141.644 kJ mol^{-1} for this molecule. Hence we have chosen to use this second treatment of similar atoms.

The authors mentioned the variety of different temperatures in the data available at the time.[76] They adjusted some heats of sublimation to a standard temperature of 298.15 K according to the method of Chickos for estimating unknown heat capacities,[77] and refitted the equations for aliphatic and aromatic hydrocarbons. The conclusion was that, while this was a potential source of error, the quality of the model is hardly affected at all.[76] As can be seen in Table S20, this estimate has some unacceptably large errors compared with experimental measurements for many of the flexible molecules.

Table S19. Definitions of the terms in the equation above for estimating the heats of sublimation from the molecular diagram. Insights we have gained into how these definitions should be interpreted are given in italics.

**Notation**	**Definition**	**Coefficient**	**Times used in our work**
C_3	Number of tertiary C atoms (C atom covalently bonded to four atoms, one of which is an H atom)	3.297	4
C_4	Number of quaternary C atoms (C atom covalently bonded to four non-H atoms)	-3.305	2
C_{arom}	Number of C atoms involved in an aromatic system, bonded to three atoms of which at least two are C atoms *(This includes carbons in pyridine and pyrazine)*	5.970	19
$C_{noC3C4,noarom}$	Number of C atoms that are neither branched nor aromatic *(Any C atom with only one or two carbons attached, that is not aromatic)*	7.631	18
CO	Number of carbonyl groups	7.341	16
CS	Number of thiocarbonyl groups	19.676	0
NO	Number of N atoms in NO_2 groups	8.466	0

	(Strictly, this is the number of O atoms in NO_2 groups. The N is not counted elsewhere)		
$N_{nitrile}$	Number of N atoms in nitrile groups	11.415	0
$N_{nonitrile}$	Number of N atoms not in nitrile groups	8.953	8
NH	Number of NH donor atoms	20.141	15
O_{ether}	Number of ether O atoms	18.249	1
OH	Number of OH donor atoms	30.172	9
SO	Number of S atoms bonded to O atoms<br>*(Similar to NO, this is the number of O atoms in SO_2 groups)*	20.585	2
S_{ether}	Number of thioether S atoms	12.840	0
F	Number of F atoms	3.127	5
Cl	Number of Cl atoms	10.456	2
Br	Number of Br atoms	12.926	0
I	Number of I atoms	19.763	0
	Constant	6.942	

Table S20. Heats of sublimation of the 20 molecules considered in this work. The estimated heat of sublimation comes from the equation reproduced in section S3.2.2 evaluated according to the definitions reproduced in Table S19.[76] Experimental heats of sublimation are ordered according to the hierarchy in section S3.2.1, with the first value reported being used in the preparation of other Figures and Tables.

Name	Estimated ΔH_{sub} /kJ mol^{-1}	Experimental ΔH_{sub} /kJ mol^{-1}
**RIGID**		
CRN	150.2	152.0 @ 298 K (CORONE)[74]<br>131.0 ± 1.7 @ 298 K[78]<br>142.6 ± 8.7 @ 298 K[79]
ACR	93.5	93.0 @ 298 K[74]<br>92.60 @ 298 K[75]<br>91.7 ± 0.4 @ 298 K[80]
PTH	92.8	106.0 @ 298 K (PHALIM04)[74]<br>106.90 @298 K[75]<br>106.3 ± 0.3 @ 298 K[81]<br>104.0 ± 0.4 @ 356 K[81]
SAC	119.0	112.60 @ 298 K[75]<br>112.6 ± 4.2 @ 298 K[82]
FLU	95.6	133.0 @ 298 K (FURACL03)[74]<br>133.20 @ 298 K[75]<br>133.2 ± 2.1 @ 298 K[83]<br>129.9 @ 394-401 K[83]<br>150 ± 2 @ 421-483 K[84]
**Carbamazepine family**		
CBZ	158.1	
DHC	158.1	
CYH	152.4	
CYT	152.4	
OXC	165.4	
**Fenamate family**		
FEA	143.9	126.0 ± 1.3 @ 298 K[85]<br>123.0 ± 1.3 @382 K[85]
MFA	159.1	136.3 ± 0.8 @ 298 K[85]***<br>132.7 ± 0.8 @ 377 K[85]
TFA	162.0	129.10 @ 298 K[75]<br>128.4 ± 0.8 @ 298 K[85]

		125.7 ± 0.8 @ 360 K[85]
FFA	160.9	121.20 @ 298 K[75]<br>121.20 ± 0.7 @ 298 K[86]<br>119.4 ± 0.7 @ 358 K[86]
NFA	163.9	130.2 ± 0.8 @ 298 K[86]<br>127.8 ± 0.8 @ 376 K[86]
**Small drugs**		
CHA	108.8	
IBP	128.3	115.8 ± 0.6 @ 298 K for racemic[87]<br>107.4 ± 0.5 @ 298 K for enantiopure[87]
NAP	148.6	130.10 @ 298 K for enantiopure[75]<br>128.3 ± 0.5 @ 341-397 K for enantiopure[88]
DES	173.2	
SMZ	193.8	

*** Private communication from Prof JBO Mitchell that this was not included in his 2010 compilation of heats of sublimation as the different thermodynamic terms reported were not consistent.

S4 Results by molecular family

S4.1 Small rigid molecules

S4.1.1 Structure optimizations

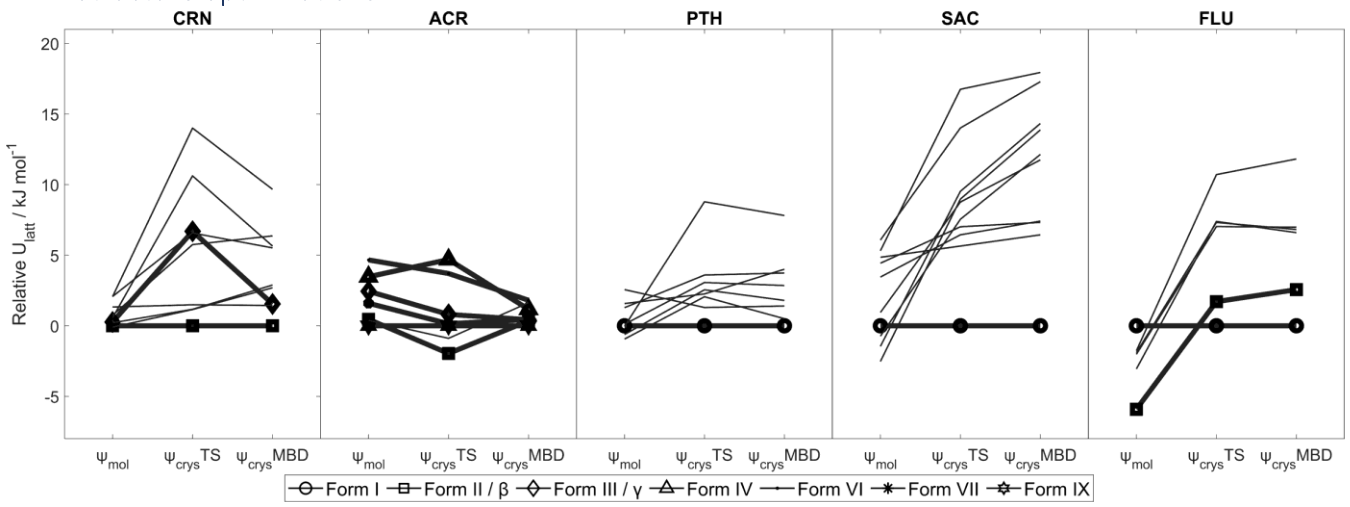

Figure S7. Relative energies of various crystal structures of CRN, ACR, PTH, SAC and FLU, with different computational models. Energies are calculated relative to that of the form believed to be most stable experimentally at low temperature (see Section S2.1 for justification). Structures with symbols are experimentally observed forms, with the form names in the legend. ψ_{mol} *denotes lattice energy minimization corresponding to the original CSP;* ψ_{crys}*TS denotes lattice energy minimization with* ψ_{crys}*(PBE+TS);* ψ_{crys}*MBD denotes single point energy calculation of the* ψ_{crys}*(PBE+TS) structure with* ψ_{crys}*(PBE+MBD).*

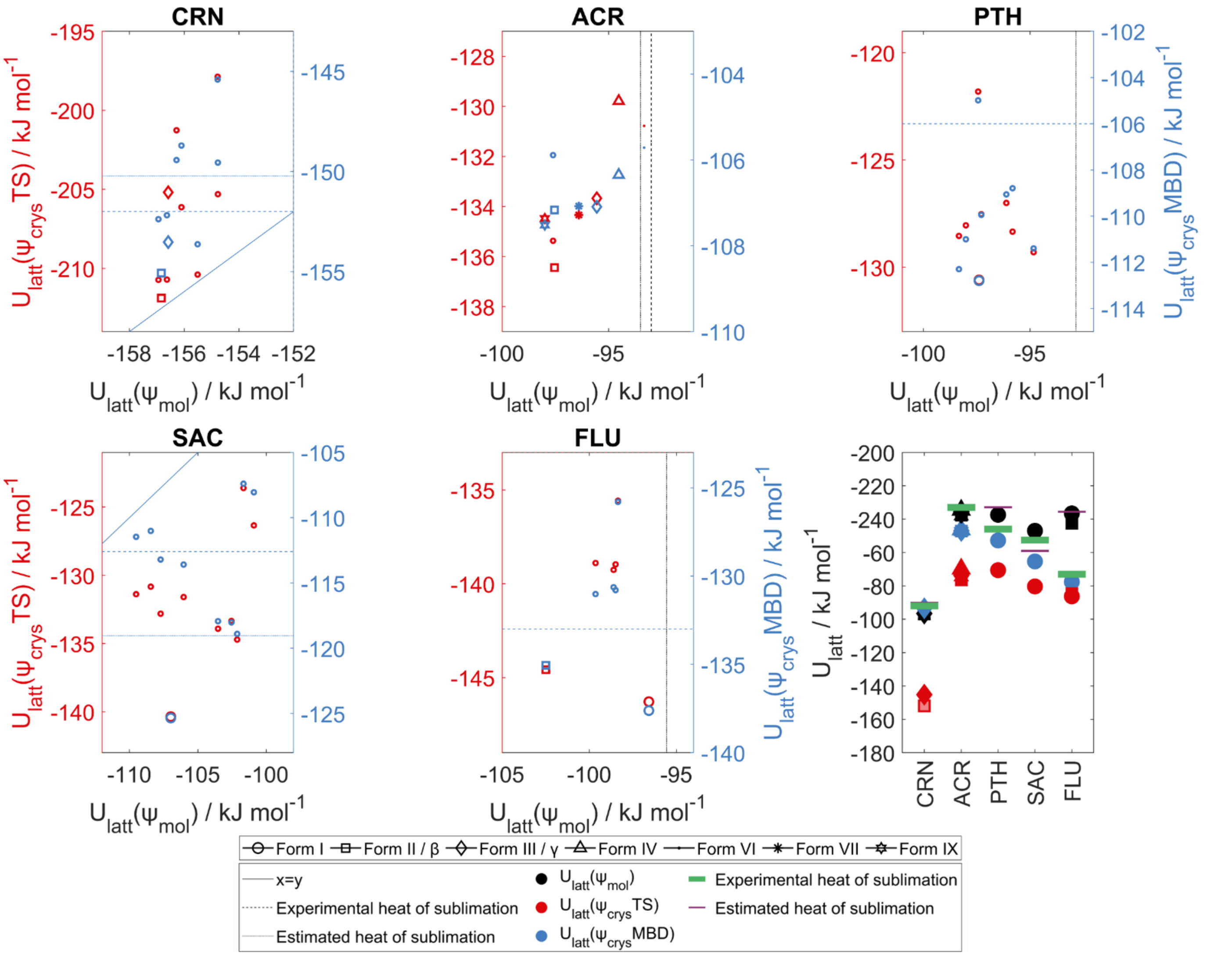

Figure S8. Comparison of lattice energies of CRN, ACR, PTH, SAC and FLU, with different computational models. Different energy scales are used for the different computational methods, colored black for ψ_{mol}, red for ψ_{crys}(PBE+TS) and blue for ψ_{crys}(PBE+MBD). The diagonal lines in the first five tiles are the x=y relationships, colored red for ψ_{mol}=ψ_{crys}(PBE+TS) and blue for ψ_{mol}=ψ_{crys}(PBE+MBD). The dashed lines are experimental heats of sublimation (where available) and the dotted lines are estimated heats of sublimation for the atomic types, placed when the scale allows, with the relevant axis colored black (vertical lines) for ψ_{mol}, red (horizontal lines) for ψ_{crys}(PBE+TS) and blue (horizontal lines) for ψ_{crys}(PBE+MBD). Similarly, when the scales allow the x=y lines to be drawn, these are included. In the bottom right corner, points are colored black for ψ_{mol}, red for ψ_{crys}(PBE+TS) and blue for ψ_{crys}(PBE+MBD), with filled symbols denoting simple solution crystallizations, shaded symbols denoting solution crystallizations with modified conditions or additives, and open symbols denoting crystallization not from solution. The green lines mark the experimental heats of sublimation (where available) and the purple lines mark the estimated heats of sublimation.

Table S21. Absolute and relative energies of various crystal structures of CRN, ACR, PTH, SAC and FLU, with different computational models.

CRN													
Label	Reason	ψ_{mol}			ψ_{crys}(PBE+TS)				ψ_{crys}(PBE+MBD) single point energy			ψ_{mol} vs. ψ_{crys}
		U_{latt} / kJ mol^{-1}	Relative U_{latt} / kJ mol^{-1}	$RMSD_{20}$ / Å	E / eV	U_{latt} / kJ mol^{-1}	Relative U_{latt} / kJ mol^{-1}	$RMSD_{20}$ / Å	E / eV	U_{latt} / kJ mol^{-1}	Relative U_{latt} / kJ mol^{-1}	$RMSD_{20}$ / Å
CRN01	Experimental (CORONE04, β form)	-156.84	0	0.199	-3980.019542	-211.875	0	0.142	-3980.156158	-155.072	0	0.235
CRN02	Experimental (CORONE03, γ form)	-156.58	0.26	0.449	-3979.950196	-205.184	6.691012	0.091	-3980.140183	-153.531	1.54138	0.471
CRN03	Lowest energy ψ_{mol} structure (Sohncke space group) (parallel stacks)	-156.94	-0.1		-3980.007619	-210.724	1.150443		-3980.128269	-152.382	2.690878	0.222
CRN04	Packing (parallel stacks)	-154.78	2.06		-3979.874415	-197.872	14.00293		-3980.055991	-145.408	9.664813	0.201
CRN05	Packing (beta stacks)	-155.51	1.33		-3980.004143	-210.389	1.485856		-3980.141254	-153.634	1.438018	0.279
CRN06	Packing (offset stacks in both directions)	-154.77	2.07		-3979.951436	-205.303	6.571384		-3980.098988	-149.556	5.516114	0.475
CRN07	Packing (double gamma stacks)	-156.28	0.56		-3979.909509	-201.258	10.61678		-3980.097665	-149.429	5.643766	0.295
CRN08	Packing (double gamma stacks)	-156.1	0.74		-3979.959969	-206.127	5.74802		-3980.090162	-148.705	6.367732	0.94
CRN09	Packing (double beta stacks)	-156.63	0.21		-3980.007442	-210.707	1.167537		-3980.126233	-152.185	2.887373	0.231
**ACR**												
Label	Reason	ψ_{mol}			ψ_{crys}(PBE+TS)				ψ_{crys}(PBE+MBD) single point energy			ψ_{mol} vs. ψ_{crys}
		U_{latt} / kJ mol^{-1}	Relative U_{latt} / kJ mol^{-1}	$RMSD_{20}$ / Å	E / eV	U_{latt} / kJ mol^{-1}	Relative U_{latt} / kJ mol^{-1}	$RMSD_{20}$ / Å	E / eV	U_{latt} / kJ mol^{-1}	Relative U_{latt} / kJ mol^{-1}	$RMSD_{20}$ / Å
ACR01	Experimental (ACRDIN04, form II)	-97.5402	0.4558	0.512	-2471.685994	-136.442	-1.96404	0.085	-2471.741426	-107.165	0.351985	0.508
ACR02	Experimental (ACRDIN09, form III)	-95.5547	2.4413	0.139	-2471.657311	-133.675	0.803494	0.178	-2471.740636	-107.089	0.428245	0.281
ACR03	Experimental (ACRDIN08, form IV, Sohncke space group)	-94.5255	3.4705	0.29	-2471.617025	-129.787	4.690573	0.407	-2471.732943	-106.347	1.170485	0.239
ACR04	Experimental (ACRDIN05, form VI)	-93.3334	4.6626	0.251	-2471.62728	-130.777	3.701098	0.225	-2471.726361	-105.712	1.805561	0.248
ACR05	Experimental (ACRDIN06, form VII)	-96.4019	1.5941	0.178	-2471.664158	-134.335	0.142887	0.107	-2471.740471	-107.073	0.444142	0.251
ACR06	Experimental (ACRDIN12, form IX)	-97.996	0	0.146	-2471.665639	-134.478	0	0.179	-2471.745074	-107.517	0	0.202
ACR07	Lowest energy ψ_{mol} structure. NB This is not the lowest energy structure following reoptimization with the updated ψ_{mol} program with improved lattice summation. See Section S2.1.2.4.	-97.6092	0.3868		-2471.674871	-135.369	-0.89078		-2471.728161	-105.885	1.631933	0.317

PTH												
Label	Reason	ψ_{mol}			ψ_{crys}(PBE+TS)				ψ_{crys}(PBE+MBD) single point energy			ψ_{mol} vs. ψ_{crys}
		U_{latt} / kJ mol^{-1}	Relative U_{latt} / kJ mol^{-1}	$RMSD_{20}$ / Å	E / eV	U_{latt} / kJ mol^{-1}	Relative U_{latt} / kJ mol^{-1}	$RMSD_{20}$ / Å	E / eV	U_{latt} / kJ mol^{-1}	Relative U_{latt} / kJ mol^{-1}	$RMSD_{20}$ / Å
PTH01	Experimental (PHALIM02, form I, $R_2^2(8)$ and both $R_2^2(10)$ and $C_1^1(5)$ C-H···O graph sets)	-97.3934	0	0.182	-2498.00999	-130.592	0	0.047	-2498.043238	-112.795	0	0.188
PTH02	Lowest energy ψ_{mol} structure ($C_1^1(4)$ graph set)	-98.3367	-0.94336		-2497.988697	-128.537	2.054514		-2498.03807	-112.296	0.498669	0.267
PTH03	Lowest energy ψ_{mol} structure in Sohncke space group ($C_1^1(4)$ with different twist from ai24 and $C_1^1(5)$ C-H···O graph sets)	-94.8323	2.561051		-2497.996576	-129.298	1.294283		-2498.028741	-111.396	1.398796	0.298
PTH04	Packing (different packing of same chain as experimental structure)	-98.0127	-0.61931		-2497.98358	-128.044	2.548272		-2498.024648	-111.001	1.793766	0.267
PTH05	Packing (different packing of same chain as experimental structure)	-97.4359	-0.04252		-2497.918955	-121.808	8.783701		-2497.962305	-104.986	7.809055	0.29
PTH06	Packing (sheet of $R_2^2(8)$ and $R_2^2(10)$ C-H···O graph sets)	-97.2874	0.105937		-2497.978217	-127.526	3.065715		-2498.013703	-109.945	2.849768	0.218
PTH07	Packing (different chain with $R_2^2(8)$ and $R_2^2(10)$ C-H···O graph sets)	-96.1118	1.281575		-2497.972764	-127	3.591845		-2498.004514	-109.059	3.736363	0.157
PTH08	Packing ($C_1^1(4)$ with same chain as bb27, and $R_2^2(10)$ C-H···O graph sets)	-95.8207	1.572682		-2497.986628	-128.338	2.254097		-2498.001747	-108.792	4.003342	0.39

SAC												
Label	Reason	ψ_{mol}			ψ_{crys}(PBE+TS)				ψ_{crys}(PBE+MBD) single point energy			ψ_{mol} vs. ψ_{crys}
		U_{latt} / kJ mol^{-1}	Relative U_{latt} / kJ mol^{-1}	$RMSD_{20}$ / Å	E / eV	U_{latt} / kJ mol^{-1}	Relative U_{latt} / kJ mol^{-1}	$RMSD_{20}$ / Å	E / eV	U_{latt} / kJ mol^{-1}	Relative U_{latt} / kJ mol^{-1}	$RMSD_{20}$ / Å
SAC01	Experimental (SCCHRN07, form I, amide dimer)	-106.98	0	0.164	-3081.942074	-140.361	0	0.178	-3082.031555	-125.343	0	0.199
SAC02	Lowest energy ψ_{mol} structure (antiparallel polar ribbon)	-109.51	-2.53		-3081.849089	-131.389	8.971793		-3081.887733	-111.466	13.87693	0.483
SAC03	Lowest energy ψ_{mol} structure in Sohncke space group (antiparallel polar ribbon)	-108.44	-1.46		-3081.843377	-130.838	9.523005		-3081.883059	-111.015	14.32789	0.534
SAC04	Packing (non-polar ribbon)	-107.72	-0.74		-3081.863894	-132.817	7.543311		-3081.905767	-113.206	12.13693	0.233
SAC05	Packing (antiparallel twisted molecule polar ribbon)	-106.04	0.94		-3081.851266	-131.599	8.761819		-3081.909771	-113.592	11.75052	0.321
SAC06	Packing (parallel twisted molecule polar ribbon)	-103.53	3.45		-3081.875295	-133.917	6.443287		-3081.954715	-117.928	7.414085	0.233
SAC07	Packing (antiparallel polar sheet)	-102.55	4.43		-3081.869408	-133.349	7.011299		-3081.955655	-118.019	7.323315	0.277
SAC08	Packing (parallel polar sheet)	-102.13	4.85		-3081.883557	-134.715	5.646119		-3081.964797	-118.901	6.441255	0.268
SAC09	Packing (parallel polar ribbon)	-101.67	5.31		-3081.76847	-123.61	16.75051		-3081.845573	-107.398	17.94482	0.456
SAC10	Packing (SO_2 dimer)	-100.91	6.07		-3081.796855	-126.349	14.01173		-3081.852362	-108.053	17.28977	0.234

FLU													
Label	Reason	ψ_{mol}			ψ_{crys}(PBE+TS)				ψ_{crys}(PBE+MBD) single point energy				ψ_{mol} vs. ψ_{crys}
		U_{latt} / kJ mol^{-1}	Relative U_{latt} / kJ mol^{-1}	$RMSD_{20}$ / Å	E / eV	U_{latt} / kJ mol^{-1}	Relative U_{latt} / kJ mol^{-1}	$RMSD_{20}$ / Å	E / eV	U_{latt} / kJ mol^{-1}	Relative U_{latt} / kJ mol^{-1}	$RMSD_{20}$ / Å
FLU01	Experimental (FURACL01, form I)	-96.5811	0	0.303	-2773.64727	-146.284	0	0.108	-2773.705522	-137.617	0	0.344
FLU02	Experimental, (FURACL03, form II) lowest energy ψ_{mol} structure, asymmetric ribbon	-102.495	-5.91353	0.252	-2773.629567	-144.576	1.708115	0.076	-2773.678978	-135.056	2.561199	0.268
FLU03	Packing, non-polar symmetric ribbon	-99.6392	-3.05805		-2773.570685	-138.895	7.389522		-2773.63715	-131.02	6.597033	0.427
FLU04	Packing, dimers	-98.3535	-1.77234		-2773.536308	-135.578	10.70644		-2773.583058	-125.801	11.81618	0.232
FLU05	Packing, antiparallel polar symmetric ribbon	-98.4885	-1.90742		-2773.571415	-138.965	7.319081		-2773.634831	-130.796	6.820738	0.179
FLU06	Lowest energy ψ_{mol} structure in Sohncke space group, antiparallel polar symmetric ribbon	-98.6039	-2.02274		-2773.574455	-139.258	7.025714		-2773.63314	-130.633	6.983946	0.278

S4.1.2 Molecule optimizations

Table S22. Gas phase molecular conformations of rigid molecules.

		**CRN**	
Conformation (dipole moment)	ψ_{mol} PBE0/6-31G(d,p) optimized	ψ_{crys}(PBE+TS) optimized	
	$E_{conf.min}(\psi_{mol})$ /H	$E_{conf.min}(\psi_{crys}$(PBE+TS)) /eV	$E_{conf.min}(\psi_{crys}$(PBE+MBD)) /eV
opt (0.0002)	-920.853132581	-3977.823652345	-3978. 548973
		**ACR**	
Conformation (dipole moment)	ψ_{mol} MP2/6-31G(d,p) optimized	ψ_{crys}(PBE+TS) optimized	
	$E_{conf.min}(\psi_{mol})$ /H	$E_{conf.min}(\psi_{crys}$(PBE+TS)) /eV	$E_{conf.min}(\psi_{crys}$(PBE+MBD)) /eV
opt (2.208)	-553.86433398381	-2470.271896586	-2470.630756
		**PTH**	
Conformation (dipole moment)	ψ_{mol} MP2/6-31G(d,p) optimized	ψ_{crys}(PBE+TS) optimized	
	$E_{conf.min}(\psi_{mol})$ /H	$E_{conf.min}(\psi_{crys}$(PBE+TS)) /eV	$E_{conf.min}(\psi_{crys}$(PBE+MBD)) /eV
opt (3.5878)	-511.62653558859	-2496.656523130	-2496.874221
		**SAC**	
Conformation (dipole moment)	ψ_{mol} PBE0/6-31G(d,p) optimized	ψ_{crys}(PBE+TS) optimized	
	$E_{conf.min}(\psi_{mol})$ /H	$E_{conf.min}(\psi_{crys}$(PBE+TS)) /eV	$E_{conf.min}(\psi_{crys}$(PBE+MBD)) /eV
opt (4.0101)	-947.568339727	-3080.487363348	-3080.732493
		**FLU**	
Conformation (dipole moment)	ψ_{mol} MP2/6-31G(d,p) optimized	ψ_{crys}(PBE+TS) optimized	
	$E_{conf.min}(\psi_{mol})$ /H	$E_{conf.min}(\psi_{crys}$(PBE+TS)) /eV	$E_{conf.min}(\psi_{crys}$(PBE+MBD)) /eV
OPT (4.5214)	-512.66327458441	-2772.131168789	-2772.279248

S4.2 Carbamazepine family

S4.2.1 Structure optimizations

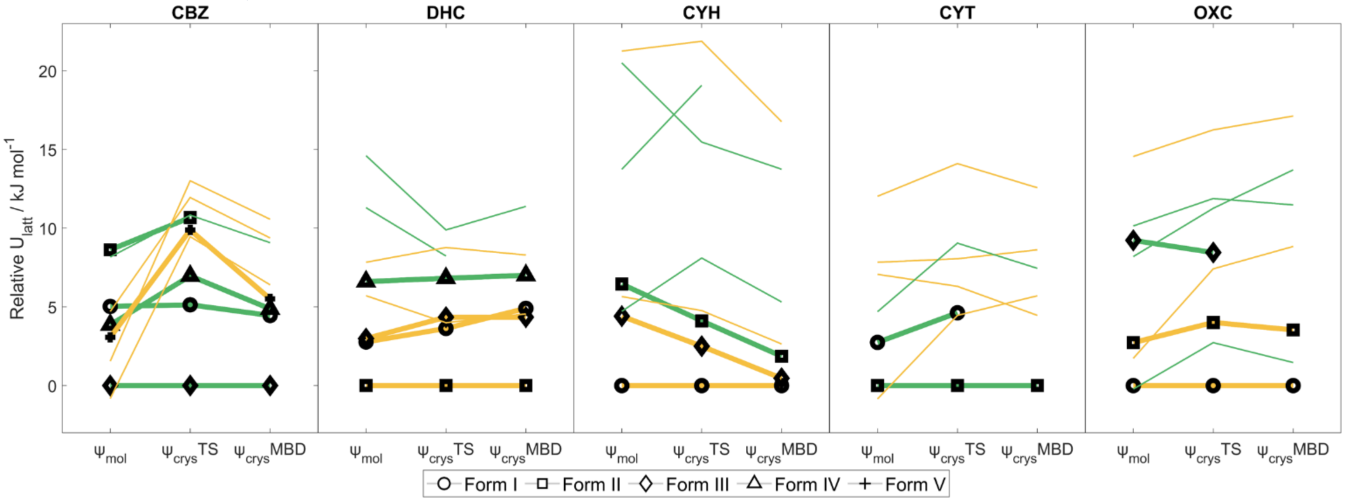

Figure S9. Relative energies of various crystal structures of CBZ, CYH, CYT, DHC and OXC, with different computational models.

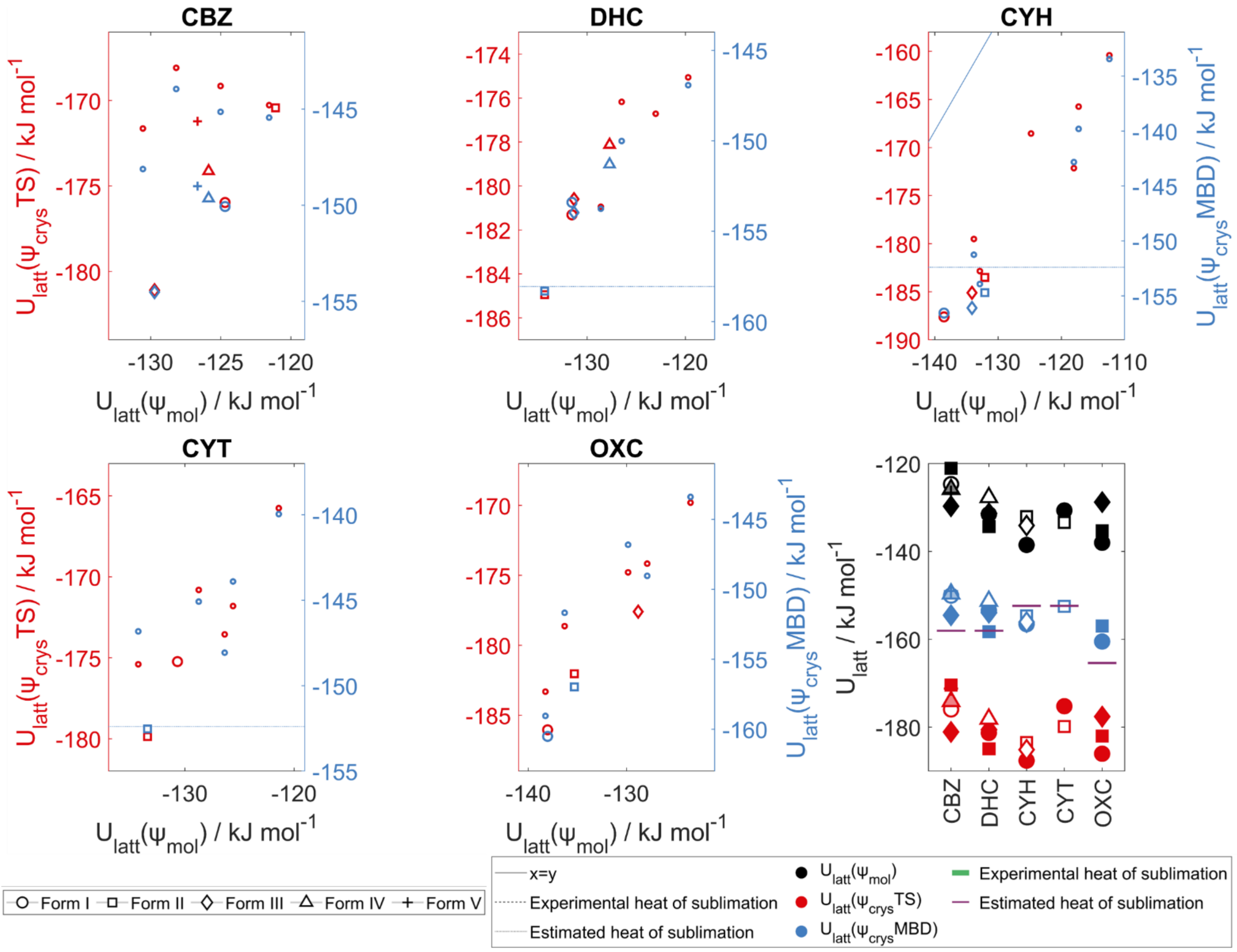

Figure S10. Comparison of lattice energies of CBZ, DHC, CYH, CYT and OXC, with different computational models. Different energy scales are used for the different computational methods, colored black for ψ_{mol}, red for ψ_{crys}(PBE+TS) and blue for ψ_{crys}(PBE+MBD). The diagonal lines in the first five tiles are the x=y relationships, colored red for ψ_{mol}=ψ_{crys}(PBE+TS) and blue for ψ_{mol}=ψ_{crys}(PBE+MBD). The dashed lines are experimental heats of sublimation (where available) and the dotted lines are estimated heats of sublimation for the atomic types, placed when the scale allows, with the relevant axis colored black (vertical lines) for ψ_{mol}, red (horizontal lines) for ψ_{crys}(PBE+TS) and blue (horizontal lines) for ψ_{crys}(PBE+MBD). Similarly, when the scales allow the x=y lines to be drawn, these are included. In the bottom right corner, points are colored black for ψ_{mol}, red for ψ_{crys}(PBE+TS) and blue for ψ_{crys}(PBE+MBD), with filled symbols denoting simple solution crystallizations, shaded symbols denoting solution crystallizations with modified conditions or additives, and open symbols denoting crystallization not from solution. The purple lines mark the estimated heats of sublimation.

Table S23. Absolute and relative energies of various crystal structures of CBZ, DHC, CYH, CYT and OXC, with different computational models.

**CBZ**												
Label	Reason	ψ_{mol}			ψ_{crys}(PBE+TS)				ψ_{crys}(PBE+MBD) single point energy			ψ_{mol} vs. ψ_{crys}
		U_{latt} / kJ mol^{-1}	Relative U_{latt} / kJ mol^{-1}	$RMSD_{20}$ / Å	E / eV	U_{latt} / kJ mol^{-1}	Relative U_{latt} / kJ mol^{-1}	$RMSD_{20}$ / Å	E / eV	U_{latt} / kJ mol^{-1}	Relative U_{latt} / kJ mol^{-1}	$RMSD_{20}$ / Å
CBZ01	Experimental (CBMZPN11, form I)	-124.695	5.03	0.194	-3551.709514	-175.967	5.132401	0.281	-3551.895316	-150.067	4.453575	0.412
CBZ02	Experimental (CBMZPN03, form II)	-121.101	8.624	0.18	-3551.652080	-170.425	10.67407	0.265				0.317
CBZ03	Experimental (CBMZPN10, form III)	-129.725	0	0.404	-3551.762707	-181.099	0	0.177	-3551.941473	-154.521	0	0.381
CBZ04	Experimental (CBMZPN12, form IV)	-125.867	3.858	0.172	-3551.690483	-174.13	6.968625	0.125	-3551.890945	-149.646	4.875319	0.244
CBZ05	Experimental (CBMZPN16, form V)	-126.658	3.067	0.366	-3551.660243	-171.213	9.886381	0.218	-3551.884362	-149.01	5.510457	0.349
CBZ06	Lowest energy ψ_{mol} structure	-130.552	-0.827		-3551.664634	-171.636	9.462722		-3551.875132	-148.12	6.401044	0.212
CBZ07	Lowest energy chiral ψ_{mol} structure	-128.178	1.547		-3551.627841	-168.086	13.01283		-3551.831975	-143.956	10.56511	0.377
CBZ08	Isostructural with CYH I	-125.018	4.707		-3551.638870	-169.15	11.94865		-3551.844296	-145.145	9.376317	0.421
CBZ09	Isostructural with DHC IV	-121.555	8.17		-3551.650478	-170.271	10.82858		-3551.847396	-145.444	9.077256	0.411
**DHC**												
Label	Reason	ψ_{mol}			ψ_{crys}(PBE+TS)				ψ_{crys}(PBE+MBD) single point energy			ψ_{mol} vs. ψ_{crys}
		U_{latt} / kJ mol^{-1}	Relative U_{latt} / kJ mol^{-1}	$RMSD_{20}$ / Å	E / eV	U_{latt} / kJ mol^{-1}	Relative U_{latt} / kJ mol^{-1}	$RMSD_{20}$ / Å	E / eV	U_{latt} / kJ mol^{-1}	Relative U_{latt} / kJ mol^{-1}	$RMSD_{20}$ / Å
DHC01	Experimental (VACTAU01, form I)	-131.555	2.772	0.242	-3584.652755	-181.313	3.630023	0.242	-3584.836489	-153.405	4.90669	0.268
DHC02	Experimental (VACTAU02, form II) lowest energy ψ_{mol} structure	-134.327	0	0.105	-3584.690377	-184.943	0	0.105	-3584.887342	-158.312	0	0.213
DHC03	Experimental (VACTAU03, form III)	-131.343	2.984	0.145	-3584.645338	-180.597	4.345729	0.145	-3584.842318	-153.967	4.344243	0.27
DHC04	Experimental (VACTAU04, form IV)	-127.721	6.606	0.131	-3584.619705	-178.124	6.818931	0.131	-3584.814743	-151.307	7.004896	0.202
DHC05	Same packing as CBZ II and CYT I, but opposite amide configuration	-123.026	11.301		-3584.605064	-176.711	8.231633					0.258
DHC06	Isostructural with CBZ III	-119.721	14.606		-3584.587914	-175.057	9.886401		-3584.769365	-146.928	11.38324	0.461
DHC07	Isostructural with CBZ06	-128.617	5.71		-3584.648967	-180.947	3.995531		-3584.840141	-153.757	4.554271	0.342
DHC08	Lowest energy chiral ψ_{mol} structure	-126.493	7.834		-3584.599480	-176.173	8.770442		-3584.801357	-150.015	8.296447	0.276
**CYH**												
Label	Reason	ψ_{mol}			ψ_{crys}(PBE+TS)				ψ_{crys}(PBE+MBD) single point energy			ψ_{mol} vs. ψ_{crys}
		U_{latt} / kJ mol^{-1}	Relative U_{latt} / kJ mol^{-1}	$RMSD_{20}$ / Å	E / eV	U_{latt} / kJ mol^{-1}	Relative U_{latt} / kJ mol^{-1}	$RMSD_{20}$ / Å	E / eV	U_{latt} / kJ mol^{-1}	Relative U_{latt} / kJ mol^{-1}	$RMSD_{20}$ / Å
CYH01	Experimental (TEVSOD, form I) lowest energy ψ_{mol} structure	-138.538	0	0.194	-3482.016707	-187.617	0	0.122	-3482.183387	-156.569	0	0.215
CYH02	Experimental (TEVSOD01, form II)	-132.091	6.447	0.172	-3481.974091	-183.505	4.111945	0.14	-3482.164093	-154.708	1.86162	0.207
CYH03	Experimental (TEVSOD02, form III)	-134.13	4.408	0.212	-3481.990802	-185.117	2.499554	0.088	-3482.178582	-156.105	0.463657	0.209

CYH04	Lowest energy chiral ψ_{mol} structure	-132.884	5.654		-3481.967332	-182.853	4.764049		-3482.155988	-153.926	2.643671	0.203
CYH05	Isostructural with DHC IV	-133.821	4.717		-3481.932731	-179.514	8.102606		-3482.128330	-151.257	5.312285	0.234
CYH06	Isostructural with CBZ06	-117.287	21.251		-3481.790005	-165.743	21.8738		-3482.009637	-139.805	16.76462	0.291
CYH07	Isostructural with CBZ III	-118.038	20.5		-3481.856352	-172.145	15.47221		-3482.040943	-142.825	13.74407	0.194
CYH08	Isostructural with CBZ II	-124.798	13.74		-3481.819032	-168.544	19.07313					0.212
CYH09	Isostructural with CBZ IV	-112.438	26.1		-3481.734648	-160.402	27.21505		-3481.943685	-133.441	23.12813	0.538
**CYT**												
Label	Reason	ψ_{mol}			ψ_{crys}(PBE+TS)				ψ_{crys}(PBE+MBD) single point energy			ψ_{mol} vs. ψ_{crys}
		U_{latt} / kJ mol^{-1}	Relative U_{latt} / kJ mol^{-1}	$RMSD_{20}$ / Å	E / eV	U_{latt} / kJ mol^{-1}	Relative U_{latt} / kJ mol^{-1}	$RMSD_{20}$ / Å	E / eV	U_{latt} / kJ mol^{-1}	Relative U_{latt} / kJ mol^{-1}	$RMSD_{20}$ / Å
CYT01	Experimental (SOGLEG, form I)	-130.682	2.744	0.209	-3449.070534	-175.236	4.624838	0.181				0.308
CYT02	Experimental (SODNOP, form II)	-133.426	0	0.244	-3449.118466	-179.861	0	0.35	-3449.310053	-152.529	0	0.253
CYT03	Lowest energy ψ_{mol} structure	-134.275	-0.849		-3449.072251	-175.402	4.459145		-3449.250919	-146.823	5.705627	0.188
CYT04	Lowest energy chiral ψ_{mol}l structure	-126.358	7.068		-3449.053116	-173.555	6.305402		-3449.263841	-148.07	4.458894	0.2
CYT05	Chain contact	-125.593	7.833		-3449.034979	-171.805	8.055442		-3449.220664	-143.904	8.624865	0.287
CYT06	Chain contact	-121.402	12.024		-3448.972302	-165.758	14.10291		-3449.179799	-139.961	12.56777	0.394
CYT07	Isostructural with DHC IV	-128.735	4.691		-3449.024669	-170.811	9.050138		-3449.232858	-145.081	7.448302	0.204
**OXC**												
Label	Reason	ψ_{mol}			ψ_{crys}(PBE+TS)				ψ_{crys}(PBE+MBD) single point energy			ψ_{mol} vs. ψ_{crys}
		U_{latt} / kJ mol^{-1}	Relative U_{latt} / kJ mol^{-1}	$RMSD_{20}$ / Å	E / eV	U_{latt} / kJ mol^{-1}	Relative U_{latt} / kJ mol^{-1}	$RMSD_{20}$ / Å	E / eV	U_{latt} / kJ mol^{-1}	Relative U_{latt} / kJ mol^{-1}	$RMSD_{20}$ / Å
OXC01	Experimental (CANDUR01, form I)	-138.034	0	0.384	-3991.195666	-186.046	0	0.171	-3991.411161	-160.516	0	0.266
OXC02	Experimental (CANDUR02, form II) lowest energy chiral ψ_{mol} structure	-135.303	2.731	0.515	-3991.154103	-182.036	4.010335	0.304	-3991.374534	-156.982	3.533981	0.267
OXC03	Experimental (CANDUR03, form III)	-128.807	9.227	0.544	-3991.108098	-177.597	8.449172	0.471				0.230
OXC04	Lowest energy ψ_{mol} structure	-138.255	-0.221		-3991.167377	-183.317	2.72958		-3991.395991	-159.053	1.463708	0.502
OXC05	Isostructural with CBZ III	-129.838	8.196		-3991.078949	-174.785	11.26172		-3991.269127	-146.812	13.70441	0.433
OXC06	Isostructural with CBZ V	-136.301	1.733		-3991.118812	-178.631	7.415487		-3991.319559	-151.678	8.83833	0.181
OXC07	Isostructural with CBZ06	-123.479	14.555		-3991.027299	-169.801	16.24529		-3991.233677	-143.391	17.1249	
OXC08	Isostructural with DHC IV	-127.892	10.142		-3991.072497	-174.162	11.88426		-3991.292192	-149.037	11.47896	

S4.2.2 Molecule optimizations

"In" and "Out" refer to the pyramidality of the NH_2 group ("In" denotes that the hydrogen atoms are both towards the rest of the molecule). "Syn" and "Anti" apply only to CYH, DHC and OXC, and refer to the sp^3 hybridized carbon atoms of the central ring ("Syn" denotes that the CH_2 group which is closest to the amide ("Up") is on the same side of the molecule as the carbonyl group).

In cases where the conformation changed in GAUSSIAN, the conformation was reset in MOLDEN (to 160 and 20°) before optimization with CASTEP. Conformations where the optimized molecule no longer matches the definition given by the name are colored grey in Table S24.

Table S24. Gas phase molecular conformations of Carbamazepine family molecules. Pyramidality is the $C\_N\_H_{up}\_H_{side}$ improper dihedral angle when viewed with the amide on the right (the closer this is to ±180 ° the flatter the functional group). The row colored yellow for each molecule is the lowest energy conformation, on which the box size convergence was tested.

**CBZ**					
Conformation (dipole moment)	ψ_{mol} PBE/6-31G(d,p) optimized		ψ_{crys}(PBE+TS) optimized		
	pyramidality / °	$E_{conf.min}(\psi_{mol})$ /H ($\Delta E_{conf.min}$ / kJ mol^{-1})	pyramidality / °	$E_{conf.min}(\psi_{crys}$(PBE+TS)) /eV ($\Delta E_{conf.min}$ / kJ mol^{-1})	$E_{conf.min}(\psi_{crys}$(PBE+TS)) /eV ($\Delta E_{conf.min}$ / kJ mol^{-1})
In (3.2885)	140.973 (In)	-762.652979584 (0)	146.52 (In)	-3549.885779 (0)	-3550.340004 (0)
Out (3.487)	-154.593 (Out)	-762.652100604 (2.31)	-156.20 (Out)	-3549.874653 (1.07)	-3550.327339 (1.22)
**DHC**					
Conformation (dipole moment)	ψ_{mol} PBE/6-31G(d,p) optimized		ψ_{crys}(PBE+TS) optimized		
	pyramidality / °	$E_{conf.min}(\psi_{mol})$ /H ($\Delta E_{conf.min}$ / kJ mol^{-1})	pyramidality / °	$E_{conf.min}(\psi_{crys}$(PBE+TS)) /eV ($\Delta E_{conf.min}$ / kJ mol^{-1})	$E_{conf.min}(\psi_{crys}$(PBE+TS)) /eV ($\Delta E_{conf.min}$ / kJ mol^{-1})
AntiIn (3.8877)	144.049 (In)	-763.860955118 (0)	150.42 (In)	-3582.773611 (0)	-3583.246587 (0)
AntiOut (3.8714)	-148.436 (Out)	-763.860677792 (0.73)	-151.48 (Out)	-3582.77334 (0.03)	-3583.244982 (0.15)
SynIn (3.5733)	142.931 (In)	-763.860106339 (2.23)	149.23 (In)	-3582.757848 (1.52)	-3583.225129 (2.07)
SynOut (3.6306)	-151.931 (Out)	-763.859604595 (3.55)	-153.15 (Out)	-3582.755096 (1.79)	-3583.221525 (2.42)
**CYH**					
Conformation (dipole moment)	ψ_{mol} PBE/6-31G(d,p) optimized		ψ_{crys}(PBE+TS) optimized		
	pyramidality / °	$E_{conf.min}(\psi_{mol})$ /H ($\Delta E_{conf.min}$ / kJ mol^{-1})	pyramidality / °	$E_{conf.min}(\psi_{crys}$(PBE+TS)) /eV ($\Delta E_{conf.min}$ / kJ mol^{-1})	$E_{conf.min}(\psi_{crys}$(PBE+TS)) /eV ($\Delta E_{conf.min}$ / kJ mol^{-1})
AntiIn (3.3127)	164.533 (In)	-747.819360130 (0.94)	168.38 (In)	-3480.058947 (1.29)	-3480.547025 (1.32)
AntiOut (3.3128)	164.546 (converted to In)	-747.819360116 (0.94)	168.41 (Converted to In)	-3480.059004 (1.29)	-
SynIn (3.4552)	174.357 (In)	-747.819719346 (0)	179.16 (Planar)	-3480.072229 (0.01)	-3480.560691 (0)
SynOut (3.4548)	174.370 (converted to In)	-747.819719256 (0)	-179.19 (Planar)	-3480.072323 (0)	-
**CYT**					
Conformation (dipole moment)	ψ_{mol} PBE/6-31G(d,p) optimized		ψ_{crys}(PBE+TS) optimized		
	pyramidality / °	$E_{conf.min}(\psi_{mol})$ /H ($\Delta E_{conf.min}$ / kJ mol^{-1})	pyramidality / °	$E_{conf.min}(\psi_{crys}$(PBE+TS)) /eV ($\Delta E_{conf.min}$ / kJ mol^{-1})	$E_{conf.min}(\psi_{crys}$(PBE+TS)) /eV ($\Delta E_{conf.min}$ / kJ mol^{-1})
In (3.0758)	158.363 (In)	-746.614828389 (0)	164.04 (In)	-3447.254372 (0)	-3447.729230 (0)
Out (3.0754)	158.254 (converted to In)	-746.614828451 (0)	164.21 (converted to In)	-3447.254399 (0)	-
**OXC**					
Conformation (dipole moment)	ψ_{mol} PBE/6-31G(d,p) optimized		ψ_{crys}(PBE+TS) optimized		
	pyramidality / °	$E_{conf.min}(\psi_{mol})$ /H ($\Delta E_{conf.min}$ / kJ mol^{-1})	pyramidality / °	$E_{conf.min}(\psi_{crys}$(PBE+TS)) /eV ($\Delta E_{conf.min}$ / kJ mol^{-1})	$E_{conf.min}(\psi_{crys}$(PBE+TS)) /eV ($\Delta E_{conf.min}$ / kJ mol^{-1})

AntiIn (3.9503)	147.030 (In)	-837.819308375 (0)	154.85 (In)	-3989.267464 (0)	-3989.747555 (0)
AntiOut (4.5165)	-155.973 (Out)	-837.818933461 (0.98)	-158.34 (Out)	-3989.26555 (0.18)	-3989.744772 (0.27)
SynIn (2.6898)	144.484 (In)	-837.817622506 (4.43)	150.40 (In)	-3989.222705 (4.32)	-3989.697369 (4.84)
SynOut (2.6945)	144.686 (converted to In)	-837.817622660 (4.43)	150.26 (Converted to In)	-3989.222526 (4.34)	-

S4.3 Fenamate Family

S4.3.1 Structure optimizations

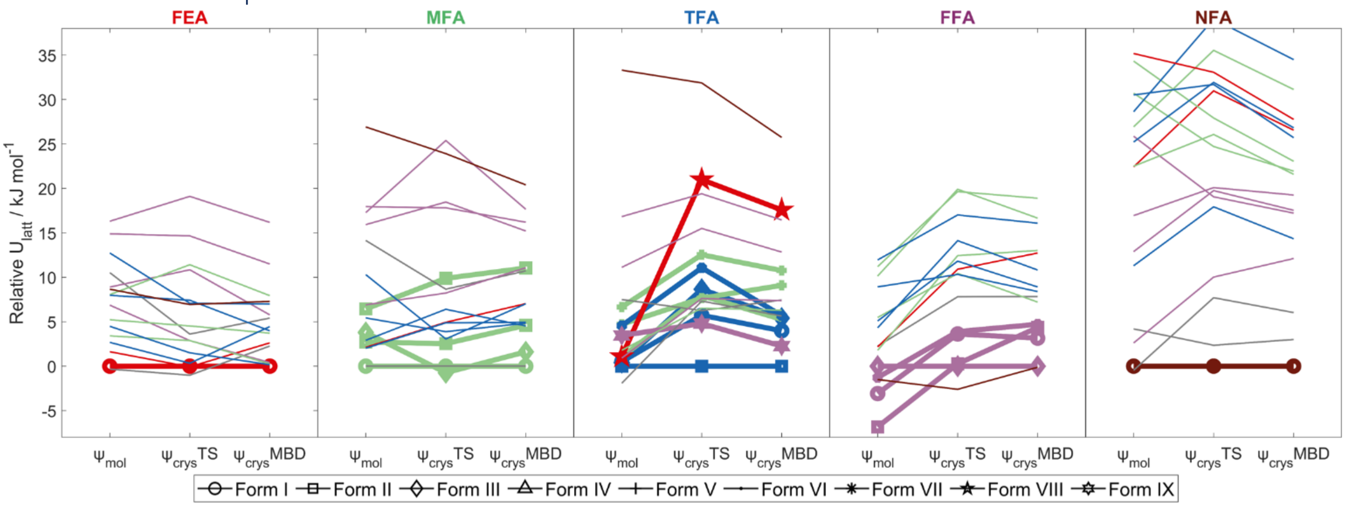

Figure S11. Relative energies of various crystal structures of FEA, MFA, TFA, FFA and NFA, with different computational models. The lines are colored by the molecular color of the experimentally observed packing (FEA=red, MFA=green, TFA=blue, FFA=lilac, NFA=brown), except when there is an isostructural relationship, in which case the color is that of the molecule which was the earliest deposited on the CSD to exhibit that packing. Uncolored lines are as yet unobserved packings.

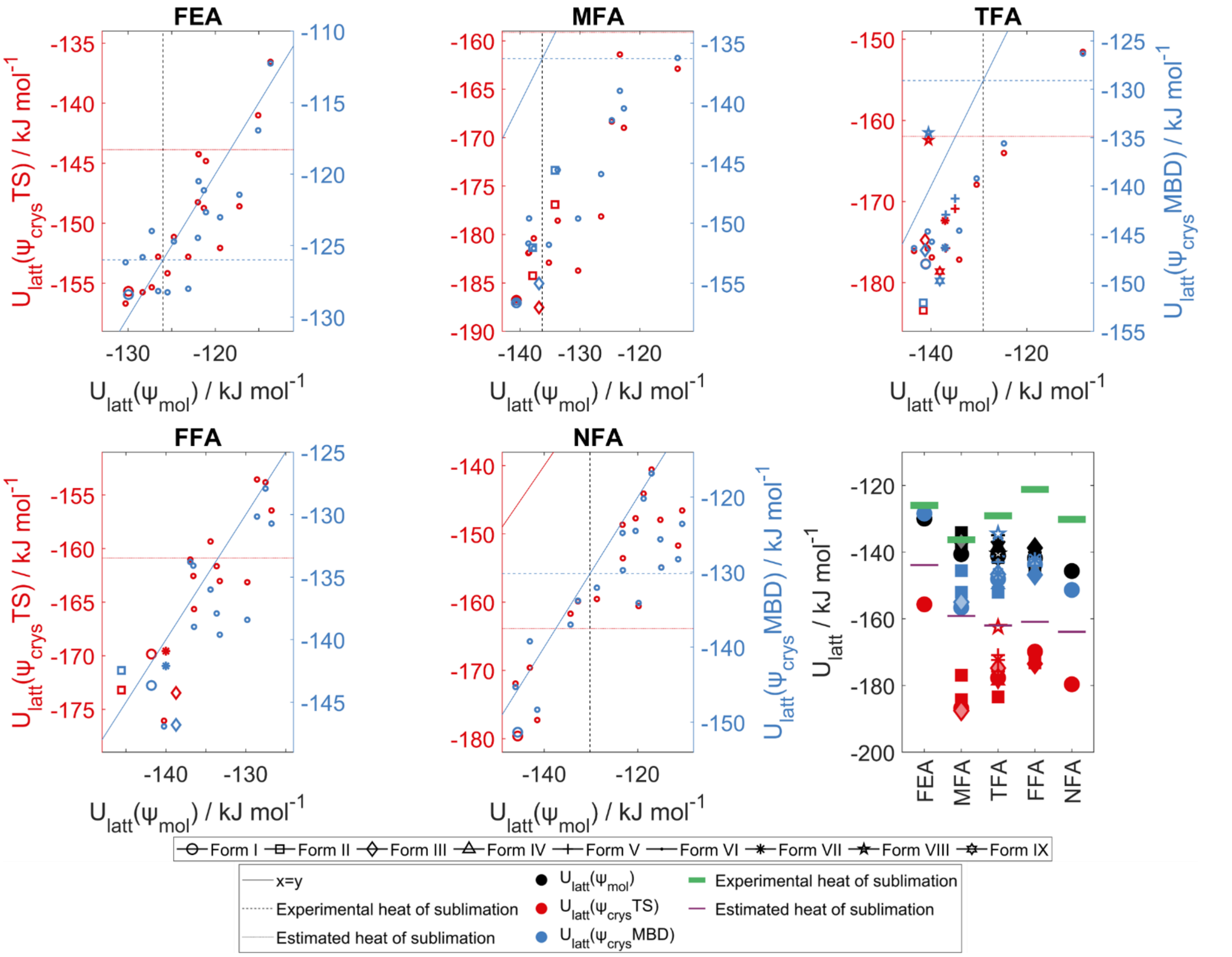

Figure S12. Comparison of lattice energies of FEA, MFA, TFA, FFA and NFA, with different computational models. Different energy scales are used for the different computational methods, colored black for ψ_{mol}, red for ψ_{crys}(PBE+TS) and blue for ψ_{crys}(PBE+MBD). The diagonal lines in the first five tiles are the x=y relationships, colored red for ψ_{mol}=ψ_{crys}(PBE+TS) and blue for ψ_{mol}=ψ_{crys}(PBE+MBD). The dashed lines are experimental heats of sublimation (where available) and the dotted lines are estimated heats of sublimation for the atomic types, placed when the scale allows, with the relevant axis colored black (vertical lines) for ψ_{mol}, red (horizontal lines) for ψ_{crys}(PBE+TS) and blue (horizontal lines) for ψ_{crys}(PBE+MBD). Similarly, when the scales allow the x=y lines to be drawn, these are included. In the bottom right corner, points are colored black for ψ_{mol}, red for ψ_{crys}(PBE+TS) and blue for ψ_{crys}(PBE+MBD), with filled symbols denoting simple solution crystallizations, shaded symbols denoting solution crystallizations with modified conditions or additives, and open symbols denoting crystallization not from solution. The green lines mark the experimental heats of sublimation and the purple lines mark the estimated heats of sublimation.

Table S25. Absolute and relative energies of various crystal structures of FEA, MFA, TFA, FFA and NFA, with different computational models.

**FEA**												
Label	Reason	ψ_{mol}			ψ_{crys}(PBE+TS)				ψ_{crys}(PBE+MBD) single point energy			ψ_{mol} vs. ψ_{crys}
		U_{latt} / kJ mol^{-1}	Relative U_{latt} / kJ mol^{-1}	RMSD$_{20}$ / Å	E / eV	U_{latt} / kJ mol^{-1}	Relative U_{latt} / kJ mol^{-1}	RMSD$_{20}$ / Å	E / eV	U_{latt} / kJ mol^{-1}	Relative U_{latt} / kJ mol^{-1}	RMSD$_{20}$ / Å
FEA01	Experimental (QQQBTY02, RT, form I)	-129.968	0	0.422	-3383.361014	-155.662	0	0.173	-3383.471930	-128.43	0	0.509
FEA02	Close match to FEA I; Isostructural with TFA VIII	-128.344	1.624		-3383.361812	-155.739	-0.07701		-3383.444771	-125.81	2.620442	0.908
FEA03	Lowest energy ψ_{mol} structure	-130.297	-0.329		-3383.371574	-156.681	-1.01892		-3383.448470	-126.167	2.263513	0.937
FEA04	Lowest energy chiral ψ_{mol} structure	-119.447	10.521		-3383.323635	-152.056	3.606556		-3383.415862	-123.021	5.409785	0.824
FEA05	Isostructural with FFA I and TFA IX	-123.103	6.865		-3383.331223	-152.788	2.874392		-3383.467694	-128.022	0.408671	0.293
FEA06	Constructed from FFA II	-115.079	14.889		-3383.209113	-141.006	14.65647		-3383.352837	-116.939	11.49093	1.192↑
FEA07	Constructed from FFA III	-121.076	8.892		-3383.248615	-144.817	10.84502		-3383.412160	-122.663	5.76698	0.205
FEA08	Constructed from FFA VII	-113.665	16.303		-3383.163026	-136.559	19.10324		-3383.304303	-112.257	16.1738	0.788↑
FEA09	Isostructural with MFA I and TFA VI	-126.557	3.411		-3383.331215	-152.787	2.875248		-3383.469367	-128.183	0.247296	0.54
FEA10	Constructed from MFA II_maj	-121.913	8.055		-3383.242734	-144.25	11.41251		-3383.389750	-120.501	7.929253	0.504
FEA11	Constructed from MFA II_min	-121.924	8.044		-3383.242779	-144.254	11.40816		-3383.389714	-120.498	7.932775	0.32
FEA12	Constructed from MFA III	-124.75	5.218		-3383.314138	-151.14	4.522946		-3383.433405	-124.713	3.717162	0.985↑
FEA13	Isostructural with TFA I	-127.291	2.677		-3383.357592	-155.332	0.330153		-3383.425813	-123.981	4.449667	1.12↑
FEA14	Constructed from TFA II	-117.237	12.731		-3383.287689	-148.588	7.074876		-3383.399508	-121.443	6.987782	0.199
FEA15	Constructed from TFA III	-122	7.968		-3383.284131	-148.244	7.418176		-3383.430766	-124.459	3.971779	0.668↑
FEA16	Isostructural with TFA VII	-125.485	4.483		-3383.345445	-154.16	1.502208		-3383.470244	-128.268	0.162629	0.293
FEA17	Constructed from NFA I	-121.311	8.657		-3383.289174	-148.731	6.931614		-3383.396319	-121.135	7.295406	0.699
**MFA**												
Label	Reason	ψ_{mol}			ψ_{crys}(PBE+TS)				ψ_{crys}(PBE+MBD) single point energy			ψ_{mol} vs. ψ_{crys}
		U_{latt} / kJ mol^{-1}	Relative U_{latt} / kJ mol^{-1}	RMSD$_{20}$ / Å	E / eV	U_{latt} / kJ mol^{-1}	Relative U_{latt} / kJ mol^{-1}	RMSD$_{20}$ / Å	E / eV	U_{latt} / kJ mol^{-1}	Relative U_{latt} / kJ mol^{-1}	RMSD$_{20}$ / Å
MFA01	Experimental (XYANAC06, form I)	-140.619	0	0.178	-3763.771021	-186.792	0	0.19	-3763.932913	-156.625	0	0.319
MFA02	Experimental (XYANAC05-maj, form II)	-137.911	2.708	0.429	-3763.744724	-184.254	2.537287	0.206	-3763.885315	-152.032	4.592636	0.336
MFA03	Experimental (XYANAC05-min, form II)	-134.184	6.435	0.575↔	-3763.668583	-176.908	9.88396	0.229↔	-3763.818479	-145.584	11.04139	0.555
MFA04	Experimental (XYANAC03, form III)	-136.836	3.783	0.541	-3763.778674	-187.53	-0.73837	0.917	-3763.916216	-155.014	1.611043	0.529
MFA05	Lowest energy ψ_{mol} structure. NB. This is not the lowest energy structure following ψ_{mol} reoptimization with consistent methodology. See Section S2.3.5.	-140.606	0.013		-3763.770736	-186.764	0.02749		-3763.932713	-156.606	0.019346	0.377
MFA06	Lowest energy chiral ψ_{mol} structure	-126.481	14.138		-3763.681298	-178.135	8.657127		-3763.821801	-145.904	10.72091	0.674
MFA07	Isostructural with FEA I and TFA VIII	-138.503	2.116		-3763.719900	-181.859	4.932507		-3763.860073	-149.597	7.028113	0.324
MFA08	Isostructural with FFA I and TFA IX	-123.34	17.279		-3763.507915	-161.405	25.38626		-3763.749959	-138.972	17.65266	0.615

MFA09	Isostructural with FFA II	-133.721	6.898		-3763.685767	-178.566	8.225893		-3763.818205	-145.557	11.06781	0.235
MFA10	Close match to FFA III	-124.695	15.924		-3763.579671	-168.329	18.46282		-3763.775334	-141.421	15.2043	0.47
MFA11	Constructed from FFA VII	-122.673	17.946		-3763.586406	-168.979	17.81297		-3763.765092	-140.432	16.19257	0.398
MFA12	Isostructural with TFA I	-135.196	5.423		-3763.730796	-182.91	3.881204		-3763.882817	-151.791	4.833613	0.327
MFA13	Isostructural with TFA II	-130.323	10.296		-3763.739292	-183.73	3.061427		-3763.860190	-149.608	7.016872	0.393
MFA14	Isostructural with TFA III	-138.607	2.012		-3763.720532	-181.92	4.871531		-3763.881648	-151.679	4.946406	0.219
MFA15	Isostructural with TFA VII	-137.711	2.908		-3763.704713	-180.394	6.397823		-3763.885973	-152.096	4.529124	0.321
MFA16	Isostructural with NFA I	-113.707	26.912		-3763.523201	-162.88	23.91143		-3763.721529	-136.229	20.39578	0.343
**TFA**												
Label	Reason	ψ_{mol}			ψ_{crys}(PBE+TS)				ψ_{crys}(PBE+MBD) single point energy			ψ_{mol} vs. ψ_{crys}
		U_{latt} / kJ mol^{-1}	Relative U_{latt} / kJ mol^{-1}	$RMSD_{20}$ / Å	E / eV	U_{latt} / kJ mol^{-1}	Relative U_{latt} / kJ mol^{-1}	$RMSD_{20}$ / Å	E / eV	U_{latt} / kJ mol^{-1}	Relative U_{latt} / kJ mol^{-1}	$RMSD_{20}$ / Å
TFA01	Experimental (KAXXAI01, form I)	-141.103	0.499	0.313	-3979.959733	-177.686	5.72699	0.11	-3980.111285	-148.065	3.97973	0.284
TFA02	Experimental (KAXXAI, form II)	-141.602	0	0.279	-3980.019088	-183.413	0	0.107	-3980.152531	-152.045	0	0.265
TFA03	Experimental (KAXXAI02, form III)	-141.205	0.397	0.299	-3979.929368	-174.756	8.65685	0.279	-3980.096556	-146.644	5.40086	0.238
TFA04	Experimental (KAXXAI04-maj, form V)	-134.952	6.65	0.197↔	-3979.889182	-170.879	12.53423	0.164↔	-3980.041054	-141.288	10.75613	0.219
TFA05	Poor match to experimental (KAXXAI04-min, form V)	-136.898	4.704	1.656↔	-3979.939753	-175.758	7.65478	1.742↔	-3980.058332	-142.955	9.08903	3.163↑
TFA06	Experimental (KAXXAI07-maj, form VI)	-140.579	1.023	0.448	-3979.936933	-175.486	7.92684	0.683	-3980.098038	-146.787	5.25791	0.369
TFA07	Experimental (KAXXAI05, form VII)	-137.05	4.552	0.875	-3979.904367	-172.344	11.06909	0.855	-3980.093627	-146.361	5.68349	0.167
TFA08	Experimental (KAXXAI06, form VIII)	-140.521	1.081	0.683	-3979.801613	-162.429	20.98350	0.268	-3979.970366	-134.468	17.57655	0.47
TFA09	Experimental (KAXXAI11, form IX)	-138.152	3.45	0.193	-3979.969448	-178.623	4.78958	0.134	-3980.129102	-149.784	2.26064	0.185
TFA10	Lowest energy ψ_{mol} structure	-143.525	-1.923		-3979.943194	-176.09	7.32276		-3980.094063	-146.403	5.64138	0.17
TFA11	Lowest energy chiral ψ_{mol} structure	-134.102	7.5		-3979.954328	-177.164	6.24845		-3980.075417	-144.604	7.44047	0.691
TFA12	Isostructural with FFA I	-140.685	0.917		-3979.940249	-175.806	7.60696		-3980.076247	-144.684	7.36041	0.302
TFA13	Close match to FFA III	-124.776	16.826		-3979.818089	-164.019	19.39376		-3979.982049	-135.595	16.44934	N/A
TFA14	Constructed from FFA VII	-130.485	11.117		-3979.858515	-167.92	15.49317		-3980.019506	-139.209	12.83516	0.389
TFA15	Close match to MFA III	-139.817	1.785		-3979.951390	-176.881	6.53196		-3980.087260	-145.747	6.29785	0.228
TFA16	Constructed from NFA I	-108.301	33.301		-3979.688840	-151.548	31.86459		-3979.885795	-126.308	25.73653	0.308
TFA17	Experimental (KAXXAI03, form IV)	-140.639	0.963	0.601↑								
**FFA**												
Label	Reason	ψ_{mol}			ψ_{crys}(PBE+TS)				ψ_{crys}(PBE+MBD) single point energy			ψ_{mol} vs. ψ_{crys}
		U_{latt} / kJ mol^{-1}	Relative U_{latt} / kJ mol^{-1}	$RMSD_{20}$ / Å	E / eV	U_{latt} / kJ mol^{-1}	Relative U_{latt} / kJ mol^{-1}	$RMSD_{20}$ / Å	E / eV	U_{latt} / kJ mol^{-1}	Relative U_{latt} / kJ mol^{-1}	$RMSD_{20}$ / Å
FFA01	Experimental (FPAMCA18, 90 K, form I)	-141.817	-3.084	0.255	-5510.444873	-169.839	3.617561	0.248	-5510.632783	-143.66	3.14866	0.144
FFA02	Experimental (FPAMCA17, 95 K, form II) lowest energy ψ_{mol} structure	-145.566	-6.833	0.125	-5510.479715	-173.201	0.255719	0.218	-5510.620295	-142.455	4.353614	0.234
FFA03	Experimental (FPAMCA19, 90 K, form III)	-138.733	0	0.147	-5510.482366	-173.457	0	0.151	-5510.665416	-146.809	0	0.19

FFA04	Experimental (FPAMCA20, 100 K, form VII)	-140.012	-1.279	0.234	-5510.442072	-169.569	3.887856	0.261	-5510.616569	-142.096	4.7131	0.204
FFA05	Lowest energy chiral ψ_{mol} structure	-136.478	2.255		-5510.401442	-165.649	7.808072		-5510.584205	-138.973	7.835806	0.168
FFA06	Isostructural with FEA I and TFA VIII	-136.57	2.163		-5510.369411	-162.558	10.89866		-5510.533504	-134.081	12.72777	0.264
FFA07	Isostructural with MFA I and TFA VI	-133.258	5.475		-5510.374320	-163.032	10.42499		-5510.590718	-139.602	7.207362	0.812
FFA08	Constructed from MFA II_maj	-127.538	11.195		-5510.278832	-153.819	19.63841		-5510.469624	-127.918	18.89141	0.252
FFA09	Constructed from MFA II_min	-136.952	1.781		-5510.353522	-161.025	12.43174		-5510.530459	-133.787	13.02157	0.284
FFA10	Constructed from MFA III	-128.594	10.139		-5510.276091	-153.554	19.90286		-5510.492837	-130.157	16.65165	0.806
FFA11	Isostructural with TFA I	-126.783	11.95		-5510.306052	-156.445	17.01195		-5510.498659	-130.719	16.08988	1.08↑
FFA12	Isostructural with TFA II	-129.815	8.918		-5510.375434	-163.139	10.31748		-5510.578412	-138.414	8.394779	2.236↑
FFA13	Isostructural with TFA III	-134.418	4.315		-5510.336065	-159.341	14.11608		-5510.553334	-135.995	10.81443	0.354
FFA14	Isostructural with TFA VII	-133.65	5.083		-5510.359943	-161.645	11.81216		-5510.573096	-137.901	8.907656	0.509
FFA15	Isostructural with NFA I	-140.238	-1.505		-5510.509366	-176.062	-2.60516		-5510.666692	-146.932	-0.12309	0.94↑
FFA16	Experimental (FPAMCA15, form IV)	-135.825	2.908	0.324↑								
FFA17	Experimental (FPAMCA16, form V)	-134.828	3.905	0.29								
FFA18	Experimental (FPAMCA14, form VI)	-135.631	3.102	0.281								

NFA

Label	Reason	ψ_{mol}			ψ_{crys}(PBE+TS)				ψ_{crys}(PBE+MBD) single point energy			ψ_{mol} vs. ψ_{crys}
		U_{latt} / kJ mol^{-1}	Relative U_{latt} / kJ mol^{-1}	$RMSD_{20}$ / Å	E / eV	U_{latt} / kJ mol^{-1}	Relative U_{latt} / kJ mol^{-1}	$RMSD_{20}$ / Å	E / eV	U_{latt} / kJ mol^{-1}	Relative U_{latt} / kJ mol^{-1}	$RMSD_{20}$ / Å
NFA01	Experimental (NIFLUM11, form I)	-145.67	0	0.127	-5613.241780	-179.625	0	0.24	-5613.382790	-151.33	0	0.316
NFA02	Lowest energy ψ_{mol} structure	-146.135	-0.465		-5613.161953	-171.922	7.702247		-5613.320320	-145.303	6.027543	0.156
NFA03	Lowest energy chiral ψ_{mol} structure	-141.481	4.189		-5613.217432	-177.275	2.349327		-5613.351702	-148.331	2.999515	0.509
NFA04	Constructed from FEA I	-123.251	22.419		-5612.920795	-148.654	30.97093		-5613.107620	-124.78	26.5503	0.436
NFA05	Constructed from FFA I	-132.78	12.89		-5613.037083	-159.874	19.75059		-5613.201083	-133.798	17.53232	0.124
NFA06	Isostructural with FFA II	-143.06	2.61		-5613.137918	-169.603	10.02135		-5613.257286	-139.221	12.10948	0.125
NFA07	Constructed from FFA III	-119.804	25.866		-5613.044324	-160.573	19.05193		-5613.204340	-134.112	17.21803	0.204
NFA08	Constructed from FFA VII	-128.73	16.94		-5613.033584	-159.536	20.08818		-5613.183223	-132.075	19.25557	0.186
NFA09	Constructed from MFA I	-123.175	22.495		-5612.971628	-153.558	26.06617		-5613.159051	-129.742	21.58786	0.422
NFA10	Constructed from MFA II_maj	-118.743	26.927		-5612.873542	-144.094	35.53016		-5613.060159	-120.201	31.12965	0.267
NFA11	Constructed from MFA II_min	-114.959	30.711		-5612.985656	-154.912	24.71263		-5613.155375	-129.388	21.94259	2.263↑
NFA12	Constructed from MFA III	-111.35	34.32		-5612.952423	-151.705	27.91922		-5613.143880	-128.279	23.05166	1.699↑
NFA13	Constructed from TFA I	-117.041	28.629		-5612.836955	-140.564	39.06037		-5613.025350	-116.842	34.48831	0.19
NFA14	Constructed from TFA II	-134.371	11.299		-5613.055982	-161.697	17.92715		-5613.234222	-136.995	14.33486	0.253
NFA15	Constructed from TFA III	-120.452	25.218		-5612.910913	-147.7	31.92441		-5613.104826	-124.51	26.81983	0.395
NFA16	Constructed from TFA VII	-115.157	30.513		-5612.913319	-147.932	31.69228		-5613.116373	-125.624	25.70578	0.247
NFA17	Constructed from TFA VIII	-110.497	35.173		-5612.899112	-146.562	33.06303		-5613.095045	-123.567	27.76365	0.563

↑ denotes structures which could only be matched if the tolerances in Mercury were increased

↔ denotes structures which could only be matched if compared with ordered versions of the experimental crystal structures

N/A denotes structures which could not be matched even if tolerances were increased to 100% and 100°

S4.3.2 Molecule optimizations

Table S26. Gas phase molecular conformations of Fenamate family molecules. "A" denotes that substituents are closer to the amine and acid groups and "B" denotes that they are further away. The row colored yellow for each molecule is the lowest energy conformation, on which the box size convergence was tested.

**FEA**			
Conformation (dipole moment)	ψ_{mol} PBE/6-31G+(d) optimized	ψ_{crys}(PBE+TS) optimized	
	$E_{conf.min}(\psi_{mol})$ /H ($\Delta E_{conf.min}$ / kJ mol^{-1})	$E_{conf.min}(\psi_{crys}$(PBE+TS)) /eV ($\Delta E_{conf.min}$ / kJ mol^{-1})	$E_{conf.min}(\psi_{crys}$(PBE+TS)) /eV ($\Delta E_{conf.min}$ / kJ mol^{-1})
opt (0.8487)	-706.409661645 (0)	-3381.747714164 (0)	-3382.140866 (0)
**MFA**			
Conformation (dipole moment)	ψ_{mol} PBE/6-31G+(d) optimized	ψ_{crys}(PBE+TS) optimized	
	$E_{conf.min}(\psi_{mol})$ /H ($\Delta E_{conf.min}$ / kJ mol^{-1})	$E_{conf.min}(\psi_{crys}$(PBE+TS)) /eV ($\Delta E_{conf.min}$ / kJ mol^{-1})	$E_{conf.min}(\psi_{crys}$(PBE+TS)) /eV ($\Delta E_{conf.min}$ / kJ mol^{-1})
A (0.7095)	-784.931023845 (0)	-3761.835094947 (0)	-3762.309638 (0)
B (1.5139)	-784.930033083 (2.60)	-3761.83185112 (0.31)	-3762.287368 (2.20)
**TFA**			
Conformation (dipole moment)	ψ_{mol} PBE/6-31G+(d) optimized	ψ_{crys}(PBE+TS) optimized	
	$E_{conf.min}(\psi_{mol})$ /H ($\Delta E_{conf.min}$ / kJ mol^{-1})	$E_{conf.min}(\psi_{crys}$(PBE+TS)) /eV ($\Delta E_{conf.min}$ / kJ mol^{-1})	$E_{conf.min}(\psi_{crys}$(PBE+TS)) /eV ($\Delta E_{conf.min}$ / kJ mol^{-1})
A (2.2034)	-1205.07420645 (0)	-3978.118180241 (0)	-3978.576728 (0)
B (1.2052)	-1205.07312378 (2.84)	-3978.107263509 (1.05)	-3978.545329 (3.20)
**FFA**			
Conformation (dipole moment)	ψ_{mol} PBE/6-31G+(d) optimized	ψ_{crys}(PBE+TS) optimized	
	$E_{conf.min}(\psi_{mol})$ /H ($\Delta E_{conf.min}$ / kJ mol^{-1})	$E_{conf.min}(\psi_{crys}$(PBE+TS)) /eV ($\Delta E_{conf.min}$ / kJ mol^{-1})	$E_{conf.min}(\psi_{crys}$(PBE+TS)) /eV ($\Delta E_{conf.min}$ / kJ mol^{-1})
A (4.1318)	-1043.16243659 (0.78)	-5508.67147433 (1.27)	-5509.129489 (1.39)
B (2.8517)	-1043.16273677 (0)	-5508.684642589 (0)	-5509.143874 (0)
**NFA**			
Conformation (dipole moment)	ψ_{mol} PBE/6-31G+(d) optimized	ψ_{crys}(PBE+TS) optimized	
	$E_{conf.min}(\psi_{mol})$ /H ($\Delta E_{conf.min}$ / kJ mol^{-1})	$E_{conf.min}(\psi_{crys}$(PBE+TS)) /eV ($\Delta E_{conf.min}$ / kJ mol^{-1})	$E_{conf.min}(\psi_{crys}$(PBE+TS)) /eV ($\Delta E_{conf.min}$ / kJ mol^{-1})
A (5.3037)	-1059.20641739 (0.46)	-5611.372779731 (0.71)	-5611.805641 (0.84)
B (4.8358)	-1059.20659196 (0)	-5611.380134521 (0)	-5611.81439 (0)

S4.4 Small drug molecules

S4.4.1 Structure optimizations

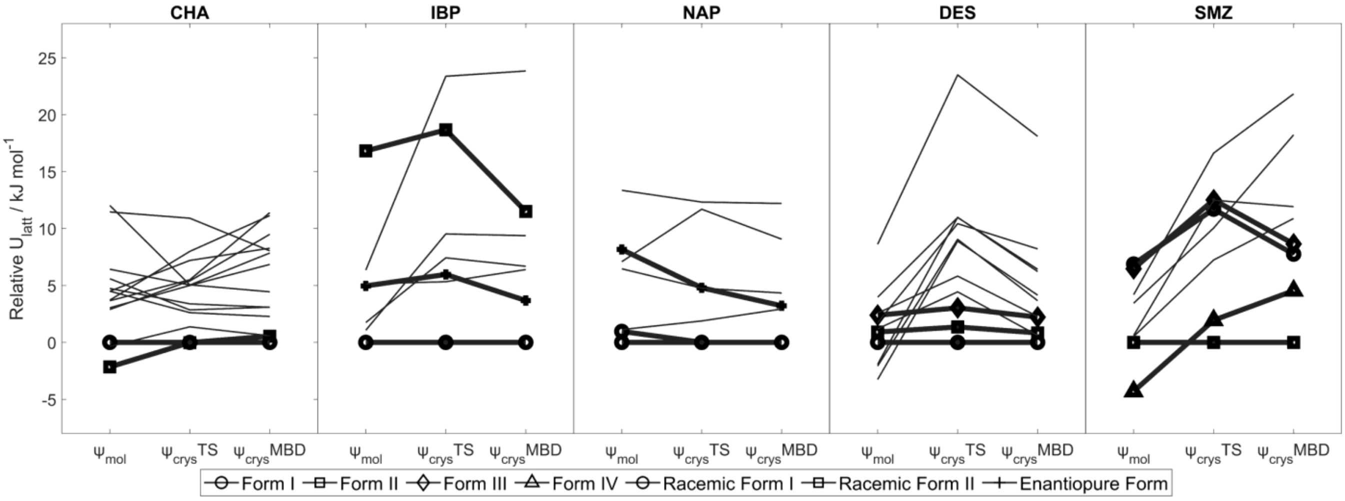

Figure S13. Relative lattice energies of carbamazepine and its analogues dihydrocarbamazepine, cyheptamide, cytenamide and oxcarbazepine. Structures in green have $R_2^2(8)$ hydrogen bonded amide dimers, whereas those in orange have $C_1^1(4)$ hydrogen bonded amide chains. Energies are calculated relative to that of the form believed to be most stable experimentally at low temperature (SI, Section S2.2.4). Structures with symbols are experimentally observed forms, with the form names in the legend. ψ_{mol} denotes lattice energy minimization corresponding to the original CSP; ψ_{crys}TS denotes lattice energy minimization with ψ_{crys}(PBE+TS); ψ_{crys}MBD denotes single point energy calculation of the ψ_{crys}(PBE+TS) structure with ψ_{crys}(PBE+MBD).

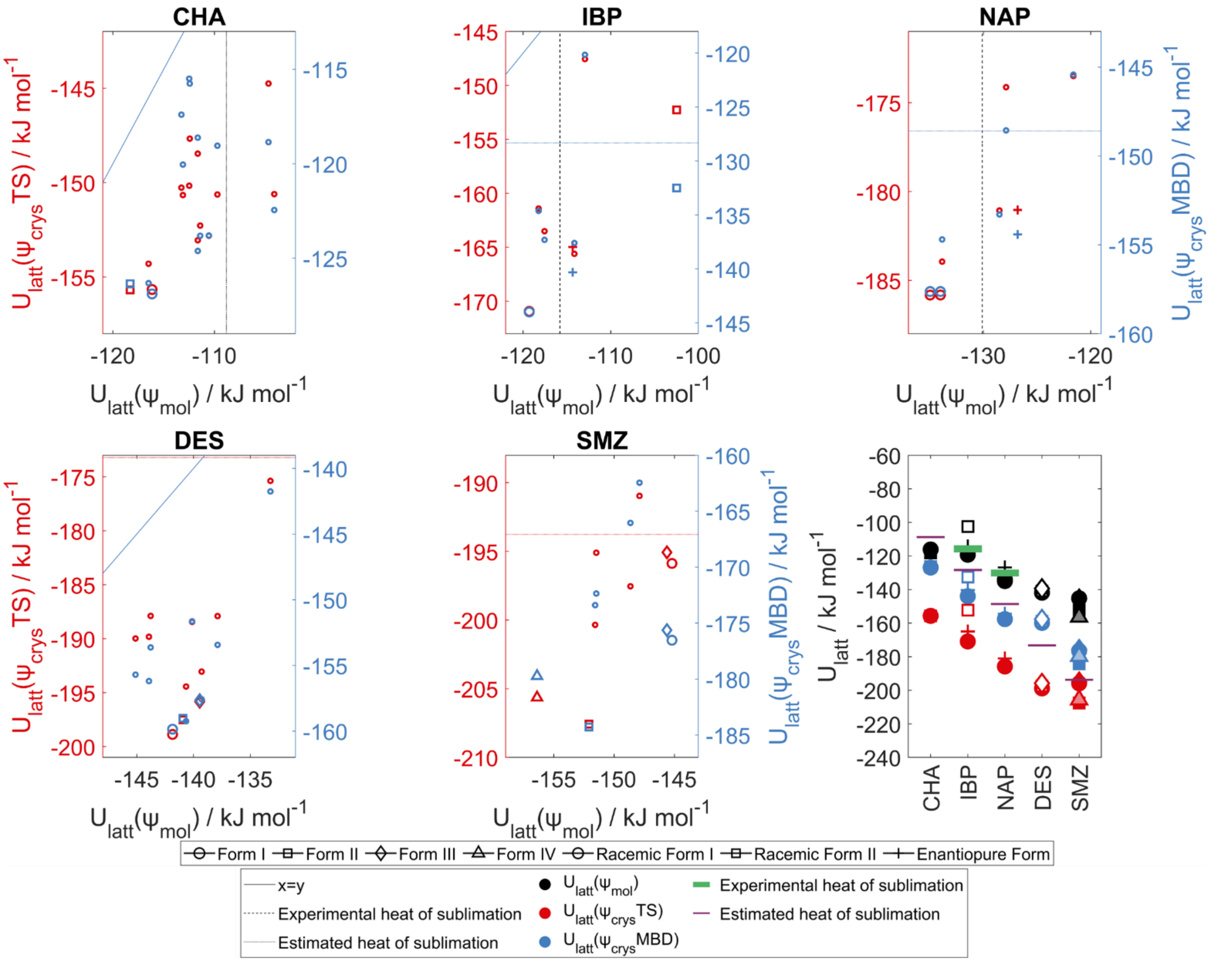

Figure S14. Comparison of lattice energies of CHA, IBP, NAP, DES and SMZ, with different computational models. Different energy scales are used for the different computational methods, colored black for ψ_{mol}, red for ψ_{crys}(PBE+TS) and blue for ψ_{crys}(PBE+MBD). The diagonal lines in the first five tiles are the x=y relationships, colored red for ψ_{mol}=ψ_{crys}(PBE+TS) and blue for ψ_{mol}=ψ_{crys}(PBE+MBD). The dashed lines are experimental heats of sublimation (where available) and the dotted lines are estimated heats of sublimation for the atomic types, placed when the scale allows, with the relevant axis colored black (vertical lines) for ψ_{mol}, red (horizontal lines) for ψ_{crys}(PBE+TS) and blue (horizontal lines) for ψ_{crys}(PBE+MBD). Similarly, when the scales allow the x=y lines to be drawn, these are included. In the bottom right corner, points are colored black for ψ_{mol}, red for ψ_{crys}(PBE+TS) and blue for ψ_{crys}(PBE+MBD), with filled symbols denoting simple solution crystallizations, shaded symbols denoting solution crystallizations with modified conditions or additives, and open symbols denoting crystallization not from solution. The green lines mark the experimental heats of sublimation (where available) and the purple lines mark the estimated heats of sublimation.

Table S27. Absolute and relative energies of various crystal structures of CHA, IBP, NAP, DES and SMZ, with different computational models.

**CHA**												
Label	Reason	ψ_{mol}			ψ_{crys}(PBE+TS)				ψ_{crys}(PBE+MBD) single point energy			ψ_{mol} vs. ψ_{crys}
		U_{latt} / kJ mol^{-1}	Relative U_{latt} / kJ mol^{-1}	$RMSD_{20}$ / Å	E / eV	U_{latt} / kJ mol^{-1}	Relative U_{latt} / kJ mol^{-1}	$RMSD_{20}$ / Å	E / eV	U_{latt} / kJ mol^{-1}	Relative U_{latt} / kJ mol^{-1}	$RMSD_{20}$ / Å
CHA01	Experimental (BZYACO03maj, form II); planar1=-0.48; lowest energy ψ_{mol} structure	-118.298	-2.159	0.23	-2997.619953	-155.68	-0.02104	0.26	-2997.727228	-126.35	0.543584	0.247
CHA02	Experimental (BZYACO, form I); planar1=4.91	-116.139	0	0.196	-2997.619735	-155.659	0	0.172	-2997.732862	-126.893	0	0.249
CHA03	planar1=0.69; I4, I10, T3, T5	-112.484	3.655		-2997.562785	-150.164	5.494898		-2997.614745	-115.496	11.39673	0.266
CHA04	planar1=-15.12; I1, I2, I4, I6	-112.43	3.709		-2997.536928	-147.669	7.989805		-2997.617438	-115.756	11.13694	0.654
CHA05	planar1=0.64; I8, T2, T5	-113.253	2.886		-2997.563843	-150.266	5.392889		-2997.634492	-117.402	9.491402	0.576
CHA06	planar1=-44.97; I1, T1	-111.642	4.497		-2997.592703	-153.051	2.608277		-2997.70927	-124.617	2.276297	0.294
CHA07	planar1=-43.94; I1, T5	-111.404	4.735		-2997.584662	-152.275	3.384126		-2997.700933	-123.812	3.080758	0.306
CHA08	planar1=-42.73; I6, I7	-110.551	5.588		-2997.590207	-152.81	2.849047		-2997.700803	-123.8	3.093301	0.299
CHA09	planar1=36.52; I3	-109.713	6.426		-2997.567583	-150.627	5.031983		-2997.651524	-119.045	7.84806	0.546
CHA10	planar1=34.68; I4	-111.646	4.493		-2997.545175	-148.465	7.19403		-2997.647111	-118.619	8.273833	0.345
CHA11	planar1=-6.58; T4	-113.105	3.034		-2997.567922	-150.66	4.999305		-2997.661887	-120.045	6.848141	0.31
CHA12	Lowest energy Region B conformation	-104.682	11.457		-2997.506696	-144.752	10.90675		-2997.649551	-118.855	8.038404	0.266
CHA13	Second lowest energy Region B conformation	-104.114	12.025		-2997.567371	-150.607	5.052464		-2997.686821	-122.451	4.442358	0.24
CHA14	planar1=-0.05; lowest energy ψ_{mol} structure in Sohncke space group	-116.489	-0.35		-2997.605583	-154.294	1.365493		-2997.726928	-126.321	0.572578	0.262

**IBP**												
Label	Reason	ψ_{mol}			ψ_{crys}(PBE+TS)				ψ_{crys}(PBE+MBD) single point energy			ψ_{mol} vs. ψ_{crys}
		U_{latt} / kJ mol^{-1}	Relative U_{latt} / kJ mol^{-1}	$RMSD_{20}$ / Å	E / eV	U_{latt} / kJ mol^{-1}	Relative U_{latt} / kJ mol^{-1}	$RMSD_{20}$ / Å	E / eV	U_{latt} / kJ mol^{-1}	Relative U_{latt} / kJ mol^{-1}	$RMSD_{20}$ / Å
IBP01	Experimental (IBPRAC01, Racemic form I), lowest energy ψ_{mol} structure (OppAt2; $R_2^2(8)$)	-119.299	0	0.143	-3221.011928	-170.923	0	0.146	-3221.11632	-143.99	0	0.239
IBP02	Experimental (JEKNOC12, Enantiopure form), lowest energy chiral ψ_{mol} structure (OppAc2; $R_2^2(8)$)	-114.341	4.958	0.156	-3220.950094	-164.957	5.966161	0.145	-3221.078218	-140.313	3.676372	0.219
IBP03	Experimental (IBPRAC04, Racemic form II) (OppAc1, OppAt2; $R_2^2(8)$)	-102.487	16.812	0.729↑	-3220.818489	-152.259	18.6643	0.621↑	-3220.997149	-132.491	11.49848	0.377
IBP04	Lowest energy Same conformation (SameAt2; $R_2^2(8)$)	-118.234	1.065		-3220.913243	-161.402	9.521802		-3221.019186	-134.618	9.372168	0.208

IBP05	Lowest energy different i-Pr conformation (OppAt3; $R_2^2(8)$)	-117.558	1.741		-3220.934982	-163.499	7.424304		-3221.047033	-137.304	6.685295	0.318
IBP06	Packing (SameAt3; $C_1^1(4)$)	-114.155	5.144		-3220.956737	-165.598	5.325216		-3221.050052	-137.596	6.394049	0.174
IBP07	Packing (OppAc1; $C_1^1(2)$)	-112.951	6.348		-3220.769697	-147.551	23.37212		-3220.869296	-120.155	23.83463	0.302
**NAP**												
Label	Reason	ψ_{mol}			ψ_{crys}(PBE+TS)				ψ_{crys}(PBE+MBD) single point energy			ψ_{mol} vs. ψ_{crys}
		U_{latt} / kJ mol^{-1}	Relative U_{latt} / kJ mol^{-1}	RMSD$_{20}$ / Å	E / eV	U_{latt} / kJ mol^{-1}	Relative U_{latt} / kJ mol^{-1}	RMSD$_{20}$ / Å	E / eV	U_{latt} / kJ mol^{-1}	Relative U_{latt} / kJ mol^{-1}	RMSD$_{20}$ / Å
NAP01	Experimental (PAPTUX, Racemic form), lowest energy ψ_{mol} structure	-134.97	0	1.051↑	-3752.288622	-185.825	0	0.198	-3752.428499	-157.626	0	1.04↑
NAP02	Experimental (COYRUD14, Enantiopure form)	-126.812	8.158	0.5	-3752.238951	-181.033	4.792591	0.238	-3752.395186	-154.412	3.214271	0.336
NAP03	Saddle point of experimental structure	-134.007	0.963	1.014↑	-3752.288588	-185.822	0.003315	0.202	-3752.428493	-157.626	0.000555	1.029↑
NAP04	Alternative conformation, same packing as NAP01 (PAPTUX)	-128.506	6.464	0.605↑	-3752.23931	-181.068	4.757953	0.601↑	-3752.383544	-153.289	4.337501	0.295
NAP05	Alternative conformation, same packing as NAP02 (COYRUD14)	-121.611	13.359	0.869↑	-3752.160915	-173.503	12.32214	0.91↑	-3752.302058	-145.426	12.19986	0.279
NAP06	Lowest energy chiral ψ_{mol} structure	-127.886	7.084		-3752.167418	-174.131	11.69468		-3752.334559	-148.562	9.063989	0.171
NAP07	Dense, low energy structure	-133.848	1.122		-3752.269121	-183.944	1.881628		-3752.4	-154.696	2.929731	0.194
**DES**												
Label	Reason	ψ_{mol}			ψ_{crys}(PBE+TS)				ψ_{crys}(PBE+MBD) single point energy			ψ_{mol} vs. ψ_{crys}
		U_{latt} / kJ mol^{-1}	Relative U_{latt} / kJ mol^{-1}	RMSD$_{20}$ / Å	E / eV	U_{latt} / kJ mol^{-1}	Relative U_{latt} / kJ mol^{-1}	RMSD$_{20}$ / Å	E / eV	U_{latt} / kJ mol^{-1}	Relative U_{latt} / kJ mol^{-1}	RMSD$_{20}$ / Å
DES01	Experimental (GEHXEX01, form I)	-141.849	0	0.163	-4278.565902	-198.869	0	0.114	-4278.759342	-159.851	0	0.185
DES02	Experimental (GEHXEX04, form II)	-140.94	0.909	0.147	-4278.551891	-197.517	1.351926	0.234	-4278.750769	-159.024	0.827111	0.197
DES03	Experimental (GEHXEX02, form III)	-139.463	2.386	0.616	-4278.534498	-195.839	3.030094	0.669	-4278.736415	-157.639	2.212157	0.22
DES04	Lowest energy ψ_{mol} structure $C_1^1(2)$ hydrogen bonding	-145.11	-3.261		-4278.47374	-189.977	8.892441		-4278.716347	-155.702	4.148434	0.271
DES05	Lowest energy chiral ψ_{mol} structure $C_1^1(2)$ hydrogen bonding	-143.776	-1.927		-4278.452083	-187.887	10.98207		-4278.694858	-153.629	6.221868	0.227
DES06	Lowest energy $C_1^1(8)$ hydrogen bonding	-140.657	1.192		-4278.519907	-194.431	4.437973		-4278.753049	-159.244	0.607169	0.252
DES07	Lowest energy $R_2^2(4)$ hydrogen bonding	-133.23	8.619		-4278.322425	-175.377	23.49239		-4278.571803	-141.756	18.09505	0.289
DES08	Lowest energy $R_2^2(16)$ hydrogen bonding	-139.288	2.561		-4278.505587	-193.05	5.819582		-4278.735742	-157.574	2.277021	0.238
DES09	Lowest energy ASA conformation	-137.883	3.966		-4278.452255	-187.904	10.96552		-4278.692802	-153.431	6.420221	0.224
DES10	Lowest energy SAE conformation	-140.124	1.725		-4278.45785	-188.444	10.42565		-4278.674265	-151.642	8.208824	0.241
DES11	Lowest energy SSE conformation	-143.926	-2.077		-4278.47211	-189.82	9.049712		-4278.721383	-156.188	3.662502	0.276

SMZ												
Label	Reason	ψ_{mol}			ψ_{crys}(PBE+TS)				ψ_{crys}(PBE+MBD) single point energy			ψ_{mol} vs. ψ_{crys}
		U_{latt} / kJ mol^{-1}	Relative U_{latt} / kJ mol^{-1}	$RMSD_{20}$ / Å	E / eV	U_{latt} / kJ mol^{-1}	Relative U_{latt} / kJ mol^{-1}	$RMSD_{20}$ / Å	E / eV	U_{latt} / kJ mol^{-1}	Relative U_{latt} / kJ mol^{-1}	$RMSD_{20}$ / Å
SMZ01	Experimental (SLFNMA02, form I)	-145.204	6.88	0.258	-4216.777016	-195.877	11.70513	0.096	-4216.984822	-176.529	7.748569	0.227
SMZ02	Experimental (SLFNMA01, form II)	-152.084	0	0.126	-4216.898329	-207.582	0	0.215	-4217.065129	-184.278	0	0.277
SMZ03	Experimental (SLFNMA03, form III)	-145.616	6.468	0.268	-4216.76861	-195.066	12.51617	0.098	-4216.975527	-175.632	8.645464	0.234
SMZ04	Experimental (SLFNMA06, form IV), lowest energy ψ_{mol} structure	-156.387	-4.303	0.154	-4216.878197	-205.64	1.942458	0.167	-4217.018056	-179.736	4.541993	0.263
SMZ05	Lowest energy chiral ψ_{mol} structure	-147.896	4.188		-4216.726047	-190.96	16.62299		-4216.838944	-162.454	21.82397	0.162
SMZ06	Distinct packing (different layer)	-151.478	0.606		-4216.768928	-195.097	12.48547		-4216.941583	-172.357	11.92062	0.29
SMZ07	Distinct packing (different layer)	-151.57	0.514		-4216.823373	-200.35	7.232232		-4216.95231	-173.392	10.88558	0.189
SMZ08	Lowest energy structure with alternative conformation	-148.647	3.437		-4216.794253	-197.541	10.04198		-4216.876178	-166.046	18.23133	0.278

↑ denotes structures which could only be matched if the tolerances in Mercury were increased.

S4.4.2 Molecule optimizations

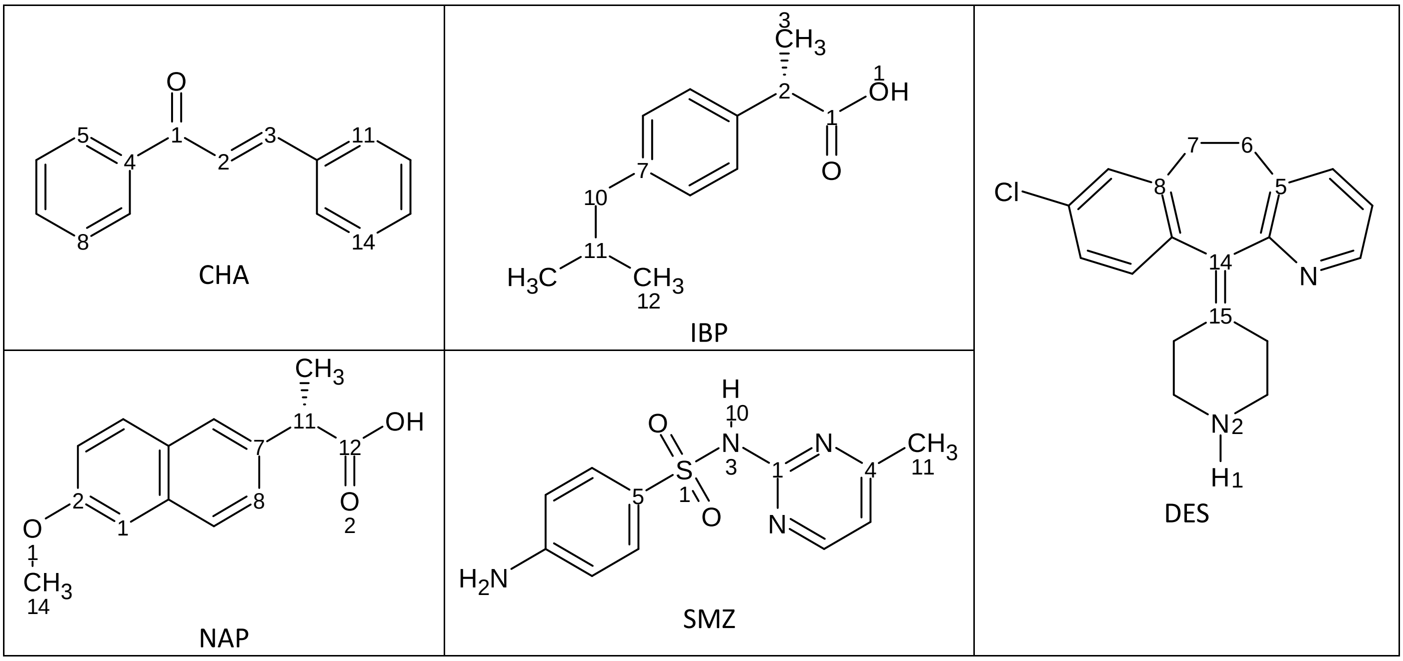

Figure S15. Molecular diagrams of CHA, IBP, NAP, DES and SMZ, showing the atom numbers used to define degrees of freedom of interest in Table S28.

Table S28. Gas phase molecular conformations of small drug molecules. CHA: ϑ_1 is C4-C1(=O)-C2=C3; planar1 is C8···C5···C11···C14. IBP: ϑ_1 is C1-C2···C10-C11, ϑ_2 is O1-C1-C2-C3, ϑ_3 is C12-C11-C10-C7. NAP: ϑ_1 is C14-O1-C2-C1, ϑ_2 is C12-C11-C7-C8, ϑ_3 is O2-C12-C11-C7. DES: ϑ_1 is C5-C6-C7-C8, ϑ_2 is N2···C15=C14···C7, ϑ_3 is H1-N2···C15=C14. SMZ: ϑ_1 is C11-C4···N3-H10, ϑ_2 is C1-N3-S1-C5.

			**CHA**		
Conformation (dipole moment)	ψ_{mol} MP2/6-31G(d,p) optimized		ψ_{crys}(PBE+TS) optimized		
	θ_1 angle / ° planar1 angle / °	$E_{conf.min}(\psi_{mol})$ /H ($\Delta E_{conf.min}$ / kJ mol^{-1})	θ_1 angle / ° planar1 angle / °	$E_{conf.min}(\psi_{crys}$(PBE+TS)) /eV ($\Delta E_{conf.min}$ / kJ mol^{-1})	$E_{conf.min}(\psi_{crys}$(PBE+TS)) /eV ($\Delta E_{conf.min}$ / kJ mol^{-1})
planar (3.4262)	169.681 -12.948	-652.03045337978 (0)	175.82 -23.61	-2996.006468 (0)	-2996.417712 (0)
twisted (3.473)	-169.899 47.492	-652.03038355150 (0.18)	-174.86 24.292	-2996.003493 (0.29)	-2996.414047 (0.35)
confB (4.3441)	-27.634 145.40	-652.02915813172 (3.40)	-23.84 123.24	-2995.979414 (2.61)	-2996.380886 (3.55)
			**IBP**		
Conformation (dipole moment)	ψ_{mol} PBE0/6-31G(d,p) optimized		ψ_{crys}(PBE+TS) optimized		
	Angles / °	$E_{conf.min}(\psi_{mol})$ /H ($\Delta E_{conf.min}$ / kJ mol^{-1})	Angles / °	$E_{conf.min}(\psi_{crys}$(PBE+TS)) /eV ($\Delta E_{conf.min}$ / kJ mol^{-1})	$E_{conf.min}(\psi_{crys}$(PBE+TS)) /eV ($\Delta E_{conf.min}$ / kJ mol^{-1})
OppAc1 (1.8364)	θ_1=-167.866 θ_2=-46.137 θ_3=63.314	-655.965359108 (5.22)	θ_1=-170.97 θ_2=-43.90 θ_3=61.94	-3219.196860139 (4.21)	-3219.579249688 (4.31)
OppAc2 (1.9473)	θ_1=-140.804 θ_2=-46.411 θ_3=173.024	-655.965374638 (5.18)	θ_1=-147.26 θ_2=-44.17 θ_3=174.26	-3219.19778444 (4.12)	-3219.580000785 (4.24)
OppAc3 (1.8898)	θ_1=-153.054 θ_2=-45.507 θ_3=-62.645	-655.964167790 (8.35)	θ_1=-160.78 θ_2=-44.21 θ_3=-62.91	-3219.175927835 (6.23)	-3219.55066339 (7.07)
OppAt1 (1.5729)	θ_1=-156.495 θ_2=148.728 θ_3=63.205	-655.967294561 (0.14)	θ_1=-158.69 θ_2=151.61 θ_3=61.90	-3219.23767778 (0.27)	-3219.621106836 (0.28)
OppAt2 (1.4065)	θ_1=-129.295 θ_2=148.559 θ_3=173.208	-655.967322804 (0.06)	θ_1=-132.74 θ_2=151.58 θ_3=174.54	-3219.238627929 (0.18)	-3219.622119363 (0.18)
OppAt3 (1.5131)	θ_1=-144.916 θ_2=148.749 θ_3=-62.974	-655.966102682 (3.27)	θ_1=-147.54 θ_2=151.39 θ_3=-63.10	-3219.217342053 (2.23)	-3219.594862545 (2.81)
SameAc1 (1.7802)	θ_1=15.197 θ_2=-45.033	-655.965402827 (5.10)	θ_1=5.76 θ_2=-44.68	-3219.198124887 (4.09)	-3219.580552103 (4.19)

	θ_3=63.034		θ_3=61.90		
SameAc2 (1.6874)	θ_1=40.493 θ_2=-45.600 θ_3=173.264	-655.965409224 (5.09)	θ_1=31.39 θ_2=-44.23 θ_3=174.47	-3219.198497808 (4.05)	-3219.580638059 (4.18)
SameAc3 (1.7628)	θ_1=28.649 θ_2=-44.964 θ_3=-62.622	-655.964204580 (8.25)	θ_1=17.35 θ_2=-44.68 θ_3=63.46	-3219.175820402 (6.24)	-3219.550068534 (7.13)
SameAt1 (1.442)	θ_1=19.617 θ_2=148.719 θ_3=63.136	-655.967347173 (0)	θ_1=19.81 θ_2=151.36 θ_3=61.49	-3219.240462473 (0)	-3219.62396495 (0)
SameAt2 (1.6099)	θ_1=52.558 θ_2=148.758 θ_3=173.129	-655.967315280 (0.08)	θ_1=44.65 θ_2=151.39 θ_3=174.24	-3219.23780092 (0.26)	-3219.621292627 (0.26)
SameAt3 (1.5462)	θ_1=33.558 θ_2=149.191 θ_3=-63.280	-655.966104448 (3.26)	θ_1=31.77 θ_2=150.88 θ_3=-63.62	-3219.220722882 (1.9)	-3219.598155121 (2.49)
			**NAP**		
Conformation (dipole moment)	ψ_{mol} PBE0/6-31G(d,p) optimized		ψ_{crys}(PBE+TS) optimized		
	Angles / °	$E_{conf.min}(\psi_{mol})$ /H ($\Delta E_{conf.min}$ / kJ mol^{-1})	Angles / °	$E_{conf.min}(\psi_{crys}$(PBE+TS)) /eV ($\Delta E_{conf.min}$ / kJ mol^{-1})	$E_{conf.min}(\psi_{crys}$(PBE+TS)) /eV ($\Delta E_{conf.min}$ / kJ mol^{-1})
AC (1.3375)	θ_1=0.037 θ_2=-56.098 θ_3=90.122	-766.763442362 (0)	θ_1=0.29 θ_2=-59.53 θ_3=95.00	-3750.362710379 (0)	-3750.794846545 (0)
AD (2.5343)	θ_1=0.031 θ_2=129.907 θ_3=89.679	-766.763058699 (1.01)	θ_1=0.02 θ_2=124.23 θ_3=94.99	-3750.353031772 (0.93)	-3750.784705709 (0.98)
AE (2.7046)	θ_1=0.141 θ_2=-64.540 θ_3=-99.301	-766.761461216 (5.2)	θ_1=-0.22 θ_2=-70.44 θ_3=-97.75	-3750.320785463 (4.05)	-3750.752203083 (4.11)
AF (1.9217)	θ_1=-0.054 θ_2=116.879 θ_3=-98.673	-766.760988688 (6.44)	θ_1=0.05 θ_2=107.63 θ_3=-96.17	-3750.308996643 (5.18)	-3750.739816772 (5.31)
BC (3.0356)	θ_1=-179.994 θ_2=-56.349 θ_3=90.379	-766.761019521 (6.36)	θ_1=-179.92 θ_2=-59.61 θ_3=94.93	-3750.302880181 (5.77)	-3750.732721754 (5.99)
BD (1.2685)	θ_1=-179.911 θ_2=130.362 θ_3=89.420	-766.760680717 (7.25)	θ_1=-179.72 θ_2=125.53 θ_3=95.15	-3750.292980954 (6.73)	-3750.723777412 (6.86)
BE (1.8626)	θ_1=179.834 θ_2=-65.206 θ_3=-99.482	-766.759119293 (11.35)	θ_1=-179.67 θ_2=-71.13 θ_3=-98.37	-3750.260818754 (9.83)	-3750.689386517 (10.18)
BF (3.1393)	θ_1=-179.792 θ_2=117.574 θ_3=-98.577	-766.75850835 (12.95)	θ_1=179.92 θ_2=94.79 θ_3=-97.27	-3750.249907817 (10.88)	-3750.681074802 (10.98)
			**DES**		
Conformation (dipole moment)	ψ_{mol} PBE0/6-31G(d,p) optimized		ψ_{crys}(PBE+TS) optimized		
	Angles / °	$E_{conf.min}(\psi_{mol})$ /H ($\Delta E_{conf.min}$ / kJ mol^{-1})	Angles / °	$E_{conf.min}(\psi_{crys}$(PBE+TS)) /eV ($\Delta E_{conf.min}$ / kJ mol^{-1})	$E_{conf.min}(\psi_{crys}$(PBE+TS)) /eV ($\Delta E_{conf.min}$ / kJ mol^{-1})
AAA (3.736)	θ_1=-58.07 θ_2=-160.5 θ_3=-177.95	-1304.03475077 (3.15)	θ_1=-57.20 θ_2=-161.12 θ_3=-178.24	-4276.479373 (2.45)	-4277.081674 (2.02)
AAE (2.4481)	θ_1=-57.88 θ_2=-160.51 θ_3=1.49	-1304.03459362 (3.56)	θ_1=-57.03 θ_2=-161.13 θ_3=1.13	-4276.471695 (3.19)	-4277.079703 (2.21)
ASA (2.3572)	θ_1=-58.22 θ_2=23.18 θ_3=178.71	-1304.03478198 (3.06)	θ_1=-57.23 θ_2=21.34 θ_3=178.72	-4276.480652 (2.33)	-4277.074867 (2.68)
ASE (3.7299)	θ_1=-58.36 θ_2=23.10 θ_3=-1.73	-1304.03474640 (3.16)	θ_1=-57.13 θ_2=21.38 θ_3=-1.50	-4276.482363 (2.17)	-4277.084032 (1.79)
SAA (3.2849)	θ_1=60.32 θ_2=-163.88 θ_3=178.92	-1304.03594875 (0)	θ_1=59.67 θ_2=-163.78 θ_3=178.84	-4276.504804 (0)	-4277.102633 (0)
SAE	θ_1=59.82	-1304.03555322	θ_1=58.81	-4276.491017	-4277.094374

(2.3397)	θ_2=-163.41<br>θ_3=-1.87	(1.04)	θ_2=-163.38<br>θ_3=-2.00	(1.33)	(0.80)
SSA<br>(2.3021)	θ_1=60.22<br>θ_2=12.98<br>θ_3=-178.47	-1304.03557164<br>(0.99)	θ_1=58.63<br>θ_2=13.60<br>θ_3=-178.41	-4276.495729<br>(0.88)	-4277.087884<br>(1.42)
SSE<br>(3.2271)	θ_1=60.29<br>θ_2=12.51<br>θ_3=2.46	-1304.03546490<br>(1.27)	θ_1=58.51<br>θ_2=13.23<br>θ_3=2.29	-4276.495543<br>(0-89)	-4277.095071<br>(0.73)
**SMZ**					
Conformation<br>(dipole moment)	ψ_{mol} PBE0/6-31G(d,p) optimized		ψ_{crys}(PBE+TS) optimized		
	Angles / °	$E_{conf.min}(\psi_{mol})$ /H<br>($\Delta E_{conf.min}$ / kJ mol^{-1})	Angles / °	$E_{conf.min}(\psi_{crys}$(PBE+TS)) /eV<br>($\Delta E_{conf.min}$ / kJ mol^{-1})	$E_{conf.min}(\psi_{crys}$(PBE+TS)) /eV<br>($\Delta E_{conf.min}$ / kJ mol^{-1})
opp<br>(6.6733)	θ_1=174.76<br>θ_2=-77.20	-1192.97281553<br>(0)	θ_1=-173.03<br>θ_2=-61.37	-4214.746925<br>(0)	-4215.155258<br>(0)
same<br>(7.325)	θ_1=4.14<br>θ_2=78.22	-1192.97242975<br>(1.01)	θ_1=1.94<br>θ_2=-67.64	-4214.723846<br>(2.23)	-4215.135765<br>(1.88)
ext<br>(5.8541)	θ_1=162.26<br>θ_2=171.64	-1192.96580900<br>(18.40)	θ_1=165.08<br>θ_2=171.43	-4214.524034<br>(21.51)	-4214.939137<br>(20.85)

* * Singlemol_cellmaker has changed the chirality of some molecules. The signs of the angles have been swapped in this table to allow easier comparisons. They will not match the .mol files included as SI.

Table S28 shows that the planar and twisted conformations of CHA are not distinct minima with the PBE method in CASTEP, optimizing to the same conformation, in agreement with the observation made in the packing preferences paper[24] that these conformations of the isolated molecule are not distinct minima with the PBE functional but are with the MP2 calculation.

S5 Comparison of results across the ψ_{mol} and ψ_{crys} datasets

S5.1 Comparison of isolated molecule energy differences with different methods

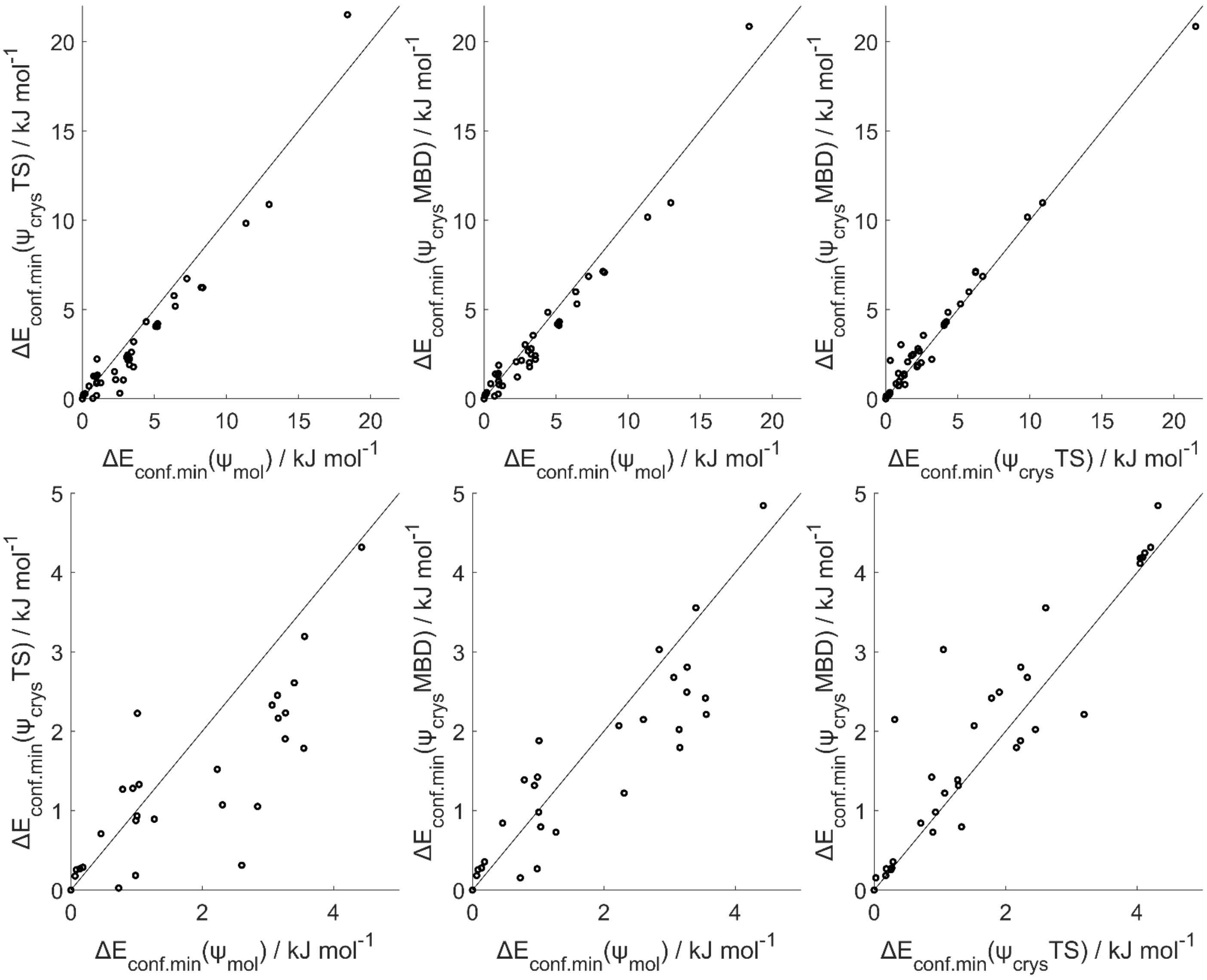

Figure S16. Plots comparing the energy of gas phase optimized local minimum conformations with the gas phase optimized global minimum of each molecule with different computational methods. Tow row – full energy range. Bottom row – only lowest 5 kJ mol^{-1} plotted. The global minima are also included with a relative energy of 0, hence if the same molecular conformation is determined to be the global minimum with both methods, this will appear at 0,0. The x=y line is added to aid comparison of the values. The conformations are identical for the energy evaluation with the two ψ_{crys} energy models (optimized with PBE+TS and single point energy evaluated with PBE+MBD).

S5.2 Comparison of absolute lattice energies with different methods

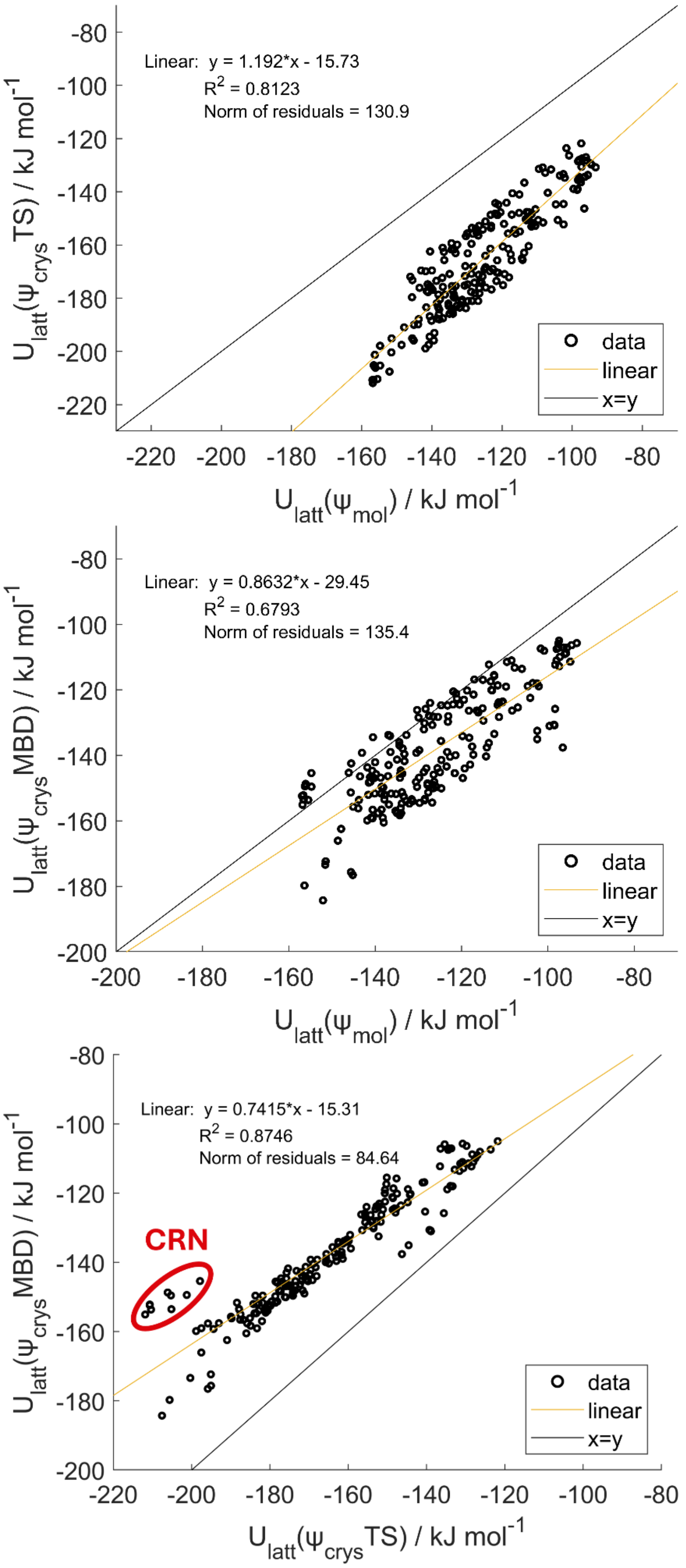

Figure S17. Plots comparing the lattice energy with different computational methods. The data is fitted to a linear equation in MATLAB (version R2024a). On the lowest chart, the highlighted cluster of points is all the crystal structures of CRN. The bottom chart compares lattice energies which differ only in the dispersion correction, demonstrating that even this difference is not systematic.

S5.3 Energy comparison between chiral and racemic crystal structures

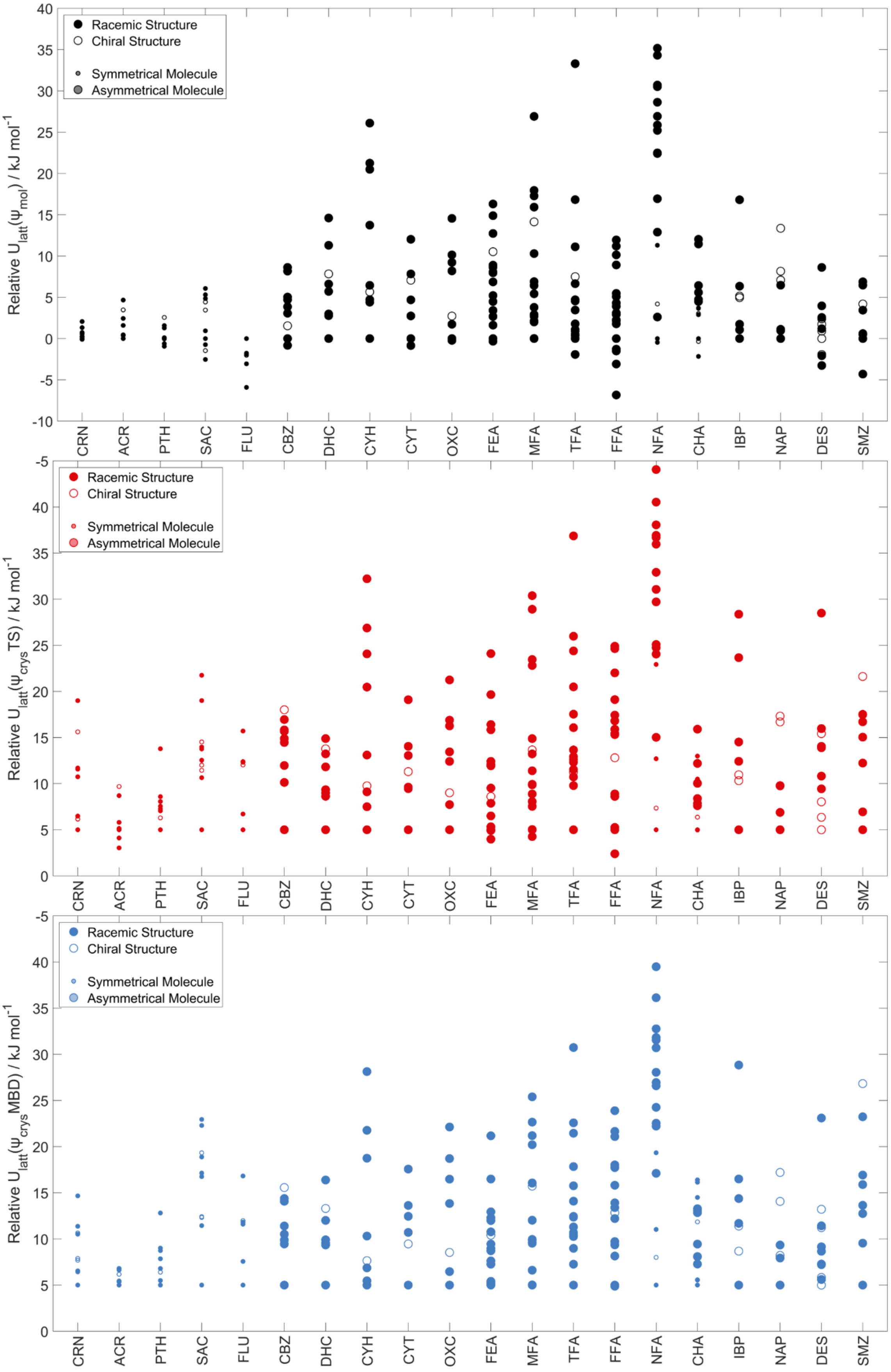

Figure S18. Relative energies of the crystal structure investigated in this work, categorized by whether the crystal structure is chiral (strictly, whether it is in a Sohncke space group, open symbols) or racemic (filled symbols), colored black for ψ_{mol}, red for ψ_{mol}(PBE+TS) and blue for ψ_{mol}(PBE+MBD). Smaller symbols denote molecules which are planar or otherwise inherently symmetrical and so cannot adopt a chiral conformation and so the crystal structures in Sohncke space groups are not chiral.

S6 Use of the database structures for evaluating the MACE-OFF23 and MACE-MP-0+D3(BJ) force fields

S6.1 Comparison of crystal structure optimizations

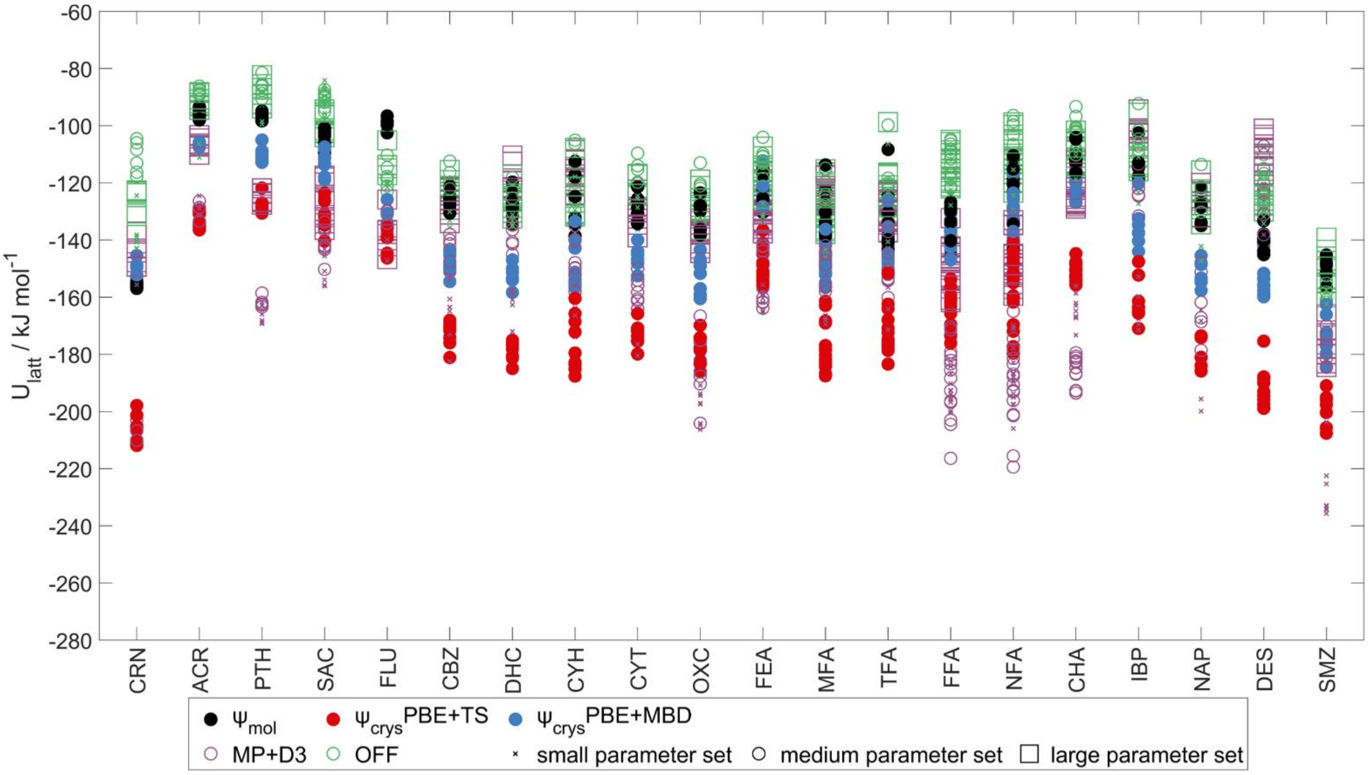

Figure S19. Comparison of lattice energies calculated with the two machine-learned force fields (MACE-MP-0+D3(BJ)=purple; MACE-OFF23=green) with the ψ_{mol} (black), ψ_{crys}(PBE+TS) (red) and ψ_{crys}(PBE+MBD) (blue) energies. All crystal structures for an individual molecule are displayed at the same x-coordinate. For more detail, see Figure S20.

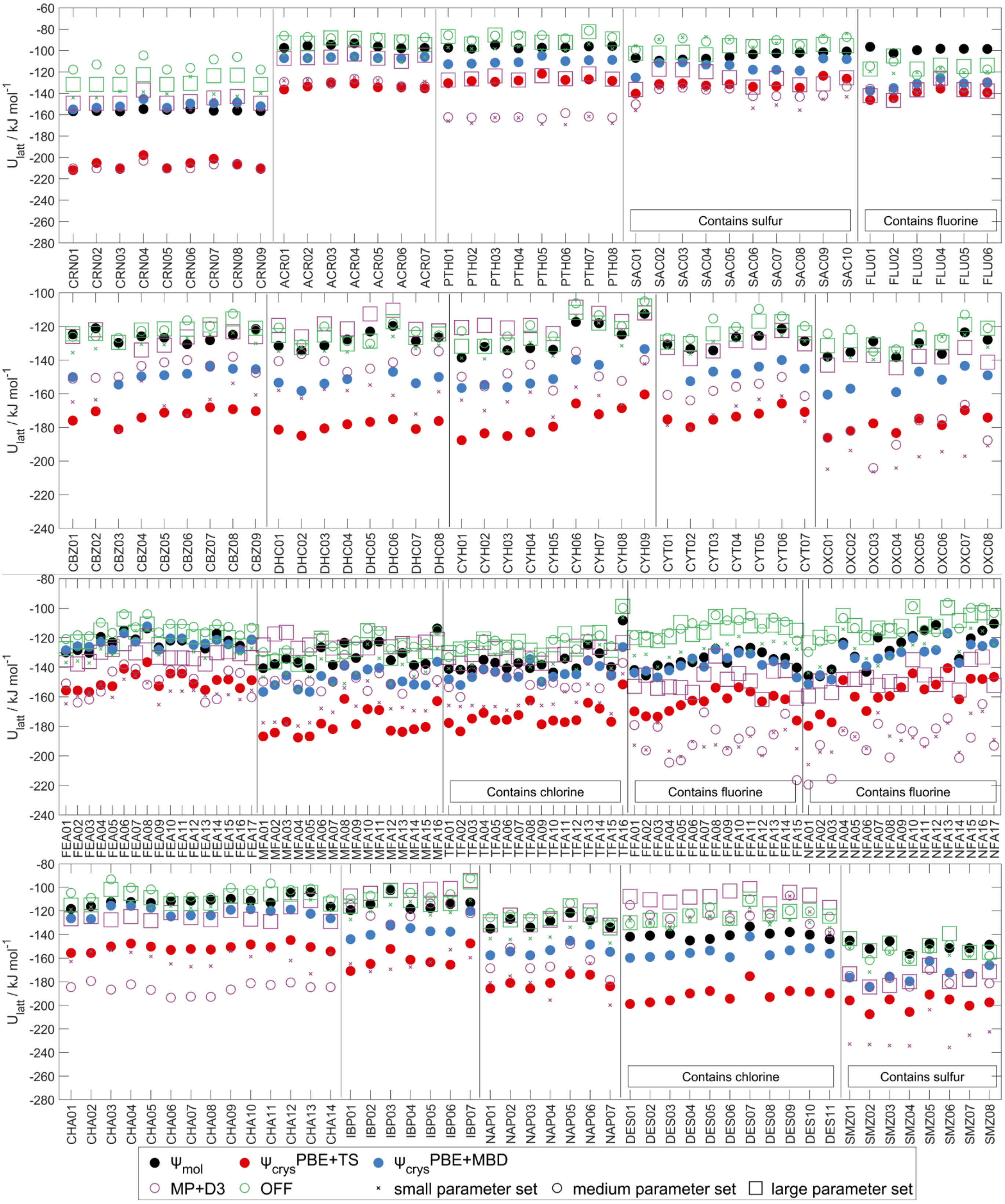

Figure S20. Comparison of lattice energies calculated with the two machine-learned force fields (MACE-MP-0+D3(BJ)=purple; MACE-OFF23=green) with the ψ_{mol} (black), ψ_{crys}(PBE+TS) (red) and ψ_{crys}(PBE+MBD) (blue) energies. The nine lattice energies calculated for each individual crystal structure are plotted at the same x-coordinate. Note that the vertical scales differ on the four panels; all lattice energies are compared on the same scale in Figure S19.

Figure S20 shows that different methods often have different energy rankings of the observed and hypothetical polymorphs when the arrangements of the symbols differ.

S6.1.1 Role of dispersion correction

Initially, the crystal structure optimizations were carried out with the two force fields without modification. The results for MACE-MP-0, as exemplified on the left of Figure S21 with the small parameter set, generally seem unrealistically small, with two structures, DES09 with the small parameter set and DES06 with the large parameter set, having positive lattice energies. Therefore, the D3(BJ) dispersion correction was added to MACE-MP-0. The righthand side of Figure S21 shows the lattice energies calculated for the MACE-MP-0+D3(BJ) potential after optimization, and an estimate of the dispersion (which is greater for the larger molecules) made by subtracting the single point energies calculated with MACE-MP-0 (i.e. without the dispersion correction).

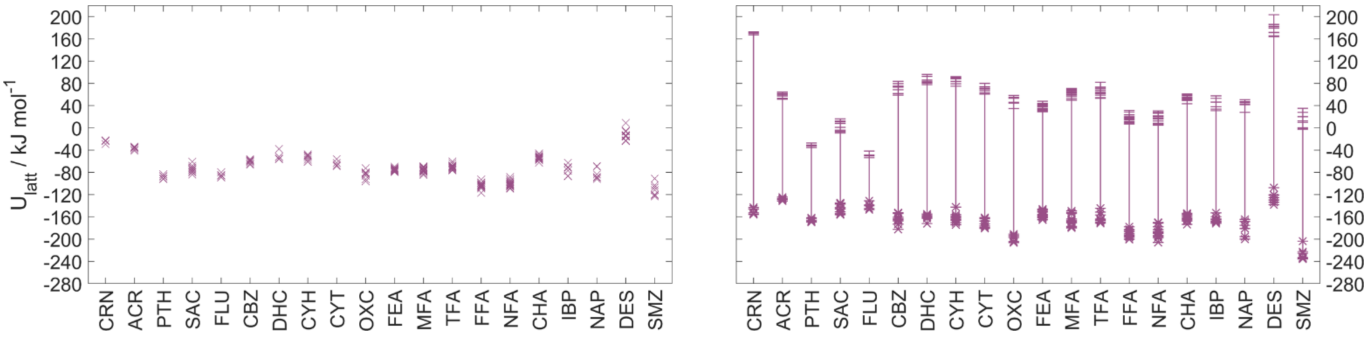

Figure S21. Lattice energies for crystal structures optimized with the MACE-MP-0 foundation model and small parameter set. Left: when no dispersion is used. Right: when the optimization is carried out including D3(BJ) dispersion, with the vertical lines showing the magnitude of the dispersion contribution, i.e. the x symbol is the optimized MACE-MP-0+D3(BJ) lattice energy and the – symbol the single point MACE-MP-0 energy.

S6.1.2 Reproduction of coronene experimental crystal structures

Table S29. Comparison of CRN experimental structures with structures minimized with the MACE force fields. The name of the force field is abbreviated to "mp" for MACE-MP-0+D3(BJ) or to "off" for MACE-OFF23. Cells are colored purple for MACE-MP-0+D3(BJ), green for MACE-OFF23, and yellow where the structural match is not correct. The heavily outlined regions are where matches should occur – only optimisations starting from CRN01 should match CORONE04 and only optimisations starting from CRN02 should match CORONE03.

Starting Structure	Force field	Parameter set	CSD structure: CORONE04 β # molecules matched	CORONE04 β $RMSD_{20}$ /Å	CORONE03 γ # molecules matched	CORONE03 γ $RMSD_{20}$ /Å
CRN01	mp	small	20 out of 20	0.74947	3 out of 20	
	mp	medium	20 out of 20	0.806854	3 out of 20	
	mp	large	11 out of 20		3 out of 20	
	off	small	11 out of 20		20 out of 20	0.310934
	off	medium	11 out of 20		3 out of 20	
	off	large	11 out of 20		3 out of 20	
CRN02	mp	small	20 out of 20	0.749445	3 out of 20	
	mp	medium	20 out of 20	0.806874	3 out of 20	
	mp	large	11 out of 20		3 out of 20	
	off	small	11 out of 20		20 out of 20	0.310936
	off	medium	20 out of 20	0.813933	20 out of 20	0.717825
	off	large	11 out of 20		3 out of 20	
CRN03	mp	small	20 out of 20	0.749522		
CRN05	mp	small	20 out of 20	0.749457		
CRN09	mp	small	20 out of 20	0.749467		

Table S29 shows the structure matches for all experimental polymorphs of coronene with the minimized structures with both force fields and all three parameter sets. Both foundation models with the large parameter set do not retain enough character of either of the experimental crystal structures for them to be deemed a successful match. Furthermore, the only convincing match ($RSMD_{20}$=0.311 Å) was between CRN02 optimized with MACE-OFF23 with the small parameter set and CRN γ (CORONE03); this force field also optimized CRN01 to the same structure. MACE-

MP-0+D3(BJ) with the small and medium parameter sets optimized both CRN01 and CRN02 to the same structure which was a poor match to CRN β (CORONE04) and not a match to CRN γ (CORONE03). MACE-OFF23 with the medium parameter set changed CRN02 to a structure which could be matched to both experimental crystal polymorphs, yet optimized CRN01 to a structure that matched neither experimental polymorph. CRN03, CRN05 and CRN09 also optimized to a structure which resembled CRN β with MACE-MP-0+D3(BJ) and the small parameter set.

S6.1.3 Comparison of experimental crystal structures of CRN β and CRN γ

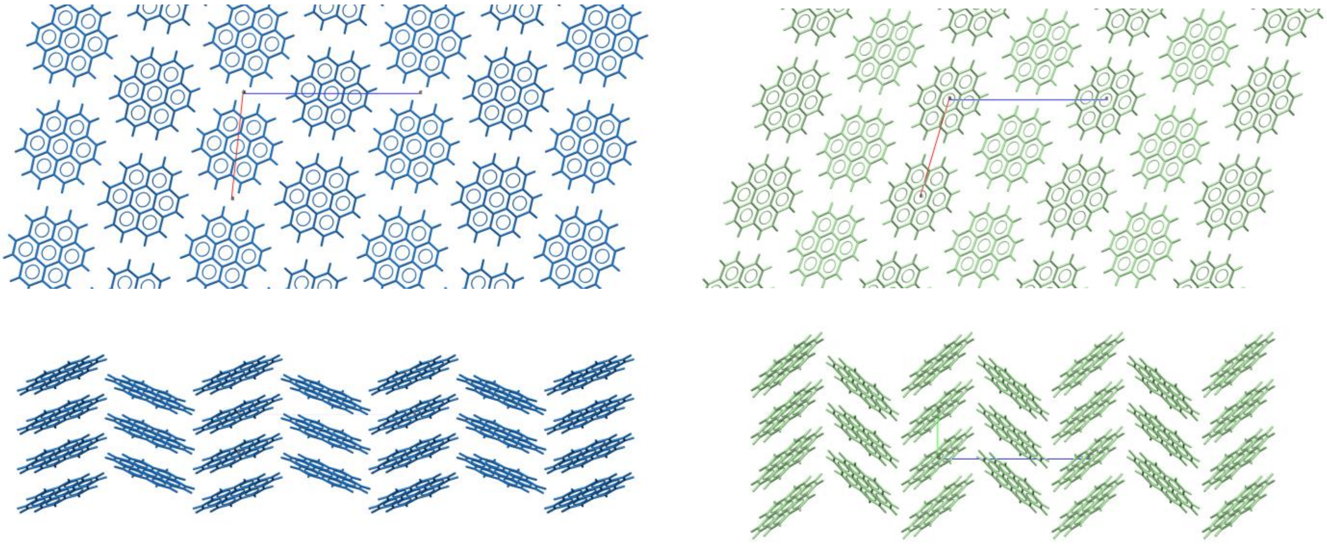

Figure S22. Comparison of crystal structures of CRN β (CORONE04, left, blue) and CRN γ (CORONE03, right, green). Top row: the view down the stacks of molecules. Bottom row: the view perpendicular to the stacks of molecules. Both phases contain two molecules per unit cell, but with a major difference in the angle between the molecular planes. However, many potential energy surfaces do not have both minima and are sufficiently flat that the molecules can slip between these structures (Table S29).

S6.2 Comparison of isolated molecule conformations

The lowest energy conformations for each molecule usually remained the same as for the ψ_{mol} and ψ_{crys}(PBE+TS) optimizations.

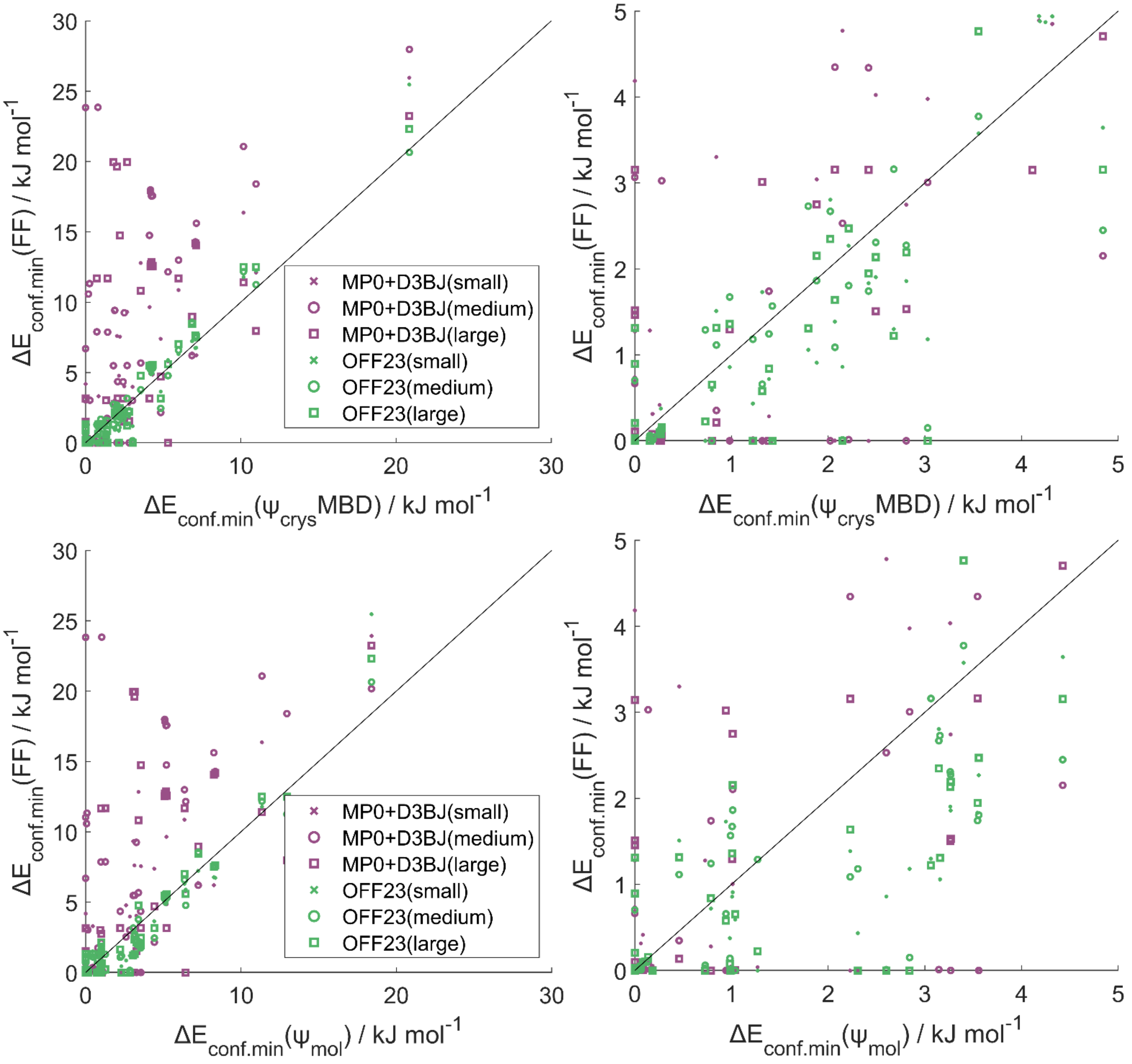

Figure S23. Scatterplots of the relative energies of the minimum energy conformations of the molecules investigated in this work, contrasting the MACE force-fields (FF) with the direct electronic structure calculations. Top – Energies of the optimized molecules calculated with ψ_{crys}(PBE+MBD) compared with the force field energies calculated starting from the ψ_{crys} optimized molecules. Bottom – Energies of the optimized molecules calculated with ψ_{mol} compared with the force field energies calculated starting from the ψ_{mol} optimized molecules. Left – the full energy range. Right – only points within 5 kJ mol^{-1} of the global minimum energy in both methods.

The conformation which was determined to be the lowest energy was not always the same with the MACE-OFF23 and MACE-MP-0+D3(BJ) optimizations. When rankings were switched, $\Delta E_{conf.min}$ could change by up to ~3 kJ mol^{-1} for DHC, CYH, MFA and TFA. However, for molecules where the conformation changed drastically (IBP, NAP and DES), rather large energy switches were observed.

Almost always, the conformation resulting from optimization starting from either the ψ_{mol} or ψ_{crys}(PBE+TS) conformations yielded the same energy, identical for MACE-OFF23 and within 0.02 kJ mol^{-1} for MACE-MP-0+D3(BJ). The exceptions were all with the MACE-MP-0+D3(BJ) optimizations, and were NFA_A with the large parameter set, CHA_planar with the small parameter set, IBP_SameAt1 with the medium parameter set, DES_SSE with the small

parameter set, and SMZ_opp with the small and medium parameter sets. Only in the case of the SMZ_opp conformation was the energy difference due to different local minima being reached from the slight differences in starting point.

S6.2.1 Conformational differences

MACE-OFF23 almost always reproduced the conformations optimized by ψ_{mol} and ψ_{crys}(PBE+TS), so there was generally good agreement between these three methods, with exceptions noted below. MACE-MP-0+D3(BJ) often gave different conformational minima, as detailed below. The differences in the isolated molecule conformational preferences can lead to poor reproduction of crystal structures, let alone lattice energies, particularly if this affects the position of the polar protons involved in hydrogen bonding or prevents the close packing of the molecules.

S6.2.1.1 Carbamazepine family

MACE-MP-0+D3(BJ) favoured non-planar nitrogen coordination at the central atom of CBZ, DHC and OXC. For these three molecules, MACE-MP-0+D3(BJ) favoured In configurations of the NH_2 group, whereas for CYH and CYT (which have a carbon atom at the centre), the NH_2 group tended to change to Out. Notwithstanding this merging of minima, the energy ranking of DHC and CYH was swapped with MACE-MP-0+D3(BJ) and the small and medium parameter sets.

S6.2.1.2 Fenamate family

The conformations resulting from optimization with the different models tended only to differ in the angle between the two aromatic rings. This varied across 20° for FEA, for example. NFA, with approximately planar molecules in the ψ_{mol} and ψ_{crys}(PBE+TS) optimized conformations, had angles between the rings of up to 30° with MACE-MP-0+D3(BJ). This change also occurred with MACE-OFF23 with the small parameter set. The lowest energy conformations of both MFA and TFA were switched with both foundation models and the large parameter set.

S6.2.1.3 Small drug molecules

CHA was the only case where the ψ_{mol} and ψ_{crys}(PBE+TS) optimized conformations differed, with three distinct minima for the former model and only two for the latter. MACE-OFF23 replicated the results of the ψ_{crys}(PBE+TS) optimizations, although MACE-MP-0+D3(BJ) optimizations sometimes replicated this and sometimes converted to the CHA_planar ψ_{mol} conformation, depending on starting point and parameter set size.

For IBP, the ψ_{mol} and ψ_{crys}(PBE+TS) optimized molecules (replicated by MACE-OFF23) had a hydrogen atom coplanar with the aromatic ring on both sides of the molecule. However, MACE-MP-0+D3(BJ) frequently altered the conformation to have a large group coplanar with the aromatic ring, which contradicts our intuition of steric interactions. Different conformations were observed as the lowest in energy with different foundation model / parameter set combinations, although energies were usually close.

In common with IBP, the isopropanoic acid group of NAP was frequently altered with MACE-MP-0+D3(BJ) so that a larger group was coplanar with the aromatic ring. While optimizations of IBP only resulted in the methyl group moving to this unfavorable position, with NAP, the acid group was found here on a few occasions. These conformational changes resulted in different conformations being found as the lowest in energy with MACE-MP-0+D3(BJ).

Optimization of DES conformations with MACE-MP-0+D3(BJ) sometimes changed the configuration of the ring amine's hydrogen atom to planar (defined as axial or equatorial in the starting conformations) and sometimes altered the angle at which the benzodiazepine group joined the 6-membered ring. Combinations of these two factors led to strange-looking conformations on some occasions. The large parameter set was more reliable in retaining the starting conformation, but only for conformations where the piperidine ring pointed away from the benzodiazepine group. Again, these conformational changes were coupled with large changes in $\Delta E_{conf.min}$, from +2 to -24 for DES_SAA vs DES_AAA with MACE-MP-0+D3(BJ) and the medium parameter set.

SMZ molecules changed considerably with MACE-MP-0+D3(BJ) optimization, notably in the angle between the SO_2 group and the neighbouring aromatic ring. Angles measured in this region for SMZ_ext differed across 3° between ψ_{mol}, ψ_{crys}(PBE+TS) and MACE-OFF23 optimized conformations, but by almost 30° for MACE-MP-0+D3(BJ)

optimized conformations. This same angle varied considerably in the other conformations, and SMZ_opp was optimized to two distinct conformations with MACE-MP-0+D3(BJ) with the small and medium parameter sets, significantly altering the gross shape of the molecule.

S7 Notes on provided datasets of computed crystal and molecular structures

Two .cif files with the ψ_{mol} and ψ_{crys}(PBE+TS) optimized crystal structures are included as Supplementary Information. These are named All_Psi_Mol.cif and All_Psi_Crys.cif respectively.

The four crystal structures which could not be optimized with the ψ_{crys}(PBE+TS) method are included in a separate .cif, called Additional_Psi_Mol.cif.

Two .mol files with the ψ_{mol} and ψ_{crys}(PBE+TS) optimized molecular conformations are also included. These are named All_Psi_Mol.mol and All_Psi_Crys.mol respectively.

S7.1 ψ_{mol} optimized crystal structures

CrystalOptimizer writes out crystal structures as .res files. These have been converted to .cif with no other alterations, and concatenated into one file.

S7.2 ψ_{crys}(PBE+TS) optimized crystal structures

CASTEP writes out crystal structures as .cell files, and these were converted to .res using cell2shx, provided by the CASTEP developers. These contained all the atoms of the entire unit cell, so were inspected with Platon and ADDSYM run to convert to a more normal crystal structure. This process removes atom labels, so these were added back in manually in Mercury to match the atom labels and ordering of the CrystalOptimizer files as far as possible.

In some cases, the symmetry adding procedure with PLATON gave the opposite hand of the molecule in the CASTEP-derived structure file, compared with the CrystalOptimizer-derived structure file. These have not been altered.

S7.3 Structures where symmetry added by Platon required special consideration

S7.3.1 CRN

Platon determined that many of the CASTEP-derived crystal structures of CRN had half molecules with inversion centres generating the rest of the molecule. These have been left as Z'=0.5 crystal structures. However, converting .res to .cif with the CCDC's tools generated the other halves of the molecules which led to complications with the testing with machine-learned potentials. These extra atoms were removed manually from the .cif files, and all bonding information (automatically generated with the CCDC's tools conversion) was also removed.

S7.3.2 PTH

Platon increased the symmetry of CASTEP-derived PTH03 to a structure which did not contain the correct molecule, and so the *P*1 structure is kept.

S7.3.3 FLU

PLATON determined incorrectly that FLU03 could be represented as a Z'=(two halves) structure, with a plane of symmetry running through O1-C1-C3-F1 in the molecule. Since this is not the correct molecular structure, the *P*1 structure is kept.

PLATON determined that FLU06 could be represented as a Z'=0.5 Pnma crystal structure, with the molecule on a mirror plane. This led to complications with the testing with machine-learned potentials, and so the symmetry was reduced to Z'=1 $P2_12_12_1$.

S7.3.4 FEA

Platon increased the symmetry of CASTEP-derived FEA14 to a structure which did not contain the correct molecule, and so the *P*1 structure is kept.

S7.3.5 MFA

The CrystalOptimizer-derived structure, MFA12, was highlighted as being a saddlepoint by the DMACRYS analysis, and so the symmetry was reduced from $P2_1/c$ to $P2_1$ in NEIGHCRYS and reminimized. However, following minimization of the resulting structure with CASTEP, PLATON determined that it could be represented as a Z′=1, $P2_1/c$ structure.

The CrystalOptimizer-derived structure, MFA15, was highlighted as being a saddlepoint by the DMACRYS analysis, and so the symmetry was reduced from $P2_1/c$ to $P2_1$ in NEIGHCRYS and reminimized. However, following minimization of the resulting structure with CASTEP, PLATON determined that it could be represented as a Z′=1, $P2_1/c$ structure.

S7.3.6 TFA

Platon determined that the CASTEP-derived equivalent to TFA I (TFA01) could be represented as a Z′=1, $P2_1/c$ structure. This gave a better overlay with the structure reported on the CSD (KAXXAI01, Z′=2, $P2_1$), so this higher symmetry structure has been retained.

The CrystalOptimizer-derived structure equivalent to TFA VII (TFA07) was highlighted as being a saddlepoint by the DMACRYS analysis, and so the symmetry was reduced from $P2_1/n$ to $P2_1$ in NEIGHCRYS and reminimized. However, following minimization of the resulting structure with CASTEP, PLATON determined that it could be represented as a Z′=1, $P2_1/c$ structure. The two computationally minimized structures gave poor overlays to the structure on the CSD (KAXXAI05, Z′=1, $P2_1/n$), so it is not clear which is the better representation.

S7.3.7 NFA

The CrystalOptimizer-derived structure for NFA in the TFA III packing (NFA15) was highlighted as being a saddlepoint by the DMACRYS analysis, and so the symmetry was reduced from $C2/c$ to $P2_1/c$ in NEIGHCRYS and reminimized. However, following minimization of the resulting structure with CASTEP, PLATON determined that it could be represented as a Z′=1, $C2/c$ structure.

S7.3.8 IBP

PLATON determined that the CASTEP-derived equivalent to racemic form II (IBP03) could be represented as a Z′=1, $P2_1/c$ structure. Both computationally minimized structures give poor overlays with the structure reported on the CSD (IBPRAC04, Z′=2, $P\bar{1}$), so this higher symmetry structure has been retained as it is not clear that it is any more incorrect than the Z′=2 model for this polymorph.

S7.3.9 NAP

The experimentally observed form of NAP was found in the search as NAP03. This was highlighted as being a saddlepoint by the DMACRYS analysis, and so the symmetry was reduced from *Pbca* to $Pbc2_1$ in NEIGHCRYS and reminimized as NAP01. Following minimization with CASTEP, Platon identified that NAP01 was in fact a Z′=1 structure in *Pbca* making it equivalent again to NAP03. We have retained the *P*1 Z′=8 version of NAP01 as the ψ_{crys} structure.

NAP03 was also optimized with CASTEP. Adding symmetry to the resulting structure with Platon yielded the expected *Pbca* structure.

S8 Full author lists for truncated references

S8.1 From main paper

(1) Burcham, C. L., Doherty, M. F., Peters, B. G., Price, S. L., Salvalaglio, M., Reutzel-Edens, S. M., Price, L. S., Addula, R. K. R., Francia, N., Khanna, V., and Zhao, Y. Pharmaceutical Digital Design: From Chemical Structure through Crystal Polymorph to Conceptual Crystallization Process. *Crystal Growth & Design* **2024**, *24* (13), 5417-5438. DOI: 10.1021/acs.cgd.3c01390.

(15) Reid, D. L., Faul, M. M., López-Mejías, V., Agarwal, P., Bergauer, M., Blue, L. E., Chaves, M. K., Chung, J. H., Cooke, M., Farrell, R. P., Huckle, J. E., Kelly, R. C., Kiang, Y. H., Li, W. K., Ortiz, A., and Wu, Q. Application of an Interdisciplinary

Approach to Form Selection in Drug Development. *Organic Process Research & Development* **2025**, *29* (2), 237-254. DOI: 10.1021/acs.oprd.4c00320

(22) Reilly, A. M., Cooper, R. I., Adjiman, C. S., Bhattacharya, S., Boese, A. D., Brandenburg, J. G., Bygrave, P. J., Bylsma, R., Campbell, J. E., Car, R., Case, D. H., Chadha, R., Cole, J. C., Cosburn, K., Cuppen, H. M., Curtis, F., Day, G. M., DiStasio Jr, R. A., Dzyabchenko, A., van Eijck, B. P., Elking, D. M., van den Ende, J. A., Facelli, J. C., Ferraro, M. B., Fusti-Molnar, L., Gatsiou, C.-A., Gee, T. S., de Gelder, R., Ghiringhelli, L. M., Goto, H., Grimme, S., Guo, R., Hofmann, D. W. M., Hoja, J., Hylton, R. K., Iuzzolino, L., Jankiewicz, W., de Jong, D. T., Kendrick, J., de Klerk, N. J. J., Ko, H.-Y., Kuleshova, L. N., Li, X., Lohani, S., Leusen, F. J. J., Lund, A. M., Lv, J., Ma, Y., Marom, N., Masunov, A. E., McCabe, P., McMahon, D. P., Meekes, H., Metz, M. P., Misquitta, A. J., Mohamed, S., Monserrat, B., Needs, R. J., Neumann, M. A., Nyman, J., Obata, S., Oberhofer, H., Oganov, A. R., Orendt, A. M., Pagola, G. I., Pantelides, C. C., Pickard, C. J., Podeszwa, R., Price, L. S., Price, S. L., Pulido, A., Read, M. G., Reuter, K., Schneider, E., Schober, C., Shields, G. P., Singh, P., Sugden, I. J., Szalewicz, K., Taylor, C. R., Tkatchenko, A., Tuckerman, M. E., Vacarro, F., Vasileiadis, M., Vazquez-Mayagoitia, A., Vogt, L., Wang, Y., Watson, R. E., de Wijs, G. A., Yang, J., Zhu, Q., and Groom, C. R. Report on the sixth blind test of organic crystal structure prediction methods. *Acta Crystallographica Section B* **2016**, *72* (4), 439-459. DOI: 10.1107/S2052520616007447.

(34) Day, G. M., Cooper, T. G., Cruz-Cabeza, A. J., Hejczyk, K. E., Ammon, H. L., Boerrigter, S. X. M., Tan, J., Della Valle, R. G., Venuti, E., Jose, J., Gadre, S. R., Desiraju, G. R., Thakur, T. S., van Eijck, B. P., Facelli, J. C., Bazterra, V. E., Ferraro, M. B., Hofmann, D. W. M., Neumann, M., Leusen, F. J. J., Kendrick, J., Price, S. L., Misquitta, A. J., Karamertzanis, P. G., Welch, G. W. A., Scheraga, H. A., Arnautova, Y. A., Schmidt, M. U., van de Streek, J., Wolf, A., and Schweizer, B. Significant progress in predicting the crystal structures of small organic molecules - a report on the fourth blind test. *Acta Crystallographica Section B - Structural Science* **2009**, *65* (2), 107-125.

(44) Hunnisett, L. M., Nyman, J., Francia, N., Abraham, N. S., Adjiman, C. S., Aitipamula, S., Alkhidir, T., Almehairbi, M., Anelli, A., Anstine, D. M., Anthony, J. E., Arnold, J. E., Bahrami, F., Bellucci, M. A., Bhardwaj, R. M., Bier, I., Bis, J. A., Boese, A. D., Bowskill, D. H., Bramley, J., Brandenburg, J. G., Braun, D. E., Butler, P. W. V., Cadden, J., Carino, S., Chan, E. J., Chang, C., Cheng, B., Clarke, S. M., Coles, S. J., Cooper, R. I., Couch, R., Cuadrado, R., Darden, T., Day, G. M., Dietrich, H., Ding, Y., DiPasquale, A., Dhokale, B., van Eijck, B. P., Elsegood, M. R. J., Firaha, D., Fu, W., Fukuzawa, K., Glover, J., Goto, H., Greenwell, C., Guo, R., Harter, J., Helfferich, J., Hofmann, D. W. M., Hoja, J., Hone, J., Hong, R., Hutchison, G., Ikabata, Y., Isayev, O., Ishaque, O., Jain, V., Jin, Y., Jing, A., Johnson, E. R., Jones, I., Jose, K. V. J., Kabova, E. A., Keates, A., Kelly, P. F., Khakimov, D., Konstantinopoulos, S., Kuleshova, L. N., Li, H., Lin, X., List, A., Liu, C., Liu, Y. M., Liu, Z., Liu, Z.-P., Lubach, J. W., Marom, N., Maryewski, A. A., Matsui, H., Mattei, A., Mayo, R. A., Melkumov, J. W., Mohamed, S., Momenzadeh Abardeh, Z., Muddana, H. S., Nakayama, N., Nayal, K. S., Neumann, M. A., Nikhar, R., Obata, S., O'Connor, D., Oganov, A. R., Okuwaki, K., Otero-de-la-Roza, A., Pantelides, C. C., Parkin, S., Pickard, C. J., Pilia, L., Pivina, T., Podeszwa, R., Price, A. J. A., Price, L. S., Price, S. L., Probert, M. R., Pulido, A., Ramteke, G. R., Rehman, A. U., Reutzel-Edens, S. M., Rogal, J., Ross, M. J., Rumson, A. F., Sadiq, G., Saeed, Z. M., Salimi, A., Salvalaglio, M., Sanders de Almada, L., Sasikumar, K., Sekharan, S., Shang, C., Shankland, K., Shinohara, K., Shi, B., Shi, X., Skillman, A. G., Song, H., Strasser, N., van de Streek, J., Sugden, I. J., Sun, G., Szalewicz, K., Tan, B. I., Tan, L., Tarczynski, F., Taylor, C. R., Tkatchenko, A., Tom, R., Tuckerman, M. E., Utsumi, Y., Vogt-Maranto, L., Weatherston, J., Wilkinson, L. J., Willacy, R. D., Wojtas, L., Woollam, G. R., Yang, Z., Yonemochi, E., Yue, X., Zeng, Q., Zhang, Y., Zhou, T., Zhou, Y., Zubatyuk, R., and Cole, J. C., The seventh blind test of crystal structure prediction: structure generation methods. *Acta Crystallographica Section B* **2024**, *80* (6), 517-547.

(45) Hunnisett, L. M., Francia, N., Nyman, J., Abraham, N. S., Aitipamula, S., Alkhidir, T., Almehairbi, M., Anelli, A., Anstine, D. M., Anthony, J. E., Arnold, J. E., Bahrami, F., Bellucci, M. A., Beran, G. J. O., Bhardwaj, R. M., Bianco, R., Bis, J. A., Boese, A. D., Bramley, J., Braun, D. E., Butler, P. W. V., Cadden, J., Carino, S., Cervinka, C., Chan, E. J., Chang, C., Clarke, S. M., Coles, S. J., Cook, C. J., Cooper, R. I., Darden, T., Day, G. M., Deng, W., Dietrich, H., DiPasquale, A., Dhokale, B., van Eijck, B. P., Elsegood, M. R. J., Firaha, D., Fu, W., Fukuzawa, K., Galanakis, N., Goto, H., Greenwell, C., Guo, R., Harter, J., Helfferich, J., Hoja, J., Hone, J., Hong, R., Husak, M., Ikabata, Y., Isayev, O., Ishaque, O., Jain, V., Jin, Y., Jing, A., Johnson, E. R., Jones, I., Jose, K. V. J., Kabova, E. A., Keates, A., Kelly, P. F., Klimes, J., Kostkova, V., Li, H., Lin, X., List, A., Liu, C., Liu, Y. M., Liu, Z., Loncaric, I., Lubach, J. W., Ludik, J., Marom, N., Matsui, H., Mattei, A., Mayo, R. A., Melkumov, J. W., Mladineo, B., Mohamed, S., Momenzadeh Abardeh, Z., Muddana, H. S., Nakayama, N., Nayal, K. S.,

Neumann, M. A., Nikhar, R., Obata, S., O'Connor, D., Oganov, A. R., Okuwaki, K., Otero-de-la-Roza, A., Parkin, S., Parunov, A., Podeszwa, R., Price, A. J. A., Price, L. S., Price, S. L., Probert, M. R., Pulido, A., Ramteke, G. R., Rehman, A. U., Reutzel-Edens, S. M., Rogal, J., Ross, M. J., Rumson, A. F., Sadiq, G., Saeed, Z. M., Salimi, A., Sasikumar, K., Sekharan, S., Shankland, K., Shi, B., Shi, X., Shinohara, K., Skillman, A. G., Song, H., Strasser, N., van de Streek, J., Sugden, I. J., Sun, G., Szalewicz, K., Tan, L., Tang, K., Tarczynski, F., Taylor, C. R., Tkatchenko, A., Tom, R., Tous, P., Tuckerman, M. E., Unzueta, P. A., Utsumi, Y., Vogt-Maranto, L., Weatherston, J., Wilkinson, L. J., Willacy, R. D., Wojtas, L., Woollam, G. R., Yang, Y., Yang, Z., Yonemochi, E., Yue, X., Zeng, Q., Zhou, T., Zhou, Y., Zubatyuk, R., and Cole, J. C. The seventh blind test of crystal structure prediction: structure ranking methods. *Acta Crystallographica Section B* **2024**, *80* (6) 548-574.

(78) Hjorth Larsen, A., Jørgen Mortensen, J., Blomqvist, J., Castelli, I. E., Christensen, R., Dułak, M., Friis, J., Groves, M. N., Hammer, B., Hargus, C., Hermes, E. D., Jennings, P. C., Bjerre Jensen, P., Kermode, J., Kitchin, J. R., Leonhard Kolsbjerg, E., Kubal, J., Kaasbjerg, K., Lysgaard, S., Bergmann Maronsson, J., Maxson, T., Olsen, T., Pastewka, L., Peterson, A., Rostgaard, C., Schiøtz, J., Schütt, O., Strange, M., Thygesen, K. S., Vegge, T., Vilhelmsen, L., Walter, M., Zeng, Z., and Jacobsen, K. W. The atomic simulation environment-a Python library for working with atoms. *Journal of Physics-Condensed Matter* **2017**, *29* (27), 273002. DOI: 10.1088/1361-648X/aa680e.

(80) Kovács, D. P., Moore, J. H., Browning, N. J., Batatia, I., Horton, J. T., Pu, Y., Kapil, V., Witt, W. C., Magdău, I.-B., Cole, D. J., and Csányi, G., 2023, "MACE-OFF23: Transferable machine learning force fields for organic molecules," arXiv preprint arXiv:2312.15211.

(81) Batatia, I., Benner, P., Chiang, Y., Elena, A. M., Kovács, D. P., Riebesell, J., Advincula, X. R., Asta, M., Avaylon, M., Baldwin, W. J., Berger, F., Bernstein, N., Bhowmik, A., Blau, S. M., Cărare, V., Darby, J. P., De, S., Pia, F. D., Deringer, V. L., Elijošius, R., El-Machachi, Z., Falcioni, F., Fako, E., Ferrari, A. C., Genreith-Schriever, A., George, J., Goodall, R. E. A., Grey, C. P., Grigorev, P., Han, S., Handley, W., Heenen, H. H., Hermansson, K., Holm, C., Jaafar, J., Hofmann, S., Jakob, K. S., Jung, H., Kapil, V., Kaplan, A. D., Karimitari, N., Kermode, J. R., Kroupa, N., Kullgren, J., Kuner, M. C., Kuryla, D., Liepuoniute, G., Margraf, J. T., Magdău, I.-B., Michaelides, A., Moore, J. H., Naik, A. A., Niblett, S. P., Norwood, S. W., O'Neill, N., Ortner, C., Persson, K. A., Reuter, K., Rosen, A. S., Schaaf, L. L., Schran, C., Shi, B. X., Sivonxay, E., Stenczel, T. K., Svahn, V., Sutton, C., Swinburne, T. D., Tilly, J., van der Oord, C., Varga-Umbrich, E., Vegge, T., Vondrák, M., Wang, Y., Witt, W. C., Zills, F., and Csányi, G., 2023, "A foundation model for atomistic materials chemistry," arXiv preprint arXiv:2401.00096.

(84) Potticary, J., Terry, L. R., Bell, C., Papanikolopoulos, A. N., Christianen, P. C. M., Engelkamp, H., Collins, A. M., Fontanesi, C., Kociok-Kohn, G., Crampin, S., Da Como, E., and Hall, S. R. An unforeseen polymorph of coronene by the application of magnetic fields during crystal growth. *Nature Communications* **2016**, *7*, 11555. DOI: 10.1038/ncomms11555.

(88) Hulme, A. T., Johnston, A., Florence, A. J., Fernandes, P., Shankland, K., Bedford, C. T., Welch, G. W. A., Sadiq, G., Haynes, D. A., Motherwell, W. D. S., Tocher, D. A., and Price, S. L. Search for a predicted hydrogen bonding motif - A multidisciplinary investigation into the polymorphism of 3-azabicyclo[3.3.1]nonane-2,4-dione. *Journal of the American Chemical Society* **2007**, *129* (12), 3649-3657.

(99) Jones, J. T. A., Hasell, T., Wu, X. F., Bacsa, J., Jelfs, K. E., Schmidtmann, M., Chong, S. Y., Adams, D. J., Trewin, A., Schiffman, F., Cora, F., Slater, B., Steiner, A., Day, G. M., and Cooper, A. I. Modular and predictable assembly of porous organic molecular crystals. *Nature* **2011**, *474* (7351), 367-371.

(122) Schmidt, M. U., Bruning, J., Glinnemann, J., Hutzler, M. W., Morschel, P., Ivashevskaya, S. N., van de Streek, J., Braga, D., Maini, L., Chierotti, M. R., and Gobetto, R. The Thermodynamically Stable Form of Solid Barbituric Acid: The Enol Tautomer. *Angewandte Chemie-International Edition* **2011**, *50* (34), 7924-7926.

(162) Bannan, C. C., Ovanesyan, G., Darden, T. A., Graves, A. P., Edge, C. M., Russo, L., Copley, R. C. B., Manas, E., Skillman, A. G., Nicholls, A., and Muddana, H. S. Crystal Structure Prediction of Drug Molecules in the Cloud: A Collaborative Blind Challenge Study. *Crystal Growth & Design* **2025** *25* (5), 1299-1314. DOI: 10.1021/acs.cgd.4c00572.

(170) Firaha, D., Liu, Y. M., van de Streek, J., Sasikumar, K., Dietrich, H., Helfferich, J., Aerts, L., Braun, D. E., Broo, A., DiPasquale, A. G., Lee, A. Y., Le Meur, S., Nilsson Lill, S. O., Lunsmann, W. J., Mattei, A., Muglia, P., Putra, O. D., Raoui, M., Reutzel-Edens, S. M., Rome, S., Sheikh, A. Y., Tkatchenko, A., Woollam, G. R., and Neumann, M. A., 2023, "Predicting crystal form stability under real-world conditions," Nature, 623(7986), 324-328.

S8.2 From SI

(3) Potticary, J., Terry, L. R., Bell, C., Papanikolopoulos, A. N., Christianen, P. C. M., Engelkamp, H., Collins, A. M., Fontanesi, C., Kociok-Kohn, G., Crampin, S., Da Como, E., and Hall, S. R. An unforeseen polymorph of coronene by the application of magnetic fields during crystal growth. *Nature Communications* **2016**, *7*, 11555. DOI: 10.1038/ncomms11555.

(7) Motherwell, W. D. S., Ammon, H. L., Dunitz, J. D., Dzyabchenko, A., Erk, P., Gavezzotti, A., Hofmann, D. W. M., Leusen, F. J. J., Lommerse, J. P. M., Mooij, W. T. M., Price, S. L., Scheraga, H., Schweizer, B., Schmidt, M. U., van Eijck, B. P., Verwer, P., and Williams, D. E. Crystal structure prediction of small organic molecules: a second blind test. *Acta Crystallographica Section B - Structural Science* **2002**, *58*, 647-661.

(31) Kataeva, O., Khrizanforov, M., Budnikova, Y., Islamov, D., Burganov, T., Vandyukov, A., Lyssenko, K., Mahns, B., Nohr, M., Hampel, S., and Knupfer, M. Crystal Growth, Dynamic and Charge Transfer Properties of New Coronene Charge Transfer Complexes. *Crystal Growth & Design* **2016**, *16* (1), 331-338. DOI: 10.1021/acs.cgd.5b01301.

S9 Supporting Information References

(1) Potticary, J.; Hall, C. L.; Guo, R.; Price, S. L.; Hall, S. R. On the Application of Strong Magnetic Fields during Organic Crystal Growth. *Crystal Growth & Design* **2021**, *21* (11), 6254-6265. DOI: 10.1021/acs.cgd.1c00723.
(2) Bannister, N.; Skelton, J.; Kociok-Köhn, G.; Batten, T.; Da Como, E.; Crampin, S. Lattice vibrations of gamma- and beta- coronene from Raman microscopy and theory. *Physical Review Materials* **2019**, *3* (12), 125601. DOI: 10.1103/PhysRevMaterials.3.125601.
(3) Potticary, J.; Terry, L. R.; Bell, C.; Papanikolopoulos, A. N.; Christianen, P. C. M.; Engelkamp, H.; Collins, A. M.; Fontanesi, C.; Kociok-Kohn, G.; Crampin, S.; et al. An unforeseen polymorph of coronene by the application of magnetic fields during crystal growth. *Nature Communications* **2016**, *7*, 11555. DOI: 10.1038/ncomms11555.
(4) Schur, E.; Bernstein, J.; Price, L. S.; Guo, R.; Price, S. L.; Lapidus, S. H.; Stephens, P. W. The (Current) Acridine Solid Form Landscape: Eight Polymorphs and a Hydrate. *Crystal Growth & Design* **2019**, *9* (18), 4884-4893. DOI: 10.1021/acs.cgd.9b00557.
(5) Braga, D.; Grepioni, F.; Maini, L.; Mazzeo, P. P.; Rubini, K. Solvent-free preparation of co-crystals of phenazine and acridine with vanillin. *Thermochimica Acta* **2010**, *507-508*, 1-8.
(6) Corpinot, M. K.; Guo, R.; Tocher, D. A.; Buanz, A. B. M.; Gaisford, S.; Price, S. L.; Bucar, D. K. Are Oxygen and Sulfur Atoms Structurally Equivalent in Organic Crystals? *Crystal Growth & Design* **2017**, *17* (2), 827-833, Article. DOI: 10.1021/acs.cgd.6b01669.
(7) Motherwell, W. D. S.; Ammon, H. L.; Dunitz, J. D.; Dzyabchenko, A.; Erk, P.; Gavezzotti, A.; Hofmann, D. W. M.; Leusen, F. J. J.; Lommerse, J. P. M.; Mooij, W. T. M.; et al. Crystal structure prediction of small organic molecules: a second blind test. *Acta Crystallographica Section B - Structural Science* **2002**, *58*, 647-661.
(8) Hulme, A. T.; Price, S. L.; Tocher, D. A. A New Polymorph of 5-Fluorouracil Found Following Computational Crystal Structure Predictions. *Journal of the American Chemical Society* **2005**, *127* (4), 1116-1117.
(9) Arlin, J. B.; Price, L. S.; Price, S. L.; Florence, A. J. A strategy for producing predicted polymorphs: catemeric carbamazepine form V. *Chemical Communications* **2011**, *47* (25), 7074-7076.
(10) Srirambhatla, V. K.; Guo, R.; Price, S. L.; Florence, A. J. Isomorphous template induced crystallisation: a robust method for the targeted crystallisation of computationally predicted metastable polymorphs. *Chemical Communications* **2016**, *52*, 7384-7386, 10.1039/C6CC01710J. DOI: 10.1039/C6CC01710J.
(11) Polyzois, H.; Guo, R.; Srirambhatla, V. K.; Warzecha, M.; Prasad, E.; Turner, A.; Halbert, G. W.; Keating, P.; Price, S. L.; Florence, A. J. Crystal Structure and Twisted Aggregates of Oxcarbazepine Form III. *Crystal Growth & Design* **2022**, *22* (7), 4146-4156. DOI: 10.1021/acs.cgd.2c00152.
(12) Fabbiani, F. P. A.; Byrne, L. T.; McKinnon, J. J.; Spackman, M. A. Solvent inclusion in the structural voids of form II carbamazepine: single-crystal X-ray diffraction, NMR spectroscopy and Hirshfeld surface analysis. *CrystEngComm* **2007**, *9* (9), 728-731.

(13) Grzesiak, A. L.; Lang, M. D.; Kim, K.; Matzger, A. J. Comparison of the four anhydrous polymorphs of carbamazepine and the crystal structure of form I. *Journal of Pharmaceutical Sciences* **2003**, *92* (11), 2260-2271.
(14) Arlin, J. B.; Johnston, A.; Miller, G. J.; Kennedy, A. R.; Price, S. L.; Florence, A. J. A predicted dimer-based polymorph of 10,11-dihydrocarbamazepine (Form IV). *CrystEngComm* **2010**, *12* (1), 64-66.
(15) Florence, A. J.; Shankland, K.; Gelbrich, T.; Hursthouse, M. B.; Shankland, N.; Johnston, A.; Fernandes, P.; Leech, C. K. A catemer-to-dimer structural transformation in cyheptamide. *CrystEngComm* **2008**, *10*, 26-28.
(16) Florence, A. J.; Bedford, C. T.; Fabbiani, F. P. A.; Shankland, K.; Gelbrich, T.; Hursthouse, M. B.; Shankland, N.; Johnston, A.; Fernandes, P. Two-dimensional similarity between forms I and II of cytenamide, a carbamazepine analogue. *CrystEngComm* **2008**, *10* (7), 811-813.
(17) Lutker, K. M.; Matzger, A. J. Crystal polymorphism in a carbamazepine derivative: Oxcarbazepine. *Journal of Pharmaceutical Sciences* **2010**, *99* (2), 794-803.
(18) Uzoh, O. G.; Cruz-Cabeza, A. J.; Price, S. L. Is the Fenamate Group a Polymorphophore? Contrasting the Crystal Energy Landscapes of Fenamic and Tolfenamic Acids. *Crystal Growth & Design* **2012**, *12* (8), 4230-4239. DOI: 10.1021/cg3007348.
(19) Case, D. H.; Srirambhatla, V. K.; Guo, R.; Watson, R. E.; Price, L. S.; Polyzois, H.; Cockcroft, J. K.; Florence, A. J.; Tocher, D. A.; Price, S. L. Successful Computationally Directed Templating of Metastable Pharmaceutical Polymorphs. *Crystal Growth & Design* **2018**, *18* (9), 5322-5331. DOI: 10.1021/acs.cgd.8b00765.
(20) Bag, P. P.; Reddy, C. M. Screening and Selective Preparation of Polymorphs by Fast Evaporation Method: A Case Study of Aspirin, Anthranilic Acid, and Niflumic Acid. *Crystal Growth & Design* **2012**, *12* (6), 2740-2743, Article. DOI: 10.1021/cg300404r.
(21) Abbas, N.; Oswald, I. D. H.; Pulham, C. R. Accessing Mefenamic Acid Form II through High-Pressure Recrystallisation. *Pharmaceutics* **2017**, *9* (2), 16. DOI: 10.3390/pharmaceutics9020016.
(22) Sacchi, P.; Reutzel-Edens, S. M.; Cruz-Cabeza, A. J. The unexpected discovery of the ninth polymorph of tolfenamic acid. *CrystEngComm* **2021**, *23*, 3636-3647. DOI: 10.1039/d1ce00343g.
(23) Ludík, J.; Kostková, V.; Kocian, Š.; Touš, P.; Štejfa, V.; Červinka, C. First-Principles Models of Polymorphism of Pharmaceuticals: Maximizing the Accuracy-to-Cost Ratio. *Journal of Chemical Theory and Computation* **2024**, *20* (7), 2858-2870. DOI: 10.1021/acs.jctc.4c00099.
(24) Price, L. S.; Price, S. L. Packing Preferences of Chalcones: A Model Conjugated Pharmaceutical Scaffold. *Crystal Growth & Design* **2022**, *22* (3), 1801-1816. DOI: 10.1021/acs.cgd.1c01381.
(25) Francia, N. F.; Price, L. S.; Salvalaglio, M. Reducing crystal structure overprediction of ibuprofen with large scale molecular dynamics simulations. *CrystEngComm* **2021**, *23* (33), 5575-5584, 10.1039/D1CE00616A. DOI: 10.1039/D1CE00616A.
(26) Derollez, P.; Correia, N. T.; Danede, F.; Capet, F.; Affouard, F.; Lefebvre, J.; Descamps, M. Ab initio structure determination of the high temperature phase of anhydrous caffeine by x-ray powder diffraction. *Acta Crystallographica Section B - Structural Science* **2005**, *61* (3), 329-334.
(27) Iuzzolino, L.; McCabe, P.; Price, S. L.; Brandenburg, J. G. Crystal structure prediction of flexible pharmaceutical-like molecules: density functional tight-binding as an intermediate optimisation method and for free energy estimation. *Faraday Discussions* **2018**, *211*, 275-296. DOI: 10.1039/C8FD00010G.
(28) Braun, D. E.; Ardid-Candel, M.; D'Oria, E.; Karamertzanis, P. G.; Arlin, J. B.; Florence, A. J.; Jones, A. G.; Price, S. L. Racemic Naproxen: A Multidisciplinary Structural and Thermodynamic Comparison with the Enantiopure Form. *Crystal Growth & Design* **2011**, *11* (12), 5659-5669.
(29) Srirambhatla, V. K.; Guo, R.; Dawson, D. M.; Price, S. L.; Florence, A. J. Reversible, Two-Step Single-Crystal to Single-Crystal Phase Transitions between Desloratadine Forms I, II, and III. *Crystal Growth & Design* **2020**, *20* (3), 1800-1810. DOI: 10.1021/acs.cgd.9b01522.
(30) Zhang, G. G. Z.; Gu, C.; Zell, M. T.; Burkhardt, R. T.; Munson, E. J.; Grant, D. J. W. Crystallization and Transitions of Sulfamerazine Polymorphs. *Journal of pharmaceutical sciences* **2002**, *91* (4), 1089-1100. DOI: 10.1002/jps.10100.
(31) Kataeva, O.; Khrizanforov, M.; Budnikova, Y.; Islamov, D.; Burganov, T.; Vandyukov, A.; Lyssenko, K.; Mahns, B.; Nohr, M.; Hampel, S.; et al. Crystal Growth, Dynamic and Charge Transfer Properties of New Coronene Charge Transfer Complexes. *Crystal Growth & Design* **2016**, *16* (1), 331-338. DOI: 10.1021/acs.cgd.5b01301.
(32) Fawcett, J. K.; Trotter, J. The crystal and molecular structure of coronene. *Proceedings of the Royal Society of London. Series A. Mathematical and Physical Sciences* **1966**, *289* (1418), 366-376.
(33) Chen, Y.; Chang, Z.; Zhang, J.; Gong, J. Bending for Better: Flexible Organic Single Crystals with Controllable Curvature and Curvature-Related Conductivity for Customized Electronic Devices. *Angewandte Chemie International Edition* **2021**, *60* (41), 22424-22431. DOI: 10.1002/anie.202108441.

(34) Mei, X. F.; Wolf, C. Formation of new polymorphs of acridine using dicarboxylic acids as crystallization templates in solution. *Crystal Growth & Design* **2004**, *4* (6), 1099-1103. DOI: 10.1021/cg0498655.
(35) Kupka, A.; Vasylyeva, V.; Hofmann, D.; Yusenko, K. V.; Merz, K. Solvent and Isotopic Effects on Acridine and Deuterated Acridine Polymorphism. *Crystal Growth & Design* **2012**, *12* (12), 5966-5971.
(36) Stephens, P. W.; Schur, E.; Lapidus, S. H.; Bernstein, J. Acridine form IX. *Acta Crystallographica Section E* **2019**, *75* (4), 489-491. DOI: 10.1107/S2056989019003645.
(37) Zakaria, C. M.; Low, J. N.; Glidewell, C. Phthalimide at 120 K: perforated molecular ribbons containing three different ring motifs. *Acta Crystallographica Section C - Crystal Structure Communications* **2002**, *58* (1), o9-o10.
(38) Du, J. J.; Váradi, L.; Williams, P. A.; Groundwater, P. W.; Overgaard, J.; Platts, J. A.; Hibbs, D. E. An analysis of the experimental and theoretical charge density distributions of the piroxicam–saccharin co-crystal and its constituents. *RSC Advances* **2016**, *6* (85), 81578-81590. DOI: 10.1039/C6RA10411H.
(39) Fernandes, P.; Shankland, K.; Florence, A. J.; Shankland, N.; Johnston, A. Solving molecular crystal structures from X-ray powder diffraction data: The challenges posed by gamma-carbamazepine and chlorothiazide N,N,-dimethylformamide (1/2) solvate. *Journal of Pharmaceutical Sciences* **2007**, *96* (5), 1192-1202.
(40) Lowes, M. M. J.; Caira, M. R.; Lotter, A. P.; Vanderwatt, J. G. Physicochemical Properties and X-Ray Structural Studies of the Trigonal Polymorph of Carbamazepine. *Journal of Pharmaceutical Sciences* **1987**, *76* (9), 744-752.
(41) Himes, V. L.; Mighell, A. D.; DeCamp, W. H. Structure of carbamazepine: 5 H -dibenz[b , f]azepine-5-carboxamide. *Acta Crystallographica Section B - Structural Crystallography and Crystal Chemistry* **1981**, *37* (12), 2242-2245.
(42) Lang, M. D.; Kampf, J. W.; Matzger, A. J. Form IV of carbamazepine. *Journal of Pharmaceutical Sciences* **2002**, *91* (4), 1186-1190.
(43) Bandoli, G.; Nicolini, M.; Ongaro, A.; Volpe, G.; Rubello, A. X-ray Crystallographic Characterization of 10,11-Dihydro-5H-dibenz[b,f]azepine-5-carboxamide. *Journal of Crystallographic and Spectroscopic Research* **1992**, *22* (2), 177-183.
(44) Harrison, W. T. A.; Yathirajan, H. S.; Anilkumar, H. G. An orthorhombic polymorph of 10,11-dihydrocarbamazepine. *Acta Crystallographica Section C - Crystal Structure Communications* **2006**, *62*, o240-o242.
(45) Leech, C. K.; Florence, A. J.; Shankland, K.; Shankland, N.; Johnston, A. 10,11-Dihydrocarbamazepine (form III). *Acta Crystallographica Section E - Structure Reports Online* **2007**, *63* (2), o675-o677.
(46) Leech, C. K.; Florence, A. J.; Shankland, K.; Shankland, N.; Johnston, A. A low-temperature redetermination of cyheptamide. *Acta Crystallographica Section E - Structure Reports Online* **2007**, *63*, O205-O206.
(47) Polyzois, H.; Guo, R.; Srirambhatla, V. K.; Warzecha, M.; Prasad, E.; Turner, A.; Halbert, G. W.; Keating, P.; Price, S. L.; Florence, A. J. Crystal Structure and Twisted Aggregates of Oxcarbazepine Form III. *Crystal Growth & Design* **2022**. DOI: 10.1021/acs.cgd.2c00152.
(48) Zhou, T.; Li, F.; Fan, Y.; Song, W.; Mu, X.; Zhang, H.; Wang, Y. Hydrogen-bonded dimer stacking induced emission of aminobenzoic acid compounds. *Chemical Communications* **2009**, (22), 3199-3201.
(49) Sbit, M.; Dupont, L.; Dideberg, O.; Liegeois, J. F.; Delarge, J. Structure de l`acide diphenylamino-2-carboxylique. *Acta Crystallographica Section C* **1987**, *43* (5), 926-928.
(50) McConnell, J. F. FZ Company. N-(2, 3-xylyl) anthranilic acid, C15H15NO2 mefenamic acid. *Cryst.Struct.Commun* **1976**, *5*, 861-864.
(51) Fang, L.; Numajiri, S.; Kobayashi, D.; Ueda, H.; Nakayama, K.; Miyamae, H.; Morimoto, Y. Physicochemical and crystallographic characterization of mefenamic acid complexes with alkanolamines. *Journal of Pharmaceutical Sciences* **2004**, *93* (1), 144-154.
(52) Yang, X.; Sarma, B.; Myerson, A. S. Polymorph Control of Micro/Nano-Sized Mefenamic Acid Crystals on Patterned Self-Assembled Monolayer Islands. *Crystal Growth & Design* **2012**, *12* (11), 5521-5528.
(53) SeethaLekshmi, S.; Guru Row, T. N. Conformational Polymorphism in a Non-steroidal Anti-inflammatory Drug, Mefenamic Acid. *Crystal Growth & Design* **2012**, *12* (8), 4283-4289. DOI: 10.1021/cg300812v.
(54) Lee, E. H.; Byrn, S. R.; Carvajal, M. T. Additive-induced metastable single crystal of mefenamic acid. *Pharmaceutical Research* **2006**, *23* (10), 2375-2380.
(55) Andersen, K. V.; Larsen, S.; Alhede, B.; Gelting, N.; Buchardt, O. Characterization of two polymorphic forms of tolfenamic acid, N-(2-methyl-3-chlorophenyl)anthranilic acid: their crystal structures and relative stabilities. *Journal of the Chemical Society, Perkin Transactions 2* **1989**, (10), 1443-1447.
(56) Lopez-Mejias, V.; Kampf, J. W.; Matzger, A. J. Polymer-Induced Heteronucleation of Tolfenamic Acid: Structural Investigation of a Pentamorph. *Journal of the American Chemical Society* **2009**, *131* (13), 4554-4555.
(57) Delaney, S. P.; Smith, T. M.; Korter, T. M. Conformational origins of polymorphism in two forms of flufenamic acid. *Journal of Molecular Structure* **2014**, *1078*, 83-89. DOI: 10.1016/j.molstruc.2014.02.001.

(58) Lopez-Mejias, V.; Kampf, J. W.; Matzger, A. J. Nonamorphism in Flufenamic Acid and a New Record for a Polymorphic Compound with Solved Structures. *Journal of the American Chemical Society* **2012**, *134* (24), 9872-9875. DOI: 10.1021/ja302601f.
(59) Tyler, A. R.; Ragbirsingh, R.; McMonagle, C. J.; Waddell, P. G.; Heaps, S. E.; Steed, J. W.; Thaw, P.; Hall, M. J.; Probert, M. R. Encapsulated Nanodroplet Crystallization of Organic-Soluble Small Molecules. *Chem* **2020**, *6* (7), 1755-1765. DOI: 10.1016/j.chempr.2020.04.009.
(60) Krishna Murthy, H. M.; Vijayan, M. 2{[3-(Trifluoromethyl)phenyl]amino}-3-pyridinecarboxylic acid (niflumic acid). *Acta Crystallographica Section B* **1979**, *35* (1), 262-263.
(61) Rabinovich, D. Topochemistry. Part XXX. Crystal and molecular structures of chalcone. *Journal of the Chemical Society B: Physical Organic* **1970**, (0), 11-16. DOI: 10.1039/J29700000011.
(62) Wu, M. H.; Yang, X. H.; Zou, W. D.; Liu, W. J.; Li, C. Refinement of the crystal structure of (E)-1,3-diphenyl-2-propen-1-one, C15H12O. *Zeitschrift Fur Kristallographie-New Crystal Structures* **2006**, *221* (3), 323-324. DOI: 10.1524/ncrs.2006.0093.
(63) Shankland, N.; Florence, A. J.; Cox, P. J.; Sheen, D. B.; Love, S. W.; Stewart, N. S.; Wilson, C. C. Crystal morphology of ibuprofen predicted from single-crystal pulsed neutron diffraction data. *Chemical Communications* **1996**, (7), 855-856.
(64) Derollez, P.; Dudognon, E.; Affouard, F.; Danede, F.; Correia, N. T.; Descamps, M. Ab initio structure determination of phase II of racemic ibuprofen by X-ray powder diffraction. *Acta Crystallographica Section B* **2010**, *66* (1), 76-80. DOI: 10.1107/S0108768109047363.
(65) King, M. D.; Buchanan, W. D.; Korter, T. M. Understanding the Terahertz Spectra of Crystalline Pharmaceuticals: Terahertz Spectroscopy and Solid-State Density Functional Theory Study of (S)-(+)-Ibuprofen and (RS)-Ibuprofen. *Journal of Pharmaceutical Sciences* **2011**, *100* (3), 1116-1129. DOI: 10.1002/jps.22339.
(66) Hachuła, B. The nature of hydrogen-bonding interactions in nonsteroidal anti-inflammatory drugs revealed by polarized IR spectroscopy. *Spectrochimica Acta Part A: Molecular and Biomolecular Spectroscopy* **2018**, *188*, 189-196. DOI: 10.1016/j.saa.2017.07.005.
(67) Ravikumar, K.; Rajan, S. S.; Pattabhi, V.; Gabe, E. J. Structure of naproxen, C14H14O3. *Acta Crystallographica Section C* **1985**, *41* (2), 280-282. DOI: 10.1107/S0108270185003626.
(68) King, M. D.; Buchanan, W. D.; Korter, T. M. Application of London-type dispersion corrections to the solid-state density functional theory simulation of the terahertz spectra of crystalline pharmaceuticals. *Physical Chemistry Chemical Physics* **2011**, *13* (10), 4250-4259. DOI: 10.1039/c0cp01595d.
(69) Tang, G.-M.; Wang, J.-H.; Zhao, C.; Wang, Y.-T.; Cui, Y.-Z.; Cheng, F.-Y.; Ng, S. W. Multi odd–even effects on cell parameters, melting points, and optical properties of chiral crystal solids based on S-naproxen. *CrystEngComm* **2015**, *17* (38), 7258-7261. DOI: 10.1039/C5CE01345C.
(70) Caria, M. R.; Mohamed, R. Positive indentification of two orthorhombic polymorphs of sulfamerazine (C11H12N4O2S), their thermal analyses and structural comparison. *Acta Crystallographica Section B* **1992**, *48* (4), 492-498. DOI: doi:10.1107/S0108768192000910.
(71) Ravindra Acharya, K.; Kuchela, K. N.; Kartha, G. Crystal structure of sulfamerazine. *Journal of Crystallographic and Spectroscopic Research* **1982**, *12* (4), 369-376. DOI: 10.1007/BF01159053.
(72) Hossain, G. A new polymorph of sulfamerazine. *Acta Crystallographica Section E* **2006**, *62* (6), o2166-o2167. DOI: 10.1107/S1600536806014449.
(73) Direm, A.; Parlak, C.; El Bali, B.; Abdelbaky, M. S. M.; García-Granda, S. Experimental and computational insights into polymorphism in an antimicrobial sulfadrug: discovery of a novel monoclinic form of sulfamerazine. *Journal of the Iranian Chemical Society* **2024**, *21* (11), 2799-2815. DOI: 10.1007/s13738-024-03110-x.
(74) Chickos, J. S.; Gavezzotti, A. Sublimation Enthalpies of Organic Compounds: A Very Large Database with a Match to Crystal Structure Determinations and a Comparison with Lattice Energies. *Crystal Growth & Design* **2019**, *19* (11), 6566-6576. DOI: 10.1021/acs.cgd.9b01006.
(75) Perlovich, G. L.; Raevsky, O. A. Sublimation of Molecular Crystals: Prediction of Sublimation Functions on the Basis of HYBOT Physicochemical Descriptors and Structural Clusterization. *Crystal Growth & Design* **2010**, *10* (6), 2707-2712. DOI: 10.1021/cg1001946.
(76) Ouvrard, C.; Mitchell, J. B. O. Can we predict lattice energy from molecular structure? *Acta Crystallographica Section B - Structural Science* **2003**, *59*, 676-685.
(77) Chickos, J. S. A protocol for correcting experimental fusion enthalpies to 298.15K and it's application in indirect measurements of sublimation enthalpy at 298.15K. *Thermochimica Acta* **1998**, *313* (1), 19-26. DOI: 10.1016/S0040-6031(97)00433-4.

(78) Torres, L. A.; Campos, M.; Martínez, M.; Rojas, A. The thermochemistry of coronene revisited. *The Journal of Chemical Thermodynamics* **2009**, *41* (8), 957-965. DOI: 10.1016/j.jct.2009.03.010.
(79) Roux, M. V.; Temprado, M.; Chickos, J. S.; Nagano, Y. Critically Evaluated Thermochemical Properties of Polycyclic Aromatic Hydrocarbons. *Journal of Physical and Chemical Reference Data* **2008**, *37* (4), 1855-1996. DOI: 10.1063/1.2955570.
(80) Sabbah, R.; Tabet, D.; Bélaadi, S. Étude thermodynamique de l'acridine. *Thermochimica Acta* **1994**, *247* (2), 201-207. DOI: 10.1016/0040-6031(94)80121-5.
(81) Ribeiro da Silva, M. A. V.; Santos, C. P. F.; Monte, M. J. S.; Sousa, C. A. D. Thermochemicalstudies of phthalimide and two N-alkylsubstitutedphthalimides (ALKYL=ETHYL AND n-PROPYL). *Journal of Thermal Analysis and Calorimetry* **2006**, *83* (3), 533-539. DOI: 10.1007/s10973-005-7448-3.
(82) Matos, M. A. R.; Miranda, M. S.; Morais, V. M. F.; Liebman, J. F. Saccharin: a combined experimental and computational thermochemical investigation of a sweetener and sulfonamide. *Molecular Physics* **2005**, *103* (2-3), 221-228. DOI: 10.1080/00268970512331316175.
(83) Szterner, P.; Kaminski, M.; Zielenkiewicz, A. Vapour pressures, molar enthalpies of sublimation and molar enthalpies of solution in water of five halogenated derivatives of uracil. *The Journal of Chemical Thermodynamics* **2002**, *34* (7), 1005-1012. DOI: 10.1006/jcht.2000.0987.
(84) Brunetti, B.; Portalone, G.; Piacente, V. Sublimation Thermodynamic Parameters for 5-Fluorouracil and Its 1-Methyl and 1,3-Dimethyl Derivatives from Vapor Pressure Measurements. *Journal of Chemical & Engineering Data* **2002**, *47* (1), 17-19. DOI: 10.1021/je010037e.
(85) Surov, A. O.; Terekhova, I. V.; Bauer-Brandl, A.; Perlovich, G. L. Thermodynamic and Structural Aspects of Some Fenamate Molecular Crystals. *Crystal Growth & Design* **2009**, *9* (7), 3265-3272.
(86) Perlovich, G. L.; Surov, A. O.; Bauer-Brandl, A. Thermodynamic properties of flufenamic and niflumic acids - Specific and non-specific interactions in solution and in crystal lattices, mechanism of solvation, partitioning and distribution. *Journal of Pharmaceutical and Biomedical Analysis* **2007**, *45* (4), 679-687.
(87) Perlovich, G. L.; Kurkov, S. V.; Hansen, L. K.; Bauer-Brandl, A. Thermodynamics of Sublimation, Crystal Lattice Energies, and Crystal Structures of Racemates and Enantiomers: (+)- and (±)-Ibuprofen. *Journal of Pharmaceutical Sciences* **2004**, *93* (3), 654-666. DOI: 10.1002/jps.10586.
(88) Perlovich, G. L.; Kurkov, S. V.; Kinchin, A. N.; Bauer-Brandl, A. Thermodynamics of solutions III: comparison of the solvation of (+)-naproxen with other NSAIDs. *European Journal of Pharmaceutics and Biopharmaceutics* **2004**, *57* (2), 411-420. DOI: 10.1016/j.ejpb.2003.10.021.
